# Sex difference in the burden of rheumatic heart disease: Insights from the Global Burden of Disease Study 2021

**DOI:** 10.1371/journal.pone.0334914

**Published:** 2025-10-22

**Authors:** Shushu Jiang, Jiakai Zhang, Menghao Shi, Yin Dong, Zhaohui Wang, Cheng Wang

**Affiliations:** 1 Department of Cardiology, Union Hospital, Tongji Medical College, Huazhong University of Science and Technology, Wuhan, China; 2 Clinic Center of Human Gene Research, Union Hospital, Tongji Medical College, Huazhong University of Science and Technology, Wuhan, China; 3 Hubei Key Laboratory of Metabolic Abnormalities and Vascular Aging Huazhong University of Science and Technology, Wuhan, China; 4 Xinxiang Medical University, Third Clinical College of Xinxiang Medical University, Xinxiang, China; 5 Hubei clinical research center for metabolic and cardiovascular disease, Huazhong University of Science and Technology, Wuhan, China; 6 Cardiovascular Center, Liyuan Hospital, Tongji Medical College, Huazhong University of Science and Technology, Wuhan, China; 7 Department of Rheumatology and Immunology, Union Hospital, Tongji Medical College, Huazhong University of Science and Technology, Wuhan, China; Tehran University of Medical Sciences, IRAN, ISLAMIC REPUBLIC OF

## Abstract

**Introduction:**

Rheumatic heart disease (RHD) shows significant sex differences in disease burden. This study assesses these differences using data from the Global Burden of Disease Study 2021 (GBD 2021).

**Methods:**

We extracted sex-specific indicators for RHD from the GBD database, including disability-adjusted life years (DALYs), mortality, and prevalence. Trends were analyzed using estimated annual percentage change (EAPC), and sex differences were assessed via female-by-male ratios.

**Results:**

From 1990 to 2021, females consistently had higher age-standardized DALYs (ASDR), mortality (ASMR), and prevalence rates (ASPR) than males. These differences were particularly pronounced in specific regions and age groups. In 2021, female ASDR and ASMR in Andorra were over three times higher than males, while in the Cook Islands, they were less than half of males’ rates. In the United States Virgin Islands, females aged 10–19 had an ASMR only 0.01 times that of males, whereas in the United Arab Emirates, females aged 70–89 had ASDR and ASMR five times higher than males. Overall, the female-by-male ratios in ASDR, ASMR, and ASPR have shown a yearly decline. However, these ratios are positively correlated with the Sociodemographic Index (SDI), with correlation coefficients of 0.1 for ASDR, 0.22 for ASMR, and 0.47 for ASPR.

**Conclusion:**

Our study reveals a persistent global sex disparity in RHD burden from 1990 to 2021, with females generally experiencing a heavier burden. These findings underscore the need for sex-specific approaches in RHD prevention and treatment and further research into the underlying factors driving these disparities.

## Introduction

Rheumatic heart disease (RHD) is a significant public health disease, originating from Group A streptococcal infections, RHD can lead to severe valve damage, significant cardiac morbidity, and premature death [1]. According to the previous Global Burden of Disease (GBD) study, RHD affects approximately 400,000 people globally, with around 310,000 deaths in 2019 [2], and ranks 25th in global disease burden and 5th among cardiovascular diseases in 2021 [3,4].

Prior studies have suggested that rheumatic heart disease (RHD) exhibits substantial sex differences, with females being more prone to RHD incidence due to a combination of physiological, socioeconomic, environmental, autoimmune, and sociocultural factors [5,6]. Global data indicate that in 2015, females had up to twice the incidence rate of males in some regions, such as Australia [7]. In 2019, the age-standardized incidence rate for females was 40.6 (95%UI: 31.1–50.4) per 100,000, and for males was 34.3 (95%UI: 26.3–42.9) per 100,000 [8]. From 1990 to 2019, the age-standardized prevalence, DALYs, and mortality rates of RHD were also higher in females than in males [8]. Additionally, compared to males, females with RHD are generally older, have more symptoms and comorbidities, and have lower socioeconomic status [9].

However, there is still a lack of research on the distribution of sex differences in RHD burden among regions and countries, the correlation between sex differences and SDI, and the changing trend of RHD sex differences over the past 32 years. This study aims to comprehensively analyze and compare the sex differences in RHD burden and their relationship with socioeconomic development status and the trend from 1990 to 2021 using the latest GBD data by level of socio-demographic index (SDI), country, and territory across all age groups, which may be crucial for policymakers to guide cost-effective interventions and resource allocation.

## Materials and methods

### Data source and disease definition

This study examines the burden of rheumatic heart disease (RHD) using data from the Global Burden of Disease Study 2021 (GBD 2021). GBD 2021 provides estimates of the burden of 371 diseases and injuries across 21 regions and 204 countries from 1990 to 2021. All this data can be freely accessed through the Global Health Data Exchange (https://ghdx.healthdata.org/gbd-2021/sources)(10). RHD, which is classified as a Level 4 cause in GBD 2021, is a cardiac condition mainly affecting heart valves, resulting from acute rheumatic fever caused by Group A Streptococcus infection [GBD cause code B.2.1, ICD-10 codes: 101–101.9, 102.0, 105–109.9] [10,11].

### Socio-demographic index (SDI)

In 2015, the Institute for Health Metrics and Evaluation (IHME) brought in an index, which is used to evaluate the development level of countries or regions by connecting social development with population health outcomes, known as the Socio - demographic Index (SDI). It is computed as the geometric mean of three indicators: the total fertility rate among those under 25, the average education level of those aged 15 and older, and the lag – distributed per capita income. In GBD 2021, SDI values were ranged from 0 to 100. 0 stands for the lowest level of income and education along with the highest fertility, and 100 stands for the highest level of income and education as well as the lowest fertility. Five SDI regions were used to classify the 204 countries and territories, namely low, low – middle, middle, high – middle, and high [10].

### Analysis metrics

Age-standardized DALYs rates (ASDR), age-standardized mortality rates (ASMR) and age-standardized prevalence rates (ASPR) of RHD were screened from the GBD database. Trends in indicators from 1990 to 2021 were tracked with the estimated annual percentage change (EAPC), which is calculated on the basis of fitting the natural logarithm rate of the regression model. with time as a variable. The formula is ,and . In this model, the variable x stands for the year. The natural logarithm of rates is represented by y. The intercept is represented by α. Beta refers to slope. ε represents the random error. The interpretation of trend results is based on the 95% CI. If the 95% CI of the corresponding EAPC estimate is > 0, the age – standardized indicator exhibits an upward trend; if < 0, it shows a decreasing trend; If the 95% confidence intervals contain 0, it means there is no statistically significant difference in trend alterations [11]. The relative sex difference (female by male ratio) was calculated by dividing male ASR by female ASR. If the ratio is > 1, it indicates that the female ASR is higher than males, and the further the ratio is from 1, the greater the sex difference [12].

### Statistical analysis and visualization

R statistical software (version 4.3.3) was utilized for performing the statistical analysis, with a P value < 0.05 was regarded as being statistically significant.

## Results

### Global-level sex difference in RHD burden

**Fig 1:**
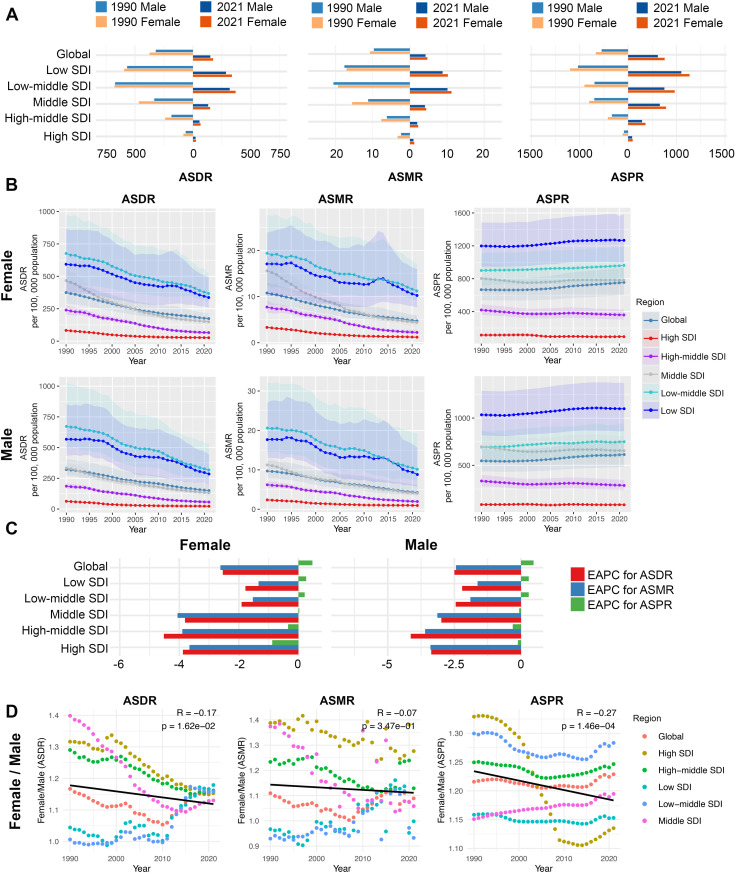
RHD burden of female and male in global and five SDI regions. A: The ASDR, ASMR, ASPR of RHD in female and male in 1990 and 2021. B: The ASDR, ASMR, ASPR of RHD in female and male from 1990 to 2021. C: The EAPC of ASDR, ASMR, ASPR in female and male from 1990 to 2021. D: The associations between the year and Female/ Male (ASDR), Female/ Male (ASMR) and Female/ Male (ASPR) in RHD across global and 5 SDI regions. RHD = Rheumatic heart disease, SDI = Socio-Demographic Index, ASDR = age-standardized DALYs rates, ASMR = age-standardized mortality rates, ASPR = age-standardized prevalence rates, EAPC = estimated annual percentage change, Female/ Male = the female by male ratio.

In 1990, age-standardized rates of DALYs (ASDR) of RHD was 374.7 (95% UI: 290.09, 461.87) in females and 321.09 (95% UI: 269.51, 407.53) in males, with females having 1.17 times the rate of males. By 2021, these rates were 173.99 (95% UI: 148.21, 211.13) in females and 150.11 (95% UI: 125.15, 204.97) in males, with females having 1.16 times the rate of males (S1 Table and Fig 1A). For age-standardized mortality rates (ASMR), females had rates of 10.77 (95% UI: 8.33, 13.01) in 1990 and 4.72 (95% UI: 4.01, 5.82) in 2021, compared to 9.71 (95% UI: 8.06, 12.33) in 1990 and 4.22 (95% UI: 3.54, 6.19) in 2021 for males. Females had 1.11 and 1.12 times the male rates in 1990 and 2021, respectively (S2 Table and Fig 1A). For age-standardized prevalence rates (ASPR), females had rates of 666.29 (95% UI: 538.76, 813.74) in 1990 and 754.77 (95% UI: 600.82, 936.66) in 2021, compared to 548.00 (95% UI: 436.06, 673.93) in 1990 and 614.20 (95% UI: 482.58, 761.31) in 2021 for males. Females had 1.22 and 1.23 times the male rates in 1990 and 2021, respectively (S3 Table and Fig 1A).

Globally, ASDR and ASMR significantly decreased from 1990 to 2021 in both sexes, with a slightly greater reduction in females. The EAPC for ASDR was −2.5 (95% CI: −2.56, −2.45) in males and −2.54 (95% CI: −2.61, −2.46) in females (S1 Table and Fig 1B). For ASMR, the EAPC was −2.44 (95% CI: −2.53, −2.36) in males and −2.62 (95% CI: −2.67, −2.57) in females (S2 Table and Fig 1B). Conversely, ASPR increased in both sexes over the same period, with an EAPC of 0.47 (95% CI: 0.42, 0.51) in males and 0.47 (95% CI: 0.42, 0.52) in females, with equal increase rates in both sexes (S3 Table and Fig 1B). Overall, these findings indicate that there has been a significant global reduction in ASDR and ASMR, while there has also been a slight but significant increase in ASPR in the burden of rheumatic heart disease from 1990 to 2021.

### SDI regional-level sex difference in RHD burden

In 2021, the highest ASDR and deaths ASMR in females were in the low-middle SDI region (ASDR: 367.82, 95% UI: 303.08–519.08; ASMR: 11.22, 95% UI: 9.14–16.96), while the highest ASPR was in the low SDI region (ASPR: 1267.26, 95% UI: 999.84–1584.18). Conversely, the lowest rates for ASDR, ASMR, and ASPR in females were all in the High SDI region (ASDR: 26.11, 95% UI: 23.28–28.82; ASMR: 1.19, 95% UI: 0.98–1.32; ASPR: 94.49, 95% UI: 86.4–103.43). Similar patterns were observed in males, with the highest ASDR and ASMR in the low-middle SDI region and the highest ASPR in the low SDI region. The lowest rates for all three metrics in males were in the High SDI region (S1, S2, S3 Tables and Fig 1A).

From 1990 to 2021, the highest ASDR and ASMR in both sexes were in the low-middle SDI region in most years and the highest ASPR were consistently in the low SDI region. While the lowest ASDR, ASMR and ASPR in both sexes were consistently in the High SDI region (S1, S2, S3 Tables and Fig 1B).

From 1990 to 2021, ASDR, ASMR, and ASPR generally decreased in females, except for an increase in ASPR in the low, low-middle, and middle SDI regions. The most significant decreases were in the high-middle SDI region (ASDR: EAPC −4.52, 95% CI: −4.67 to −4.37; ASMR: EAPC −4.06, 95% CI: −4.15 to −3.96; ASPR: EAPC −0.88, 95% CI: −1.03 to −0.73). In males, these rates decreased in all regions except for an increase in ASPR in the low and low-middle SDI regions, with the most significant decreases in the high-middle SDI region (ASDR: EAPC −4.14, 95% CI: −4.26 to −4.02; ASMR: EAPC −3.6, 95% CI: −3.73 to −3.46; ASPR: EAPC −0.35, 95% CI: −0.43 to −0.27) (S1, S2, S3 Tables and Fig 1C).

Over the past 32 years, the disease burden was consistently higher in females than in males across all SDI regions, as indicated by the female by male ratio being greater than 1 in most years (Female/ Male > 1). The female by male ratio of ASDR and ASPR showed a decreasing trend (ASDR: p = 0.0162, ASPR: p = 0.000146) and the female by male ratio of ASDR in all five SDI regions tended toward 1.15 from 1990 to 2021. Notably, before 2001, the High SDI region had the highest female by male ratio of ASPR, but from 2000 to 2009, the gender difference in ASPR in the High SDI region sharply decreased, becoming the lowest among the five regions by 2008 (Fig 1D).

Overall, despite a global decline in RHD burden, the highest burden remained in the low and low-middle SDI regions. Additionally, although the gender difference in ASDR and ASMR showed a decreasing trend over the past 32 years, females continued to experience a higher disease burden than males across all five SDI regions.

### GBD regional-level sex difference in RHD burden

In 2021, among the 21 GBD regions, Oceania had the highest ASDR in both males (522.58, 95% UI: 281.96, 1033.97) and females (529.33, 95% UI: 297.26, 1124.69), with females having 1.01 times the rate of males. South Asia had the highest ASMR in both sexes, with rates of 13.86 (95% UI: 10.32, 25.28) in males and 15.91 (95% UI: 12.84, 23.65) in females, where females had 1.15 times the rate of males. The highest ASPR was in Central Sub-Saharan Africa for males (1677.11, 95% UI: 1297.49, 2097.18) and Eastern Sub-Saharan Africa for females (1707.03, 95% UI: 1342.58, 2143.18) (S1, S2, S3 Tables and S1A Fig).

From 1990 to 2021, ASDR and ASMR decreased globally across all 21 GBD regions. The largest decreases were in Eastern Europe (ASDR: EAPC −6.76, 95% CI: −7.3, -6.23; ASMR: EAPC −5.62, 95% CI: −5.78, -5.46) and Central Latin America (ASMR: EAPC −5.86, 95% CI: −6.02, -5.7). However, ASPR increased in over half of the regions, with the highest increases in South Asia for males (EAPC 0.28, 95% CI: 0.21, 0.35) and Western Sub-Saharan Africa for females (EAPC 0.27, 95% CI: 0.25, 0.3). The largest ASPR decrease was in Eastern Europe (EAPC −1.8, 95% CI: −1.87, -1.72) (S1, S2, S3 Tables and S1B Fig).

**Fig 2:**
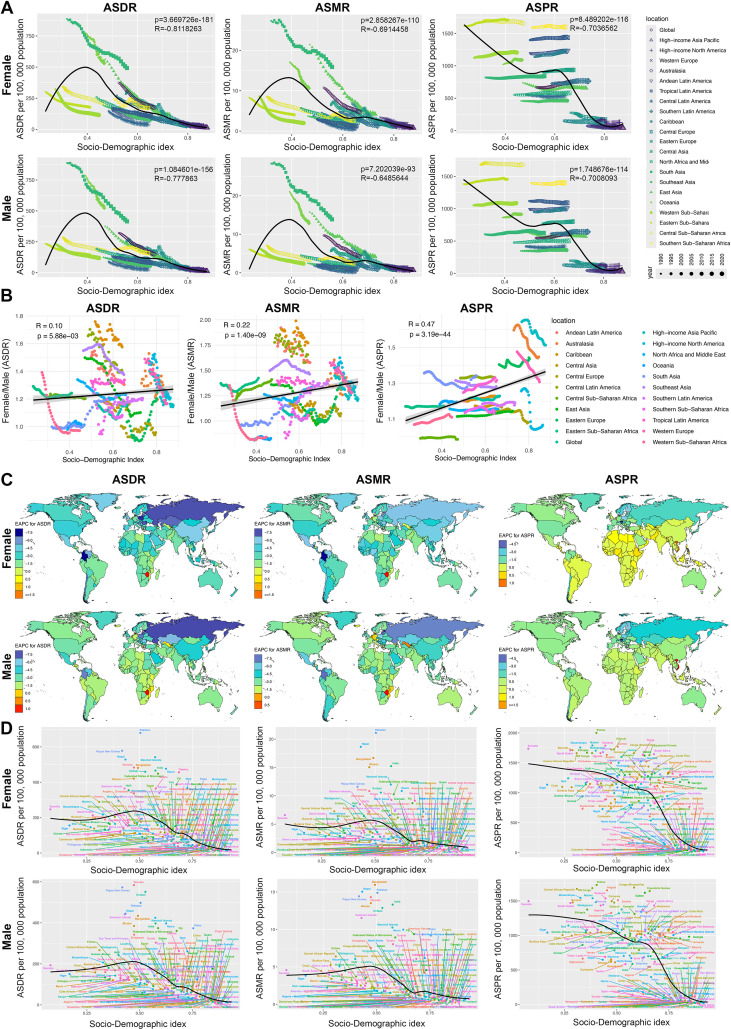
RHD burden of female and male in 21 GBD regions and 204 countries. A: The associations between the SDI and ASDR, ASMR, ASPR of RHD in females and males across 21 GBD regions. B: The associations between the SDI and Female/ Male (ASDR), Female/ Male (ASMR) and Female/ Male (ASPR) of RHD across 21 GBD regions. C: The EAPC of ASDR, ASMR, ASPR in female and male across 204 countries from 1990 to 2021. D: The associations between the SDI and ASDR, ASMR, ASPR of RHD in females and males across 204 countries in 2021. RHD = Rheumatic heart disease, SDI = Socio-Demographic Index, ASDR = age-standardized DALYs rates, ASMR = age-standardized mortality rates, ASPR = age-standardized prevalence rates, EAPC = Estimated Annual Percentage Change, Female/ Male = the female by male ratio.

The correlation models of SDI with age-standardized rates (ASR) were similar for both sexes. When SDI was below 0.4, ASDR and ASMR positively correlated with SDI. Above 0.4, they generally showed a negative correlation. ASPR was mostly negatively correlated with SDI, with an upward trend when SDI was between 0.5 and 0.6 (Fig 2A).

Over the past 32 years, in most GBD regions, females had a higher disease burden than males, with sex differences in ASDR, ASMR, and ASPR positively correlating with SDI. As SDI increased, so did the gender gap, with High-income Asia Pacific showing the largest ASPR gender difference (Fig 2B). In 2021, the Caribbean had the highest sex difference in ASDR and ASMR, with females at 1.58- and 1.71-times male rates, respectively. Eastern Europe had the largest sex difference in ASPR, with females at 1.44 times male rates. Female ASDR and ASMR were higher than male rates in most regions, except Central Europe and Western Sub-Saharan Africa for ASDR, and Oceania and Western Sub-Saharan Africa for ASMR. Female ASPR was higher in all regions except Central Sub-Saharan Africa (S1, S2, S3 Tables and S1A Fig). Overall, these findings highlight significant regional disparities in RHD burden, with females consistently bearing a higher burden across most regions and a positive correlation between sex differences and SDI.

### Country-level sex difference in RHD burden

In 2021, Pakistan had the highest ASDR and ASMR in females, while Colombia had the lowest ASDR and Guatemala had the lowest ASMR. For males, Vanuatu had the highest ASDR and ASMR, while Andorra had the lowest ASDR and Guatemala had the lowest ASMR. Eritrea had the highest ASPR in both sexes, while Finland had the lowest ASPR in females and Sweden had the lowest in males (S4 and S5 Tables).

From 1990 to 2021, most countries saw a decline in ASDR and ASMR for both sexes. Female ASDR increased only in Lesotho and Zimbabwe (EAPCs: 0.54 (95% CI: 0.14, 0.95) and 1.91 (95% CI: 1.24, 2.57)), with the largest decrease in Colombia (EAPC: −7.52 (95% CI: −7.93, −7.1)). For males, ASDR rose only in the Northern Mariana Islands and Zimbabwe (EAPCs: 0.12 (95% CI: −0.04, 0.28) and 1.04 (95% CI: 0.66, 1.42)), with the largest drop in Estonia (EAPC: −8.36 (95% CI: −8.88, −7.83)). For ASMR, female rates increased in the United Arab Emirates, Uzbekistan, Lesotho, Netherlands, and Zimbabwe (largest increase in Zimbabwe: EAPC 1.86 (95% CI: 1.08, 2.64)), with the largest decrease in Colombia (EAPC −8.37 (95% CI: −8.95, −7.8)). Male rates rose in Uzbekistan, Northern Mariana Islands, and Zimbabwe (largest increase in Zimbabwe: EAPC 0.68 (95% CI: 0.28, 1.07)), with the largest drop in Estonia (EAPC −8.74 (95% CI: −9.28, −8.2)). For ASPR, female rates fell in 48% of countries (largest decrease in Austria: EAPC −5.2 (95% CI: −5.56, −4.83)), with the largest increase in Fiji (EAPC 1.26 (95% CI: 0.89, 1.63)). Male rates decreased in 55.9% of countries (largest decrease in Austria: EAPC −5.37 (95% CI: −5.8, −4.95)), with the largest increase in Viet Nam (EAPC 1.99 (95% CI: 1.59, 2.4)) (S6 Table and Fig 2C). The correlation patterns of SDI with ASR were similar between males and females. When SDI was between 0 and 0.5, ASDR and ASMR increased with SDI. However, when SDI exceeded 0.5, ASDR and ASMR decreased. ASPR was generally negatively correlated with SDI, with a sharp decline when SDI exceeded 0.6 (Fig 2D).

**Fig 3:**
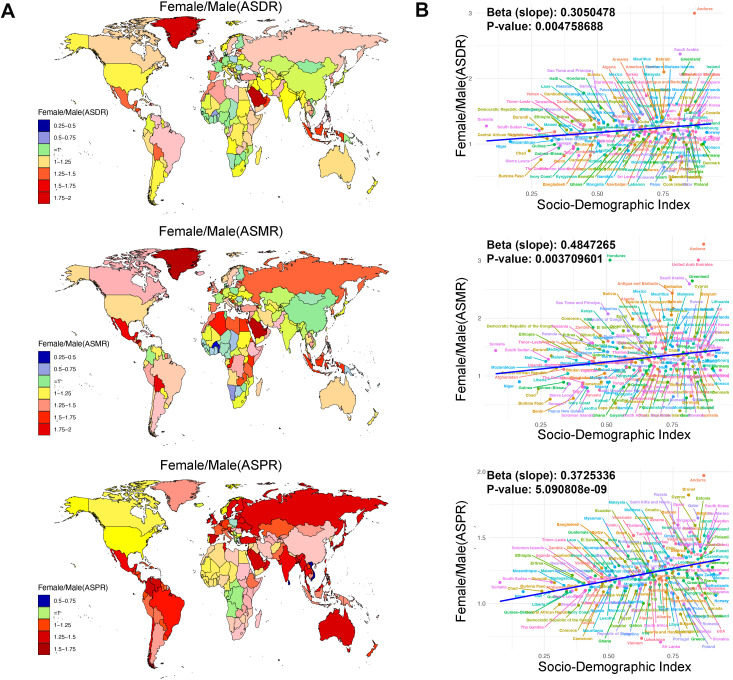
The gender difference of RHD burden in 204 countries. A: The Female/ Male (ASDR), Female/ Male (ASMR) and Female/ Male (ASPR) of RHD across 204 countries in 2021. B: The associations between the SDI and The Female/ Male (ASDR), Female/ Male (ASMR) and Female/ Male (ASPR) of RHD across 204 countries in 2021. RHD = Rheumatic heart disease, SDI = Socio-Demographic Index, ASDR = age-standardized DALYs rates, ASMR = age-standardized mortality rates, ASPR = age-standardized prevalence rates, Female/ Male = the female by male ratio.

Globally, in only 17% of countries was the ASDR higher in males than in females, with the most significant difference observed in the Cook Islands, where the female ASDR was 0.45 times that of males. Similarly, in just 19% of countries, the ASMR was higher in males, with the largest disparity also in the Cook Islands, where the female ASMR was 0.40 times that of males. For ASPR, only 11% of countries had higher rates in males, with the greatest difference in Sri Lanka, where the female ASPR was 0.71 times that of males. Conversely, in countries where females had higher ASDR, ASMR, and ASPR than males, the most pronounced differences were in Andorra, where the female ASDR was 3 times that of males, the female ASMR was 3.28 times that of males, and the female ASPR was 1.97 times that of males (S5 Table and Fig 3A). Additionally, the relative differences in ASDR, ASMR, and ASPR between females and males (Female/Male) are positively correlated with the Socio-Demographic Index (SDI) (Fig 3B). Overall, these findings highlight significant global disparities in the burden of rheumatic heart disease, with notable variations in ASDR, ASMR, and ASPR across countries and sexes.

### Sex differences across all age groups

**Fig 4:**
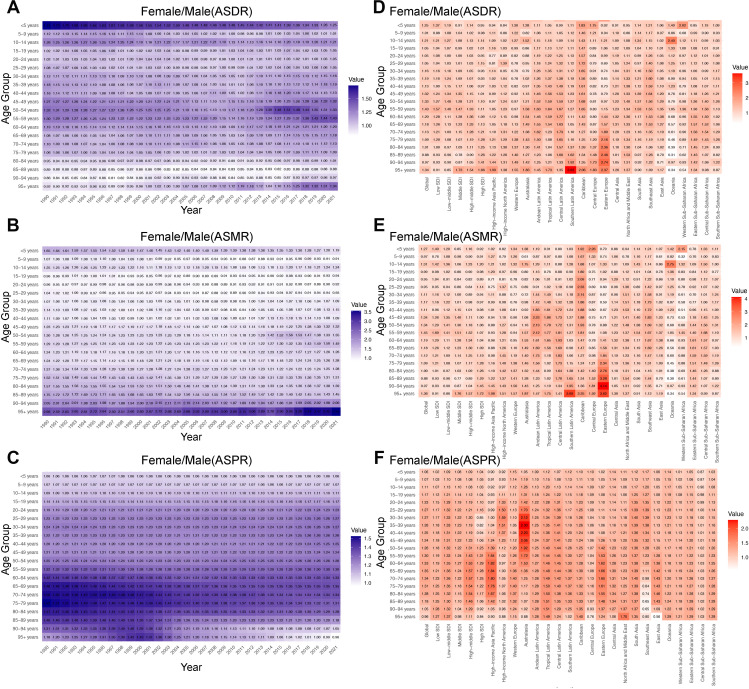
Sex Differences of RHD burden in Different Age Groups worldwide. A-C: The global Female/ Male (ASDR), Female/ Male (ASMR) and Female/ Male (ASPR) of RHD from 1990 to 2021. D-F: The Female/ Male (ASDR), Female/ Male (ASMR) and Female/ Male (ASPR) of RHD across five SDI regions and 21 GBD regions in 2021. RHD = Rheumatic heart disease, SDI = Socio-Demographic Index, ASDR = age-standardized DALYs rates, ASMR = age-standardized mortality rates, ASPR = age-standardized prevalence rates, Female/ Male = the female by male ratio.

Except for the age group of 80–94 years, from 1990 to 2021, the ASDR of females was generally higher than that of males across all age groups. The gender difference in ASDR in the age group “<5 years” continued to decline from 1990 to 2021, decreasing from 1.74 in 1990 (the highest among all age groups) to 1.25. The gender difference in ASDR in the “95+ years” age group showed an overall upward trend, increasing from 0.92 in 1990 to 1.34 in 2021 (Fig 4A). Except for the age group of 15–29 years, from 1990 to 2021, the ASMR of females was generally higher than that of males across all age groups. The gender difference in the “95+ years” age group remained the largest over the past 32 years and showed an overall upward trend, increasing from 2.66 in 1990 to 3.55 in 2021 (Fig 4B). From 1990 to 2021, in almost all years and age groups, the ASPR of females was consistently higher than that of males, and the gender difference in ASPR among people aged 60–89 showed an overall downward trend (Fig 4C).

In 2021, the distribution of gender differences in ASDR and ASMR was similar across the 5 SDI regions and 21 GBD regions globally. The largest gender difference in ASDR was found in the “95+ years” age group in Southern Latin America (Female/Male: 3.63). In addition, in Oceania, the gender difference in ASDR among people aged over 45 was generally less than 1, indicating that the ASDR burden of males was greater than that of females, with a gender difference value of only 0.31 in the 85–89 age group (Fig 4D). The largest gender difference in ASMR was found in the 90–94 age group in Eastern Europe (Female/Male: 4.14). Similarly, in Oceania, the gender difference in ASMR among people aged over 45 was generally less than 1, with a gender difference value of only 0.30 in the 85–89 age group (Fig 4E). However, in 2021, the overall ASPR of females was greater than that of males, with the largest difference found in the 35–39 age group in Australasia (Female/Male: 2.30) (Fig 4F).

In 2021, among 204 countries worldwide, the burden of RHD, as measured by DALYs, mortality, and prevalence, was higher in females than in males for the majority of nations. However, in the age groups of “5-9 years,” “15-19 years,” “20-24 years,” and “25-29 years,” fewer than 50% of countries reported a higher female mortality burden compared to males (S2 Fig and S7 Table). Additionally, it is noteworthy that in the United States Virgin Islands, the male mortality burden significantly exceeded that of females in the age groups of “10-14 years” and “15-19 years” (with a Female-by-Male ratio of 0.01). In the United Arab Emirates, females in the age group of 70–89 years experienced DALYs and mortality more than five times greater than that of males (S8 Table). Overall, across major regions and countries globally, the DALYs, mortality, and prevalence burden of RHD in females was greater than that in males across most age groups. Notably, the gender difference in ASDR and ASMR of RHD in the “95+ years” age group showed a continuous increasing trend from 1990 to 2021.

## Discussion

Using the GBD 2021 database, this study found that from 1990 to 2021, global DALYs and mortality rates for RHD significantly decreased in both sexes, while prevalence increased in Low SDI and Low-middle SDI regions. This increase may be driven by population growth and advancements in diagnostic technologies [13]. Over the past 32 years, Low-middle SDI and Low SDI regions consistently had the highest disease burden, while High SDI regions had the lowest. This may be due to the better prevention and control of RHD in High SDI regions, which have more developed economies, abundant medical resources, and stronger public health system.

While previous studies have shown that females have a higher RHD burden than males, our study confirms this and reveals some new and interesting findings. First, the overall changes in RHD burden were similar between sexes. However, the female prevalence in High SDI regions had an EAPC 7.33 times that of males: −0.88 (95% CI: −1.03, −0.73) in females versus −0.12 (95% CI: −0.22, −0.02) in males. Notably, before 2001, High SDI regions had the highest female-to-male ratio of ASPR, but from 2000 to 2009, this gender difference sharply decreased, becoming the lowest among all regions by 2008. This may be due to targeted health policies and initiatives for females in High SDI regions in the early 2000s, which focused on preventing and managing chronic diseases like cardiovascular diseases, thereby improving female health and reducing RHD sex differences [14].

Second, among the 204 countries globally, DALYs and mortality rates for RHD significantly decreased in both sexes from 1990 to 2021, with the most significant declines in Colombia for females and Estonia for males. However, Zimbabwe experienced a significant increase in RHD burden over the past 32 years in both sexes. In over 80% of countries, females had a higher disease burden than males, with the largest difference in Andorra (female burden over three times that of males). Conversely, in the Cook Islands, female burden was only 0.4 times that of males. Focusing on Zimbabwe, Andorra, and the Cook Islands could provide new insights for RHD prevention and treatment and for reducing RHD sex differences.

In terms of age-specific sex differences, females had a higher disease burden than males across all age groups globally. The sex difference in mortality in the “95+ years” age group was the highest and increased continuously from 2.88 times in 1990 to 3.55 times in 2021. In 2021, females had a significantly higher burden than males in the “95+ years” age group in Southern Latin America (Female/ Male of ASDR: 3.63, Female/ Male of ASMR: 3.69) and the over-75 age group in Eastern Europe (Female/ Male ranging from 2.16 to 4.14). Conversely, males had a higher burden than females in the 75–89 age group (Female/ Male ranging from 0.30 to 0.46) and over-95 age group (Female/ Male of ASDR and ASMR: 0.34) in Oceania, and the 5–9 age group in Western Sub-Saharan Africa (Female/ Male of ASMR: 0.32). Notably, in the United States Virgin Islands, females aged 10–19 years had a burden only 0.01 times that of males, while in the United Arab Emirates, females aged 70–89 years had a burden 5 times that of males. These findings highlight the significant age-related sex differences, especially in younger and older age groups.

Regarding the correlation of sex differences with time and SDI, global sex differences in DALYs and prevalence of RHD were negatively correlated with time, indicating a decreasing trend from 1990 to 2021. However, sex differences in RHD were positively correlated with SDI, indicating an increasing trend with higher SDI. This highlights the importance of developing sex-specific health policies to address RHD burden, particularly in High SDI regions.

## Conclusions

In conclusion, our study indicates that, despite slight improvements, females consistently experienced a higher RHD burden compared to males from 1990 to 2021. Additionally, the correlation between RHD sex differences and SDI, along with disparities across age groups and countries, underscores the necessity for gender-targeted health policies to mitigate these differences. A prompt and collaborative global response is crucial to address the ongoing and future gender disparities in RHD.

## Supporting information

S1 Fig RHD burden of female and male in 21 GBD regions. A: The ASDR, ASMR, ASPR of RHD in female and male in 1990 and 2021 in 21GBD regions. B: The EAPC of ASDR, ASMR, ASPR in female and male from 1990 to 2021 in 21 GBD regions.
(TIF)

S2 Fig The percentage of countries which the female by male ratio beyond 1. Female/ Male = the female by male ratio.
(TIF)

S1 Table The ASDR and the EAPC of ASDR in RHD by gender. (DOCX)

S2 Table The ASMR and the EAPC of ASMR in RHD by gender. (DOCX)

S3 Table The ASPR and the EAPC of ASPR in RHD by gender. (DOCX)

S4 Table Global Extreme Values of ASDR, ASMR, and ASPR by Sex in 2021 across 204 countries. (DOCX)

S5 Table The ASDR, ASMR, ASPR in female and male and the gender difference of RHD across 204 countries. (DOCX)

S6 Table The EAPC of ASDR, ASMR, ASPR in female and male and the gender difference of RHD across 204 countries. (DOCX)

S7 Table Global Values of Female/Male in 2021 across all age groups among 204 countries. (DOCX)

S8 Table Global Extreme Values of Female/Male in 2021 across 204 countries. (DOCX)
