# Supplementary figures and images for: Sex difference in the burden of rheumatic heart disease: Insights from the Global Burden of Disease Study 2021
Source: PLoS One. 2025 Oct 22;20(10):e0334914. doi: 10.1371/journal.pone.0334914 (PMC12543145; doi:10.1371/journal.pone.0334914)

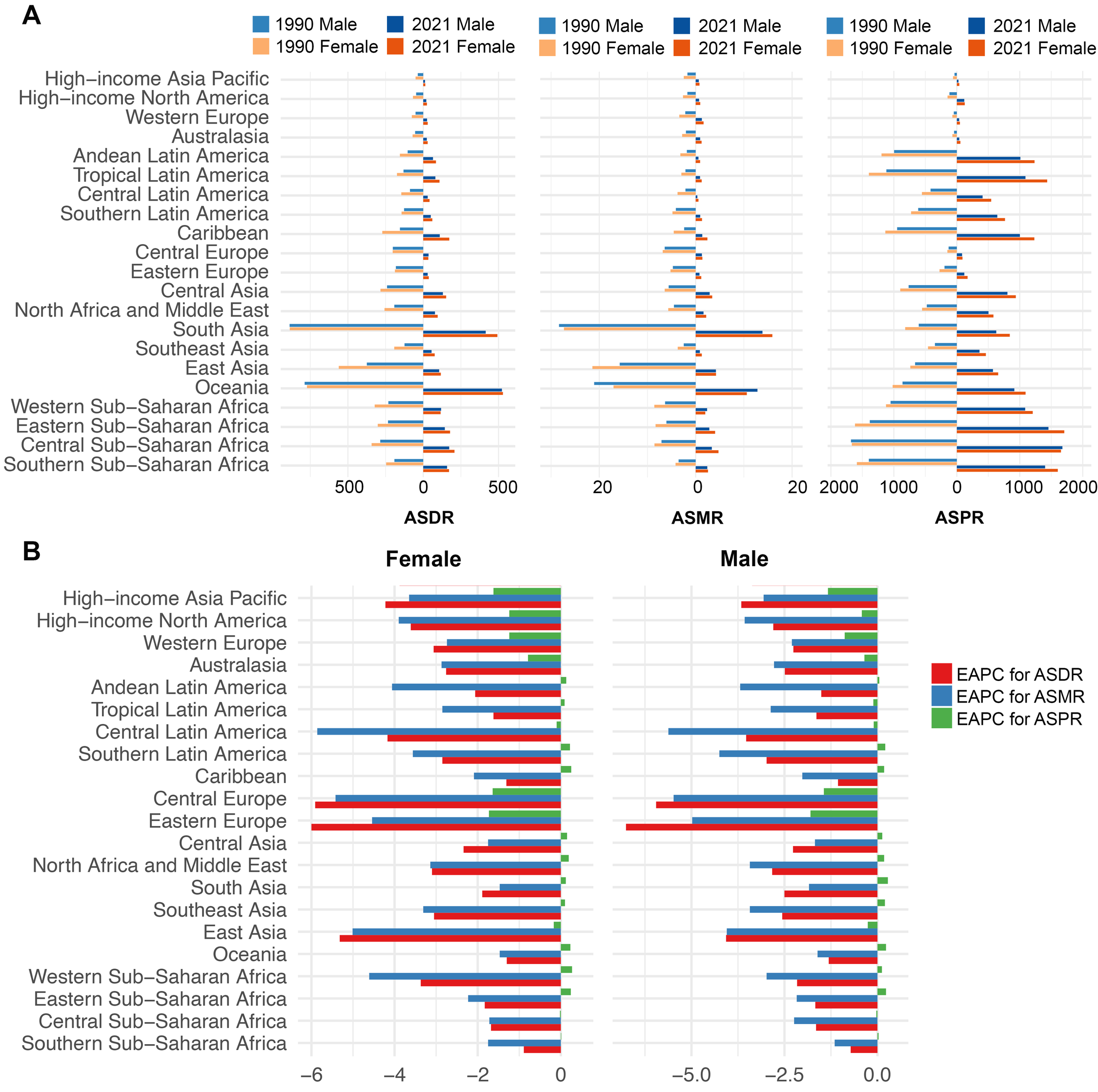

Supplement: S1 Fig — A: The ASDR, ASMR, ASPR of RHD in female and male in 1990 and 2021 in 21GBD regions. B: The EAPC of ASDR, ASMR, ASPR in female and male from 1990 to 2021 in 21 GBD regions. (TIF) [file pone.0334914.s001.tif]

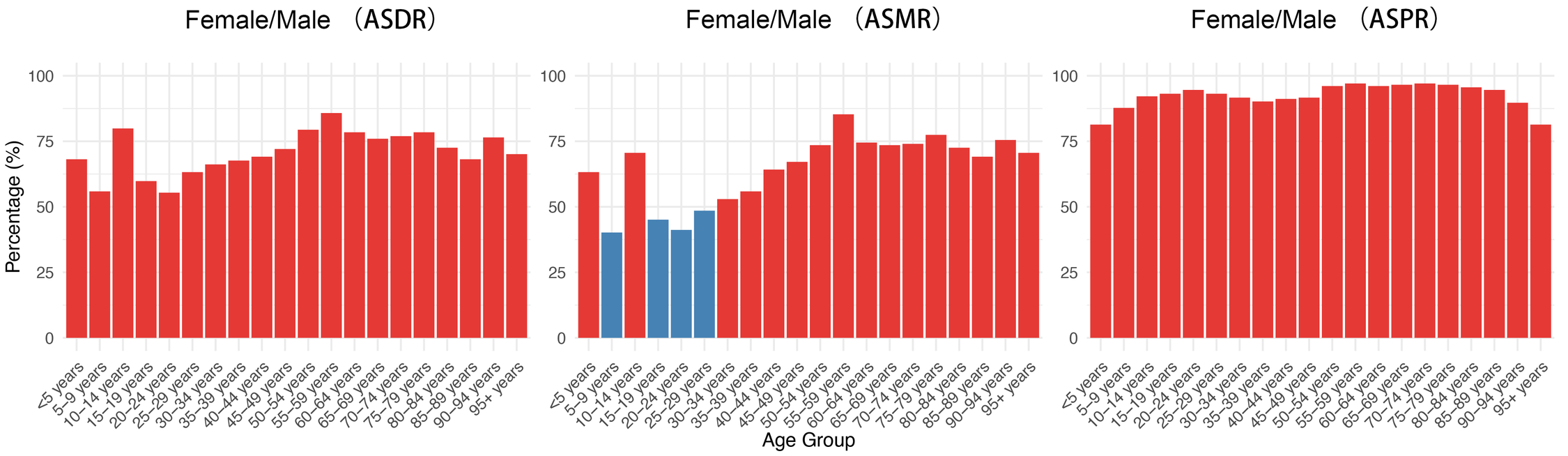

Supplement: S2 Fig — Female/ Male = the female by male ratio. (TIF) [file pone.0334914.s002.tif]
