# Supplementary material for: Sex difference in the burden of rheumatic heart disease: Insights from the Global Burden of Disease Study 2021
Source: PLoS One. 2025 Oct 22;20(10):e0334914. doi: 10.1371/journal.pone.0334914 (PMC12543145; doi:10.1371/journal.pone.0334914)
Supplement: S1 Table — (DOCX) [file pone.0334914.s003.docx]

**S1 Table :** The ASDR and the EAPC of ASDR in RHD by gender.

| **Region** | **Age-Standardized Rate Per 100 , 000 People (95% UI)** | | | | | | **Estimated Annual Percentage Change of DALYs Rate  from 1990 to 2021 (95% CI)** | | |
| --- | --- | --- | --- | --- | --- | --- | --- | --- | --- |
|  | **DALYs Rate in 1990** | | | **DALYs Rate in 2021** | | |  |  |  |
|  |  |  |  |  |  |  |  |  |  |
|  | **Male** | **Female** | **Female / Male** | **Male** | **Female** | **Female / Male** | **Male** | **Female** | **Female / Male** |
| Global | 321.09 （269.51 , 407.53) | 374.7 （290.09 , 461.87) | 1.17 | 150.11 （125.15 , 204.97) | 173.99 （148.21 , 211.13) | 1.16 | -2.5 （-2.56 , -2.45) | -2.54 （-2.61 , -2.46) | 1.02 |
| Low SDI | 567.92 （433.65 , 854.43) | 593.16 （375.6 , 866.3) | 1.04 | 285.36 （199.79 , 444.24) | 336.45 （259.51 , 493.36) | 1.18 | -2.21 （-2.38 , -2.04) | -1.78 （-1.89 , -1.67) | 0.81 |
| Low-middle SDI | 672.1 （498.83 , 1025.31) | 676.45 （451.67 , 975.3) | 1.01 | 317.45 （243.87 , 532.43) | 367.82 （303.08 , 519.08) | 1.16 | -2.45 （-2.58 , -2.31) | -1.91 （-1.98 , -1.84) | 0.78 |
| Middle SDI | 333.86 （280.6 , 380.58) | 466.9 （377.07 , 557.91) | 1.40 | 131.3 （108.68 , 158.07) | 148.41 （119.68 , 186.86) | 1.13 | -2.99 （-3.05 , -2.93) | -3.81 （-3.96 , -3.67) | 1.27 |
| High-middle SDI | 185.9 （163.54 , 209.75) | 239.82 （203.7 , 279.66) | 1.29 | 56.61 （46.44 , 68.17) | 65.76 （53.89 , 80.39) | 1.16 | -4.14 （-4.26 , -4.02) | -4.52 （-4.67 , -4.37) | 1.09 |
| High SDI | 62.88 （60.64 , 65.69) | 82.77 （78.04 , 87.2) | 1.32 | 22.65 （20.53 , 24.94) | 26.11 （23.28 , 28.82) | 1.15 | -3.37 （-3.73 , -3.02) | -3.88 （-4.2 , -3.56) | 1.15 |
| High-income Asia Pacific | 36.19 （34.69 , 37.58) | 50.78 （46.86 , 53.81) | 1.40 | 11.86 （10.88 , 12.65) | 13.69 （11.47 , 15.32) | 1.15 | -3.66 （-3.74 , -3.57) | -4.22 （-4.36 , -4.08) | 1.15 |
| High-income North America | 46.94 （44.44 , 49.66) | 68.08 （63.88 , 71.81) | 1.45 | 20.36 （17.51 , 22.9) | 24.6 （21.68 , 27.05) | 1.21 | -2.8 （-3.36 , -2.25) | -3.61 （-4.16 , -3.06) | 1.29 |
| Western Europe | 50.92 （49.4 , 52.61) | 75.78 （71.52 , 78.79) | 1.49 | 25.55 （23.56 , 27.27) | 29.81 （26.13 , 32.28) | 1.17 | -2.26 （-2.48 , -2.03) | -3.06 （-3.25 , -2.86) | 1.35 |
| Australasia | 53.7 （50.69 , 56.58) | 70.59 （65.58 , 75.68) | 1.31 | 23.9 （21.74 , 26) | 29.52 （26.43 , 32.37) | 1.24 | -2.49 （-2.77 , -2.19) | -2.76 （-2.91 , -2.6) | 1.11 |
| Andean Latin America | 103.32 （79.84 , 133.22) | 154.87 （125.35 , 201.29) | 1.50 | 64.87 （45.74 , 92.73) | 84.58 （61.25 , 118.86) | 1.30 | -1.51 （-1.62 , -1.4) | -2.06 （-2.18 , -1.93) | 1.36 |
| Tropical Latin America | 130.99 （110.29 , 160) | 174.25 （147.44 , 208.22) | 1.33 | 80.79 （59.97 , 108.14) | 108.06 （81.98 , 144.54) | 1.34 | -1.64 （-1.71 , -1.57) | -1.62 （-1.69 , -1.55) | 0.99 |
| Central Latin America | 87.99 （80.14 , 98.49) | 145.68 （135.09 , 159.44) | 1.66 | 29.76 （21.43 , 40.23) | 41.92 （31.82 , 56.16) | 1.41 | -3.53 （-3.73 , -3.33) | -4.17 （-4.38 , -3.97) | 1.18 |
| Southern Latin America | 127.28 （113.98 , 144.77) | 144.02 （128.72 , 163.63) | 1.13 | 50.22 （37.8 , 66.71) | 60.51 （46.34 , 78.67) | 1.20 | -2.98 （-3.07 , -2.88) | -2.85 （-2.93 , -2.76) | 0.96 |
| Caribbean | 154.61 （124.71 , 190.15) | 271.91 （201.59 , 344.97) | 1.76 | 109.81 （82.19 , 146.04) | 173 （127.6 , 233.02) | 1.58 | -1.06 （-1.18 , -0.94) | -1.31 （-1.43 , -1.2) | 1.24 |
| Central Europe | 201.9 （195.01 , 209.39) | 202.86 （194.89 , 211.83) | 1.00 | 35.35 （31.26 , 39.22) | 33.84 （29.41 , 38.08) | 0.96 | -5.95 （-6.46 , -5.45) | -5.91 （-6.37 , -5.46) | 0.99 |
| Eastern Europe | 181.74 （171.67 , 202.83) | 187.95 （179.2 , 202.57) | 1.03 | 29.57 （26.41 , 33.58) | 36.88 （32.74 , 42.03) | 1.25 | -6.76 （-7.3 , -6.23) | -6 （-6.3 , -5.69) | 0.89 |
| Central Asia | 240.37 （213.69 , 272.84) | 283.95 （256.21 , 322.19) | 1.18 | 130.62 （111.19 , 154.77) | 152.16 （128.33 , 180.97) | 1.16 | -2.27 （-2.45 , -2.08) | -2.34 （-2.51 , -2.18) | 1.03 |
| North Africa and Middle East | 191.99 （130.34 , 247.03) | 257.16 （158.21 , 364.13) | 1.34 | 78.03 （64.74 , 94.81) | 96.11 （78.18 , 119.09) | 1.23 | -2.83 （-2.86 , -2.79) | -3.1 （-3.21 , -2.99) | 1.10 |
| South Asia | 887.34 （667.65 , 1344.54) | 886.32 （605.71 , 1308.32) | 1.00 | 415.02 （321.71 , 694.06) | 492.53 （400.79 , 702.01) | 1.19 | -2.5 （-2.67 , -2.34) | -1.89 （-1.98 , -1.8) | 0.76 |
| Southeast Asia | 124.21 （89 , 154.17) | 192.47 （125.23 , 248.28) | 1.55 | 55.45 （42.11 , 74.09) | 76.31 （58.35 , 99) | 1.38 | -2.56 （-2.63 , -2.5) | -3.05 （-3.11 , -2.99) | 1.19 |
| East Asia | 374.43 （297.76 , 441.91) | 561.06 （435.76 , 700.33) | 1.50 | 105.97 （81.09 , 132.38) | 116.79 （88.54 , 153.55) | 1.10 | -4.07 （-4.19 , -3.96) | -5.32 （-5.47 , -5.17) | 1.31 |
| Oceania | 787.5 （496.43 , 1644.13) | 771.98 （384.65 , 1872.63) | 0.98 | 522.58 （281.96 , 1033.97) | 529.33 （297.26 , 1124.69) | 1.01 | -1.31 （-1.35 , -1.27) | -1.3 （-1.37 , -1.23) | 0.99 |
| Western Sub-Saharan Africa | 231.52 （174.9 , 305.31) | 322.21 （222.25 , 434.02) | 1.39 | 118.81 （89.4 , 155.05) | 115.45 （88.25 , 148.19) | 0.97 | -2.16 （-2.24 , -2.09) | -3.37 （-3.63 , -3.11) | 1.56 |
| Eastern Sub-Saharan Africa | 233.79 （181.28 , 295.62) | 301.96 （231.72 , 376.78) | 1.29 | 143.46 （109.34 , 188.46) | 178.26 （135.84 , 230.06) | 1.24 | -1.67 （-1.71 , -1.63) | -1.83 （-1.91 , -1.76) | 1.10 |
| Central Sub-Saharan Africa | 285.59 （204.94 , 409.58) | 342.82 （204.6 , 508.66) | 1.20 | 172.51 （115.25 , 324.55) | 207.51 （140.46 , 312.44) | 1.20 | -1.65 （-1.7 , -1.6) | -1.68 （-1.75 , -1.61) | 1.02 |
| Southern Sub-Saharan Africa | 191.41 （153.72 , 240.54) | 246.28 （203.96 , 303.29) | 1.29 | 158.21 （123 , 200.39) | 171.32 （130.99 , 219.47) | 1.08 | -0.72 （-0.94 , -0.5) | -0.89 （-1.23 , -0.55) | 1.24 |
| Abbreviations: RHD = Rheumatic heart disease, ASDR = age-standardized DALYs rates, EAPC  =  Estimated Annual Percentage Change, UI = Uncertainty Intervals, CI = Confidence Intervals. | | | | | | | | | |
