# Supplementary material for: Sex difference in the burden of rheumatic heart disease: Insights from the Global Burden of Disease Study 2021
Source: PLoS One. 2025 Oct 22;20(10):e0334914. doi: 10.1371/journal.pone.0334914 (PMC12543145; doi:10.1371/journal.pone.0334914)
Supplement: S2 Table — (DOCX) [file pone.0334914.s004.docx]

**S2 Table :** The ASMR and the EAPC of ASMR in RHD by gender.

| **Region** | **Age-Standardized Rate Per 100 , 000 People (95% UI)** | | | | | | **Estimated Annual Percentage Change of Deaths Rate  from 1990 to 2021 (95% CI)** | | |
| --- | --- | --- | --- | --- | --- | --- | --- | --- | --- |
|  | **Deaths Rate in1990** | | | **Deaths Rate in 2021** | | |  |  |  |
|  |  |  |  |  |  |  |  |  |  |
|  | **Male** | **Female** | **Female / Male** | **Male** | **Female** | **Female / Male** | **Male** | **Female** | **Female / Male** |
| Global | 9.71 （8.06 , 12.33) | 10.77 （8.33 , 13.01) | 1.11 | 4.22 （3.54 , 6.19) | 4.72 （4.01 , 5.82) | 1.12 | -2.44 （-2.53 , -2.36) | -2.62 （-2.67 , -2.57) | 1.07 |
| Low SDI | 17.67 （13.1 , 27.12) | 17.09 （10.07 , 23.92) | 0.97 | 8.82 （5.67 , 15.58) | 10.24 （7.65 , 15.95) | 1.16 | -1.63 （-1.81 , -1.45) | -1.34 （-1.46 , -1.22) | 0.82 |
| Low-middle SDI | 20.62 （14.46 , 32.18) | 19.4 （12.45 , 27.74) | 0.94 | 10.14 （7.37 , 19.22) | 11.22 （9.14 , 16.96) | 1.11 | -1.9 （-2.04 , -1.75) | -1.53 （-1.6 , -1.46) | 0.81 |
| Middle SDI | 11.26 （9.28 , 13.14) | 15.59 （12.38 , 19.05) | 1.38 | 4.06 （3.25 , 4.97) | 4.39 （3.28 , 5.97) | 1.08 | -3.14 （-3.2 , -3.08) | -4.06 （-4.15 , -3.96) | 1.29 |
| High-middle SDI | 6.21 （5.36 , 7.12) | 7.72 （6.53 , 9.14) | 1.24 | 1.97 （1.6 , 2.38) | 2.23 （1.79 , 2.82) | 1.13 | -3.6 （-3.73 , -3.46) | -3.9 （-4.07 , -3.73) | 1.08 |
| High SDI | 2.38 （2.28 , 2.47) | 3.32 （3.06 , 3.5) | 1.39 | 0.96 （0.85 , 1.02) | 1.19 （0.98 , 1.32) | 1.24 | -3.4 （-3.6 , -3.21) | -3.66 （-3.82 , -3.49) | 1.08 |
| High-income Asia Pacific | 1.71 （1.6 , 1.77) | 2.47 （2.16 , 2.63) | 1.44 | 0.65 （0.57 , 0.69) | 0.79 （0.59 , 0.92) | 1.22 | -3.06 （-3.13 , -2.99) | -3.65 （-3.71 , -3.59) | 1.19 |
| High-income North America | 1.72 （1.64 , 1.78) | 2.63 （2.39 , 2.76) | 1.53 | 0.71 （0.6 , 0.78) | 0.95 （0.79 , 1.04) | 1.34 | -3.57 （-3.94 , -3.2) | -3.9 （-4.22 , -3.57) | 1.09 |
| Western Europe | 2.16 （2.06 , 2.24) | 3.4 （3.12 , 3.57) | 1.57 | 1.28 （1.16 , 1.37) | 1.64 （1.35 , 1.81) | 1.28 | -2.3 （-2.52 , -2.09) | -2.74 （-2.88 , -2.59) | 1.19 |
| Australasia | 2.02 （1.9 , 2.13) | 2.81 （2.55 , 3.01) | 1.39 | 0.93 （0.83 , 1.01) | 1.23 （1.04 , 1.37) | 1.32 | -2.78 （-3.01 , -2.55) | -2.87 （-3.02 , -2.71) | 1.03 |
| Andean Latin America | 1.83 （1.52 , 2.32) | 3.18 （2.69 , 4.41) | 1.74 | 0.58 （0.44 , 0.74) | 0.92 （0.71 , 1.17) | 1.59 | -3.69 （-3.8 , -3.59) | -4.06 （-4.14 , -3.99) | 1.10 |
| Tropical Latin America | 2.12 （2.03 , 2.23) | 2.97 （2.81 , 3.12) | 1.40 | 0.91 （0.79 , 0.98) | 1.25 （1.06 , 1.35) | 1.37 | -2.87 （-2.96 , -2.77) | -2.85 （-2.95 , -2.75) | 0.99 |
| Central Latin America | 2.12 （2.05 , 2.18) | 3.76 （3.63 , 3.88) | 1.77 | 0.35 （0.25 , 0.42) | 0.58 （0.41 , 0.68) | 1.66 | -5.62 （-5.78 , -5.46) | -5.86 （-6.02 , -5.7) | 1.04 |
| Southern Latin America | 4.11 （3.9 , 4.33) | 4.82 （4.4 , 5.17) | 1.17 | 0.94 （0.84 , 1.03) | 1.31 （1.13 , 1.47) | 1.39 | -4.25 （-4.47 , -4.02) | -3.56 （-3.82 , -3.3) | 0.84 |
| Caribbean | 2.39 （1.9 , 3.09) | 4.55 （3.31 , 6.23) | 1.90 | 1.37 （1.04 , 1.88) | 2.43 （1.75 , 3.54) | 1.77 | -2.02 （-2.15 , -1.9) | -2.09 （-2.19 , -1.98) | 1.03 |
| Central Europe | 6.43 （6.23 , 6.66) | 6.84 （6.56 , 7.15) | 1.06 | 1.29 （1.14 , 1.42) | 1.39 （1.2 , 1.55) | 1.08 | -5.48 （-5.78 , -5.18) | -5.42 （-5.7 , -5.15) | 0.99 |
| Eastern Europe | 4.73 （4.47 , 5.38) | 5.23 （4.99 , 5.65) | 1.11 | 0.79 （0.71 , 0.89) | 1.16 （1.03 , 1.3) | 1.47 | -4.98 （-5.54 , -4.42) | -4.54 （-4.9 , -4.17) | 0.91 |
| Central Asia | 5.61 （4.94 , 6.67) | 6.43 （5.82 , 7.67) | 1.15 | 2.9 （2.54 , 3.3) | 3.43 （2.96 , 3.93) | 1.18 | -1.68 （-1.88 , -1.48) | -1.75 （-1.93 , -1.58) | 1.04 |
| North Africa and Middle East | 4.51 （2.91 , 6.33) | 5.69 （3.33 , 9.6) | 1.26 | 1.63 （1.34 , 2) | 2.18 （1.8 , 2.66) | 1.34 | -3.43 （-3.5 , -3.35) | -3.14 （-3.24 , -3.05) | 0.92 |
| South Asia | 28.35 （20.35 , 43.81) | 27.33 （18.01 , 39.17) | 0.96 | 13.86 （10.32 , 25.28) | 15.91 （12.84 , 23.65) | 1.15 | -1.84 （-2 , -1.68) | -1.47 （-1.56 , -1.37) | 0.80 |
| Southeast Asia | 2.48 （1.65 , 3.24) | 3.72 （2.21 , 5.33) | 1.50 | 0.87 （0.61 , 1.27) | 1.27 （0.9 , 1.77) | 1.46 | -3.43 （-3.47 , -3.4) | -3.31 （-3.4 , -3.22) | 0.97 |
| East Asia | 15.76 （12.51 , 19.1) | 21.43 （16.64 , 27.21) | 1.36 | 4.2 （3.12 , 5.28) | 4.22 （3.02 , 5.94) | 1.00 | -4.05 （-4.14 , -3.96) | -5.01 （-5.18 , -4.83) | 1.24 |
| Oceania | 21.06 （13.25 , 47.46) | 17.05 （7.89 , 45.27) | 0.81 | 12.82 （6.24 , 28.5) | 10.61 （5.29 , 25.51) | 0.83 | -1.61 （-1.63 , -1.58) | -1.47 （-1.53 , -1.4) | 0.91 |
| Western Sub-Saharan Africa | 6.35 （4.48 , 8.71) | 8.58 （5.52 , 12.56) | 1.35 | 2.37 （1.84 , 3.22) | 1.99 （1.63 , 2.46) | 0.84 | -2.98 （-3.06 , -2.89) | -4.61 （-4.81 , -4.4) | 1.55 |
| Eastern Sub-Saharan Africa | 6.07 （4.52 , 7.44) | 8.32 （6.31 , 10.64) | 1.37 | 2.83 （2.18 , 3.83) | 4.03 （2.72 , 5.02) | 1.42 | -2.17 （-2.3 , -2.04) | -2.23 （-2.33 , -2.13) | 1.03 |
| Central Sub-Saharan Africa | 7.09 （4.61 , 11.52) | 8.55 （4.12 , 14.7) | 1.21 | 3.35 （1.96 , 9.24) | 4.73 （2.57 , 8.42) | 1.41 | -2.24 （-2.32 , -2.15) | -1.72 （-1.82 , -1.63) | 0.77 |
| Southern Sub-Saharan Africa | 3.54 （2.91 , 4.75) | 4.18 （3.55 , 5.46) | 1.18 | 2.41 （1.87 , 2.94) | 2.56 （1.89 , 3.21) | 1.06 | -1.15 （-1.38 , -0.91) | -1.75 （-2.04 , -1.46) | 1.52 |
| Abbreviations: RHD = Rheumatic heart disease, ASMR = age-standardized mortality rates, EAPC  =  Estimated Annual Percentage Change, UI = Uncertainty Intervals, CI = Confidence Intervals. | | | | | | | | | |
