# Supplementary material for: Sex difference in the burden of rheumatic heart disease: Insights from the Global Burden of Disease Study 2021
Source: PLoS One. 2025 Oct 22;20(10):e0334914. doi: 10.1371/journal.pone.0334914 (PMC12543145; doi:10.1371/journal.pone.0334914)
Supplement: S3 Table — (DOCX) [file pone.0334914.s005.docx]

**S3 Table :** The ASPR and the EAPC of ASPR in RHD by gender.

| **Region** | **Age-Standardized Rate Per 100 , 000 People (95% UI)** | | | | | | **Estimated Annual Percentage Change of Prevalence Rate  from 1990 to 2021 (95% CI)** | | |
| --- | --- | --- | --- | --- | --- | --- | --- | --- | --- |
|  |  |  |  |  |  |  |  |  |  |
|  | **Prevalence Rate in 1990** | | | **Prevalence Rate in 2021** | | |  |  |  |
|  | **Male** | **Female** | **Female / Male** | **Male** | **Female** | **Female / Male** | **Male** | **Female** | **Female / Male** |
| Global | 548 （436.06 , 673.93) | 666.29 （538.76 , 813.74) | 1.22 | 614.2 （482.58 , 761.31) | 754.77 （600.82 , 936.66) | 1.23 | 0.47 （0.42 , 0.51) | 0.47 （0.42 , 0.52) | 1.00 |
| Low SDI | 1034.59 （818.23 , 1287.41) | 1198.51 （946.5 , 1483.79) | 1.16 | 1099.06 （862.63 , 1369.56) | 1267.26 （999.84 , 1584.18) | 1.15 | 0.29 （0.25 , 0.32) | 0.26 （0.23 , 0.29) | 0.90 |
| Low-middle SDI | 693.38 （545.68 , 864.37) | 899.96 （718.66 , 1111.43) | 1.30 | 749.86 （582.45 , 935.21) | 962.38 （763.88 , 1197.12) | 1.28 | 0.29 （0.26 , 0.32) | 0.21 （0.2 , 0.22) | 0.72 |
| Middle SDI | 697.78 （547.37 , 867.03) | 803.09 （631.16 , 991.36) | 1.15 | 655.41 （510.49 , 810.15) | 783.04 （618.55 , 966.97) | 1.19 | -0.07 （-0.14 , 0) | 0.03 （-0.04 , 0.1) | -0.43 |
| High-middle SDI | 335.04 （279.3 , 400) | 418.95 （358.41 , 490.21) | 1.25 | 287.86 （236.49 , 346.57) | 358.84 （302.59 , 425.23) | 1.25 | -0.31 （-0.4 , -0.23) | -0.35 （-0.43 , -0.27) | 1.13 |
| High SDI | 85.73 （76.11 , 97.17) | 113.94 （100.9 , 129.17) | 1.33 | 83.25 （75.76 , 91.99) | 94.49 （86.4 , 103.43) | 1.14 | -0.12 （-0.22 , -0.02) | -0.88 （-1.03 , -0.73) | 7.33 |
| High-income Asia Pacific | 36.92 （31.9 , 42.73) | 60.68 （51.48 , 70.4) | 1.64 | 26.21 （22.91 , 29.71) | 39.66 （33.98 , 45.54) | 1.51 | -1.33 （-1.41 , -1.24) | -1.62 （-1.71 , -1.53) | 1.22 |
| High-income North America | 117.32 （102 , 136.29) | 147.43 （126.4 , 171.8) | 1.26 | 118.34 （108.11 , 129.84) | 126.13 （115.62 , 138.46) | 1.07 | -0.42 （-0.68 , -0.16) | -1.24 （-1.65 , -0.83) | 2.95 |
| Western Europe | 48.23 （42.07 , 55.86) | 71.17 （63.28 , 81.66) | 1.48 | 37.63 （32.6 , 43.47) | 49.2 （42.96 , 57.2) | 1.31 | -0.88 （-0.92 , -0.84) | -1.24 （-1.29 , -1.2) | 1.41 |
| Australasia | 44.01 （37.5 , 51.74) | 70.28 （60.92 , 82.71) | 1.60 | 39.18 （33.41 , 45.62) | 56.69 （47.8 , 65.98) | 1.45 | -0.35 （-0.51 , -0.19) | -0.79 （-0.95 , -0.63) | 2.26 |
| Andean Latin America | 995.86 （772.96 , 1250.89) | 1196.46 （944.16 , 1479.23) | 1.20 | 1009.97 （783.33 , 1264.76) | 1234.44 （975.34 , 1546.37) | 1.22 | 0.05 （0.03 , 0.07) | 0.13 （0.11 , 0.15) | 2.60 |
| Tropical Latin America | 1119.35 （868.93 , 1400.78) | 1397.28 （1111.67 , 1731.91) | 1.25 | 1090.33 （851.73 , 1360.41) | 1435.23 （1144.43 , 1778.85) | 1.32 | -0.11 （-0.13 , -0.09) | 0.09 （0.07 , 0.1) | -0.82 |
| Central Latin America | 417.37 （335.01 , 514.07) | 554.69 （449.59 , 678.09) | 1.33 | 409.34 （324.37 , 505.05) | 545.81 （441.77 , 666.66) | 1.33 | -0.1 （-0.16 , -0.04) | -0.1 （-0.16 , -0.04) | 1.00 |
| Southern Latin America | 613.81 （479.66 , 765.44) | 726.87 （587.25 , 888.81) | 1.18 | 642.94 （507.58 , 795.86) | 765.08 （609.03 , 951.61) | 1.19 | 0.21 （0.18 , 0.25) | 0.22 （0.19 , 0.25) | 1.05 |
| Caribbean | 948.08 （749.78 , 1177) | 1136.22 （912.48 , 1413.11) | 1.20 | 1001.59 （780.94 , 1247.77) | 1231.38 （979.79 , 1517.65) | 1.23 | 0.18 （0.17 , 0.19) | 0.25 （0.24 , 0.26) | 1.39 |
| Central Europe | 130.67 （117.13 , 146.25) | 150.42 （134.59 , 167.92) | 1.15 | 83.3 （73.33 , 94.2) | 90 （80.02 , 101.27) | 1.08 | -1.44 （-1.74 , -1.14) | -1.64 （-1.98 , -1.29) | 1.14 |
| Eastern Europe | 195.98 （171.92 , 222.86) | 275.18 （243.17 , 310.09) | 1.40 | 119.01 （103.68 , 136.28) | 170.93 （149.5 , 195.41) | 1.44 | -1.8 （-1.87 , -1.72) | -1.73 （-1.81 , -1.64) | 0.96 |
| Central Asia | 766.11 （603.82 , 939.87) | 898.88 （724.09 , 1102.13) | 1.17 | 803.66 （630.45 , 1005.11) | 936.85 （736.62 , 1155.02) | 1.17 | 0.13 （0.11 , 0.15) | 0.15 （0.13 , 0.17) | 1.15 |
| North Africa and Middle East | 477.48 （376.51 , 590.69) | 552.01 （441.83 , 676.48) | 1.16 | 503.4 （396.22 , 618.46) | 581.51 （461.54 , 707.85) | 1.16 | 0.18 （0.14 , 0.22) | 0.19 （0.16 , 0.23) | 1.06 |
| South Asia | 604.85 （475.02 , 755.36) | 820.47 （649.35 , 1024.12) | 1.36 | 626.46 （482.7 , 783.53) | 840.97 （660.95 , 1058.72) | 1.34 | 0.28 （0.21 , 0.35) | 0.12 （0.1 , 0.15) | 0.43 |
| Southeast Asia | 347.93 （281.19 , 423.45) | 457.76 （369.61 , 554.68) | 1.32 | 360.02 （290.02 , 439.15) | 461.27 （368.2 , 563.8) | 1.28 | 0.2 （0.17 , 0.23) | 0.1 （0.07 , 0.14) | 0.50 |
| East Asia | 663.35 （516.5 , 823.13) | 739.1 （590.04 , 902.65) | 1.11 | 574.08 （447.64 , 708.63) | 656.78 （528.4 , 806.59) | 1.14 | -0.26 （-0.4 , -0.13) | -0.17 （-0.32 , -0.03) | 0.65 |
| Oceania | 862.71 （672.9 , 1058.65) | 1019.85 （805.08 , 1242.39) | 1.18 | 913.63 （714.07 , 1115.13) | 1091.98 （861.54 , 1340.77) | 1.20 | 0.23 （0.19 , 0.28) | 0.23 （0.19 , 0.28) | 1.00 |
| Western Sub-Saharan Africa | 1052.52 （822.02 , 1310.82) | 1124.96 （876.29 , 1390.38) | 1.07 | 1085.94 （848.18 , 1346.97) | 1205.5 （938.29 , 1500.08) | 1.11 | 0.12 （0.09 , 0.14) | 0.27 （0.25 , 0.3) | 2.25 |
| Eastern Sub-Saharan Africa | 1382.41 （1092.75 , 1726.62) | 1617.92 （1280.78 , 2004.41) | 1.17 | 1455.31 （1141.99 , 1799.94) | 1707.03 （1342.58 , 2143.18) | 1.17 | 0.23 （0.22 , 0.25) | 0.24 （0.22 , 0.26) | 1.04 |
| Central Sub-Saharan Africa | 1681.76 （1299.97 , 2087.33) | 1668.17 （1307.7 , 2067.65) | 0.99 | 1677.11 （1297.49 , 2097.18) | 1654.22 （1295.75 , 2082.23) | 0.99 | -0.03 （-0.04 , -0.01) | -0.02 （-0.04 , 0) | 0.67 |
| Southern Sub-Saharan Africa | 1397.69 （1097.88 , 1745.21) | 1587.94 （1252.83 , 1990.96) | 1.14 | 1402.43 （1100.19 , 1744.67) | 1603.83 （1266.62 , 1992.89) | 1.14 | 0.03 （0.01 , 0.05) | 0.01 （-0.01 , 0.02) | 0.33 |
| Abbreviations: RHD = Rheumatic heart disease, ASPR = age-standardized prevalence rates, EAPC  =  Estimated Annual Percentage Change, UI = Uncertainty Intervals, CI = Confidence Intervals. | | | | | | | | | |
