# Supplementary material for: Sex difference in the burden of rheumatic heart disease: Insights from the Global Burden of Disease Study 2021
Source: PLoS One. 2025 Oct 22;20(10):e0334914. doi: 10.1371/journal.pone.0334914 (PMC12543145; doi:10.1371/journal.pone.0334914)
Supplement: S4 Table — (DOCX) [file pone.0334914.s006.docx]

**S4 Table :** Global Extreme Values of ASDR, ASMR, and ASPR by Sex in 2021 across 204 countries.

| **Indicator** | **Sex** | **Country** | **Value (95% UI)** |
| --- | --- | --- | --- |
| The highest ASDR | Female | Pakistan | 679.05 (465.19, 1046.38) |
| The lowest ASDR | Female | Colombia | 8.75 (7.05, 10.49) |
| The highest ASDR | Male | Vanuatu | 584.02 (340.29, 837.57) |
| The lowest ASDR | Male | Andorra | 6.36 (4.61, 8.59) |
| The highest ASMR | Female | Pakistan | 21.09 (14.51, 32.16) |
| The lowest ASMR | Female | Guatemala | 0.19 (0.15, 0.23) |
| The highest ASMR | Male | Vanuatu | 14.31 (7.53, 21.31) |
| The lowest ASMR | Male | Guatemala | 0.13 (0.10, 0.16) |
| The highest ASPR | Female | Eritrea | 2000.21 (1582.66, 2509.08) |
| The lowest ASPR | Female | Finland | 19.82 (15.78, 23.85) |
| The highest ASPR | Male | Eritrea | 1732.00 (1334.83, 2155.26) |
| The lowest ASPR | Male | Sweden | 14.62 (12.39, 17.38) |
| Abbreviations: ASDR: Age-standardized disability-adjusted life-years (DALYs) rate per 100,000. ASMR: Age-standardized mortality rate per 100,000. ASPR: Age-standardized prevalence rate per 100,000. 95% UI: 95% uncertainty interval. | | | |
