# Supplementary material for: Sex difference in the burden of rheumatic heart disease: Insights from the Global Burden of Disease Study 2021
Source: PLoS One. 2025 Oct 22;20(10):e0334914. doi: 10.1371/journal.pone.0334914 (PMC12543145; doi:10.1371/journal.pone.0334914)
Supplement: S5 Table — (DOCX) [file pone.0334914.s007.docx]

**S5 Table:** The ASDR, ASMR, ASPR in female and male and the gender difference of RHD across 204 countries

| **Country** | **year** | **ASDR** | | | **ASMR** | | | **ASPR** | | | **SDI** |
| --- | --- | --- | --- | --- | --- | --- | --- | --- | --- | --- | --- |
|  |  | **Female** | **Male** | **Female/Male** | **Female** | **Male** | **Female/Male** | **Female** | **Male** | **Female/Male** |  |
| Afghanistan | 2021 | 189.44 | 159.52 | 1.19 | 4.39 | 3.94 | 1.12 | 732.03 | 664.62 | 1.10 | 0.34 |
| Albania | 2021 | 91.75 | 77.89 | 1.18 | 1.53 | 1.19 | 1.29 | 1164.50 | 990.55 | 1.18 | 0.71 |
| Algeria | 2021 | 76.79 | 55.08 | 1.39 | 1.89 | 1.06 | 1.79 | 603.79 | 545.61 | 1.11 | 0.66 |
| American Samoa | 2021 | 290.52 | 202.15 | 1.44 | 6.31 | 4.21 | 1.50 | 1238.53 | 1013.02 | 1.22 | 0.72 |
| Andorra | 2021 | 19.05 | 6.36 | 3.00 | 0.85 | 0.26 | 3.28 | 47.43 | 24.06 | 1.97 | 0.87 |
| Angola | 2021 | 210.24 | 178.79 | 1.18 | 4.76 | 3.62 | 1.31 | 1632.46 | 1653.69 | 0.99 | 0.45 |
| Antigua and Barbuda | 2021 | 97.72 | 65.28 | 1.50 | 0.95 | 0.39 | 2.43 | 1341.73 | 1077.75 | 1.24 | 0.75 |
| Argentina | 2021 | 80.49 | 67.37 | 1.19 | 1.62 | 1.15 | 1.40 | 1095.35 | 930.00 | 1.18 | 0.72 |
| Armenia | 2021 | 118.55 | 86.06 | 1.38 | 2.36 | 1.21 | 1.96 | 1210.48 | 1012.64 | 1.20 | 0.70 |
| Australia | 2021 | 25.93 | 20.64 | 1.26 | 1.11 | 0.83 | 1.34 | 55.44 | 37.26 | 1.49 | 0.84 |
| Austria | 2021 | 32.92 | 28.71 | 1.15 | 1.86 | 1.50 | 1.24 | 64.54 | 50.16 | 1.29 | 0.85 |
| Azerbaijan | 2021 | 98.88 | 88.13 | 1.12 | 1.45 | 1.29 | 1.12 | 1116.77 | 942.03 | 1.19 | 0.69 |
| Bahrain | 2021 | 29.44 | 14.02 | 2.10 | 1.05 | 0.48 | 2.17 | 76.86 | 47.69 | 1.61 | 0.75 |
| Bangladesh | 2021 | 481.20 | 434.20 | 1.11 | 16.68 | 15.95 | 1.05 | 890.48 | 610.94 | 1.46 | 0.49 |
| Barbados | 2021 | 92.19 | 65.97 | 1.40 | 0.78 | 0.38 | 2.06 | 1332.40 | 1082.40 | 1.23 | 0.75 |
| Belarus | 2021 | 45.98 | 40.06 | 1.15 | 1.34 | 1.04 | 1.28 | 175.35 | 128.63 | 1.36 | 0.78 |
| Belgium | 2021 | 31.80 | 21.48 | 1.48 | 1.74 | 1.08 | 1.62 | 53.96 | 36.77 | 1.47 | 0.85 |
| Belize | 2021 | 91.47 | 74.63 | 1.23 | 0.76 | 0.62 | 1.23 | 1300.17 | 1023.08 | 1.27 | 0.61 |
| Benin | 2021 | 99.22 | 120.68 | 0.82 | 1.59 | 2.58 | 0.61 | 1148.40 | 1039.60 | 1.10 | 0.37 |
| Bermuda | 2021 | 13.75 | 18.96 | 0.73 | 0.31 | 0.48 | 0.65 | 51.48 | 40.14 | 1.28 | 0.82 |
| Bhutan | 2021 | 420.42 | 384.51 | 1.09 | 15.04 | 13.50 | 1.11 | 825.38 | 650.28 | 1.27 | 0.47 |
| Bolivia | 2021 | 117.49 | 78.89 | 1.49 | 2.21 | 1.11 | 1.98 | 1231.78 | 1017.67 | 1.21 | 0.60 |
| Bosnia and Herzegovina | 2021 | 21.51 | 14.98 | 1.44 | 0.83 | 0.51 | 1.65 | 46.12 | 42.49 | 1.09 | 0.72 |
| Botswana | 2021 | 119.96 | 121.56 | 0.99 | 1.45 | 1.80 | 0.81 | 1519.32 | 1330.52 | 1.14 | 0.64 |
| Brazil | 2021 | 108.41 | 80.93 | 1.34 | 1.25 | 0.91 | 1.37 | 1438.59 | 1090.88 | 1.32 | 0.65 |
| Brunei | 2021 | 52.26 | 36.11 | 1.45 | 2.45 | 1.76 | 1.39 | 90.28 | 49.50 | 1.82 | 0.81 |
| Bulgaria | 2021 | 62.13 | 59.00 | 1.05 | 2.08 | 1.90 | 1.10 | 58.85 | 56.40 | 1.04 | 0.77 |
| Burkina Faso | 2021 | 138.29 | 181.74 | 0.76 | 3.14 | 5.24 | 0.60 | 1092.14 | 996.03 | 1.10 | 0.29 |
| Burundi | 2021 | 195.01 | 158.36 | 1.23 | 4.58 | 3.39 | 1.35 | 1711.96 | 1478.56 | 1.16 | 0.29 |
| Cambodia | 2021 | 110.42 | 78.28 | 1.41 | 1.83 | 1.25 | 1.46 | 741.40 | 620.28 | 1.20 | 0.47 |
| Cameroon | 2021 | 112.86 | 114.71 | 0.98 | 2.11 | 2.35 | 0.90 | 1137.92 | 1045.29 | 1.09 | 0.48 |
| Canada | 2021 | 26.53 | 20.60 | 1.29 | 1.13 | 0.79 | 1.44 | 118.26 | 109.56 | 1.08 | 0.87 |
| Cape Verde | 2021 | 81.21 | 98.42 | 0.83 | 0.92 | 1.76 | 0.53 | 1225.48 | 1093.14 | 1.12 | 0.53 |
| Central African Republic | 2021 | 288.90 | 266.91 | 1.08 | 7.25 | 6.39 | 1.14 | 1663.12 | 1673.94 | 0.99 | 0.31 |
| Chad | 2021 | 158.02 | 176.40 | 0.90 | 3.49 | 4.47 | 0.78 | 1099.79 | 1005.77 | 1.09 | 0.24 |
| Chile | 2021 | 19.94 | 16.12 | 1.24 | 0.80 | 0.59 | 1.34 | 66.19 | 48.33 | 1.37 | 0.77 |
| China | 2021 | 117.34 | 107.37 | 1.09 | 4.26 | 4.31 | 0.99 | 662.73 | 578.73 | 1.15 | 0.72 |
| Colombia | 2021 | 8.75 | 7.78 | 1.13 | 0.21 | 0.20 | 1.04 | 54.63 | 34.91 | 1.56 | 0.66 |
| Comoros | 2021 | 169.64 | 124.46 | 1.36 | 3.63 | 2.14 | 1.70 | 1722.70 | 1497.75 | 1.15 | 0.48 |
| Republic of Congo | 2021 | 206.82 | 153.45 | 1.35 | 4.68 | 2.62 | 1.78 | 1660.96 | 1694.46 | 0.98 | 0.58 |
| Cook Islands | 2021 | 43.23 | 95.30 | 0.45 | 0.97 | 2.44 | 0.40 | 118.76 | 129.13 | 0.92 | 0.78 |
| Costa Rica | 2021 | 86.07 | 70.36 | 1.22 | 0.61 | 0.56 | 1.09 | 1443.48 | 1123.86 | 1.28 | 0.70 |
| Ivory Coast | 2021 | 111.76 | 121.03 | 0.92 | 2.00 | 2.52 | 0.79 | 1157.75 | 1050.89 | 1.10 | 0.43 |
| Croatia | 2021 | 28.76 | 25.94 | 1.11 | 1.34 | 1.10 | 1.22 | 51.81 | 36.50 | 1.42 | 0.80 |
| Cuba | 2021 | 106.24 | 83.67 | 1.27 | 1.24 | 0.88 | 1.40 | 1312.56 | 1067.97 | 1.23 | 0.67 |
| Cyprus | 2021 | 51.03 | 33.79 | 1.51 | 3.10 | 1.85 | 1.68 | 42.18 | 26.49 | 1.59 | 0.84 |
| Czech Republic | 2021 | 29.34 | 28.51 | 1.03 | 1.37 | 1.18 | 1.17 | 65.42 | 58.69 | 1.11 | 0.83 |
| Denmark | 2021 | 20.72 | 20.91 | 0.99 | 1.10 | 1.10 | 1.00 | 24.88 | 17.92 | 1.39 | 0.90 |
| Djibouti | 2021 | 162.22 | 125.35 | 1.29 | 3.44 | 2.17 | 1.59 | 1724.90 | 1476.81 | 1.17 | 0.49 |
| Dominica | 2021 | 125.99 | 89.16 | 1.41 | 1.66 | 1.00 | 1.66 | 1316.94 | 1069.18 | 1.23 | 0.75 |
| Dominican Republic | 2021 | 114.35 | 82.58 | 1.38 | 1.25 | 0.73 | 1.72 | 1267.28 | 1015.59 | 1.25 | 0.62 |
| Democratic Republic of the Congo | 2021 | 204.09 | 168.22 | 1.21 | 4.66 | 3.22 | 1.45 | 1662.60 | 1685.52 | 0.99 | 0.38 |
| Ecuador | 2021 | 77.42 | 61.76 | 1.25 | 0.72 | 0.56 | 1.29 | 1222.72 | 982.74 | 1.24 | 0.66 |
| Egypt | 2021 | 197.31 | 158.50 | 1.24 | 6.16 | 3.56 | 1.73 | 862.18 | 787.74 | 1.09 | 0.61 |
| El Salvador | 2021 | 73.69 | 54.30 | 1.36 | 0.37 | 0.21 | 1.78 | 1308.65 | 992.99 | 1.32 | 0.56 |
| Equatorial Guinea | 2021 | 135.54 | 121.02 | 1.12 | 2.21 | 1.57 | 1.41 | 1629.43 | 1643.21 | 0.99 | 0.66 |
| Eritrea | 2021 | 241.10 | 178.78 | 1.35 | 6.02 | 3.70 | 1.63 | 2000.21 | 1732.00 | 1.15 | 0.40 |
| Estonia | 2021 | 27.93 | 18.32 | 1.52 | 0.75 | 0.43 | 1.74 | 176.79 | 118.93 | 1.49 | 0.84 |
| Eswatini | 2021 | 157.63 | 171.03 | 0.92 | 2.58 | 3.03 | 0.85 | 1490.11 | 1311.74 | 1.14 | 0.59 |
| Ethiopia | 2021 | 163.70 | 126.56 | 1.29 | 3.43 | 2.43 | 1.41 | 1670.84 | 1375.88 | 1.21 | 0.36 |
| Federated States of Micronesia | 2021 | 448.54 | 374.17 | 1.20 | 10.26 | 8.93 | 1.15 | 1131.21 | 935.21 | 1.21 | 0.59 |
| Fiji | 2021 | 387.24 | 360.69 | 1.07 | 8.69 | 9.37 | 0.93 | 912.60 | 760.75 | 1.20 | 0.68 |
| Finland | 2021 | 15.94 | 16.82 | 0.95 | 0.69 | 0.74 | 0.93 | 19.82 | 14.72 | 1.35 | 0.86 |
| France | 2021 | 23.44 | 23.69 | 0.99 | 1.25 | 1.18 | 1.06 | 38.45 | 31.31 | 1.23 | 0.84 |
| Gabon | 2021 | 145.65 | 135.36 | 1.08 | 2.72 | 2.14 | 1.27 | 1621.55 | 1618.56 | 1.00 | 0.63 |
| Georgia | 2021 | 162.72 | 176.12 | 0.92 | 4.09 | 4.57 | 0.89 | 1172.47 | 982.25 | 1.19 | 0.85 |
| Georgia | 2021 | 162.72 | 176.12 | 0.92 | 4.09 | 4.57 | 0.89 | 1172.47 | 982.25 | 1.19 | 0.73 |
| Germany | 2021 | 41.20 | 41.18 | 1.00 | 2.54 | 2.25 | 1.13 | 43.23 | 33.93 | 1.27 | 0.90 |
| Ghana | 2021 | 85.41 | 91.38 | 0.93 | 1.23 | 1.52 | 0.81 | 1121.93 | 1023.91 | 1.10 | 0.56 |
| Greece | 2021 | 14.73 | 12.83 | 1.15 | 0.78 | 0.56 | 1.41 | 27.13 | 27.36 | 0.99 | 0.79 |
| Greenland | 2021 | 36.95 | 17.89 | 2.07 | 1.43 | 0.54 | 2.64 | 149.58 | 126.12 | 1.19 | 0.83 |
| Grenada | 2021 | 151.15 | 125.45 | 1.20 | 2.37 | 2.00 | 1.19 | 1306.29 | 1076.12 | 1.21 | 0.67 |
| Guam | 2021 | 112.89 | 142.02 | 0.79 | 1.36 | 2.20 | 0.62 | 1181.19 | 957.75 | 1.23 | 0.80 |
| Guatemala | 2021 | 66.93 | 51.72 | 1.29 | 0.19 | 0.13 | 1.48 | 1295.93 | 1005.61 | 1.29 | 0.54 |
| Guinea | 2021 | 132.76 | 133.87 | 0.99 | 2.91 | 3.26 | 0.89 | 939.57 | 863.37 | 1.09 | 0.34 |
| Guinea-Bissau | 2021 | 168.77 | 179.96 | 0.94 | 3.87 | 4.46 | 0.87 | 1138.22 | 1045.85 | 1.09 | 0.35 |
| Guyana | 2021 | 119.19 | 104.62 | 1.14 | 1.42 | 1.31 | 1.08 | 1278.81 | 1030.74 | 1.24 | 0.65 |
| Haiti | 2021 | 354.90 | 199.70 | 1.78 | 6.42 | 3.49 | 1.84 | 1359.25 | 1123.12 | 1.21 | 0.45 |
| Honduras | 2021 | 90.94 | 58.59 | 1.55 | 1.03 | 0.34 | 3.01 | 1297.89 | 1039.56 | 1.25 | 0.51 |
| Hungary | 2021 | 29.37 | 26.64 | 1.10 | 1.24 | 1.02 | 1.21 | 68.20 | 58.11 | 1.17 | 0.79 |
| Iceland | 2021 | 15.42 | 12.07 | 1.28 | 0.75 | 0.53 | 1.42 | 22.57 | 21.70 | 1.04 | 0.88 |
| India | 2021 | 465.93 | 397.00 | 1.17 | 15.23 | 13.32 | 1.14 | 799.35 | 587.21 | 1.36 | 0.58 |
| Indonesia | 2021 | 76.77 | 48.28 | 1.59 | 1.65 | 0.96 | 1.72 | 225.97 | 189.28 | 1.19 | 0.66 |
| Iran | 2021 | 74.68 | 65.03 | 1.15 | 1.58 | 1.20 | 1.32 | 758.11 | 663.04 | 1.14 | 0.70 |
| Iraq | 2021 | 95.71 | 81.56 | 1.17 | 2.01 | 1.72 | 1.17 | 786.95 | 700.15 | 1.12 | 0.66 |
| Ireland | 2021 | 19.26 | 11.70 | 1.65 | 0.92 | 0.55 | 1.68 | 35.65 | 25.18 | 1.42 | 0.87 |
| Israel | 2021 | 32.41 | 25.38 | 1.28 | 1.67 | 1.20 | 1.40 | 45.03 | 32.35 | 1.39 | 0.81 |
| Italy | 2021 | 35.74 | 29.36 | 1.22 | 1.79 | 1.30 | 1.38 | 74.58 | 61.46 | 1.21 | 0.81 |
| Jamaica | 2021 | 112.37 | 88.36 | 1.27 | 1.23 | 0.93 | 1.32 | 1325.50 | 1089.30 | 1.22 | 0.68 |
| Japan | 2021 | 14.24 | 12.97 | 1.10 | 0.82 | 0.70 | 1.18 | 42.57 | 28.09 | 1.52 | 0.87 |
| Jordan | 2021 | 18.70 | 15.13 | 1.24 | 0.57 | 0.39 | 1.46 | 48.01 | 35.73 | 1.34 | 0.73 |
| Kazakhstan | 2021 | 59.66 | 55.52 | 1.07 | 1.95 | 1.79 | 1.09 | 117.54 | 95.47 | 1.23 | 0.73 |
| Kenya | 2021 | 136.21 | 103.24 | 1.32 | 2.75 | 1.58 | 1.74 | 1599.22 | 1376.66 | 1.16 | 0.52 |
| Kiribati | 2021 | 459.50 | 551.07 | 0.83 | 10.56 | 12.87 | 0.82 | 1127.44 | 935.14 | 1.21 | 0.53 |
| Kuwait | 2021 | 16.38 | 14.49 | 1.13 | 0.42 | 0.37 | 1.12 | 58.69 | 43.91 | 1.34 | 0.85 |
| Kyrgyzstan | 2021 | 149.65 | 135.28 | 1.11 | 3.02 | 2.74 | 1.10 | 1085.77 | 918.58 | 1.18 | 0.60 |
| Laos | 2021 | 171.87 | 123.96 | 1.39 | 2.74 | 1.91 | 1.44 | 994.82 | 784.59 | 1.27 | 0.49 |
| Latvia | 2021 | 41.60 | 30.40 | 1.37 | 1.18 | 0.76 | 1.56 | 189.43 | 136.04 | 1.39 | 0.83 |
| Lebanon | 2021 | 34.29 | 30.67 | 1.12 | 1.12 | 1.01 | 1.12 | 55.05 | 43.75 | 1.26 | 0.74 |
| Lesotho | 2021 | 193.47 | 199.13 | 0.97 | 3.58 | 4.00 | 0.89 | 1501.88 | 1321.42 | 1.14 | 0.51 |
| Liberia | 2021 | 130.42 | 121.83 | 1.07 | 2.63 | 2.53 | 1.04 | 1165.75 | 1069.72 | 1.09 | 0.35 |
| Libya | 2021 | 100.82 | 75.43 | 1.34 | 1.67 | 1.05 | 1.59 | 766.73 | 673.45 | 1.14 | 0.73 |
| Lithuania | 2021 | 37.81 | 28.87 | 1.31 | 1.04 | 0.70 | 1.49 | 179.99 | 128.54 | 1.40 | 0.86 |
| Luxembourg | 2021 | 24.03 | 19.12 | 1.26 | 1.20 | 0.95 | 1.27 | 54.29 | 42.25 | 1.28 | 0.88 |
| Madagascar | 2021 | 294.99 | 242.09 | 1.22 | 8.17 | 6.05 | 1.35 | 1703.40 | 1482.83 | 1.15 | 0.40 |
| Malawi | 2021 | 164.41 | 142.99 | 1.15 | 3.35 | 2.63 | 1.27 | 1791.05 | 1539.54 | 1.16 | 0.38 |
| Malaysia | 2021 | 80.64 | 59.92 | 1.35 | 0.99 | 0.64 | 1.54 | 944.25 | 710.64 | 1.33 | 0.74 |
| Maldives | 2021 | 67.01 | 48.96 | 1.37 | 0.66 | 0.36 | 1.86 | 876.13 | 698.75 | 1.25 | 0.65 |
| Mali | 2021 | 145.11 | 118.18 | 1.23 | 2.79 | 2.23 | 1.25 | 1241.45 | 1143.21 | 1.09 | 0.27 |
| Malta | 2021 | 24.05 | 14.93 | 1.61 | 1.14 | 0.65 | 1.76 | 23.36 | 17.34 | 1.35 | 0.80 |
| Marshall Islands | 2021 | 542.07 | 424.51 | 1.28 | 11.97 | 10.25 | 1.17 | 1124.93 | 934.10 | 1.20 | 0.57 |
| Mauritania | 2021 | 110.03 | 98.01 | 1.12 | 2.13 | 1.82 | 1.17 | 1146.34 | 1061.00 | 1.08 | 0.50 |
| Mauritius | 2021 | 78.39 | 51.46 | 1.52 | 0.82 | 0.40 | 2.02 | 886.71 | 686.66 | 1.29 | 0.72 |
| Mexico | 2021 | 46.13 | 31.03 | 1.49 | 0.80 | 0.42 | 1.89 | 537.41 | 398.56 | 1.35 | 0.66 |
| Moldova | 2021 | 42.66 | 36.77 | 1.16 | 1.23 | 1.00 | 1.23 | 136.70 | 100.78 | 1.36 | 0.73 |
| Monaco | 2021 | 13.38 | 12.96 | 1.03 | 0.60 | 0.55 | 1.09 | 36.34 | 29.72 | 1.22 | 0.91 |
| Mongolia | 2021 | 156.37 | 162.16 | 0.96 | 3.32 | 3.57 | 0.93 | 1148.31 | 971.37 | 1.18 | 0.62 |
| Montenegro | 2021 | 38.52 | 37.75 | 1.02 | 1.52 | 1.37 | 1.11 | 61.58 | 57.84 | 1.06 | 0.80 |
| Morocco | 2021 | 110.00 | 76.46 | 1.44 | 2.33 | 1.55 | 1.50 | 794.45 | 707.97 | 1.12 | 0.56 |
| Mozambique | 2021 | 217.50 | 206.59 | 1.05 | 5.90 | 5.07 | 1.16 | 1757.11 | 1544.92 | 1.14 | 0.33 |
| Myanmar | 2021 | 135.82 | 113.91 | 1.19 | 1.95 | 1.79 | 1.09 | 1008.50 | 702.56 | 1.44 | 0.53 |
| Namibia | 2021 | 133.79 | 148.96 | 0.90 | 1.97 | 2.66 | 0.74 | 1523.69 | 1340.62 | 1.14 | 0.62 |
| Nauru | 2021 | 476.56 | 397.10 | 1.20 | 10.86 | 9.97 | 1.09 | 216.94 | 179.78 | 1.21 | 0.63 |
| Nepal | 2021 | 528.63 | 444.64 | 1.19 | 18.64 | 15.04 | 1.24 | 728.57 | 647.94 | 1.12 | 0.43 |
| Netherlands | 2021 | 17.39 | 11.60 | 1.50 | 1.02 | 0.64 | 1.59 | 33.25 | 26.54 | 1.25 | 0.89 |
| New Zealand | 2021 | 47.88 | 40.50 | 1.18 | 1.83 | 1.42 | 1.29 | 63.15 | 49.36 | 1.28 | 0.85 |
| Nicaragua | 2021 | 83.44 | 62.49 | 1.34 | 0.58 | 0.37 | 1.58 | 1379.28 | 1079.90 | 1.28 | 0.52 |
| Niger | 2021 | 155.83 | 150.17 | 1.04 | 3.45 | 3.83 | 0.90 | 1135.71 | 1041.72 | 1.09 | 0.17 |
| Nigeria | 2021 | 108.16 | 107.09 | 1.01 | 1.60 | 1.80 | 0.89 | 1279.37 | 1139.03 | 1.12 | 0.50 |
| Niue | 2021 | 366.83 | 260.73 | 1.41 | 7.18 | 6.17 | 1.16 | 161.40 | 135.04 | 1.20 | 0.73 |
| North Korea | 2021 | 193.04 | 141.77 | 1.36 | 6.59 | 4.94 | 1.33 | 751.56 | 671.83 | 1.12 | 0.57 |
| North Macedonia | 2021 | 45.22 | 33.31 | 1.36 | 1.97 | 1.31 | 1.51 | 57.18 | 53.20 | 1.07 | 0.75 |
| Northern Mariana Islands | 2021 | 197.61 | 123.76 | 1.60 | 4.14 | 2.26 | 1.83 | 1231.50 | 1005.53 | 1.22 | 0.77 |
| Norway | 2021 | 13.72 | 11.65 | 1.18 | 0.87 | 0.65 | 1.34 | 22.51 | 21.21 | 1.06 | 0.92 |
| Oman | 2021 | 39.69 | 24.86 | 1.60 | 1.30 | 0.90 | 1.45 | 70.72 | 48.69 | 1.45 | 0.77 |
| Pakistan | 2021 | 679.05 | 497.63 | 1.36 | 21.09 | 14.89 | 1.42 | 1088.61 | 894.99 | 1.22 | 0.50 |
| Palau | 2021 | 241.15 | 373.46 | 0.65 | 5.91 | 9.18 | 0.64 | 175.17 | 145.88 | 1.20 | 0.75 |
| Palestine | 2021 | 81.56 | 60.52 | 1.35 | 1.56 | 0.98 | 1.60 | 784.45 | 686.29 | 1.14 | 0.63 |
| Panama | 2021 | 81.01 | 62.68 | 1.29 | 0.60 | 0.46 | 1.31 | 1306.11 | 1018.82 | 1.28 | 0.71 |
| Papua New Guinea | 2021 | 578.06 | 572.80 | 1.01 | 11.60 | 14.42 | 0.80 | 1096.53 | 920.80 | 1.19 | 0.42 |
| Paraguay | 2021 | 94.78 | 76.12 | 1.25 | 0.94 | 0.76 | 1.23 | 1341.11 | 1083.63 | 1.24 | 0.64 |
| Peru | 2021 | 78.65 | 62.44 | 1.26 | 0.68 | 0.47 | 1.46 | 1240.53 | 1020.31 | 1.22 | 0.66 |
| Philippines | 2021 | 89.44 | 65.97 | 1.36 | 1.19 | 0.86 | 1.39 | 710.00 | 546.11 | 1.30 | 0.65 |
| Poland | 2021 | 28.52 | 35.09 | 0.81 | 1.33 | 1.36 | 0.98 | 50.25 | 54.97 | 0.91 | 0.81 |
| Portugal | 2021 | 27.09 | 24.13 | 1.12 | 1.33 | 1.08 | 1.23 | 37.07 | 34.23 | 1.08 | 0.74 |
| Puerto Rico | 2021 | 19.09 | 16.53 | 1.16 | 0.44 | 0.35 | 1.25 | 51.83 | 36.73 | 1.41 | 0.83 |
| Qatar | 2021 | 23.05 | 26.06 | 0.88 | 0.82 | 0.94 | 0.87 | 86.50 | 52.46 | 1.65 | 0.85 |
| Romania | 2021 | 33.43 | 41.48 | 0.81 | 1.34 | 1.54 | 0.87 | 81.20 | 70.76 | 1.15 | 0.77 |
| Russia | 2021 | 36.26 | 27.44 | 1.32 | 1.20 | 0.76 | 1.57 | 179.94 | 125.00 | 1.44 | 0.81 |
| Rwanda | 2021 | 159.74 | 123.20 | 1.30 | 3.41 | 2.19 | 1.56 | 1679.83 | 1446.49 | 1.16 | 0.44 |
| Saint Kitts and Nevis | 2021 | 24.65 | 20.81 | 1.18 | 0.61 | 0.52 | 1.18 | 61.65 | 40.03 | 1.54 | 0.75 |
| Saint Lucia | 2021 | 124.88 | 85.97 | 1.45 | 1.59 | 0.87 | 1.82 | 1353.68 | 1071.60 | 1.26 | 0.67 |
| Saint Vincent and the Grenadines | 2021 | 128.48 | 112.26 | 1.14 | 1.58 | 1.54 | 1.02 | 1296.33 | 1060.19 | 1.22 | 0.64 |
| Samoa | 2021 | 338.75 | 260.75 | 1.30 | 7.36 | 5.97 | 1.23 | 1192.78 | 993.98 | 1.20 | 0.59 |
| San Marino | 2021 | 26.36 | 17.87 | 1.48 | 1.24 | 0.78 | 1.59 | 56.04 | 37.56 | 1.49 | 0.89 |
| Sao Tome and Principe | 2021 | 191.62 | 120.86 | 1.59 | 5.11 | 2.47 | 2.07 | 1254.82 | 1130.59 | 1.11 | 0.51 |
| Saudi Arabia | 2021 | 40.01 | 16.85 | 2.37 | 1.15 | 0.44 | 2.59 | 72.27 | 46.92 | 1.54 | 0.82 |
| Senegal | 2021 | 115.08 | 119.16 | 0.97 | 2.12 | 2.47 | 0.86 | 1185.26 | 1084.26 | 1.09 | 0.41 |
| Serbia | 2021 | 30.53 | 25.33 | 1.21 | 1.26 | 0.96 | 1.31 | 54.61 | 54.77 | 1.00 | 0.79 |
| Seychelles | 2021 | 61.11 | 52.36 | 1.17 | 0.43 | 0.53 | 0.81 | 891.74 | 682.77 | 1.31 | 0.73 |
| Sierra Leone | 2021 | 135.67 | 139.29 | 0.97 | 2.62 | 3.04 | 0.86 | 1182.97 | 1081.09 | 1.09 | 0.36 |
| Singapore | 2021 | 13.17 | 9.68 | 1.36 | 0.63 | 0.46 | 1.37 | 29.72 | 20.79 | 1.43 | 0.86 |
| Slovakia | 2021 | 31.62 | 29.63 | 1.07 | 1.12 | 0.94 | 1.19 | 94.60 | 98.52 | 0.96 | 0.81 |
| Slovenia | 2021 | 41.96 | 35.16 | 1.19 | 2.35 | 1.72 | 1.37 | 63.50 | 52.29 | 1.21 | 0.84 |
| Solomon Islands | 2021 | 364.65 | 441.58 | 0.83 | 8.51 | 11.54 | 0.74 | 1093.40 | 912.71 | 1.20 | 0.43 |
| Somalia | 2021 | 246.59 | 193.47 | 1.27 | 6.60 | 4.58 | 1.44 | 1736.02 | 1496.81 | 1.16 | 0.08 |
| South Africa | 2021 | 135.44 | 118.01 | 1.15 | 1.74 | 1.48 | 1.17 | 1645.18 | 1432.75 | 1.15 | 0.68 |
| South Korea | 2021 | 10.62 | 7.39 | 1.44 | 0.56 | 0.36 | 1.55 | 30.42 | 20.15 | 1.51 | 0.89 |
| South Sudan | 2021 | 213.68 | 177.17 | 1.21 | 5.07 | 3.93 | 1.29 | 1708.27 | 1469.49 | 1.16 | 0.28 |
| Spain | 2021 | 42.28 | 29.42 | 1.44 | 2.23 | 1.45 | 1.54 | 66.88 | 41.91 | 1.60 | 0.77 |
| Sri Lanka | 2021 | 25.51 | 24.58 | 1.04 | 0.55 | 0.51 | 1.08 | 53.94 | 76.22 | 0.71 | 0.70 |
| Sudan | 2021 | 131.68 | 110.57 | 1.19 | 2.84 | 2.32 | 1.22 | 672.08 | 584.34 | 1.15 | 0.54 |
| Suriname | 2021 | 107.96 | 80.54 | 1.34 | 1.14 | 0.75 | 1.51 | 1228.37 | 1006.11 | 1.22 | 0.63 |
| Sweden | 2021 | 11.85 | 9.00 | 1.32 | 0.72 | 0.52 | 1.37 | 21.47 | 14.62 | 1.47 | 0.89 |
| Switzerland | 2021 | 16.65 | 15.00 | 1.11 | 0.78 | 0.71 | 1.10 | 43.80 | 35.62 | 1.23 | 0.93 |
| Syria | 2021 | 100.81 | 83.08 | 1.21 | 2.39 | 1.64 | 1.45 | 752.65 | 676.88 | 1.11 | 0.62 |
| Taiwan (Province of China) | 2021 | 20.53 | 17.33 | 1.18 | 0.63 | 0.56 | 1.12 | 162.03 | 116.79 | 1.39 | 0.87 |
| Tajikistan | 2021 | 123.42 | 109.76 | 1.12 | 1.94 | 1.76 | 1.10 | 1144.06 | 980.11 | 1.17 | 0.54 |
| Tanzania | 2021 | 172.16 | 135.54 | 1.27 | 3.88 | 2.47 | 1.57 | 1787.85 | 1536.73 | 1.16 | 0.45 |
| Thailand | 2021 | 56.26 | 44.47 | 1.27 | 0.41 | 0.31 | 1.31 | 823.83 | 617.80 | 1.33 | 0.68 |
| The Bahamas | 2021 | 90.81 | 71.29 | 1.27 | 0.72 | 0.51 | 1.41 | 1299.18 | 1041.71 | 1.25 | 0.81 |
| The Gambia | 2021 | 123.85 | 129.46 | 0.96 | 2.42 | 2.74 | 0.88 | 1188.92 | 1080.50 | 1.10 | 0.41 |
| Timor-Leste | 2021 | 153.30 | 125.72 | 1.22 | 2.62 | 2.08 | 1.26 | 884.46 | 689.54 | 1.28 | 0.44 |
| Togo | 2021 | 121.95 | 131.46 | 0.93 | 2.43 | 2.99 | 0.81 | 1144.26 | 1043.56 | 1.10 | 0.41 |
| Tokelau | 2021 | 456.16 | 262.42 | 1.74 | 9.14 | 6.07 | 1.51 | 179.34 | 119.63 | 1.50 | 0.69 |
| Tonga | 2021 | 184.62 | 156.17 | 1.18 | 3.03 | 2.64 | 1.15 | 1633.97 | 1365.46 | 1.20 | 0.63 |
| Trinidad and Tobago | 2021 | 109.77 | 90.14 | 1.22 | 1.13 | 0.94 | 1.21 | 1318.70 | 1049.12 | 1.26 | 0.77 |
| Tunisia | 2021 | 38.37 | 36.47 | 1.05 | 1.30 | 1.29 | 1.01 | 60.82 | 44.81 | 1.36 | 0.68 |
| Turkey | 2021 | 29.26 | 21.01 | 1.39 | 1.03 | 0.69 | 1.48 | 58.00 | 40.46 | 1.43 | 0.71 |
| Turkmenistan | 2021 | 171.08 | 143.60 | 1.19 | 3.17 | 2.72 | 1.17 | 1104.92 | 945.99 | 1.17 | 0.68 |
| Tuvalu | 2021 | 364.23 | 303.30 | 1.20 | 9.27 | 8.03 | 1.15 | 202.29 | 141.15 | 1.43 | 0.58 |
| Uganda | 2021 | 137.31 | 122.02 | 1.13 | 2.55 | 2.09 | 1.22 | 1664.40 | 1433.62 | 1.16 | 0.42 |
| UK | 2021 | 18.22 | 13.41 | 1.36 | 0.88 | 0.59 | 1.48 | 49.91 | 36.51 | 1.37 | 0.86 |
| Ukraine | 2021 | 37.10 | 34.32 | 1.08 | 1.03 | 0.87 | 1.19 | 141.85 | 97.60 | 1.45 | 0.76 |
| United Arab Emirates | 2021 | 116.15 | 73.68 | 1.58 | 4.19 | 1.39 | 3.01 | 764.44 | 673.72 | 1.13 | 0.85 |
| Uruguay | 2021 | 17.12 | 14.48 | 1.18 | 0.68 | 0.53 | 1.29 | 70.67 | 55.23 | 1.28 | 0.72 |
| USA | 2021 | 24.35 | 20.32 | 1.20 | 0.93 | 0.70 | 1.32 | 127.15 | 119.57 | 1.06 | 0.86 |
| Uzbekistan | 2021 | 228.47 | 183.06 | 1.25 | 5.37 | 4.23 | 1.27 | 1137.46 | 982.61 | 1.16 | 0.66 |
| Vanuatu | 2021 | 483.19 | 584.02 | 0.83 | 10.84 | 14.31 | 0.76 | 1246.18 | 1018.47 | 1.22 | 0.47 |
| Venezuela | 2021 | 18.33 | 13.62 | 1.35 | 0.49 | 0.38 | 1.26 | 53.39 | 35.22 | 1.52 | 0.60 |
| Vietnam | 2021 | 37.47 | 37.51 | 1.00 | 0.89 | 0.81 | 1.09 | 109.62 | 148.94 | 0.74 | 0.63 |
| Virgin Islands | 2021 | 24.79 | 30.62 | 0.81 | 0.54 | 0.57 | 0.94 | 59.17 | 42.75 | 1.38 | 0.82 |
| Yemen | 2021 | 156.36 | 120.98 | 1.29 | 3.52 | 2.82 | 1.25 | 892.52 | 807.67 | 1.11 | 0.45 |
| Zambia | 2021 | 176.24 | 133.82 | 1.32 | 4.11 | 2.56 | 1.60 | 1769.83 | 1510.80 | 1.17 | 0.51 |
| Zimbabwe | 2021 | 336.30 | 342.13 | 0.98 | 7.21 | 7.52 | 0.96 | 1497.71 | 1326.73 | 1.13 | 0.47 |
| Abbreviations: RHD = Rheumatic heart disease, ASDR = age-standardized DALYs rates, ASMR = age-standardized mortality rates, ASPR = age-standardized prevalence rates, SDI = Socio-Demographic Index. | | | | | | | | | | | |
