# Supplementary material for: Sex difference in the burden of rheumatic heart disease: Insights from the Global Burden of Disease Study 2021
Source: PLoS One. 2025 Oct 22;20(10):e0334914. doi: 10.1371/journal.pone.0334914 (PMC12543145; doi:10.1371/journal.pone.0334914)
Supplement: S6 Table — (DOCX) [file pone.0334914.s008.docx]

**S6 Table:** The EAPC of ASDR, ASMR, ASPR in female and male and the gender difference of RHD across 204 countries.

| **Country** | **The EAPC for ASDR (95% CI)** | | | **The EAPC for ASMR (95% CI)** | | **The EAPC for ASPR (95% CI)** | |
| --- | --- | --- | --- | --- | --- | --- | --- |
|  | **Female** | **Male** |  | **Female** | **Male** | **Female** | **Male** |
| Afghanistan | -1.97 (-2.1 , -1.84) | -2.9 (-3.14 , -2.66) |  | -1.84 (-1.97 , -1.71) | -3.1 (-3.38 , -2.83) | 0.06 (0.06 , 0.07) | 0.04 (0.03 , 0.05) |
| Albania | -2.7 (-3.01 , -2.39) | -2.89 (-3.21 , -2.57) |  | -4.36 (-4.82 , -3.9) | -4.96 (-5.47 , -4.45) | 0.02 (0 , 0.05) | -0.15 (-0.18 , -0.13) |
| Algeria | -2.03 (-2.12 , -1.95) | -2.07 (-2.18 , -1.97) |  | -1.7 (-1.91 , -1.48) | -2.56 (-2.69 , -2.42) | 0.3 (0.2 , 0.41) | 0.3 (0.2 , 0.4) |
| American Samoa | -0.62 (-0.7 , -0.54) | -1.6 (-1.7 , -1.5) |  | -1 (-1.11 , -0.88) | -2.07 (-2.21 , -1.94) | 0.1 (0.08 , 0.11) | 0.1 (0.1 , 0.11) |
| Andorra | -2.31 (-2.45 , -2.17) | -1.99 (-2.2 , -1.78) |  | -2.16 (-2.32 , -2) | -2.02 (-2.29 , -1.75) | -0.91 (-1.04 , -0.79) | -0.45 (-0.55 , -0.36) |
| Angola | -2.34 (-2.43 , -2.25) | -2.22 (-2.27 , -2.18) |  | -2.76 (-2.9 , -2.63) | -3.06 (-3.13 , -2.99) | 0 (-0.01 , 0.02) | 0.01 (0 , 0.03) |
| Antigua and Barbuda | -1.38 (-1.55 , -1.2) | -1.15 (-1.27 , -1.03) |  | -2.71 (-3.05 , -2.36) | -3.15 (-3.45 , -2.86) | 0.07 (0.05 , 0.08) | 0.01 (-0.01 , 0.02) |
| Argentina | -2.27 (-2.37 , -2.17) | -2.51 (-2.61 , -2.41) |  | -3.68 (-3.91 , -3.45) | -4.26 (-4.45 , -4.06) | 0.18 (0.15 , 0.21) | 0.16 (0.12 , 0.2) |
| Armenia | -2.17 (-2.37 , -1.96) | -2.4 (-2.63 , -2.17) |  | -2.28 (-2.72 , -1.82) | -3.35 (-3.76 , -2.93) | -0.15 (-0.16 , -0.14) | -0.24 (-0.26 , -0.22) |
| Australia | -2.53 (-2.84 , -2.22) | -2.18 (-2.55 , -1.82) |  | -2.31 (-2.69 , -1.92) | -1.97 (-2.4 , -1.53) | -0.8 (-0.98 , -0.62) | -0.48 (-0.65 , -0.32) |
| Austria | -2.87 (-3.29 , -2.45) | -2.23 (-2.67 , -1.79) |  | -1.81 (-2.25 , -1.38) | -1.07 (-1.53 , -0.61) | -5.2 (-5.56 , -4.83) | -5.37 (-5.8 , -4.95) |
| Azerbaijan | -1.75 (-1.99 , -1.5) | -1.61 (-1.81 , -1.4) |  | -2.38 (-2.79 , -1.96) | -2.25 (-2.57 , -1.93) | -0.06 (-0.06 , -0.05) | -0.15 (-0.17 , -0.13) |
| Bahamas | -1.23 (-1.34 , -1.12) | -1.06 (-1.19 , -0.92) |  | -2.78 (-3.01 , -2.55) | -2.62 (-2.95 , -2.29) | 0.08 (0.07 , 0.09) | 0.02 (0.01 , 0.03) |
| Bahrain | -5.86 (-6.36 , -5.35) | -6.33 (-6.94 , -5.71) |  | -5.83 (-6.29 , -5.36) | -6.55 (-7.14 , -5.95) | -0.69 (-0.77 , -0.62) | -0.88 (-0.93 , -0.83) |
| Bangladesh | -1.85 (-1.99 , -1.72) | -2.31 (-2.52 , -2.11) |  | -1.69 (-1.9 , -1.49) | -2.1 (-2.41 , -1.8) | 0.03 (-0.02 , 0.09) | 0.27 (0.23 , 0.32) |
| Barbados | -1.15 (-1.27 , -1.04) | -1.16 (-1.33 , -0.99) |  | -2.56 (-2.79 , -2.32) | -3.49 (-3.96 , -3.02) | 0.14 (0.12 , 0.16) | 0.1 (0.08 , 0.12) |
| Belarus | -3.47 (-4.07 , -2.86) | -3.63 (-4.49 , -2.75) |  | -3.57 (-4.22 , -2.92) | -3.69 (-4.6 , -2.77) | -1.77 (-1.84 , -1.69) | -1.73 (-1.81 , -1.65) |
| Belgium | -1 (-1.25 , -0.74) | -1.13 (-1.32 , -0.94) |  | -0.51 (-0.83 , -0.18) | -0.83 (-1.08 , -0.57) | 0 (-0.81 , 0.82) | -0.46 (-1.37 , 0.47) |
| Belize | -1.28 (-1.45 , -1.11) | -0.97 (-1.21 , -0.73) |  | -2.71 (-3.09 , -2.33) | -1.94 (-2.5 , -1.39) | 0.09 (0.08 , 0.1) | 0.02 (0.01 , 0.04) |
| Benin | -2.53 (-2.85 , -2.22) | -2.08 (-2.28 , -1.88) |  | -4.04 (-4.44 , -3.64) | -2.92 (-3.12 , -2.72) | 0.16 (0.12 , 0.19) | 0.08 (0.05 , 0.11) |
| Bermuda | -4.51 (-4.89 , -4.13) | -3.39 (-3.68 , -3.1) |  | -4.82 (-5.24 , -4.39) | -3.34 (-3.64 , -3.04) | -1.41 (-1.58 , -1.23) | -1.31 (-1.43 , -1.18) |
| Bhutan | -2.85 (-2.96 , -2.73) | -3.03 (-3.09 , -2.96) |  | -2.38 (-2.48 , -2.27) | -2.86 (-2.95 , -2.78) | 0.06 (0.04 , 0.08) | 0.01 (0 , 0.03) |
| Bolivia (Plurinational State of) | -2.68 (-2.85 , -2.52) | -1.82 (-1.93 , -1.71) |  | -3.59 (-3.76 , -3.42) | -3.14 (-3.27 , -3.01) | 0.03 (0.02 , 0.04) | -0.03 (-0.04 , -0.02) |
| Bosnia and Herzegovina | -5 (-5.38 , -4.61) | -5.04 (-5.53 , -4.55) |  | -4.87 (-5.28 , -4.46) | -5.01 (-5.49 , -4.53) | -0.89 (-1.07 , -0.7) | 0.17 (0.06 , 0.27) |
| Botswana | -1.79 (-2.24 , -1.35) | -2.6 (-2.73 , -2.48) |  | -3.31 (-3.97 , -2.65) | -3.95 (-4.17 , -3.74) | -0.04 (-0.04 , -0.03) | -0.03 (-0.05 , -0.01) |
| Brazil | -1.63 (-1.7 , -1.56) | -1.66 (-1.73 , -1.59) |  | -2.93 (-3.09 , -2.76) | -2.82 (-2.97 , -2.68) | 0.09 (0.08 , 0.1) | -0.11 (-0.13 , -0.09) |
| Brunei Darussalam | -2.32 (-2.45 , -2.18) | -1.93 (-2.22 , -1.64) |  | -1.93 (-2.09 , -1.76) | -1.19 (-1.59 , -0.78) | -1.1 (-1.19 , -1.02) | -1.04 (-1.12 , -0.96) |
| Bulgaria | -4.69 (-5.19 , -4.18) | -4.64 (-5.24 , -4.03) |  | -4.7 (-5.2 , -4.21) | -4.56 (-5.16 , -3.96) | -1.78 (-2.03 , -1.53) | -1.58 (-1.89 , -1.26) |
| Burkina Faso | -2.34 (-2.63 , -2.04) | -1.64 (-1.84 , -1.45) |  | -2.84 (-3.2 , -2.48) | -1.98 (-2.2 , -1.76) | 0.04 (0.02 , 0.05) | -0.01 (-0.02 , -0.01) |
| Burundi | -2.62 (-2.81 , -2.42) | -2.43 (-2.63 , -2.23) |  | -3.38 (-3.62 , -3.14) | -3.29 (-3.53 , -3.06) | 0.21 (0.19 , 0.22) | 0.16 (0.15 , 0.17) |
| Cabo Verde | -3.55 (-4.28 , -2.81) | -2.65 (-2.98 , -2.32) |  | -6.15 (-7.29 , -5) | -4.09 (-4.61 , -3.56) | 0.07 (0.05 , 0.09) | -0.01 (-0.03 , 0.01) |
| Cambodia | -3.5 (-3.67 , -3.33) | -3.31 (-3.46 , -3.16) |  | -3.94 (-4.12 , -3.77) | -4.13 (-4.28 , -3.98) | -0.17 (-0.25 , -0.1) | -0.23 (-0.31 , -0.15) |
| Cameroon | -2.88 (-3.06 , -2.71) | -1.56 (-1.71 , -1.41) |  | -4.27 (-4.51 , -4.03) | -2.54 (-2.75 , -2.34) | 0.28 (0.22 , 0.34) | 0.19 (0.13 , 0.24) |
| Canada | -2.81 (-3.22 , -2.4) | -2.15 (-2.56 , -1.74) |  | -2.56 (-3.02 , -2.1) | -2.14 (-2.59 , -1.69) | -0.82 (-0.9 , -0.73) | -0.44 (-0.52 , -0.36) |
| Central African Republic | -1.52 (-1.6 , -1.43) | -1.48 (-1.54 , -1.42) |  | -1.76 (-1.87 , -1.65) | -1.86 (-1.94 , -1.79) | 0.06 (0.04 , 0.07) | 0.05 (0.04 , 0.06) |
| Chad | -2.24 (-2.44 , -2.04) | -1.37 (-1.43 , -1.31) |  | -3 (-3.27 , -2.74) | -1.85 (-1.93 , -1.76) | 0.07 (0.05 , 0.09) | 0 (-0.01 , 0.01) |
| Chile | -6.32 (-6.58 , -6.05) | -5.93 (-6.14 , -5.71) |  | -6.08 (-6.37 , -5.79) | -5.75 (-5.99 , -5.51) | -2.17 (-2.31 , -2.03) | -1.76 (-1.9 , -1.62) |
| China | -5.38 (-5.53 , -5.23) | -4.1 (-4.21 , -3.98) |  | -5.54 (-5.71 , -5.38) | -4.19 (-4.32 , -4.06) | -0.17 (-0.31 , -0.02) | -0.27 (-0.41 , -0.13) |
| Colombia | -7.52 (-7.93 , -7.1) | -7.05 (-7.5 , -6.59) |  | -8.37 (-8.95 , -7.8) | -7.64 (-8.22 , -7.06) | -1.07 (-1.34 , -0.81) | -1.04 (-1.24 , -0.84) |
| Comoros | -1.75 (-1.89 , -1.62) | -1.69 (-1.87 , -1.51) |  | -2.45 (-2.6 , -2.3) | -2.76 (-2.98 , -2.55) | 0.12 (0.11 , 0.14) | 0.09 (0.08 , 0.1) |
| Congo | -2.31 (-2.48 , -2.13) | -2.56 (-2.7 , -2.43) |  | -2.88 (-3.08 , -2.68) | -3.86 (-3.97 , -3.75) | -0.18 (-0.3 , -0.07) | -0.21 (-0.32 , -0.09) |
| Cook Islands | -2.86 (-3.1 , -2.62) | -1.9 (-2.07 , -1.74) |  | -2.94 (-3.16 , -2.72) | -2.14 (-2.28 , -1.99) | -0.21 (-0.39 , -0.02) | 0 (-0.06 , 0.05) |
| Costa Rica | -2.53 (-2.67 , -2.39) | -2.14 (-2.26 , -2.01) |  | -6.19 (-6.4 , -5.97) | -5.46 (-5.77 , -5.16) | 0.02 (0.01 , 0.03) | 0.03 (0.02 , 0.04) |
| Croatia | -4.79 (-5.48 , -4.09) | -5.39 (-6.15 , -4.61) |  | -4.08 (-4.95 , -3.2) | -4.89 (-5.79 , -3.98) | -1.7 (-2.01 , -1.4) | -2.46 (-2.74 , -2.17) |
| Cuba | -1.94 (-2.1 , -1.78) | -1.51 (-1.69 , -1.33) |  | -3 (-3.31 , -2.68) | -2.43 (-2.79 , -2.07) | -0.04 (-0.06 , -0.02) | -0.11 (-0.12 , -0.09) |
| Cyprus | -4.19 (-4.42 , -3.96) | -3.32 (-3.51 , -3.12) |  | -4.06 (-4.38 , -3.73) | -3.36 (-3.62 , -3.09) | -2.37 (-2.88 , -1.86) | -1.61 (-1.99 , -1.24) |
| Czechia | -5.15 (-5.77 , -4.53) | -5.43 (-6.16 , -4.69) |  | -4.2 (-4.87 , -3.53) | -4.7 (-5.5 , -3.9) | -1.17 (-1.46 , -0.88) | -1.22 (-1.55 , -0.88) |
| Côte d'Ivoire | -1.88 (-2.12 , -1.64) | -2.11 (-2.2 , -2.02) |  | -3.11 (-3.42 , -2.8) | -3.04 (-3.16 , -2.93) | 0.1 (0.08 , 0.11) | 0.03 (0.01 , 0.04) |
| Democratic People's Republic of Korea | -2.06 (-2.12 , -1.99) | -1.86 (-1.91 , -1.81) |  | -2.21 (-2.32 , -2.09) | -2.17 (-2.23 , -2.11) | -0.28 (-0.31 , -0.25) | -0.3 (-0.34 , -0.26) |
| Democratic Republic of the Congo | -1.41 (-1.48 , -1.33) | -1.34 (-1.43 , -1.26) |  | -1.64 (-1.75 , -1.52) | -2.08 (-2.24 , -1.92) | -0.01 (-0.04 , 0.02) | -0.03 (-0.06 , 0) |
| Denmark | -2.01 (-2.25 , -1.76) | -1.49 (-1.75 , -1.23) |  | -1.09 (-1.39 , -0.79) | -0.68 (-0.97 , -0.38) | -1.93 (-2.63 , -1.21) | -2.03 (-3.2 , -0.83) |
| Djibouti | -1.5 (-1.57 , -1.43) | -1.64 (-1.73 , -1.55) |  | -2.28 (-2.38 , -2.18) | -2.76 (-2.89 , -2.64) | 0.2 (0.18 , 0.22) | 0.15 (0.14 , 0.16) |
| Dominica | -1.19 (-1.36 , -1.02) | -1.03 (-1.14 , -0.91) |  | -2.17 (-2.39 , -1.96) | -2.24 (-2.4 , -2.08) | 0.02 (0 , 0.04) | -0.03 (-0.05 , -0.01) |
| Dominican Republic | -1.34 (-1.45 , -1.22) | -0.92 (-1.05 , -0.79) |  | -2.19 (-2.34 , -2.04) | -1.81 (-2 , -1.61) | 0.06 (0.06 , 0.07) | 0.02 (0.01 , 0.02) |
| Ecuador | -2.23 (-2.42 , -2.04) | -1.87 (-2.06 , -1.68) |  | -4.58 (-5.03 , -4.13) | -4 (-4.42 , -3.57) | 0.12 (0.1 , 0.13) | 0.03 (0.02 , 0.04) |
| Egypt | -3.58 (-3.78 , -3.39) | -3.2 (-3.26 , -3.14) |  | -2.63 (-2.78 , -2.49) | -3.17 (-3.28 , -3.06) | 0.3 (0.24 , 0.35) | 0.27 (0.21 , 0.34) |
| El Salvador | -1.33 (-1.48 , -1.17) | -1.08 (-1.23 , -0.94) |  | -4.68 (-5.04 , -4.33) | -4.73 (-5.11 , -4.35) | 0.05 (0.04 , 0.06) | 0.03 (0.03 , 0.04) |
| Equatorial Guinea | -4.16 (-4.54 , -3.78) | -4.16 (-4.59 , -3.73) |  | -6.03 (-6.49 , -5.56) | -6.88 (-7.42 , -6.35) | -0.11 (-0.14 , -0.07) | -0.13 (-0.16 , -0.09) |
| Eritrea | -1.71 (-1.87 , -1.56) | -2.05 (-2.14 , -1.96) |  | -2.17 (-2.34 , -2.01) | -2.8 (-2.91 , -2.69) | 0.15 (0.05 , 0.25) | 0.12 (-0.02 , 0.26) |
| Estonia | -7.09 (-7.49 , -6.68) | -8.36 (-8.88 , -7.83) |  | -7.32 (-7.71 , -6.92) | -8.74 (-9.28 , -8.2) | -1.65 (-1.76 , -1.54) | -2.04 (-2.23 , -1.86) |
| Eswatini | -1.34 (-1.69 , -0.99) | -1.2 (-1.48 , -0.92) |  | -2.09 (-2.56 , -1.61) | -1.93 (-2.27 , -1.58) | 0.02 (0.01 , 0.03) | 0.03 (0.01 , 0.05) |
| Ethiopia | -3.06 (-3.23 , -2.89) | -2.62 (-2.71 , -2.53) |  | -4.24 (-4.49 , -3.99) | -3.89 (-4.06 , -3.72) | 0.51 (0.45 , 0.57) | 0.59 (0.53 , 0.65) |
| Fiji | -2.01 (-2.11 , -1.9) | -1.89 (-1.95 , -1.83) |  | -2.27 (-2.4 , -2.13) | -2.25 (-2.33 , -2.17) | 1.26 (0.89 , 1.63) | 1.16 (0.81 , 1.52) |
| Finland | -3.1 (-3.26 , -2.94) | -2.67 (-2.84 , -2.51) |  | -3.2 (-3.36 , -3.05) | -2.51 (-2.65 , -2.38) | -3.61 (-4.42 , -2.79) | -3.83 (-4.64 , -3.01) |
| France | -2.78 (-3.11 , -2.44) | -2.23 (-2.56 , -1.9) |  | -2.62 (-3.03 , -2.2) | -2.15 (-2.55 , -1.75) | -1.14 (-1.21 , -1.06) | -0.81 (-0.91 , -0.7) |
| Gabon | -1.97 (-2.11 , -1.83) | -2.01 (-2.08 , -1.95) |  | -3 (-3.23 , -2.77) | -3.48 (-3.52 , -3.44) | 0.06 (0.05 , 0.08) | 0.04 (0.03 , 0.06) |
| Gambia | -2.41 (-2.76 , -2.06) | -1.71 (-1.89 , -1.52) |  | -3.51 (-3.98 , -3.04) | -2.51 (-2.74 , -2.27) | 0.19 (0.18 , 0.21) | 0.12 (0.1 , 0.13) |
| Georgia | -2.08 (-2.49 , -1.68) | -1.59 (-2.01 , -1.16) |  | -1.04 (-1.6 , -0.48) | -0.57 (-1.16 , 0.03) | 0.02 (-0.02 , 0.05) | -0.07 (-0.11 , -0.03) |
| Germany | -1.99 (-2.48 , -1.5) | -1.16 (-1.7 , -0.62) |  | -1.09 (-1.52 , -0.67) | -0.4 (-0.88 , 0.08) | -0.97 (-1.2 , -0.74) | -0.46 (-0.71 , -0.21) |
| Ghana | -3.44 (-3.72 , -3.15) | -2.32 (-2.51 , -2.13) |  | -5.57 (-6.01 , -5.13) | -3.68 (-3.96 , -3.39) | 0.03 (0.02 , 0.05) | -0.05 (-0.06 , -0.04) |
| Greece | -1.65 (-2.37 , -0.93) | -0.84 (-1.38 , -0.29) |  | -1.49 (-2.32 , -0.65) | -1.03 (-1.73 , -0.33) | -1.22 (-1.49 , -0.95) | -0.78 (-0.94 , -0.62) |
| Greenland | -5.07 (-5.42 , -4.73) | -3.68 (-3.84 , -3.51) |  | -5.43 (-5.8 , -5.06) | -4.24 (-4.44 , -4.04) | -1.81 (-1.93 , -1.68) | -0.55 (-0.57 , -0.53) |
| Grenada | -2.13 (-2.41 , -1.84) | -1.62 (-1.87 , -1.37) |  | -2.73 (-3.15 , -2.31) | -2.06 (-2.55 , -1.58) | 0.02 (0.01 , 0.03) | -0.03 (-0.04 , -0.02) |
| Guam | -1.39 (-1.84 , -0.93) | -0.17 (-0.3 , -0.03) |  | -3.31 (-4.01 , -2.61) | -0.79 (-1.02 , -0.56) | 0.16 (0.15 , 0.18) | 0.16 (0.15 , 0.17) |
| Guatemala | -1.56 (-1.81 , -1.3) | -1.29 (-1.46 , -1.12) |  | -7.13 (-7.45 , -6.81) | -7.02 (-7.28 , -6.77) | 0.34 (0.29 , 0.38) | 0.34 (0.29 , 0.38) |
| Guinea | -3.11 (-3.41 , -2.81) | -1.83 (-1.9 , -1.77) |  | -3.9 (-4.25 , -3.55) | -2.43 (-2.5 , -2.37) | 0.27 (0.23 , 0.32) | 0.2 (0.15 , 0.25) |
| Guinea-Bissau | -3.24 (-3.51 , -2.98) | -2.27 (-2.37 , -2.17) |  | -3.78 (-4.11 , -3.46) | -2.77 (-2.87 , -2.67) | 0.15 (0.13 , 0.17) | 0.1 (0.08 , 0.11) |
| Guyana | -1.51 (-1.71 , -1.3) | -1.35 (-1.52 , -1.18) |  | -2.49 (-2.81 , -2.17) | -2.11 (-2.36 , -1.85) | 0.03 (0.01 , 0.04) | 0 (-0.01 , 0.01) |
| Haiti | -2.18 (-2.28 , -2.07) | -1.67 (-1.83 , -1.51) |  | -2.37 (-2.45 , -2.29) | -2.06 (-2.23 , -1.89) | 0.2 (0.18 , 0.21) | 0.13 (0.11 , 0.15) |
| Honduras | -0.63 (-0.71 , -0.56) | -0.42 (-0.45 , -0.38) |  | -1.07 (-1.31 , -0.83) | -1.64 (-1.78 , -1.49) | 0.04 (0.02 , 0.06) | 0.03 (0.02 , 0.05) |
| Hungary | -6.18 (-6.85 , -5.5) | -6.63 (-7.28 , -5.97) |  | -5.57 (-6.26 , -4.86) | -6.05 (-6.71 , -5.39) | -2.18 (-2.57 , -1.8) | -2.31 (-2.68 , -1.93) |
| Iceland | -1.9 (-2.14 , -1.66) | -1.05 (-1.4 , -0.7) |  | -1.56 (-1.79 , -1.33) | -0.87 (-1.17 , -0.57) | -1.41 (-1.51 , -1.3) | -0.38 (-0.51 , -0.24) |
| India | -2.03 (-2.14 , -1.91) | -2.63 (-2.83 , -2.44) |  | -1.73 (-1.89 , -1.57) | -2.34 (-2.54 , -2.15) | 0.06 (0.01 , 0.12) | 0.24 (0.15 , 0.34) |
| Indonesia | -3.42 (-3.53 , -3.31) | -2.66 (-2.72 , -2.6) |  | -3.33 (-3.46 , -3.21) | -2.95 (-3.03 , -2.88) | 0.24 (0.2 , 0.29) | 0.4 (0.35 , 0.45) |
| Iran (Islamic Republic of) | -2.43 (-2.65 , -2.21) | -2.07 (-2.31 , -1.83) |  | -2.81 (-3.14 , -2.48) | -2.81 (-3.18 , -2.44) | 0.08 (0.07 , 0.09) | 0.04 (0.03 , 0.06) |
| Iraq | -2.59 (-2.77 , -2.42) | -2.58 (-2.65 , -2.51) |  | -2.88 (-3.07 , -2.69) | -3.28 (-3.4 , -3.15) | 0.05 (0.04 , 0.06) | 0.05 (0.03 , 0.06) |
| Ireland | -3.19 (-3.4 , -2.98) | -2.78 (-2.94 , -2.61) |  | -2.92 (-3.16 , -2.68) | -2.64 (-2.84 , -2.44) | -1.68 (-1.85 , -1.51) | -0.99 (-1.14 , -0.85) |
| Israel | -2.75 (-2.96 , -2.54) | -1.79 (-1.94 , -1.64) |  | -2.33 (-2.59 , -2.07) | -1.47 (-1.64 , -1.29) | -1.25 (-1.29 , -1.21) | -0.39 (-0.43 , -0.35) |
| Italy | -2.85 (-3.06 , -2.63) | -2.26 (-2.58 , -1.94) |  | -2.03 (-2.25 , -1.81) | -1.64 (-1.96 , -1.31) | -1.15 (-1.45 , -0.84) | -0.54 (-0.81 , -0.28) |
| Jamaica | -1.34 (-1.54 , -1.14) | -1.56 (-1.87 , -1.25) |  | -2.43 (-2.73 , -2.13) | -2.71 (-3.25 , -2.16) | 0.21 (0.18 , 0.23) | 0.16 (0.14 , 0.18) |
| Japan | -4.19 (-4.36 , -4.02) | -3.48 (-3.58 , -3.37) |  | -3.65 (-3.78 , -3.53) | -3.09 (-3.17 , -3.01) | -1.58 (-1.68 , -1.47) | -1.32 (-1.43 , -1.2) |
| Jordan | -4.33 (-4.72 , -3.94) | -2.54 (-2.66 , -2.43) |  | -3.81 (-4.26 , -3.37) | -2.66 (-2.81 , -2.51) | -0.89 (-0.97 , -0.81) | -0.45 (-0.54 , -0.36) |
| Kazakhstan | -6.48 (-6.92 , -6.04) | -6.46 (-7.01 , -5.9) |  | -5.73 (-6.12 , -5.34) | -5.71 (-6.19 , -5.24) | -1.97 (-2.06 , -1.88) | -1.79 (-1.9 , -1.68) |
| Kenya | -0.56 (-0.69 , -0.44) | -0.62 (-0.75 , -0.48) |  | -0.94 (-1.16 , -0.71) | -1.43 (-1.68 , -1.18) | 0.19 (0.17 , 0.2) | 0.06 (0.03 , 0.09) |
| Kiribati | -1.27 (-1.36 , -1.18) | -1.17 (-1.23 , -1.11) |  | -1.23 (-1.33 , -1.13) | -1.26 (-1.33 , -1.2) | 0.06 (0.05 , 0.07) | 0.08 (0.07 , 0.08) |
| Kuwait | -6.05 (-7.14 , -4.95) | -6.1 (-7.51 , -4.66) |  | -6.26 (-7.5 , -5) | -6.15 (-7.74 , -4.53) | -1.13 (-1.21 , -1.05) | -0.72 (-0.77 , -0.67) |
| Kyrgyzstan | -3.21 (-3.58 , -2.83) | -3.31 (-3.81 , -2.82) |  | -3.48 (-3.93 , -3.03) | -3.6 (-4.16 , -3.03) | -0.06 (-0.07 , -0.05) | -0.15 (-0.17 , -0.12) |
| Lao People's Democratic Republic | -3.3 (-3.38 , -3.22) | -3.18 (-3.22 , -3.15) |  | -3.89 (-3.99 , -3.78) | -4.03 (-4.1 , -3.96) | -0.04 (-0.17 , 0.09) | -0.06 (-0.23 , 0.11) |
| Latvia | -5.71 (-6.1 , -5.32) | -6.96 (-7.5 , -6.42) |  | -5.89 (-6.29 , -5.48) | -7.21 (-7.77 , -6.66) | -1.76 (-2.09 , -1.43) | -2.06 (-2.23 , -1.9) |
| Lebanon | -5.76 (-6.04 , -5.48) | -4.66 (-5.04 , -4.28) |  | -5.75 (-6 , -5.5) | -4.98 (-5.37 , -4.59) | -0.81 (-0.89 , -0.73) | -0.3 (-0.38 , -0.22) |
| Lesotho | 0.54 (0.14 , 0.95) | -0.41 (-0.61 , -0.2) |  | 0.5 (-0.06 , 1.07) | -0.96 (-1.19 , -0.73) | 0 (-0.01 , 0.01) | 0.03 (0.02 , 0.05) |
| Liberia | -3.05 (-3.41 , -2.69) | -2.27 (-2.46 , -2.09) |  | -4.01 (-4.37 , -3.65) | -3.3 (-3.51 , -3.09) | 0.17 (0.16 , 0.19) | 0.1 (0.08 , 0.11) |
| Libya | -0.84 (-1.02 , -0.65) | -1.36 (-1.6 , -1.12) |  | -1.02 (-1.25 , -0.8) | -2.08 (-2.39 , -1.77) | 0.31 (0.3 , 0.32) | 0.3 (0.29 , 0.31) |
| Lithuania | -6.61 (-6.91 , -6.32) | -7.51 (-7.98 , -7.04) |  | -6.67 (-7.03 , -6.32) | -7.69 (-8.24 , -7.14) | -3.32 (-3.77 , -2.88) | -2.79 (-3.07 , -2.52) |
| Luxembourg | -2.16 (-2.31 , -2.01) | -2.42 (-2.59 , -2.25) |  | -1.76 (-1.99 , -1.53) | -1.98 (-2.21 , -1.76) | -1.24 (-2.13 , -0.36) | -0.35 (-1.02 , 0.33) |
| Madagascar | -1.13 (-1.17 , -1.09) | -1.1 (-1.16 , -1.03) |  | -1.31 (-1.37 , -1.26) | -1.41 (-1.5 , -1.33) | 0.18 (0.17 , 0.19) | 0.16 (0.15 , 0.17) |
| Malawi | -1.45 (-1.54 , -1.35) | -1.08 (-1.22 , -0.94) |  | -2.11 (-2.29 , -1.93) | -1.77 (-2.02 , -1.53) | 0.16 (0.14 , 0.17) | 0.14 (0.13 , 0.15) |
| Malaysia | -2.29 (-2.57 , -2.01) | -2.07 (-2.37 , -1.77) |  | -3.77 (-4.05 , -3.49) | -3.68 (-4.02 , -3.33) | 0.16 (0.13 , 0.19) | 0.13 (0.1 , 0.17) |
| Maldives | -4.59 (-4.92 , -4.25) | -2.93 (-3.16 , -2.71) |  | -6.69 (-6.98 , -6.4) | -5.49 (-5.75 , -5.23) | -0.01 (-0.02 , 0) | -0.07 (-0.08 , -0.06) |
| Mali | -3.13 (-3.49 , -2.78) | -1.31 (-1.53 , -1.1) |  | -4.12 (-4.53 , -3.7) | -2.06 (-2.36 , -1.76) | 0.08 (0.06 , 0.1) | -0.02 (-0.04 , 0) |
| Malta | -2.46 (-2.58 , -2.34) | -2.14 (-2.24 , -2.04) |  | -2.5 (-2.62 , -2.38) | -2.23 (-2.33 , -2.12) | -1.98 (-2.43 , -1.52) | -1.57 (-2.03 , -1.1) |
| Marshall Islands | -1.28 (-1.48 , -1.07) | -2.14 (-2.2 , -2.07) |  | -1.67 (-1.87 , -1.48) | -2.44 (-2.5 , -2.37) | 0.08 (0.07 , 0.09) | 0.08 (0.07 , 0.08) |
| Mauritania | -3.36 (-3.7 , -3.03) | -2.39 (-2.58 , -2.2) |  | -4.78 (-5.18 , -4.38) | -3.69 (-3.9 , -3.47) | 0.07 (0.04 , 0.1) | -0.01 (-0.03 , 0.02) |
| Mauritius | -1.85 (-2.19 , -1.5) | -1.09 (-1.33 , -0.84) |  | -3.75 (-4.19 , -3.3) | -3.08 (-3.45 , -2.71) | -0.05 (-0.08 , -0.03) | -0.07 (-0.11 , -0.04) |
| Mexico | -4.7 (-4.88 , -4.52) | -3.89 (-4.08 , -3.69) |  | -6.15 (-6.31 , -5.99) | -5.73 (-5.94 , -5.53) | -0.58 (-0.62 , -0.54) | -0.55 (-0.59 , -0.51) |
| Micronesia (Federated States of) | -2.02 (-2.15 , -1.89) | -2.2 (-2.3 , -2.09) |  | -2.24 (-2.39 , -2.09) | -2.51 (-2.62 , -2.39) | 0.05 (0.05 , 0.06) | 0.07 (0.06 , 0.08) |
| Monaco | -1.4 (-1.67 , -1.14) | -1.4 (-1.45 , -1.36) |  | -1.31 (-1.59 , -1.02) | -1.49 (-1.53 , -1.44) | -0.43 (-0.5 , -0.36) | -0.4 (-0.48 , -0.32) |
| Mongolia | -3.35 (-3.56 , -3.14) | -2.39 (-2.61 , -2.18) |  | -3.77 (-4.03 , -3.51) | -3.22 (-3.47 , -2.96) | -0.01 (-0.02 , 0) | -0.09 (-0.1 , -0.08) |
| Montenegro | -1.97 (-2.31 , -1.62) | -1.87 (-2.1 , -1.64) |  | -1.45 (-1.73 , -1.16) | -1.56 (-1.81 , -1.31) | -0.06 (-0.13 , 0.01) | 0.22 (0.08 , 0.36) |
| Morocco | -2.11 (-2.18 , -2.04) | -2.15 (-2.23 , -2.06) |  | -1.94 (-2.03 , -1.85) | -2.33 (-2.45 , -2.22) | 0.04 (0.02 , 0.06) | 0 (-0.01 , 0.02) |
| Mozambique | -0.59 (-0.71 , -0.47) | -0.18 (-0.34 , -0.01) |  | -0.64 (-0.82 , -0.46) | -0.32 (-0.53 , -0.11) | 0.04 (-0.01 , 0.09) | 0.05 (-0.03 , 0.13) |
| Myanmar | -4.21 (-4.41 , -4.01) | -3.48 (-3.6 , -3.36) |  | -4.97 (-5.24 , -4.7) | -4.25 (-4.4 , -4.09) | -0.24 (-0.33 , -0.15) | 0.05 (0.03 , 0.08) |
| Namibia | -2.11 (-2.21 , -2) | -1.89 (-1.99 , -1.79) |  | -3.38 (-3.57 , -3.19) | -2.76 (-2.89 , -2.62) | -0.04 (-0.06 , -0.03) | -0.06 (-0.08 , -0.04) |
| Nauru | -1.3 (-1.74 , -0.86) | -1.59 (-1.99 , -1.19) |  | -1.52 (-1.92 , -1.12) | -1.71 (-2.09 , -1.32) | -0.82 (-0.86 , -0.78) | -0.59 (-0.65 , -0.52) |
| Nepal | -2.1 (-2.35 , -1.85) | -2.7 (-2.83 , -2.57) |  | -1.71 (-1.99 , -1.44) | -2.64 (-2.78 , -2.49) | 0.17 (0.1 , 0.25) | 0.14 (0.06 , 0.21) |
| Netherlands | -0.6 (-1.41 , 0.21) | -1.15 (-1.8 , -0.48) |  | 0.41 (-0.71 , 1.55) | -0.46 (-1.3 , 0.39) | -0.05 (-0.26 , 0.17) | -0.14 (-0.3 , 0.01) |
| New Zealand | -3.27 (-3.63 , -2.91) | -3.16 (-3.68 , -2.63) |  | -3.02 (-3.45 , -2.58) | -3.07 (-3.65 , -2.49) | -0.74 (-0.81 , -0.67) | 0.24 (0.05 , 0.44) |
| Nicaragua | -1.96 (-2.05 , -1.87) | -1.65 (-1.75 , -1.55) |  | -4.74 (-4.86 , -4.62) | -4.9 (-5.01 , -4.78) | 0.05 (0.04 , 0.06) | 0 (-0.05 , 0.06) |
| Niger | -3.42 (-3.78 , -3.06) | -1.98 (-2.14 , -1.82) |  | -3.94 (-4.36 , -3.51) | -2.39 (-2.55 , -2.23) | 0.17 (0.15 , 0.19) | 0.09 (0.07 , 0.11) |
| Nigeria | -3.94 (-4.22 , -3.66) | -2.53 (-2.59 , -2.48) |  | -5.9 (-6.21 , -5.59) | -4.04 (-4.11 , -3.97) | 0.43 (0.39 , 0.46) | 0.21 (0.17 , 0.25) |
| Niue | -1.69 (-2.3 , -1.08) | -1.73 (-1.94 , -1.51) |  | -2.07 (-2.47 , -1.66) | -1.97 (-2.12 , -1.82) | -0.82 (-0.86 , -0.79) | -0.56 (-0.59 , -0.53) |
| North Macedonia | -4.19 (-4.41 , -3.96) | -4.17 (-4.43 , -3.91) |  | -3.61 (-3.82 , -3.39) | -3.95 (-4.23 , -3.67) | -1.79 (-1.94 , -1.64) | -0.94 (-1.05 , -0.84) |
| Northern Mariana Islands | -0.81 (-1 , -0.61) | 0.12 (-0.04 , 0.28) |  | -1.12 (-1.4 , -0.85) | 0.13 (-0.13 , 0.4) | 0.14 (0.11 , 0.17) | 0.16 (0.13 , 0.19) |
| Norway | -3.77 (-4.97 , -2.55) | -3.89 (-5.03 , -2.74) |  | -3.52 (-4.79 , -2.24) | -3.65 (-4.81 , -2.47) | -2.01 (-2.65 , -1.36) | -2.41 (-3.48 , -1.33) |
| Oman | -3.93 (-4.08 , -3.79) | -3.29 (-3.65 , -2.92) |  | -3.59 (-3.77 , -3.4) | -3.08 (-3.46 , -2.71) | 0.32 (0.19 , 0.44) | 0.6 (0.46 , 0.74) |
| Pakistan | -1.1 (-1.41 , -0.79) | -1.82 (-2 , -1.63) |  | -1.27 (-1.59 , -0.94) | -2.14 (-2.33 , -1.95) | 0.42 (0.28 , 0.56) | 0.29 (0.15 , 0.44) |
| Palau | -0.92 (-1.06 , -0.79) | -1.26 (-1.38 , -1.14) |  | -1.08 (-1.22 , -0.93) | -1.55 (-1.69 , -1.41) | -0.82 (-0.88 , -0.76) | -0.41 (-0.44 , -0.38) |
| Palestine | -2.07 (-2.14 , -2) | -2.46 (-2.62 , -2.3) |  | -2.68 (-2.78 , -2.57) | -3.85 (-3.94 , -3.75) | 0.01 (0 , 0.03) | 0 (-0.01 , 0.01) |
| Panama | -2.41 (-2.6 , -2.22) | -2.04 (-2.18 , -1.91) |  | -5.73 (-6.02 , -5.45) | -5.38 (-5.65 , -5.12) | 0.01 (0 , 0.03) | 0 (-0.01 , 0.01) |
| Papua New Guinea | -1.45 (-1.54 , -1.35) | -1.44 (-1.49 , -1.39) |  | -1.68 (-1.75 , -1.6) | -1.65 (-1.69 , -1.6) | 0.12 (0.09 , 0.14) | 0.12 (0.1 , 0.15) |
| Paraguay | -0.94 (-1 , -0.89) | -0.83 (-0.87 , -0.79) |  | -1.95 (-2.07 , -1.82) | -1.71 (-1.8 , -1.63) | 0.07 (0.06 , 0.07) | -0.03 (-0.04 , -0.02) |
| Peru | -1.66 (-1.85 , -1.47) | -1.22 (-1.35 , -1.08) |  | -4.26 (-4.62 , -3.9) | -3.7 (-3.97 , -3.42) | 0.16 (0.13 , 0.19) | 0.08 (0.05 , 0.11) |
| Philippines | -1.2 (-1.36 , -1.04) | -1.49 (-1.63 , -1.35) |  | -1.97 (-2.1 , -1.83) | -2.42 (-2.53 , -2.31) | 0.38 (0.3 , 0.46) | 0.32 (0.24 , 0.4) |
| Poland | -7.17 (-7.69 , -6.65) | -7.18 (-7.74 , -6.62) |  | -6.17 (-6.73 , -5.61) | -6.59 (-7.18 , -6.01) | -3.72 (-4.85 , -2.58) | -3.13 (-4.1 , -2.15) |
| Portugal | -4.13 (-4.51 , -3.75) | -2.8 (-3.21 , -2.39) |  | -3.53 (-3.83 , -3.23) | -2.47 (-2.82 , -2.11) | -2.15 (-2.33 , -1.97) | -1.16 (-1.42 , -0.9) |
| Puerto Rico | -3.43 (-3.8 , -3.05) | -2.67 (-3.21 , -2.13) |  | -3.55 (-3.93 , -3.18) | -2.78 (-3.34 , -2.21) | -1.01 (-1.17 , -0.86) | -0.3 (-0.4 , -0.21) |
| Qatar | -3.75 (-4.02 , -3.49) | -4.43 (-4.64 , -4.21) |  | -3.66 (-4.07 , -3.25) | -4.79 (-5.1 , -4.47) | -1.39 (-1.45 , -1.34) | -0.84 (-0.92 , -0.76) |
| Republic of Korea | -4.08 (-4.19 , -3.96) | -4.12 (-4.26 , -3.98) |  | -3.54 (-3.7 , -3.38) | -3.84 (-4.02 , -3.67) | -1.21 (-1.32 , -1.09) | -0.79 (-0.89 , -0.69) |
| Republic of Moldova | -6.85 (-7.22 , -6.48) | -7.1 (-7.55 , -6.65) |  | -6.66 (-7.05 , -6.28) | -7.04 (-7.49 , -6.58) | -2.24 (-2.3 , -2.18) | -2.17 (-2.29 , -2.06) |
| Romania | -6.7 (-7.17 , -6.22) | -5.87 (-6.44 , -5.29) |  | -5.94 (-6.4 , -5.47) | -5.16 (-5.73 , -4.58) | -1.31 (-1.78 , -0.84) | -0.61 (-1.11 , -0.11) |
| Russian Federation | -6.74 (-7.02 , -6.46) | -7.66 (-8.16 , -7.17) |  | -6.21 (-6.5 , -5.92) | -7.4 (-7.88 , -6.91) | -1.95 (-2.06 , -1.84) | -2.07 (-2.16 , -1.98) |
| Rwanda | -3.53 (-3.87 , -3.2) | -2.53 (-2.8 , -2.26) |  | -4.57 (-4.98 , -4.16) | -3.72 (-4.07 , -3.36) | 0.04 (0.03 , 0.05) | 0 (-0.01 , 0.01) |
| Saint Kitts and Nevis | -5.25 (-5.58 , -4.93) | -3.53 (-4.01 , -3.06) |  | -4.83 (-5.1 , -4.55) | -3.28 (-3.73 , -2.84) | -2.24 (-2.45 , -2.03) | -1.15 (-1.29 , -1.02) |
| Saint Lucia | -2.48 (-2.72 , -2.23) | -1.84 (-1.99 , -1.68) |  | -4.21 (-4.57 , -3.85) | -3.62 (-3.92 , -3.32) | 0.03 (0.01 , 0.04) | -0.01 (-0.02 , 0.01) |
| Saint Vincent and the Grenadines | -1.84 (-2.07 , -1.61) | -1.64 (-1.79 , -1.49) |  | -2.87 (-3.2 , -2.53) | -2.41 (-2.64 , -2.17) | 0.03 (0.02 , 0.03) | -0.02 (-0.04 , -0.01) |
| Samoa | -0.59 (-0.76 , -0.42) | -1.12 (-1.29 , -0.96) |  | -0.89 (-1.09 , -0.69) | -1.53 (-1.71 , -1.35) | 0.07 (0.04 , 0.09) | 0.09 (0.07 , 0.11) |
| San Marino | -2.1 (-2.37 , -1.83) | -1.97 (-2.29 , -1.64) |  | -2.43 (-2.76 , -2.09) | -2.06 (-2.49 , -1.64) | -0.63 (-0.66 , -0.61) | -0.55 (-0.59 , -0.51) |
| Sao Tome and Principe | -2.05 (-2.38 , -1.72) | -1.62 (-1.76 , -1.49) |  | -2.26 (-2.61 , -1.9) | -2.33 (-2.5 , -2.16) | 0.16 (0.14 , 0.18) | 0.1 (0.07 , 0.13) |
| Saudi Arabia | -3.97 (-4.07 , -3.87) | -4.2 (-4.42 , -3.99) |  | -3.79 (-3.91 , -3.68) | -4.48 (-4.69 , -4.27) | -0.8 (-0.91 , -0.69) | -0.78 (-0.83 , -0.74) |
| Senegal | -2.95 (-3.3 , -2.6) | -2.32 (-2.52 , -2.11) |  | -3.98 (-4.44 , -3.53) | -3.2 (-3.45 , -2.95) | 0 (-0.03 , 0.04) | -0.09 (-0.12 , -0.06) |
| Serbia | -3.98 (-4.27 , -3.69) | -3.84 (-4.07 , -3.61) |  | -4.05 (-4.39 , -3.72) | -3.86 (-4.09 , -3.64) | -0.54 (-0.64 , -0.45) | -0.54 (-0.82 , -0.26) |
| Seychelles | -1.07 (-1.18 , -0.96) | -1.71 (-1.88 , -1.54) |  | -2.4 (-2.54 , -2.26) | -3.05 (-3.26 , -2.84) | -0.08 (-0.1 , -0.05) | -0.11 (-0.13 , -0.09) |
| Sierra Leone | -2.32 (-2.51 , -2.14) | -1.87 (-1.96 , -1.79) |  | -3.16 (-3.36 , -2.95) | -2.63 (-2.71 , -2.56) | 0.15 (0.14 , 0.17) | 0.1 (0.08 , 0.11) |
| Singapore | -5.03 (-5.14 , -4.92) | -4.84 (-4.98 , -4.7) |  | -4.57 (-4.7 , -4.45) | -4.4 (-4.54 , -4.25) | -3.61 (-3.94 , -3.29) | -2.49 (-2.75 , -2.23) |
| Slovakia | -3.47 (-3.63 , -3.32) | -3.9 (-4.05 , -3.75) |  | -3.3 (-3.49 , -3.11) | -3.88 (-4.1 , -3.65) | -0.41 (-1.03 , 0.22) | -0.61 (-1.31 , 0.1) |
| Slovenia | -5.11 (-5.57 , -4.66) | -5.63 (-6.21 , -5.04) |  | -3.57 (-4.01 , -3.12) | -4.32 (-4.91 , -3.72) | -1.82 (-2.4 , -1.23) | -1.72 (-2.46 , -0.98) |
| Solomon Islands | -0.99 (-1.09 , -0.89) | -1.58 (-1.63 , -1.53) |  | -1.25 (-1.35 , -1.15) | -1.8 (-1.85 , -1.75) | 0.06 (0.04 , 0.07) | 0.06 (0.06 , 0.07) |
| Somalia | -1.56 (-1.68 , -1.44) | -1.58 (-1.71 , -1.46) |  | -1.87 (-2.01 , -1.73) | -2.07 (-2.23 , -1.92) | 0.17 (0.15 , 0.18) | 0.15 (0.14 , 0.16) |
| South Africa | -1.93 (-2.27 , -1.59) | -1.36 (-1.59 , -1.13) |  | -2.62 (-3.06 , -2.17) | -2.26 (-2.69 , -1.82) | -0.01 (-0.03 , 0.01) | 0.03 (0 , 0.05) |
| South Sudan | -0.93 (-1.21 , -0.64) | -1.14 (-1.34 , -0.94) |  | -1.36 (-1.71 , -1.02) | -1.71 (-1.91 , -1.51) | 0.24 (0.22 , 0.26) | 0.19 (0.18 , 0.21) |
| Spain | -4.03 (-4.17 , -3.88) | -3.29 (-3.46 , -3.12) |  | -3.43 (-3.55 , -3.31) | -2.58 (-2.72 , -2.44) | -1.3 (-1.48 , -1.13) | -0.63 (-0.8 , -0.45) |
| Sri Lanka | -4.24 (-4.41 , -4.08) | -3.97 (-4.19 , -3.75) |  | -4.23 (-4.35 , -4.1) | -4.13 (-4.33 , -3.93) | -0.7 (-0.87 , -0.53) | -0.14 (-0.19 , -0.09) |
| Sudan | -3.66 (-3.72 , -3.61) | -3.09 (-3.23 , -2.96) |  | -3.22 (-3.29 , -3.16) | -3.17 (-3.3 , -3.04) | 0.13 (0.12 , 0.15) | 0.14 (0.12 , 0.16) |
| Suriname | -1.66 (-1.88 , -1.44) | -1.37 (-1.46 , -1.27) |  | -2.89 (-3.17 , -2.6) | -2.47 (-2.62 , -2.32) | 0 (-0.02 , 0.01) | -0.05 (-0.07 , -0.04) |
| Sweden | -3.5 (-3.84 , -3.16) | -3.34 (-3.68 , -3) |  | -3.12 (-3.46 , -2.78) | -3.02 (-3.37 , -2.67) | -2.86 (-3.35 , -2.37) | -3.2 (-3.99 , -2.41) |
| Switzerland | -3.49 (-3.61 , -3.36) | -3.33 (-3.45 , -3.2) |  | -3.59 (-3.78 , -3.4) | -3.2 (-3.36 , -3.04) | -1.69 (-1.91 , -1.47) | -2.35 (-2.62 , -2.08) |
| Syrian Arab Republic | -3.83 (-4.23 , -3.42) | -3.36 (-3.68 , -3.04) |  | -3.88 (-4.31 , -3.45) | -3.84 (-4.17 , -3.51) | 0.01 (0 , 0.03) | -0.02 (-0.03 , 0) |
| Taiwan (Province of China) | -6.33 (-6.78 , -5.87) | -5.15 (-5.76 , -4.53) |  | -7.51 (-8 , -7.02) | -5.9 (-6.59 , -5.19) | -1.69 (-1.92 , -1.45) | -0.71 (-0.97 , -0.46) |
| Tajikistan | -1.95 (-2.11 , -1.79) | -1.68 (-1.81 , -1.56) |  | -2.62 (-2.9 , -2.35) | -2.63 (-2.89 , -2.37) | 0.05 (0.02 , 0.07) | -0.02 (-0.04 , -0.01) |
| Thailand | -1.53 (-1.72 , -1.35) | -1.47 (-1.6 , -1.34) |  | -3.12 (-3.47 , -2.77) | -3.11 (-3.36 , -2.86) | -0.07 (-0.08 , -0.06) | -0.11 (-0.12 , -0.1) |
| Timor-Leste | -2.34 (-2.73 , -1.95) | -1.85 (-2.15 , -1.56) |  | -2.69 (-3.08 , -2.31) | -2.35 (-2.65 , -2.04) | -0.03 (-0.05 , -0.02) | -0.05 (-0.06 , -0.04) |
| Togo | -2.77 (-3.06 , -2.47) | -1.82 (-1.89 , -1.76) |  | -3.73 (-4.09 , -3.36) | -2.47 (-2.57 , -2.36) | 0.05 (0.04 , 0.07) | -0.02 (-0.04 , 0) |
| Tokelau | -2.63 (-3.17 , -2.08) | -2.55 (-2.89 , -2.22) |  | -2.93 (-3.29 , -2.58) | -2.95 (-3.15 , -2.74) | -1.15 (-1.21 , -1.1) | -0.52 (-0.57 , -0.47) |
| Tonga | -1.34 (-1.4 , -1.27) | -0.92 (-1.01 , -0.83) |  | -1.91 (-1.97 , -1.85) | -1.65 (-1.73 , -1.57) | 0.03 (-0.12 , 0.18) | 0.01 (-0.17 , 0.2) |
| Trinidad and Tobago | -1.89 (-2.06 , -1.71) | -1.23 (-1.38 , -1.08) |  | -3.53 (-3.82 , -3.24) | -2.46 (-2.76 , -2.17) | 0.12 (0.11 , 0.13) | 0.09 (0.08 , 0.1) |
| Tunisia | -2.84 (-2.99 , -2.68) | -1.75 (-2.01 , -1.5) |  | -2.12 (-2.25 , -1.98) | -1.24 (-1.49 , -0.98) | -0.8 (-0.87 , -0.73) | -0.39 (-0.41 , -0.36) |
| Turkey | -3.02 (-3.46 , -2.58) | -3.2 (-3.58 , -2.81) |  | -2.19 (-2.78 , -1.6) | -3.06 (-3.52 , -2.6) | -0.7 (-0.87 , -0.53) | -0.26 (-0.37 , -0.15) |
| Turkmenistan | -2.89 (-3.12 , -2.66) | -2.93 (-3.27 , -2.6) |  | -3.52 (-3.82 , -3.21) | -3.68 (-4.13 , -3.23) | 0.02 (0 , 0.03) | -0.07 (-0.08 , -0.06) |
| Tuvalu | -2.99 (-3.15 , -2.84) | -2.69 (-2.81 , -2.57) |  | -2.94 (-3.08 , -2.79) | -2.74 (-2.86 , -2.63) | -1.1 (-1.18 , -1.03) | -0.64 (-0.7 , -0.58) |
| Uganda | -1.73 (-1.9 , -1.56) | -2.05 (-2.22 , -1.88) |  | -2.91 (-3.16 , -2.67) | -3.48 (-3.67 , -3.29) | 0.03 (-0.06 , 0.12) | 0.01 (-0.1 , 0.12) |
| Ukraine | -3.1 (-3.71 , -2.48) | -3.63 (-4.54 , -2.71) |  | -3.28 (-3.91 , -2.64) | -3.81 (-4.73 , -2.88) | -0.72 (-0.77 , -0.66) | -0.7 (-0.76 , -0.64) |
| United Arab Emirates | -0.47 (-0.75 , -0.19) | -2.43 (-2.63 , -2.23) |  | 1.39 (0.87 , 1.91) | -2.76 (-3.25 , -2.27) | 0.01 (-0.03 , 0.04) | -0.04 (-0.07 , -0.01) |
| United Kingdom | -5.11 (-5.34 , -4.88) | -3.96 (-4.2 , -3.71) |  | -4.94 (-5.16 , -4.72) | -3.95 (-4.21 , -3.7) | -0.27 (-0.3 , -0.23) | -0.35 (-0.39 , -0.32) |
| United Republic of Tanzania | -1.09 (-1.13 , -1.04) | -1.21 (-1.25 , -1.17) |  | -1.78 (-1.84 , -1.72) | -2.23 (-2.3 , -2.16) | 0.28 (0.25 , 0.3) | 0.24 (0.22 , 0.25) |
| United States of America | -3.71 (-4.28 , -3.14) | -2.88 (-3.45 , -2.31) |  | -3.57 (-4.09 , -3.04) | -2.99 (-3.56 , -2.42) | -1.28 (-1.73 , -0.84) | -0.41 (-0.7 , -0.13) |
| United States Virgin Islands | -3.58 (-3.72 , -3.45) | -1.18 (-1.43 , -0.94) |  | -3.87 (-4.01 , -3.73) | -1.66 (-1.89 , -1.43) | -0.74 (-0.81 , -0.68) | -0.26 (-0.34 , -0.18) |
| Uruguay | -3.42 (-3.57 , -3.27) | -3.32 (-3.44 , -3.2) |  | -3.43 (-3.62 , -3.24) | -3.56 (-3.69 , -3.42) | -0.36 (-0.5 , -0.22) | -0.08 (-0.2 , 0.04) |
| Uzbekistan | -0.49 (-0.66 , -0.31) | -0.23 (-0.43 , -0.04) |  | 0.01 (-0.19 , 0.21) | 0.24 (-0.04 , 0.53) | 0.28 (0.25 , 0.32) | 0.22 (0.19 , 0.24) |
| Vanuatu | -1.38 (-1.5 , -1.26) | -1.68 (-1.75 , -1.61) |  | -1.65 (-1.76 , -1.54) | -1.92 (-1.99 , -1.86) | 0.03 (0.02 , 0.04) | 0.05 (0.04 , 0.05) |
| Venezuela (Bolivarian Republic of) | -5.67 (-6.12 , -5.22) | -5.06 (-5.43 , -4.69) |  | -5.87 (-6.29 , -5.45) | -5.28 (-5.7 , -4.85) | -1.24 (-1.46 , -1.01) | -1 (-1.13 , -0.87) |
| Viet Nam | -3.22 (-3.44 , -3) | -2.72 (-2.93 , -2.52) |  | -3.41 (-3.55 , -3.27) | -3.32 (-3.45 , -3.18) | 1.02 (0.62 , 1.43) | 1.99 (1.59 , 2.4) |
| Yemen | -2.54 (-2.65 , -2.44) | -2.98 (-3.12 , -2.83) |  | -2.42 (-2.56 , -2.29) | -3.43 (-3.62 , -3.24) | 0.15 (-0.05 , 0.34) | 0.08 (-0.13 , 0.3) |
| Zambia | -1.99 (-2.1 , -1.88) | -1.36 (-1.54 , -1.18) |  | -2.64 (-2.82 , -2.46) | -2.12 (-2.41 , -1.83) | 0.04 (0 , 0.08) | 0 (-0.02 , 0.02) |
| Zimbabwe | 1.91 (1.24 , 2.57) | 1.04 (0.66 , 1.42) |  | 1.86 (1.08 , 2.64) | 0.68 (0.28 , 1.07) | 0.1 (0.06 , 0.13) | 0.07 (0.06 , 0.09) |
| Abbreviations: RHD = Rheumatic heart disease, ASDR = age-standardized DALYs rates, ASMR = age-standardized mortality rates, ASPR = age-standardized prevalence rates, EAPC  =  Estimated Annual Percentage Change, CI = Confidence Intervals. | | | | | | | |
