# Supplementary material for: Sex difference in the burden of rheumatic heart disease: Insights from the Global Burden of Disease Study 2021
Source: PLoS One. 2025 Oct 22;20(10):e0334914. doi: 10.1371/journal.pone.0334914 (PMC12543145; doi:10.1371/journal.pone.0334914)
Supplement: S7 Table — (DOCX) [file pone.0334914.s009.docx]

**S7 Table:** Global Values of Female/Male in 2021 across all age groups among 204 countries.

| **Country** | **Age Group** | **Female/Male** | | |
| --- | --- | --- | --- | --- |
|  |  | **ASDR** | **ASMR** | **ASPR** |
| Ukraine | <5 years | 0.49 | 0.18 | 1.04 |
| Brunei Darussalam | <5 years | 0.50 | 0.45 | 0.95 |
| United Arab Emirates | <5 years | 0.52 | 0.41 | 1.11 |
| Norway | <5 years | 0.54 | 0.45 | 0.81 |
| Madagascar | <5 years | 0.54 | 0.45 | 1.04 |
| Mozambique | <5 years | 0.54 | 0.33 | 1.03 |
| Azerbaijan | <5 years | 0.62 | 0.27 | 1.15 |
| Sao Tome and Principe | <5 years | 0.68 | 0.46 | 1.00 |
| Colombia | <5 years | 0.70 | 0.47 | 1.20 |
| Qatar | <5 years | 0.72 | 0.66 | 1.34 |
| Belarus | <5 years | 0.73 | 0.34 | 0.99 |
| Vanuatu | <5 years | 0.74 | 0.67 | 1.16 |
| Sudan | <5 years | 0.74 | 0.73 | 1.12 |
| Venezuela (Bolivarian Republic of) | <5 years | 0.75 | 0.64 | 1.20 |
| Tunisia | <5 years | 0.75 | 0.72 | 1.30 |
| Guam | <5 years | 0.76 | 0.63 | 1.15 |
| Nepal | <5 years | 0.77 | 0.74 | 0.98 |
| Samoa | <5 years | 0.77 | 0.68 | 1.17 |
| Maldives | <5 years | 0.78 | 0.52 | 1.14 |
| Gabon | <5 years | 0.79 | 0.67 | 0.88 |
| North Macedonia | <5 years | 0.79 | 0.58 | 1.11 |
| Djibouti | <5 years | 0.79 | 0.62 | 1.03 |
| Republic of Moldova | <5 years | 0.79 | 0.72 | 0.99 |
| Afghanistan | <5 years | 0.81 | 0.80 | 1.11 |
| Cook Islands | <5 years | 0.81 | 0.73 | 1.48 |
| Malawi | <5 years | 0.82 | 0.70 | 1.04 |
| Bhutan | <5 years | 0.82 | 0.81 | 1.07 |
| Fiji | <5 years | 0.82 | 0.80 | 1.17 |
| Egypt | <5 years | 0.83 | 0.83 | 1.09 |
| Palau | <5 years | 0.84 | 0.81 | 1.36 |
| Uganda | <5 years | 0.84 | 0.74 | 1.04 |
| United States of America | <5 years | 0.84 | 0.82 | 0.92 |
| Ghana | <5 years | 0.86 | 0.77 | 0.99 |
| Lesotho | <5 years | 0.86 | 0.78 | 1.02 |
| Zambia | <5 years | 0.86 | 0.75 | 1.03 |
| Canada | <5 years | 0.87 | 0.84 | 0.90 |
| Lebanon | <5 years | 0.87 | 0.83 | 1.34 |
| Burundi | <5 years | 0.87 | 0.79 | 1.04 |
| Micronesia (Federated States of) | <5 years | 0.87 | 0.79 | 1.16 |
| Dominican Republic | <5 years | 0.87 | 0.68 | 1.10 |
| Eritrea | <5 years | 0.87 | 0.82 | 1.03 |
| Kuwait | <5 years | 0.88 | 0.85 | 1.37 |
| Eswatini | <5 years | 0.88 | 0.84 | 1.00 |
| Uzbekistan | <5 years | 0.88 | 0.84 | 1.14 |
| Syrian Arab Republic | <5 years | 0.89 | 0.88 | 1.11 |
| Japan | <5 years | 0.90 | 0.99 | 0.89 |
| United Republic of Tanzania | <5 years | 0.91 | 0.85 | 1.03 |
| Somalia | <5 years | 0.91 | 0.87 | 1.04 |
| Equatorial Guinea | <5 years | 0.91 | 1.03 | 0.85 |
| South Africa | <5 years | 0.92 | 0.86 | 1.04 |
| Tajikistan | <5 years | 0.93 | 0.89 | 1.15 |
| Congo | <5 years | 0.93 | 0.96 | 0.87 |
| Viet Nam | <5 years | 0.93 | 0.89 | 1.09 |
| Austria | <5 years | 0.93 | 0.98 | 0.81 |
| Timor-Leste | <5 years | 0.93 | 0.92 | 1.14 |
| India | <5 years | 0.94 | 0.92 | 1.15 |
| Uruguay | <5 years | 0.96 | 0.87 | 1.06 |
| South Sudan | <5 years | 0.97 | 0.96 | 1.03 |
| Oman | <5 years | 0.97 | 0.95 | 1.35 |
| Guatemala | <5 years | 0.97 | 0.57 | 1.04 |
| Peru | <5 years | 0.98 | 0.75 | 1.08 |
| Panama | <5 years | 0.99 | 0.68 | 1.06 |
| Latvia | <5 years | 0.99 | 0.78 | 0.99 |
| Chile | <5 years | 0.99 | 1.01 | 1.07 |
| Honduras | <5 years | 1.00 | 0.63 | 1.05 |
| Costa Rica | <5 years | 1.01 | 0.42 | 1.07 |
| Namibia | <5 years | 1.01 | 1.00 | 1.02 |
| Tonga | <5 years | 1.01 | 0.90 | 1.16 |
| Kenya | <5 years | 1.01 | 0.85 | 1.04 |
| Cyprus | <5 years | 1.01 | 0.95 | 1.25 |
| Nicaragua | <5 years | 1.02 | 0.74 | 1.05 |
| Ethiopia | <5 years | 1.02 | 0.98 | 1.09 |
| El Salvador | <5 years | 1.03 | 0.46 | 1.06 |
| Solomon Islands | <5 years | 1.03 | 0.99 | 1.15 |
| Seychelles | <5 years | 1.04 | 0.87 | 1.16 |
| Iraq | <5 years | 1.04 | 1.03 | 1.10 |
| Mauritania | <5 years | 1.04 | 1.06 | 0.99 |
| Brazil | <5 years | 1.05 | 0.90 | 1.12 |
| Russian Federation | <5 years | 1.05 | 1.07 | 1.02 |
| Kiribati | <5 years | 1.05 | 1.05 | 1.16 |
| Mexico | <5 years | 1.05 | 1.03 | 1.07 |
| Rwanda | <5 years | 1.05 | 1.06 | 1.03 |
| China | <5 years | 1.06 | 1.06 | 1.06 |
| Republic of Korea | <5 years | 1.06 | 1.18 | 0.93 |
| Democratic People's Republic of Korea | <5 years | 1.06 | 1.05 | 1.05 |
| Turkmenistan | <5 years | 1.06 | 1.02 | 1.15 |
| Lao People's Democratic Republic | <5 years | 1.06 | 1.06 | 1.12 |
| Saint Vincent and the Grenadines | <5 years | 1.06 | 1.16 | 1.10 |
| Jordan | <5 years | 1.06 | 1.05 | 1.37 |
| Suriname | <5 years | 1.07 | 1.07 | 1.09 |
| Mongolia | <5 years | 1.07 | 1.04 | 1.14 |
| Sri Lanka | <5 years | 1.07 | 1.01 | 1.13 |
| Belize | <5 years | 1.07 | 1.08 | 1.09 |
| Lithuania | <5 years | 1.08 | 1.04 | 0.98 |
| Georgia | <5 years | 1.08 | 1.08 | 1.14 |
| Paraguay | <5 years | 1.09 | 1.15 | 1.09 |
| Taiwan (Province of China) | <5 years | 1.10 | 0.90 | 1.32 |
| Australia | <5 years | 1.10 | 1.02 | 1.08 |
| Democratic Republic of the Congo | <5 years | 1.11 | 1.24 | 0.87 |
| Armenia | <5 years | 1.11 | 0.95 | 1.15 |
| Denmark | <5 years | 1.11 | 1.10 | 1.16 |
| Iran (Islamic Republic of) | <5 years | 1.11 | 1.07 | 1.12 |
| Estonia | <5 years | 1.11 | 1.52 | 0.98 |
| Saudi Arabia | <5 years | 1.11 | 1.03 | 1.40 |
| Bolivia (Plurinational State of) | <5 years | 1.12 | 1.17 | 1.09 |
| Argentina | <5 years | 1.12 | 1.04 | 1.13 |
| Malaysia | <5 years | 1.12 | 1.20 | 1.15 |
| Italy | <5 years | 1.13 | 1.11 | 1.17 |
| Thailand | <5 years | 1.14 | 1.09 | 1.16 |
| Botswana | <5 years | 1.14 | 1.17 | 1.02 |
| Cuba | <5 years | 1.15 | 1.32 | 1.10 |
| Bahamas | <5 years | 1.15 | 1.51 | 1.10 |
| Greece | <5 years | 1.15 | 1.39 | 0.69 |
| Luxembourg | <5 years | 1.17 | 1.18 | 1.14 |
| Netherlands | <5 years | 1.17 | 1.19 | 1.16 |
| Singapore | <5 years | 1.17 | 1.53 | 0.95 |
| Cabo Verde | <5 years | 1.17 | 1.50 | 0.99 |
| Grenada | <5 years | 1.17 | 1.47 | 1.09 |
| Kazakhstan | <5 years | 1.18 | 1.20 | 1.15 |
| Czechia | <5 years | 1.19 | 1.25 | 1.13 |
| Iceland | <5 years | 1.19 | 1.16 | 1.44 |
| Guyana | <5 years | 1.20 | 1.38 | 1.09 |
| Turkiye | <5 years | 1.21 | 1.19 | 1.37 |
| Indonesia | <5 years | 1.21 | 1.21 | 1.19 |
| Trinidad and Tobago | <5 years | 1.21 | 1.45 | 1.11 |
| France | <5 years | 1.21 | 1.22 | 1.15 |
| Zimbabwe | <5 years | 1.21 | 1.24 | 1.02 |
| Barbados | <5 years | 1.22 | 3.57 | 1.09 |
| Jamaica | <5 years | 1.22 | 1.61 | 1.10 |
| American Samoa | <5 years | 1.23 | 1.28 | 1.16 |
| Finland | <5 years | 1.23 | 1.25 | 1.14 |
| Liberia | <5 years | 1.23 | 1.30 | 1.00 |
| Sweden | <5 years | 1.24 | 1.54 | 1.20 |
| Myanmar | <5 years | 1.25 | 1.27 | 1.23 |
| Switzerland | <5 years | 1.25 | 1.31 | 1.15 |
| Marshall Islands | <5 years | 1.26 | 1.28 | 1.15 |
| Pakistan | <5 years | 1.26 | 1.27 | 1.04 |
| Palestine | <5 years | 1.27 | 1.28 | 1.10 |
| Slovakia | <5 years | 1.27 | 1.28 | 1.15 |
| Philippines | <5 years | 1.27 | 1.37 | 1.16 |
| Tuvalu | <5 years | 1.28 | 1.27 | 1.63 |
| Puerto Rico | <5 years | 1.29 | 1.42 | 1.26 |
| Central African Republic | <5 years | 1.30 | 1.35 | 0.87 |
| Israel | <5 years | 1.30 | 1.35 | 1.15 |
| Croatia | <5 years | 1.31 | 1.23 | 1.48 |
| Angola | <5 years | 1.32 | 1.48 | 0.87 |
| Ireland | <5 years | 1.33 | 1.49 | 1.14 |
| Comoros | <5 years | 1.33 | 1.53 | 1.04 |
| Kyrgyzstan | <5 years | 1.34 | 1.52 | 1.14 |
| Slovenia | <5 years | 1.35 | 1.45 | 1.30 |
| Belgium | <5 years | 1.36 | 1.35 | 1.36 |
| Mauritius | <5 years | 1.36 | 2.01 | 1.15 |
| Portugal | <5 years | 1.38 | 1.54 | 0.89 |
| Ecuador | <5 years | 1.38 | 2.51 | 1.09 |
| Benin | <5 years | 1.38 | 1.46 | 1.00 |
| Bosnia and Herzegovina | <5 years | 1.39 | 1.70 | 1.16 |
| Serbia | <5 years | 1.40 | 1.60 | 1.12 |
| Northern Mariana Islands | <5 years | 1.41 | 1.50 | 1.15 |
| Germany | <5 years | 1.41 | 1.46 | 1.15 |
| Cambodia | <5 years | 1.41 | 1.56 | 1.09 |
| Libya | <5 years | 1.42 | 1.44 | 1.10 |
| Papua New Guinea | <5 years | 1.43 | 1.45 | 1.14 |
| Spain | <5 years | 1.46 | 1.49 | 1.30 |
| Togo | <5 years | 1.47 | 1.61 | 0.99 |
| Bermuda | <5 years | 1.48 | 1.77 | 1.26 |
| Montenegro | <5 years | 1.50 | 1.87 | 1.15 |
| Algeria | <5 years | 1.50 | 1.58 | 1.09 |
| Bulgaria | <5 years | 1.50 | 1.58 | 1.17 |
| Saint Lucia | <5 years | 1.50 | 5.20 | 1.09 |
| Malta | <5 years | 1.50 | 1.59 | 1.13 |
| Nauru | <5 years | 1.50 | 1.51 | 1.54 |
| Dominica | <5 years | 1.51 | 2.18 | 1.11 |
| C么te d'Ivoire | <5 years | 1.52 | 1.64 | 0.99 |
| Romania | <5 years | 1.52 | 1.68 | 1.17 |
| Gambia | <5 years | 1.52 | 1.68 | 1.00 |
| United Kingdom | <5 years | 1.54 | 1.80 | 1.17 |
| Guinea-Bissau | <5 years | 1.58 | 1.71 | 0.99 |
| Hungary | <5 years | 1.59 | 1.99 | 1.17 |
| Poland | <5 years | 1.59 | 2.25 | 1.04 |
| Bahrain | <5 years | 1.59 | 1.62 | 1.39 |
| Niue | <5 years | 1.59 | 1.62 | 1.11 |
| Cameroon | <5 years | 1.60 | 1.74 | 1.00 |
| Sierra Leone | <5 years | 1.60 | 1.68 | 1.00 |
| Antigua and Barbuda | <5 years | 1.60 | 4.34 | 1.08 |
| Yemen | <5 years | 1.65 | 1.68 | 1.09 |
| Burkina Faso | <5 years | 1.66 | 1.74 | 1.00 |
| Senegal | <5 years | 1.77 | 2.07 | 1.00 |
| Monaco | <5 years | 1.79 | 2.10 | 1.15 |
| Chad | <5 years | 1.80 | 1.86 | 0.99 |
| Mali | <5 years | 1.80 | 1.98 | 1.01 |
| Haiti | <5 years | 1.91 | 1.96 | 1.10 |
| Saint Kitts and Nevis | <5 years | 1.93 | 2.34 | 1.24 |
| Guinea | <5 years | 2.12 | 2.28 | 0.99 |
| Morocco | <5 years | 2.13 | 2.38 | 1.11 |
| Nigeria | <5 years | 2.16 | 2.27 | 1.02 |
| Bangladesh | <5 years | 2.19 | 2.32 | 1.19 |
| Tokelau | <5 years | 2.31 | 2.34 | 1.80 |
| New Zealand | <5 years | 2.34 | 3.83 | 0.95 |
| Albania | <5 years | 2.59 | 4.22 | 1.13 |
| Niger | <5 years | 2.67 | 2.87 | 0.99 |
| Andorra | <5 years | 2.80 | 4.54 | 1.14 |
| United States Virgin Islands | <5 years | 3.36 | 5.15 | 1.25 |
| San Marino | <5 years | 3.59 | 5.29 | 1.16 |
| Greenland | <5 years | 3.73 | 5.34 | 0.91 |
| United States Virgin Islands | 10-14 years | 0.20 | 0.01 | 1.30 |
| Qatar | 10-14 years | 0.33 | 0.22 | 1.34 |
| Malta | 10-14 years | 0.35 | 0.31 | 1.07 |
| Guam | 10-14 years | 0.54 | 0.03 | 1.14 |
| North Macedonia | 10-14 years | 0.66 | 0.60 | 1.06 |
| Greece | 10-14 years | 0.67 | 0.59 | 0.62 |
| Portugal | 10-14 years | 0.68 | 0.60 | 0.83 |
| Serbia | 10-14 years | 0.73 | 0.53 | 1.10 |
| Austria | 10-14 years | 0.78 | 0.78 | 0.76 |
| Viet Nam | 10-14 years | 0.79 | 0.76 | 1.07 |
| Romania | 10-14 years | 0.81 | 0.75 | 1.13 |
| Czechia | 10-14 years | 0.81 | 0.63 | 1.10 |
| Luxembourg | 10-14 years | 0.82 | 0.41 | 1.13 |
| Bermuda | 10-14 years | 0.83 | 0.68 | 1.26 |
| Egypt | 10-14 years | 0.84 | 0.77 | 1.03 |
| Eswatini | 10-14 years | 0.85 | 0.64 | 1.07 |
| Denmark | 10-14 years | 0.85 | 0.80 | 1.12 |
| Nepal | 10-14 years | 0.87 | 0.84 | 0.99 |
| Kuwait | 10-14 years | 0.87 | 0.83 | 1.41 |
| Bhutan | 10-14 years | 0.88 | 0.81 | 1.08 |
| Jordan | 10-14 years | 0.89 | 0.84 | 1.40 |
| Grenada | 10-14 years | 0.89 | 0.55 | 1.14 |
| Namibia | 10-14 years | 0.90 | 0.65 | 1.07 |
| Lesotho | 10-14 years | 0.90 | 0.76 | 1.08 |
| Colombia | 10-14 years | 0.91 | 0.81 | 1.36 |
| Gabon | 10-14 years | 0.91 | 0.99 | 0.90 |
| Mozambique | 10-14 years | 0.91 | 0.44 | 1.07 |
| Bulgaria | 10-14 years | 0.92 | 0.87 | 1.14 |
| Norway | 10-14 years | 0.93 | 1.28 | 0.79 |
| Botswana | 10-14 years | 0.93 | 0.71 | 1.07 |
| Greenland | 10-14 years | 0.94 | 0.83 | 1.01 |
| Congo | 10-14 years | 0.95 | 1.34 | 0.90 |
| Venezuela (Bolivarian Republic of) | 10-14 years | 0.95 | 0.89 | 1.35 |
| Canada | 10-14 years | 0.96 | 0.91 | 0.99 |
| Cabo Verde | 10-14 years | 0.97 | 0.70 | 1.02 |
| France | 10-14 years | 0.97 | 0.89 | 1.14 |
| Angola | 10-14 years | 0.98 | 1.41 | 0.89 |
| Central African Republic | 10-14 years | 0.98 | 1.15 | 0.91 |
| Jamaica | 10-14 years | 0.99 | 0.65 | 1.14 |
| Zimbabwe | 10-14 years | 0.99 | 0.96 | 1.08 |
| Equatorial Guinea | 10-14 years | 0.99 | 1.83 | 0.91 |
| Ghana | 10-14 years | 1.00 | 0.85 | 1.02 |
| Democratic Republic of the Congo | 10-14 years | 1.00 | 1.60 | 0.91 |
| Timor-Leste | 10-14 years | 1.00 | 0.96 | 1.16 |
| Netherlands | 10-14 years | 1.01 | 0.94 | 1.12 |
| Benin | 10-14 years | 1.02 | 1.00 | 1.03 |
| China | 10-14 years | 1.02 | 0.85 | 1.05 |
| Libya | 10-14 years | 1.02 | 0.99 | 1.07 |
| United States of America | 10-14 years | 1.03 | 1.04 | 0.97 |
| Israel | 10-14 years | 1.03 | 1.01 | 1.10 |
| Japan | 10-14 years | 1.03 | 1.14 | 0.90 |
| Madagascar | 10-14 years | 1.03 | 0.97 | 1.09 |
| Uzbekistan | 10-14 years | 1.04 | 0.91 | 1.15 |
| Syrian Arab Republic | 10-14 years | 1.04 | 1.04 | 1.06 |
| Mongolia | 10-14 years | 1.05 | 0.86 | 1.15 |
| Saint Kitts and Nevis | 10-14 years | 1.06 | 1.04 | 1.24 |
| Cuba | 10-14 years | 1.06 | 0.77 | 1.15 |
| Maldives | 10-14 years | 1.06 | 0.91 | 1.17 |
| United Arab Emirates | 10-14 years | 1.07 | 1.08 | 1.07 |
| C么te d'Ivoire | 10-14 years | 1.07 | 1.18 | 1.02 |
| Guyana | 10-14 years | 1.07 | 0.94 | 1.15 |
| Guatemala | 10-14 years | 1.07 | 0.68 | 1.10 |
| United Republic of Tanzania | 10-14 years | 1.07 | 0.96 | 1.10 |
| Malawi | 10-14 years | 1.07 | 0.94 | 1.11 |
| Lao People's Democratic Republic | 10-14 years | 1.07 | 1.06 | 1.13 |
| Sao Tome and Principe | 10-14 years | 1.07 | 1.13 | 1.05 |
| United Kingdom | 10-14 years | 1.08 | 1.00 | 1.13 |
| Ukraine | 10-14 years | 1.08 | 1.07 | 1.07 |
| Georgia | 10-14 years | 1.08 | 0.84 | 1.13 |
| Malaysia | 10-14 years | 1.08 | 0.96 | 1.17 |
| Iraq | 10-14 years | 1.08 | 1.11 | 1.06 |
| Democratic People's Republic of Korea | 10-14 years | 1.09 | 1.12 | 1.07 |
| Nigeria | 10-14 years | 1.09 | 1.16 | 1.06 |
| Ireland | 10-14 years | 1.09 | 1.07 | 1.12 |
| Gambia | 10-14 years | 1.09 | 1.23 | 1.03 |
| Finland | 10-14 years | 1.09 | 1.09 | 1.09 |
| Zambia | 10-14 years | 1.09 | 1.03 | 1.10 |
| Costa Rica | 10-14 years | 1.09 | 0.68 | 1.13 |
| Djibouti | 10-14 years | 1.10 | 1.10 | 1.10 |
| Uganda | 10-14 years | 1.10 | 1.03 | 1.10 |
| Kenya | 10-14 years | 1.10 | 0.96 | 1.10 |
| South Africa | 10-14 years | 1.10 | 1.16 | 1.08 |
| Cambodia | 10-14 years | 1.10 | 1.10 | 1.09 |
| Mexico | 10-14 years | 1.10 | 0.87 | 1.15 |
| Albania | 10-14 years | 1.10 | 0.69 | 1.14 |
| Guinea-Bissau | 10-14 years | 1.10 | 1.19 | 1.04 |
| Afghanistan | 10-14 years | 1.10 | 1.14 | 1.05 |
| Cameroon | 10-14 years | 1.10 | 1.36 | 1.02 |
| Honduras | 10-14 years | 1.11 | 1.22 | 1.11 |
| Togo | 10-14 years | 1.11 | 1.29 | 1.04 |
| Paraguay | 10-14 years | 1.11 | 1.03 | 1.14 |
| Dominican Republic | 10-14 years | 1.11 | 1.05 | 1.16 |
| Iran (Islamic Republic of) | 10-14 years | 1.11 | 1.19 | 1.07 |
| Panama | 10-14 years | 1.11 | 0.84 | 1.14 |
| Germany | 10-14 years | 1.11 | 1.11 | 1.12 |
| Somalia | 10-14 years | 1.12 | 1.14 | 1.10 |
| Belize | 10-14 years | 1.12 | 0.95 | 1.16 |
| Myanmar | 10-14 years | 1.13 | 1.09 | 1.26 |
| Mauritania | 10-14 years | 1.13 | 1.58 | 1.03 |
| Tajikistan | 10-14 years | 1.13 | 1.11 | 1.15 |
| Argentina | 10-14 years | 1.13 | 1.29 | 1.13 |
| Burkina Faso | 10-14 years | 1.13 | 1.30 | 1.04 |
| Turkmenistan | 10-14 years | 1.13 | 1.10 | 1.15 |
| Bahamas | 10-14 years | 1.13 | 0.99 | 1.15 |
| Peru | 10-14 years | 1.14 | 1.23 | 1.13 |
| Thailand | 10-14 years | 1.14 | 1.09 | 1.16 |
| Senegal | 10-14 years | 1.14 | 1.47 | 1.02 |
| Eritrea | 10-14 years | 1.14 | 1.26 | 1.10 |
| El Salvador | 10-14 years | 1.14 | 1.46 | 1.12 |
| Nicaragua | 10-14 years | 1.14 | 1.54 | 1.12 |
| Bolivia (Plurinational State of) | 10-14 years | 1.14 | 1.23 | 1.13 |
| Montenegro | 10-14 years | 1.15 | 1.11 | 1.13 |
| Rwanda | 10-14 years | 1.15 | 1.43 | 1.10 |
| Puerto Rico | 10-14 years | 1.16 | 1.10 | 1.29 |
| Comoros | 10-14 years | 1.16 | 1.47 | 1.09 |
| Azerbaijan | 10-14 years | 1.16 | 1.22 | 1.15 |
| Lebanon | 10-14 years | 1.17 | 1.15 | 1.35 |
| Tunisia | 10-14 years | 1.17 | 1.14 | 1.37 |
| Indonesia | 10-14 years | 1.17 | 1.17 | 1.21 |
| Ecuador | 10-14 years | 1.18 | 1.47 | 1.13 |
| Saint Lucia | 10-14 years | 1.18 | 1.24 | 1.14 |
| Armenia | 10-14 years | 1.18 | 1.43 | 1.15 |
| Switzerland | 10-14 years | 1.18 | 1.25 | 1.13 |
| Sierra Leone | 10-14 years | 1.18 | 1.42 | 1.04 |
| South Sudan | 10-14 years | 1.18 | 1.31 | 1.11 |
| Brazil | 10-14 years | 1.18 | 1.11 | 1.20 |
| Trinidad and Tobago | 10-14 years | 1.19 | 1.32 | 1.15 |
| Brunei Darussalam | 10-14 years | 1.19 | 1.22 | 1.01 |
| Dominica | 10-14 years | 1.20 | 1.33 | 1.14 |
| Burundi | 10-14 years | 1.20 | 1.57 | 1.09 |
| Russian Federation | 10-14 years | 1.20 | 1.34 | 1.07 |
| Liberia | 10-14 years | 1.20 | 1.65 | 1.03 |
| Barbados | 10-14 years | 1.20 | 2.40 | 1.11 |
| Republic of Korea | 10-14 years | 1.21 | 1.33 | 0.96 |
| Uruguay | 10-14 years | 1.21 | 1.35 | 1.11 |
| Philippines | 10-14 years | 1.21 | 1.22 | 1.19 |
| Sudan | 10-14 years | 1.22 | 1.27 | 1.08 |
| Ethiopia | 10-14 years | 1.22 | 1.53 | 1.15 |
| Kyrgyzstan | 10-14 years | 1.22 | 1.50 | 1.15 |
| Guinea | 10-14 years | 1.22 | 1.51 | 1.03 |
| Algeria | 10-14 years | 1.23 | 1.50 | 1.04 |
| Chad | 10-14 years | 1.23 | 1.50 | 1.02 |
| Chile | 10-14 years | 1.23 | 1.24 | 1.14 |
| Slovakia | 10-14 years | 1.23 | 1.17 | 1.11 |
| Mauritius | 10-14 years | 1.23 | 1.38 | 1.14 |
| Australia | 10-14 years | 1.24 | 1.29 | 1.25 |
| Antigua and Barbuda | 10-14 years | 1.27 | 2.53 | 1.13 |
| India | 10-14 years | 1.27 | 1.33 | 1.19 |
| Saint Vincent and the Grenadines | 10-14 years | 1.28 | 1.59 | 1.12 |
| Mali | 10-14 years | 1.29 | 2.03 | 1.02 |
| Palestine | 10-14 years | 1.29 | 1.54 | 1.06 |
| Kazakhstan | 10-14 years | 1.30 | 1.33 | 1.17 |
| Sri Lanka | 10-14 years | 1.31 | 1.32 | 1.20 |
| Monaco | 10-14 years | 1.33 | 1.48 | 1.10 |
| Bosnia and Herzegovina | 10-14 years | 1.35 | 1.43 | 1.12 |
| Seychelles | 10-14 years | 1.35 | 7.90 | 1.16 |
| Taiwan (Province of China) | 10-14 years | 1.35 | 1.40 | 1.30 |
| Niger | 10-14 years | 1.37 | 2.21 | 1.02 |
| Morocco | 10-14 years | 1.40 | 1.98 | 1.07 |
| Pakistan | 10-14 years | 1.40 | 1.50 | 1.06 |
| Singapore | 10-14 years | 1.41 | 1.71 | 0.97 |
| San Marino | 10-14 years | 1.42 | 1.60 | 1.14 |
| Belgium | 10-14 years | 1.42 | 1.41 | 1.38 |
| Spain | 10-14 years | 1.42 | 1.45 | 1.26 |
| Lithuania | 10-14 years | 1.43 | 1.56 | 1.01 |
| Belarus | 10-14 years | 1.44 | 1.70 | 1.03 |
| Estonia | 10-14 years | 1.48 | 2.21 | 1.00 |
| Yemen | 10-14 years | 1.54 | 1.96 | 1.05 |
| Kiribati | 10-14 years | 1.57 | 1.70 | 1.18 |
| Turkiye | 10-14 years | 1.59 | 1.60 | 1.44 |
| Republic of Moldova | 10-14 years | 1.62 | 1.78 | 1.03 |
| Tonga | 10-14 years | 1.64 | 3.02 | 1.16 |
| Haiti | 10-14 years | 1.65 | 1.90 | 1.12 |
| Vanuatu | 10-14 years | 1.66 | 1.85 | 1.18 |
| Suriname | 10-14 years | 1.72 | 4.16 | 1.13 |
| Fiji | 10-14 years | 1.76 | 1.90 | 1.19 |
| Palau | 10-14 years | 1.76 | 1.80 | 1.26 |
| Hungary | 10-14 years | 1.79 | 2.30 | 1.13 |
| Bangladesh | 10-14 years | 1.80 | 2.00 | 1.23 |
| Poland | 10-14 years | 1.86 | 2.37 | 1.07 |
| Northern Mariana Islands | 10-14 years | 1.87 | 4.41 | 1.17 |
| Italy | 10-14 years | 1.88 | 2.19 | 1.15 |
| Croatia | 10-14 years | 1.98 | 2.39 | 1.38 |
| Samoa | 10-14 years | 2.03 | 2.86 | 1.18 |
| Saudi Arabia | 10-14 years | 2.07 | 2.19 | 1.52 |
| Latvia | 10-14 years | 2.07 | 5.72 | 1.02 |
| Solomon Islands | 10-14 years | 2.07 | 2.64 | 1.18 |
| Micronesia (Federated States of) | 10-14 years | 2.10 | 2.70 | 1.18 |
| Bahrain | 10-14 years | 2.12 | 2.23 | 1.47 |
| American Samoa | 10-14 years | 2.12 | 3.59 | 1.18 |
| Cook Islands | 10-14 years | 2.13 | 2.25 | 1.34 |
| Tuvalu | 10-14 years | 2.13 | 2.16 | 1.47 |
| Andorra | 10-14 years | 2.21 | 3.57 | 1.13 |
| Cyprus | 10-14 years | 2.23 | 2.54 | 1.16 |
| Oman | 10-14 years | 2.36 | 2.49 | 1.42 |
| Marshall Islands | 10-14 years | 2.37 | 3.00 | 1.17 |
| Nauru | 10-14 years | 2.49 | 2.52 | 1.44 |
| Papua New Guinea | 10-14 years | 2.52 | 2.87 | 1.17 |
| Sweden | 10-14 years | 2.88 | 5.80 | 1.15 |
| New Zealand | 10-14 years | 3.00 | 4.81 | 1.31 |
| Iceland | 10-14 years | 3.15 | 4.19 | 1.45 |
| Slovenia | 10-14 years | 3.23 | 12.00 | 1.24 |
| Niue | 10-14 years | 3.58 | 3.62 | 1.19 |
| Tokelau | 10-14 years | 3.65 | 3.68 | 1.61 |
| United States Virgin Islands | 15-19 years | 0.05 | 0.01 | 1.33 |
| Iceland | 15-19 years | 0.22 | 0.03 | 1.41 |
| Cyprus | 15-19 years | 0.44 | 0.11 | 1.16 |
| Cook Islands | 15-19 years | 0.45 | 0.40 | 1.31 |
| Puerto Rico | 15-19 years | 0.54 | 0.47 | 1.31 |
| Bermuda | 15-19 years | 0.55 | 0.50 | 1.26 |
| Qatar | 15-19 years | 0.59 | 0.53 | 1.35 |
| Denmark | 15-19 years | 0.60 | 0.53 | 1.13 |
| Montenegro | 15-19 years | 0.66 | 0.62 | 1.12 |
| Greece | 15-19 years | 0.66 | 0.64 | 0.61 |
| Nepal | 15-19 years | 0.67 | 0.62 | 1.06 |
| Guam | 15-19 years | 0.69 | 0.44 | 1.16 |
| Czechia | 15-19 years | 0.70 | 0.61 | 1.09 |
| Bhutan | 15-19 years | 0.70 | 0.61 | 1.17 |
| Viet Nam | 15-19 years | 0.70 | 0.68 | 1.10 |
| Bulgaria | 15-19 years | 0.70 | 0.68 | 1.13 |
| Venezuela (Bolivarian Republic of) | 15-19 years | 0.72 | 0.64 | 1.45 |
| Australia | 15-19 years | 0.72 | 0.64 | 1.29 |
| Slovenia | 15-19 years | 0.72 | 0.61 | 1.22 |
| United Arab Emirates | 15-19 years | 0.74 | 0.37 | 1.07 |
| Kiribati | 15-19 years | 0.74 | 0.68 | 1.20 |
| Norway | 15-19 years | 0.74 | 0.67 | 0.80 |
| North Macedonia | 15-19 years | 0.75 | 0.72 | 1.05 |
| Finland | 15-19 years | 0.76 | 0.73 | 1.10 |
| Israel | 15-19 years | 0.76 | 0.70 | 1.10 |
| Solomon Islands | 15-19 years | 0.78 | 0.67 | 1.21 |
| Ukraine | 15-19 years | 0.79 | 0.70 | 1.06 |
| France | 15-19 years | 0.79 | 0.71 | 1.12 |
| Vanuatu | 15-19 years | 0.80 | 0.75 | 1.20 |
| Malta | 15-19 years | 0.81 | 0.75 | 1.07 |
| Germany | 15-19 years | 0.81 | 0.78 | 1.11 |
| Poland | 15-19 years | 0.82 | 0.74 | 1.09 |
| Zimbabwe | 15-19 years | 0.83 | 0.77 | 1.12 |
| Portugal | 15-19 years | 0.83 | 0.82 | 0.83 |
| Uruguay | 15-19 years | 0.83 | 0.74 | 1.17 |
| Romania | 15-19 years | 0.84 | 0.80 | 1.12 |
| Lithuania | 15-19 years | 0.84 | 0.72 | 1.01 |
| Hungary | 15-19 years | 0.84 | 0.77 | 1.11 |
| Chad | 15-19 years | 0.85 | 0.64 | 1.06 |
| Colombia | 15-19 years | 0.85 | 0.70 | 1.44 |
| Cabo Verde | 15-19 years | 0.85 | 0.34 | 1.05 |
| Guinea-Bissau | 15-19 years | 0.87 | 0.69 | 1.08 |
| Gambia | 15-19 years | 0.88 | 0.63 | 1.07 |
| Luxembourg | 15-19 years | 0.88 | 0.79 | 1.13 |
| Mozambique | 15-19 years | 0.89 | 0.38 | 1.11 |
| Saint Kitts and Nevis | 15-19 years | 0.89 | 0.87 | 1.25 |
| Eswatini | 15-19 years | 0.90 | 0.73 | 1.12 |
| Croatia | 15-19 years | 0.91 | 0.85 | 1.38 |
| India | 15-19 years | 0.92 | 0.84 | 1.28 |
| Namibia | 15-19 years | 0.92 | 0.66 | 1.10 |
| Uzbekistan | 15-19 years | 0.92 | 0.72 | 1.15 |
| Tuvalu | 15-19 years | 0.92 | 0.91 | 1.41 |
| Tunisia | 15-19 years | 0.92 | 0.87 | 1.40 |
| Belgium | 15-19 years | 0.93 | 0.83 | 1.36 |
| Central African Republic | 15-19 years | 0.93 | 0.84 | 0.99 |
| Gabon | 15-19 years | 0.94 | 0.78 | 0.98 |
| Georgia | 15-19 years | 0.94 | 0.67 | 1.13 |
| Ghana | 15-19 years | 0.94 | 0.58 | 1.05 |
| Sierra Leone | 15-19 years | 0.95 | 0.78 | 1.07 |
| Tonga | 15-19 years | 0.95 | 0.75 | 1.17 |
| C么te d'Ivoire | 15-19 years | 0.96 | 0.79 | 1.06 |
| Chile | 15-19 years | 0.96 | 0.88 | 1.19 |
| Myanmar | 15-19 years | 0.96 | 0.85 | 1.34 |
| Senegal | 15-19 years | 0.96 | 0.77 | 1.06 |
| Nauru | 15-19 years | 0.96 | 0.96 | 1.37 |
| Benin | 15-19 years | 0.96 | 0.72 | 1.07 |
| Egypt | 15-19 years | 0.97 | 0.94 | 1.02 |
| Malaysia | 15-19 years | 0.97 | 0.68 | 1.23 |
| Madagascar | 15-19 years | 0.97 | 0.76 | 1.14 |
| Slovakia | 15-19 years | 0.97 | 0.91 | 1.09 |
| Togo | 15-19 years | 0.97 | 0.82 | 1.07 |
| Thailand | 15-19 years | 0.98 | 0.63 | 1.23 |
| Equatorial Guinea | 15-19 years | 0.98 | 1.03 | 0.98 |
| Saint Vincent and the Grenadines | 15-19 years | 0.98 | 0.79 | 1.17 |
| Taiwan (Province of China) | 15-19 years | 0.98 | 0.85 | 1.34 |
| Guinea | 15-19 years | 0.98 | 0.88 | 1.07 |
| Austria | 15-19 years | 0.98 | 0.99 | 0.80 |
| South Africa | 15-19 years | 0.99 | 0.64 | 1.12 |
| Lesotho | 15-19 years | 0.99 | 0.90 | 1.12 |
| Nigeria | 15-19 years | 0.99 | 0.75 | 1.09 |
| Congo | 15-19 years | 1.00 | 1.10 | 0.98 |
| Botswana | 15-19 years | 1.00 | 0.82 | 1.10 |
| Cameroon | 15-19 years | 1.00 | 0.90 | 1.06 |
| Sao Tome and Principe | 15-19 years | 1.00 | 0.91 | 1.08 |
| China | 15-19 years | 1.01 | 0.77 | 1.08 |
| Angola | 15-19 years | 1.01 | 1.16 | 0.97 |
| Democratic Republic of the Congo | 15-19 years | 1.01 | 1.14 | 0.98 |
| Burkina Faso | 15-19 years | 1.01 | 0.95 | 1.07 |
| Democratic People's Republic of Korea | 15-19 years | 1.02 | 0.91 | 1.09 |
| Netherlands | 15-19 years | 1.02 | 0.93 | 1.12 |
| United States of America | 15-19 years | 1.03 | 0.97 | 1.11 |
| Liberia | 15-19 years | 1.03 | 1.02 | 1.06 |
| Mauritania | 15-19 years | 1.04 | 0.95 | 1.07 |
| Lebanon | 15-19 years | 1.04 | 1.02 | 1.36 |
| Syrian Arab Republic | 15-19 years | 1.05 | 1.05 | 1.05 |
| Iraq | 15-19 years | 1.05 | 1.06 | 1.05 |
| Canada | 15-19 years | 1.05 | 0.98 | 1.10 |
| Mongolia | 15-19 years | 1.06 | 0.93 | 1.16 |
| Iran (Islamic Republic of) | 15-19 years | 1.06 | 1.09 | 1.06 |
| Malawi | 15-19 years | 1.06 | 0.68 | 1.16 |
| Libya | 15-19 years | 1.07 | 1.07 | 1.07 |
| Sri Lanka | 15-19 years | 1.07 | 1.06 | 1.21 |
| Micronesia (Federated States of) | 15-19 years | 1.07 | 1.05 | 1.20 |
| Eritrea | 15-19 years | 1.07 | 0.84 | 1.14 |
| Uganda | 15-19 years | 1.08 | 0.77 | 1.14 |
| Azerbaijan | 15-19 years | 1.08 | 0.94 | 1.15 |
| Japan | 15-19 years | 1.08 | 1.10 | 0.91 |
| Somalia | 15-19 years | 1.08 | 0.91 | 1.15 |
| Kenya | 15-19 years | 1.08 | 0.71 | 1.14 |
| Albania | 15-19 years | 1.08 | 0.59 | 1.14 |
| United Kingdom | 15-19 years | 1.08 | 1.01 | 1.12 |
| United Republic of Tanzania | 15-19 years | 1.08 | 0.80 | 1.14 |
| Serbia | 15-19 years | 1.09 | 1.05 | 1.07 |
| Djibouti | 15-19 years | 1.09 | 0.70 | 1.16 |
| Niger | 15-19 years | 1.10 | 1.18 | 1.06 |
| Cuba | 15-19 years | 1.10 | 0.92 | 1.18 |
| Suriname | 15-19 years | 1.10 | 1.02 | 1.16 |
| Switzerland | 15-19 years | 1.10 | 1.08 | 1.13 |
| Trinidad and Tobago | 15-19 years | 1.10 | 0.99 | 1.18 |
| Timor-Leste | 15-19 years | 1.10 | 1.07 | 1.23 |
| Russian Federation | 15-19 years | 1.11 | 1.09 | 1.09 |
| Marshall Islands | 15-19 years | 1.11 | 1.09 | 1.19 |
| Argentina | 15-19 years | 1.11 | 0.91 | 1.14 |
| Tajikistan | 15-19 years | 1.11 | 1.05 | 1.16 |
| Bosnia and Herzegovina | 15-19 years | 1.11 | 1.06 | 1.10 |
| Armenia | 15-19 years | 1.12 | 1.03 | 1.16 |
| South Sudan | 15-19 years | 1.12 | 1.06 | 1.15 |
| Paraguay | 15-19 years | 1.12 | 0.98 | 1.18 |
| Brunei Darussalam | 15-19 years | 1.12 | 1.13 | 1.03 |
| Burundi | 15-19 years | 1.13 | 1.10 | 1.14 |
| Brazil | 15-19 years | 1.13 | 0.75 | 1.24 |
| Zambia | 15-19 years | 1.13 | 1.11 | 1.14 |
| Dominican Republic | 15-19 years | 1.13 | 1.05 | 1.20 |
| Samoa | 15-19 years | 1.14 | 1.12 | 1.20 |
| Saint Lucia | 15-19 years | 1.14 | 1.00 | 1.20 |
| Belize | 15-19 years | 1.14 | 0.95 | 1.20 |
| Yemen | 15-19 years | 1.14 | 1.31 | 1.05 |
| Rwanda | 15-19 years | 1.14 | 1.08 | 1.15 |
| Ecuador | 15-19 years | 1.14 | 0.87 | 1.19 |
| Philippines | 15-19 years | 1.15 | 1.10 | 1.24 |
| Algeria | 15-19 years | 1.15 | 1.38 | 1.04 |
| Morocco | 15-19 years | 1.15 | 1.30 | 1.06 |
| Fiji | 15-19 years | 1.15 | 1.14 | 1.19 |
| Guyana | 15-19 years | 1.16 | 1.10 | 1.19 |
| Turkmenistan | 15-19 years | 1.16 | 1.19 | 1.14 |
| Comoros | 15-19 years | 1.16 | 1.38 | 1.14 |
| Peru | 15-19 years | 1.17 | 1.11 | 1.18 |
| Grenada | 15-19 years | 1.17 | 1.17 | 1.18 |
| Panama | 15-19 years | 1.17 | 0.81 | 1.22 |
| Maldives | 15-19 years | 1.17 | 1.09 | 1.24 |
| Bahamas | 15-19 years | 1.17 | 1.15 | 1.19 |
| Guatemala | 15-19 years | 1.18 | 0.81 | 1.20 |
| El Salvador | 15-19 years | 1.18 | 0.94 | 1.21 |
| Ethiopia | 15-19 years | 1.18 | 1.14 | 1.19 |
| Costa Rica | 15-19 years | 1.18 | 0.88 | 1.21 |
| Mexico | 15-19 years | 1.18 | 0.95 | 1.23 |
| Mali | 15-19 years | 1.18 | 1.52 | 1.05 |
| Nicaragua | 15-19 years | 1.19 | 1.19 | 1.20 |
| Seychelles | 15-19 years | 1.19 | 0.45 | 1.21 |
| Palestine | 15-19 years | 1.19 | 1.45 | 1.06 |
| Cambodia | 15-19 years | 1.20 | 1.23 | 1.15 |
| Jamaica | 15-19 years | 1.20 | 1.26 | 1.19 |
| Belarus | 15-19 years | 1.21 | 1.24 | 1.02 |
| Bolivia (Plurinational State of) | 15-19 years | 1.21 | 1.33 | 1.18 |
| Indonesia | 15-19 years | 1.21 | 1.20 | 1.27 |
| Republic of Moldova | 15-19 years | 1.22 | 1.23 | 1.03 |
| Honduras | 15-19 years | 1.22 | 1.96 | 1.20 |
| Barbados | 15-19 years | 1.22 | 1.63 | 1.16 |
| Northern Mariana Islands | 15-19 years | 1.23 | 1.26 | 1.19 |
| Sudan | 15-19 years | 1.23 | 1.37 | 1.08 |
| San Marino | 15-19 years | 1.23 | 1.23 | 1.15 |
| Antigua and Barbuda | 15-19 years | 1.24 | 1.47 | 1.18 |
| Jordan | 15-19 years | 1.24 | 1.23 | 1.42 |
| Turkiye | 15-19 years | 1.24 | 1.21 | 1.45 |
| Italy | 15-19 years | 1.25 | 1.25 | 1.15 |
| American Samoa | 15-19 years | 1.27 | 1.30 | 1.20 |
| Kuwait | 15-19 years | 1.27 | 1.24 | 1.43 |
| Dominica | 15-19 years | 1.27 | 1.43 | 1.19 |
| Kyrgyzstan | 15-19 years | 1.27 | 1.55 | 1.16 |
| Monaco | 15-19 years | 1.28 | 1.31 | 1.10 |
| Kazakhstan | 15-19 years | 1.29 | 1.30 | 1.18 |
| Republic of Korea | 15-19 years | 1.31 | 1.39 | 0.98 |
| Afghanistan | 15-19 years | 1.31 | 1.47 | 1.04 |
| Lao People's Democratic Republic | 15-19 years | 1.31 | 1.37 | 1.20 |
| Mauritius | 15-19 years | 1.32 | 1.51 | 1.18 |
| New Zealand | 15-19 years | 1.33 | 1.32 | 1.38 |
| Spain | 15-19 years | 1.35 | 1.35 | 1.21 |
| Latvia | 15-19 years | 1.37 | 1.43 | 1.02 |
| Pakistan | 15-19 years | 1.38 | 1.44 | 1.14 |
| Papua New Guinea | 15-19 years | 1.41 | 1.45 | 1.19 |
| Bangladesh | 15-19 years | 1.45 | 1.49 | 1.33 |
| Ireland | 15-19 years | 1.47 | 1.49 | 1.13 |
| Tokelau | 15-19 years | 1.75 | 1.76 | 1.58 |
| Haiti | 15-19 years | 1.80 | 2.23 | 1.16 |
| Bahrain | 15-19 years | 1.87 | 1.90 | 1.49 |
| Niue | 15-19 years | 1.95 | 1.96 | 1.20 |
| Sweden | 15-19 years | 2.01 | 2.25 | 1.16 |
| Saudi Arabia | 15-19 years | 2.08 | 2.16 | 1.54 |
| Oman | 15-19 years | 2.22 | 2.29 | 1.45 |
| Greenland | 15-19 years | 2.28 | 3.59 | 1.12 |
| Singapore | 15-19 years | 2.30 | 2.65 | 0.99 |
| Palau | 15-19 years | 2.36 | 2.39 | 1.22 |
| Andorra | 15-19 years | 2.36 | 2.96 | 1.14 |
| Estonia | 15-19 years | 2.38 | 3.08 | 0.99 |
| Cook Islands | 20-24 years | 0.28 | 0.26 | 1.36 |
| Cyprus | 20-24 years | 0.34 | 0.30 | 1.20 |
| Norway | 20-24 years | 0.43 | 0.27 | 0.83 |
| United States Virgin Islands | 20-24 years | 0.44 | 0.42 | 1.41 |
| Greece | 20-24 years | 0.47 | 0.44 | 0.62 |
| Guam | 20-24 years | 0.48 | 0.15 | 1.20 |
| Montenegro | 20-24 years | 0.51 | 0.47 | 1.08 |
| Malta | 20-24 years | 0.51 | 0.46 | 1.11 |
| Kiribati | 20-24 years | 0.52 | 0.45 | 1.23 |
| Bulgaria | 20-24 years | 0.53 | 0.51 | 1.13 |
| Denmark | 20-24 years | 0.56 | 0.51 | 1.17 |
| Vanuatu | 20-24 years | 0.57 | 0.50 | 1.24 |
| Bermuda | 20-24 years | 0.59 | 0.53 | 1.30 |
| Austria | 20-24 years | 0.60 | 0.56 | 0.94 |
| France | 20-24 years | 0.61 | 0.55 | 1.14 |
| Singapore | 20-24 years | 0.63 | 0.53 | 1.02 |
| Solomon Islands | 20-24 years | 0.65 | 0.54 | 1.24 |
| Qatar | 20-24 years | 0.69 | 0.61 | 1.38 |
| Nepal | 20-24 years | 0.69 | 0.61 | 1.14 |
| Romania | 20-24 years | 0.69 | 0.65 | 1.10 |
| Japan | 20-24 years | 0.70 | 0.64 | 0.94 |
| Switzerland | 20-24 years | 0.71 | 0.60 | 1.16 |
| Bhutan | 20-24 years | 0.71 | 0.59 | 1.29 |
| Fiji | 20-24 years | 0.72 | 0.67 | 1.21 |
| Israel | 20-24 years | 0.74 | 0.69 | 1.12 |
| Finland | 20-24 years | 0.75 | 0.72 | 1.15 |
| Viet Nam | 20-24 years | 0.75 | 0.73 | 1.23 |
| Ukraine | 20-24 years | 0.79 | 0.74 | 1.06 |
| Tuvalu | 20-24 years | 0.79 | 0.79 | 1.58 |
| Latvia | 20-24 years | 0.80 | 0.74 | 1.02 |
| Czechia | 20-24 years | 0.80 | 0.77 | 1.07 |
| Iceland | 20-24 years | 0.81 | 0.72 | 1.41 |
| Ireland | 20-24 years | 0.82 | 0.70 | 1.16 |
| Cabo Verde | 20-24 years | 0.82 | 0.35 | 1.06 |
| Portugal | 20-24 years | 0.83 | 0.82 | 0.84 |
| Luxembourg | 20-24 years | 0.83 | 0.73 | 1.14 |
| Venezuela (Bolivarian Republic of) | 20-24 years | 0.84 | 0.76 | 1.62 |
| Gambia | 20-24 years | 0.84 | 0.59 | 1.09 |
| United Kingdom | 20-24 years | 0.85 | 0.76 | 1.13 |
| Tonga | 20-24 years | 0.85 | 0.58 | 1.20 |
| Slovenia | 20-24 years | 0.85 | 0.78 | 1.22 |
| San Marino | 20-24 years | 0.86 | 0.79 | 1.16 |
| United Arab Emirates | 20-24 years | 0.86 | 0.62 | 1.08 |
| Australia | 20-24 years | 0.86 | 0.79 | 1.40 |
| United States of America | 20-24 years | 0.86 | 0.78 | 1.34 |
| Croatia | 20-24 years | 0.87 | 0.83 | 1.37 |
| Uruguay | 20-24 years | 0.87 | 0.78 | 1.28 |
| Republic of Moldova | 20-24 years | 0.87 | 0.83 | 1.02 |
| Italy | 20-24 years | 0.88 | 0.84 | 1.17 |
| Monaco | 20-24 years | 0.88 | 0.85 | 1.10 |
| Germany | 20-24 years | 0.88 | 0.85 | 1.13 |
| Brunei Darussalam | 20-24 years | 0.88 | 0.85 | 1.06 |
| Tunisia | 20-24 years | 0.88 | 0.84 | 1.45 |
| Puerto Rico | 20-24 years | 0.88 | 0.84 | 1.38 |
| Micronesia (Federated States of) | 20-24 years | 0.88 | 0.83 | 1.23 |
| Nauru | 20-24 years | 0.88 | 0.88 | 1.57 |
| Bangladesh | 20-24 years | 0.89 | 0.78 | 1.48 |
| North Macedonia | 20-24 years | 0.89 | 0.86 | 1.03 |
| Belgium | 20-24 years | 0.89 | 0.82 | 1.39 |
| Chad | 20-24 years | 0.89 | 0.75 | 1.09 |
| Ghana | 20-24 years | 0.89 | 0.53 | 1.08 |
| Belarus | 20-24 years | 0.89 | 0.85 | 1.01 |
| Hungary | 20-24 years | 0.90 | 0.86 | 1.08 |
| India | 20-24 years | 0.90 | 0.80 | 1.37 |
| Guinea-Bissau | 20-24 years | 0.91 | 0.80 | 1.08 |
| Benin | 20-24 years | 0.91 | 0.64 | 1.09 |
| Poland | 20-24 years | 0.91 | 0.86 | 1.11 |
| Egypt | 20-24 years | 0.91 | 0.85 | 1.04 |
| Senegal | 20-24 years | 0.93 | 0.74 | 1.08 |
| Samoa | 20-24 years | 0.93 | 0.86 | 1.22 |
| Gabon | 20-24 years | 0.93 | 0.68 | 0.99 |
| Saint Kitts and Nevis | 20-24 years | 0.93 | 0.90 | 1.31 |
| Bosnia and Herzegovina | 20-24 years | 0.94 | 0.89 | 1.07 |
| Slovakia | 20-24 years | 0.94 | 0.88 | 1.06 |
| Colombia | 20-24 years | 0.94 | 0.83 | 1.60 |
| Central African Republic | 20-24 years | 0.95 | 0.87 | 1.00 |
| New Zealand | 20-24 years | 0.95 | 0.91 | 1.53 |
| Sri Lanka | 20-24 years | 0.95 | 0.94 | 1.32 |
| Nigeria | 20-24 years | 0.96 | 0.64 | 1.11 |
| C么te d'Ivoire | 20-24 years | 0.96 | 0.82 | 1.08 |
| Burkina Faso | 20-24 years | 0.96 | 0.84 | 1.08 |
| American Samoa | 20-24 years | 0.97 | 0.88 | 1.22 |
| Belize | 20-24 years | 0.98 | 0.33 | 1.26 |
| Togo | 20-24 years | 0.98 | 0.87 | 1.08 |
| Serbia | 20-24 years | 0.98 | 0.96 | 1.03 |
| Georgia | 20-24 years | 0.99 | 0.77 | 1.13 |
| Equatorial Guinea | 20-24 years | 0.99 | 1.00 | 1.00 |
| Cameroon | 20-24 years | 0.99 | 0.88 | 1.08 |
| Eswatini | 20-24 years | 0.99 | 0.85 | 1.13 |
| Namibia | 20-24 years | 0.99 | 0.75 | 1.11 |
| Marshall Islands | 20-24 years | 1.00 | 0.97 | 1.23 |
| Mauritania | 20-24 years | 1.00 | 0.83 | 1.11 |
| Lebanon | 20-24 years | 1.01 | 0.98 | 1.39 |
| Democratic Republic of the Congo | 20-24 years | 1.01 | 1.12 | 0.99 |
| Iran (Islamic Republic of) | 20-24 years | 1.01 | 0.87 | 1.08 |
| Mozambique | 20-24 years | 1.02 | 0.75 | 1.12 |
| Sierra Leone | 20-24 years | 1.02 | 0.95 | 1.09 |
| Guinea | 20-24 years | 1.02 | 0.97 | 1.09 |
| Spain | 20-24 years | 1.02 | 1.00 | 1.20 |
| Kuwait | 20-24 years | 1.02 | 0.94 | 1.48 |
| Angola | 20-24 years | 1.02 | 1.15 | 0.99 |
| Taiwan (Province of China) | 20-24 years | 1.03 | 0.91 | 1.43 |
| Congo | 20-24 years | 1.04 | 1.27 | 0.99 |
| Madagascar | 20-24 years | 1.04 | 0.90 | 1.16 |
| Netherlands | 20-24 years | 1.05 | 1.00 | 1.13 |
| China | 20-24 years | 1.05 | 0.86 | 1.12 |
| Sao Tome and Principe | 20-24 years | 1.06 | 1.04 | 1.11 |
| Albania | 20-24 years | 1.07 | 0.56 | 1.14 |
| Russian Federation | 20-24 years | 1.07 | 1.04 | 1.11 |
| South Africa | 20-24 years | 1.07 | 0.92 | 1.13 |
| Niger | 20-24 years | 1.07 | 1.07 | 1.09 |
| Zimbabwe | 20-24 years | 1.07 | 1.06 | 1.12 |
| Democratic People's Republic of Korea | 20-24 years | 1.07 | 1.01 | 1.12 |
| Saint Lucia | 20-24 years | 1.08 | 0.78 | 1.25 |
| Republic of Korea | 20-24 years | 1.08 | 1.06 | 1.01 |
| Iraq | 20-24 years | 1.08 | 1.11 | 1.08 |
| United Republic of Tanzania | 20-24 years | 1.09 | 0.78 | 1.16 |
| Malawi | 20-24 years | 1.09 | 0.77 | 1.16 |
| Botswana | 20-24 years | 1.09 | 1.04 | 1.11 |
| Uganda | 20-24 years | 1.09 | 0.80 | 1.15 |
| Liberia | 20-24 years | 1.09 | 1.14 | 1.08 |
| Jordan | 20-24 years | 1.09 | 1.06 | 1.45 |
| Saint Vincent and the Grenadines | 20-24 years | 1.10 | 0.95 | 1.21 |
| Chile | 20-24 years | 1.10 | 1.06 | 1.27 |
| Lesotho | 20-24 years | 1.10 | 1.09 | 1.13 |
| Trinidad and Tobago | 20-24 years | 1.10 | 0.90 | 1.23 |
| Eritrea | 20-24 years | 1.11 | 0.94 | 1.15 |
| Djibouti | 20-24 years | 1.11 | 0.79 | 1.17 |
| Northern Mariana Islands | 20-24 years | 1.11 | 1.01 | 1.22 |
| Algeria | 20-24 years | 1.11 | 1.18 | 1.08 |
| Turkiye | 20-24 years | 1.11 | 1.06 | 1.49 |
| Kenya | 20-24 years | 1.11 | 0.79 | 1.15 |
| Argentina | 20-24 years | 1.11 | 1.02 | 1.14 |
| Syrian Arab Republic | 20-24 years | 1.12 | 1.22 | 1.07 |
| Myanmar | 20-24 years | 1.13 | 0.99 | 1.44 |
| Somalia | 20-24 years | 1.13 | 1.06 | 1.17 |
| Thailand | 20-24 years | 1.14 | 0.80 | 1.30 |
| Ecuador | 20-24 years | 1.14 | 0.64 | 1.22 |
| Lithuania | 20-24 years | 1.14 | 1.12 | 1.02 |
| Guyana | 20-24 years | 1.14 | 1.04 | 1.23 |
| Palestine | 20-24 years | 1.15 | 1.26 | 1.10 |
| Azerbaijan | 20-24 years | 1.15 | 1.16 | 1.15 |
| Cuba | 20-24 years | 1.15 | 0.95 | 1.21 |
| Tajikistan | 20-24 years | 1.15 | 1.18 | 1.16 |
| Zambia | 20-24 years | 1.16 | 1.23 | 1.16 |
| Rwanda | 20-24 years | 1.16 | 1.17 | 1.16 |
| Burundi | 20-24 years | 1.16 | 1.20 | 1.16 |
| Bahamas | 20-24 years | 1.17 | 0.99 | 1.23 |
| Libya | 20-24 years | 1.17 | 1.32 | 1.10 |
| Armenia | 20-24 years | 1.18 | 1.27 | 1.16 |
| Mongolia | 20-24 years | 1.18 | 1.25 | 1.16 |
| South Sudan | 20-24 years | 1.18 | 1.29 | 1.16 |
| Kyrgyzstan | 20-24 years | 1.18 | 1.30 | 1.15 |
| Peru | 20-24 years | 1.19 | 1.03 | 1.21 |
| Morocco | 20-24 years | 1.19 | 1.50 | 1.09 |
| Palau | 20-24 years | 1.20 | 1.19 | 1.38 |
| Malaysia | 20-24 years | 1.20 | 0.98 | 1.32 |
| Canada | 20-24 years | 1.20 | 1.15 | 1.23 |
| Yemen | 20-24 years | 1.20 | 1.56 | 1.08 |
| Comoros | 20-24 years | 1.21 | 1.67 | 1.15 |
| Paraguay | 20-24 years | 1.21 | 1.18 | 1.22 |
| Papua New Guinea | 20-24 years | 1.21 | 1.21 | 1.22 |
| Costa Rica | 20-24 years | 1.21 | 0.71 | 1.27 |
| Uzbekistan | 20-24 years | 1.21 | 1.34 | 1.14 |
| Maldives | 20-24 years | 1.22 | 1.06 | 1.30 |
| Brazil | 20-24 years | 1.22 | 1.00 | 1.29 |
| Ethiopia | 20-24 years | 1.22 | 1.34 | 1.21 |
| Antigua and Barbuda | 20-24 years | 1.22 | 1.19 | 1.23 |
| Jamaica | 20-24 years | 1.22 | 1.25 | 1.23 |
| Mexico | 20-24 years | 1.23 | 0.83 | 1.31 |
| Suriname | 20-24 years | 1.23 | 1.35 | 1.20 |
| Dominican Republic | 20-24 years | 1.25 | 1.29 | 1.24 |
| Sudan | 20-24 years | 1.25 | 1.41 | 1.12 |
| Grenada | 20-24 years | 1.26 | 1.36 | 1.21 |
| Mali | 20-24 years | 1.26 | 1.52 | 1.08 |
| Guatemala | 20-24 years | 1.26 | 0.92 | 1.29 |
| El Salvador | 20-24 years | 1.26 | 1.13 | 1.28 |
| Panama | 20-24 years | 1.26 | 1.12 | 1.28 |
| Sweden | 20-24 years | 1.27 | 1.21 | 1.20 |
| Dominica | 20-24 years | 1.27 | 1.42 | 1.23 |
| Bolivia (Plurinational State of) | 20-24 years | 1.28 | 1.54 | 1.23 |
| Mauritius | 20-24 years | 1.28 | 1.33 | 1.25 |
| Nicaragua | 20-24 years | 1.28 | 1.71 | 1.26 |
| Turkmenistan | 20-24 years | 1.28 | 1.47 | 1.14 |
| Philippines | 20-24 years | 1.30 | 1.31 | 1.29 |
| Honduras | 20-24 years | 1.31 | 2.87 | 1.28 |
| Timor-Leste | 20-24 years | 1.31 | 1.32 | 1.31 |
| Barbados | 20-24 years | 1.32 | 2.25 | 1.22 |
| Cambodia | 20-24 years | 1.35 | 1.47 | 1.23 |
| Pakistan | 20-24 years | 1.37 | 1.40 | 1.23 |
| Indonesia | 20-24 years | 1.39 | 1.41 | 1.33 |
| Kazakhstan | 20-24 years | 1.43 | 1.46 | 1.21 |
| Niue | 20-24 years | 1.44 | 1.44 | 1.42 |
| Afghanistan | 20-24 years | 1.49 | 1.82 | 1.08 |
| Lao People's Democratic Republic | 20-24 years | 1.55 | 1.71 | 1.29 |
| Seychelles | 20-24 years | 1.62 | 2.71 | 1.28 |
| Tokelau | 20-24 years | 1.66 | 1.66 | 1.74 |
| Greenland | 20-24 years | 1.74 | 1.91 | 1.28 |
| Andorra | 20-24 years | 1.76 | 1.88 | 1.18 |
| Estonia | 20-24 years | 1.77 | 2.14 | 0.99 |
| Oman | 20-24 years | 1.81 | 1.82 | 1.51 |
| Saudi Arabia | 20-24 years | 1.99 | 2.03 | 1.60 |
| Haiti | 20-24 years | 2.01 | 2.73 | 1.23 |
| Bahrain | 20-24 years | 2.03 | 2.08 | 1.53 |
| Brunei Darussalam | 25-29 years | 0.16 | 0.12 | 1.08 |
| United States Virgin Islands | 25-29 years | 0.29 | 0.27 | 1.51 |
| Palau | 25-29 years | 0.38 | 0.37 | 1.54 |
| Cook Islands | 25-29 years | 0.42 | 0.39 | 1.40 |
| Iceland | 25-29 years | 0.45 | 0.39 | 1.37 |
| Greece | 25-29 years | 0.54 | 0.52 | 0.62 |
| Bermuda | 25-29 years | 0.57 | 0.53 | 1.36 |
| Poland | 25-29 years | 0.59 | 0.54 | 1.10 |
| Latvia | 25-29 years | 0.59 | 0.51 | 1.00 |
| Ukraine | 25-29 years | 0.59 | 0.55 | 1.06 |
| Singapore | 25-29 years | 0.59 | 0.51 | 1.04 |
| France | 25-29 years | 0.60 | 0.54 | 1.14 |
| Romania | 25-29 years | 0.61 | 0.59 | 1.04 |
| Cyprus | 25-29 years | 0.63 | 0.61 | 1.20 |
| Bulgaria | 25-29 years | 0.67 | 0.66 | 1.08 |
| Guam | 25-29 years | 0.68 | 0.28 | 1.24 |
| Monaco | 25-29 years | 0.68 | 0.65 | 1.10 |
| Slovenia | 25-29 years | 0.70 | 0.64 | 1.15 |
| Russian Federation | 25-29 years | 0.70 | 0.58 | 1.12 |
| Switzerland | 25-29 years | 0.72 | 0.65 | 1.17 |
| Bhutan | 25-29 years | 0.74 | 0.60 | 1.34 |
| Austria | 25-29 years | 0.75 | 0.72 | 1.13 |
| Italy | 25-29 years | 0.76 | 0.73 | 1.16 |
| Germany | 25-29 years | 0.76 | 0.73 | 1.13 |
| Nepal | 25-29 years | 0.76 | 0.69 | 1.18 |
| Japan | 25-29 years | 0.77 | 0.71 | 0.96 |
| New Zealand | 25-29 years | 0.78 | 0.74 | 1.90 |
| Finland | 25-29 years | 0.79 | 0.76 | 1.17 |
| Bangladesh | 25-29 years | 0.79 | 0.67 | 1.53 |
| United Kingdom | 25-29 years | 0.79 | 0.73 | 1.15 |
| Czechia | 25-29 years | 0.80 | 0.77 | 0.99 |
| Vanuatu | 25-29 years | 0.82 | 0.75 | 1.26 |
| Solomon Islands | 25-29 years | 0.82 | 0.72 | 1.24 |
| Sweden | 25-29 years | 0.83 | 0.73 | 1.26 |
| Slovakia | 25-29 years | 0.83 | 0.80 | 0.98 |
| Lithuania | 25-29 years | 0.84 | 0.80 | 1.01 |
| San Marino | 25-29 years | 0.84 | 0.79 | 1.16 |
| Qatar | 25-29 years | 0.85 | 0.79 | 1.43 |
| Republic of Moldova | 25-29 years | 0.86 | 0.83 | 1.02 |
| Kyrgyzstan | 25-29 years | 0.87 | 0.57 | 1.15 |
| Belgium | 25-29 years | 0.88 | 0.82 | 1.41 |
| Uruguay | 25-29 years | 0.88 | 0.79 | 1.38 |
| Puerto Rico | 25-29 years | 0.88 | 0.85 | 1.48 |
| Chile | 25-29 years | 0.89 | 0.82 | 1.37 |
| Chad | 25-29 years | 0.89 | 0.65 | 1.10 |
| Belarus | 25-29 years | 0.90 | 0.88 | 1.00 |
| North Macedonia | 25-29 years | 0.92 | 0.90 | 0.97 |
| Montenegro | 25-29 years | 0.92 | 0.91 | 0.99 |
| Gambia | 25-29 years | 0.92 | 0.63 | 1.10 |
| Cabo Verde | 25-29 years | 0.92 | 0.42 | 1.08 |
| Kiribati | 25-29 years | 0.93 | 0.88 | 1.24 |
| Croatia | 25-29 years | 0.93 | 0.90 | 1.25 |
| Eswatini | 25-29 years | 0.94 | 0.70 | 1.13 |
| Sri Lanka | 25-29 years | 0.94 | 0.92 | 1.55 |
| Tunisia | 25-29 years | 0.94 | 0.91 | 1.52 |
| Estonia | 25-29 years | 0.94 | 0.94 | 0.96 |
| Gabon | 25-29 years | 0.95 | 0.72 | 1.00 |
| Viet Nam | 25-29 years | 0.95 | 0.92 | 1.38 |
| India | 25-29 years | 0.95 | 0.86 | 1.41 |
| Georgia | 25-29 years | 0.95 | 0.72 | 1.14 |
| Hungary | 25-29 years | 0.95 | 0.94 | 1.00 |
| Guinea-Bissau | 25-29 years | 0.96 | 0.84 | 1.09 |
| Ghana | 25-29 years | 0.96 | 0.55 | 1.09 |
| Benin | 25-29 years | 0.96 | 0.66 | 1.10 |
| Burkina Faso | 25-29 years | 0.97 | 0.77 | 1.09 |
| Namibia | 25-29 years | 0.97 | 0.68 | 1.12 |
| Israel | 25-29 years | 0.98 | 0.96 | 1.11 |
| Equatorial Guinea | 25-29 years | 0.98 | 0.94 | 1.00 |
| Mozambique | 25-29 years | 0.98 | 0.62 | 1.13 |
| C么te d'Ivoire | 25-29 years | 0.98 | 0.77 | 1.10 |
| Central African Republic | 25-29 years | 0.99 | 0.98 | 1.00 |
| Democratic Republic of the Congo | 25-29 years | 0.99 | 1.00 | 1.00 |
| Senegal | 25-29 years | 0.99 | 0.77 | 1.10 |
| Portugal | 25-29 years | 1.00 | 1.01 | 0.80 |
| Togo | 25-29 years | 1.00 | 0.84 | 1.09 |
| Nigeria | 25-29 years | 1.00 | 0.63 | 1.12 |
| Azerbaijan | 25-29 years | 1.00 | 0.68 | 1.15 |
| Turkmenistan | 25-29 years | 1.01 | 0.93 | 1.14 |
| Spain | 25-29 years | 1.01 | 0.99 | 1.13 |
| Cameroon | 25-29 years | 1.02 | 0.89 | 1.09 |
| Madagascar | 25-29 years | 1.02 | 0.85 | 1.16 |
| Egypt | 25-29 years | 1.02 | 1.01 | 1.06 |
| Colombia | 25-29 years | 1.02 | 0.85 | 1.80 |
| Republic of Korea | 25-29 years | 1.02 | 0.98 | 1.03 |
| Mongolia | 25-29 years | 1.03 | 0.81 | 1.16 |
| Angola | 25-29 years | 1.03 | 1.16 | 1.00 |
| Grenada | 25-29 years | 1.03 | 0.84 | 1.23 |
| Guinea | 25-29 years | 1.04 | 0.98 | 1.10 |
| South Africa | 25-29 years | 1.04 | 0.82 | 1.14 |
| Sierra Leone | 25-29 years | 1.04 | 0.97 | 1.10 |
| Australia | 25-29 years | 1.04 | 0.99 | 1.66 |
| Mauritania | 25-29 years | 1.04 | 0.83 | 1.12 |
| Congo | 25-29 years | 1.05 | 1.34 | 1.00 |
| Lebanon | 25-29 years | 1.05 | 1.02 | 1.44 |
| Botswana | 25-29 years | 1.05 | 0.88 | 1.12 |
| Serbia | 25-29 years | 1.06 | 1.06 | 0.95 |
| Liberia | 25-29 years | 1.06 | 1.04 | 1.10 |
| Niger | 25-29 years | 1.06 | 1.02 | 1.10 |
| Iran (Islamic Republic of) | 25-29 years | 1.06 | 0.98 | 1.10 |
| Tajikistan | 25-29 years | 1.07 | 0.94 | 1.16 |
| Lesotho | 25-29 years | 1.07 | 1.02 | 1.13 |
| Armenia | 25-29 years | 1.07 | 0.81 | 1.16 |
| Seychelles | 25-29 years | 1.08 | 0.84 | 1.32 |
| China | 25-29 years | 1.08 | 0.95 | 1.13 |
| Norway | 25-29 years | 1.08 | 1.13 | 0.86 |
| Bosnia and Herzegovina | 25-29 years | 1.08 | 1.08 | 0.98 |
| Malawi | 25-29 years | 1.09 | 0.78 | 1.16 |
| Malta | 25-29 years | 1.09 | 1.08 | 1.13 |
| Uganda | 25-29 years | 1.09 | 0.78 | 1.15 |
| United Republic of Tanzania | 25-29 years | 1.09 | 0.76 | 1.16 |
| Uzbekistan | 25-29 years | 1.10 | 1.07 | 1.14 |
| Netherlands | 25-29 years | 1.10 | 1.08 | 1.13 |
| Albania | 25-29 years | 1.10 | 0.84 | 1.15 |
| Djibouti | 25-29 years | 1.11 | 0.79 | 1.17 |
| Jordan | 25-29 years | 1.11 | 1.06 | 1.49 |
| Eritrea | 25-29 years | 1.11 | 0.95 | 1.15 |
| Argentina | 25-29 years | 1.12 | 1.05 | 1.15 |
| Turkiye | 25-29 years | 1.12 | 1.06 | 1.54 |
| Zimbabwe | 25-29 years | 1.12 | 1.13 | 1.13 |
| Kuwait | 25-29 years | 1.12 | 1.04 | 1.53 |
| Somalia | 25-29 years | 1.13 | 1.03 | 1.17 |
| Kenya | 25-29 years | 1.13 | 0.98 | 1.16 |
| Fiji | 25-29 years | 1.14 | 1.12 | 1.22 |
| Trinidad and Tobago | 25-29 years | 1.15 | 0.99 | 1.27 |
| Guyana | 25-29 years | 1.15 | 1.03 | 1.25 |
| Tonga | 25-29 years | 1.15 | 1.07 | 1.21 |
| Pakistan | 25-29 years | 1.15 | 1.13 | 1.27 |
| Burundi | 25-29 years | 1.15 | 1.12 | 1.17 |
| Rwanda | 25-29 years | 1.15 | 1.14 | 1.17 |
| Ecuador | 25-29 years | 1.16 | 0.69 | 1.25 |
| Sao Tome and Principe | 25-29 years | 1.16 | 1.29 | 1.12 |
| Zambia | 25-29 years | 1.17 | 1.24 | 1.18 |
| Timor-Leste | 25-29 years | 1.17 | 1.08 | 1.36 |
| South Sudan | 25-29 years | 1.17 | 1.27 | 1.17 |
| Comoros | 25-29 years | 1.17 | 1.36 | 1.16 |
| Peru | 25-29 years | 1.18 | 0.88 | 1.23 |
| Bahamas | 25-29 years | 1.18 | 1.03 | 1.25 |
| Cuba | 25-29 years | 1.19 | 1.05 | 1.23 |
| Belize | 25-29 years | 1.19 | 1.00 | 1.29 |
| Iraq | 25-29 years | 1.19 | 1.45 | 1.10 |
| Mali | 25-29 years | 1.21 | 1.50 | 1.10 |
| Myanmar | 25-29 years | 1.21 | 1.06 | 1.50 |
| Ethiopia | 25-29 years | 1.21 | 1.22 | 1.22 |
| Paraguay | 25-29 years | 1.22 | 1.13 | 1.25 |
| Palestine | 25-29 years | 1.22 | 1.54 | 1.13 |
| Denmark | 25-29 years | 1.22 | 1.22 | 1.20 |
| Democratic People's Republic of Korea | 25-29 years | 1.22 | 1.40 | 1.14 |
| Bolivia (Plurinational State of) | 25-29 years | 1.23 | 1.18 | 1.25 |
| Algeria | 25-29 years | 1.23 | 1.57 | 1.10 |
| Ireland | 25-29 years | 1.24 | 1.22 | 1.17 |
| Syrian Arab Republic | 25-29 years | 1.24 | 1.53 | 1.09 |
| Saint Kitts and Nevis | 25-29 years | 1.24 | 1.22 | 1.40 |
| Brazil | 25-29 years | 1.25 | 1.02 | 1.32 |
| Taiwan (Province of China) | 25-29 years | 1.25 | 1.13 | 1.58 |
| Suriname | 25-29 years | 1.25 | 1.35 | 1.23 |
| Thailand | 25-29 years | 1.25 | 1.03 | 1.35 |
| Micronesia (Federated States of) | 25-29 years | 1.26 | 1.26 | 1.24 |
| Saint Vincent and the Grenadines | 25-29 years | 1.26 | 1.31 | 1.24 |
| Costa Rica | 25-29 years | 1.27 | 1.04 | 1.31 |
| Barbados | 25-29 years | 1.27 | 1.42 | 1.25 |
| Tuvalu | 25-29 years | 1.28 | 1.27 | 1.77 |
| Sudan | 25-29 years | 1.29 | 1.46 | 1.14 |
| American Samoa | 25-29 years | 1.29 | 1.32 | 1.24 |
| Mexico | 25-29 years | 1.29 | 0.99 | 1.37 |
| Papua New Guinea | 25-29 years | 1.29 | 1.31 | 1.23 |
| Yemen | 25-29 years | 1.30 | 1.81 | 1.09 |
| Canada | 25-29 years | 1.31 | 1.27 | 1.34 |
| Libya | 25-29 years | 1.31 | 1.74 | 1.13 |
| Samoa | 25-29 years | 1.32 | 1.36 | 1.23 |
| Malaysia | 25-29 years | 1.32 | 1.24 | 1.37 |
| Guatemala | 25-29 years | 1.32 | 1.35 | 1.33 |
| Nicaragua | 25-29 years | 1.33 | 1.97 | 1.29 |
| El Salvador | 25-29 years | 1.33 | 1.43 | 1.33 |
| Maldives | 25-29 years | 1.33 | 1.36 | 1.32 |
| Jamaica | 25-29 years | 1.33 | 1.57 | 1.25 |
| Kazakhstan | 25-29 years | 1.34 | 1.35 | 1.21 |
| United Arab Emirates | 25-29 years | 1.35 | 1.96 | 1.10 |
| Honduras | 25-29 years | 1.36 | 3.29 | 1.31 |
| Morocco | 25-29 years | 1.36 | 2.23 | 1.12 |
| Dominican Republic | 25-29 years | 1.36 | 1.59 | 1.26 |
| Antigua and Barbuda | 25-29 years | 1.37 | 2.15 | 1.26 |
| Dominica | 25-29 years | 1.38 | 1.76 | 1.25 |
| Panama | 25-29 years | 1.39 | 2.50 | 1.32 |
| United States of America | 25-29 years | 1.40 | 1.38 | 1.53 |
| Saint Lucia | 25-29 years | 1.40 | 1.67 | 1.28 |
| Philippines | 25-29 years | 1.41 | 1.49 | 1.33 |
| Northern Mariana Islands | 25-29 years | 1.42 | 1.64 | 1.25 |
| Cambodia | 25-29 years | 1.47 | 1.71 | 1.26 |
| Nauru | 25-29 years | 1.48 | 1.48 | 1.71 |
| Marshall Islands | 25-29 years | 1.57 | 1.64 | 1.25 |
| Lao People's Democratic Republic | 25-29 years | 1.58 | 1.73 | 1.34 |
| Mauritius | 25-29 years | 1.59 | 2.15 | 1.30 |
| Indonesia | 25-29 years | 1.61 | 1.68 | 1.38 |
| Venezuela (Bolivarian Republic of) | 25-29 years | 1.63 | 1.60 | 1.81 |
| Afghanistan | 25-29 years | 1.63 | 2.10 | 1.11 |
| Niue | 25-29 years | 1.74 | 1.74 | 1.67 |
| Andorra | 25-29 years | 1.97 | 2.11 | 1.21 |
| Luxembourg | 25-29 years | 2.10 | 2.62 | 1.15 |
| Bahrain | 25-29 years | 2.13 | 2.20 | 1.59 |
| Oman | 25-29 years | 2.16 | 2.19 | 1.57 |
| Saudi Arabia | 25-29 years | 2.17 | 2.20 | 1.68 |
| Haiti | 25-29 years | 2.17 | 2.86 | 1.26 |
| Tokelau | 25-29 years | 2.20 | 2.21 | 1.96 |
| Greenland | 25-29 years | 3.24 | 3.83 | 1.40 |
| Palau | 30-34 years | 0.44 | 0.43 | 1.58 |
| Cook Islands | 30-34 years | 0.45 | 0.43 | 1.38 |
| New Zealand | 30-34 years | 0.50 | 0.47 | 2.39 |
| Ukraine | 30-34 years | 0.56 | 0.52 | 1.05 |
| Greece | 30-34 years | 0.58 | 0.57 | 0.62 |
| Italy | 30-34 years | 0.60 | 0.58 | 1.09 |
| Poland | 30-34 years | 0.61 | 0.58 | 1.03 |
| Lithuania | 30-34 years | 0.62 | 0.57 | 0.99 |
| Monaco | 30-34 years | 0.63 | 0.60 | 1.09 |
| Latvia | 30-34 years | 0.64 | 0.60 | 0.98 |
| United States Virgin Islands | 30-34 years | 0.71 | 0.69 | 1.61 |
| Austria | 30-34 years | 0.71 | 0.69 | 1.23 |
| France | 30-34 years | 0.71 | 0.68 | 1.12 |
| Israel | 30-34 years | 0.72 | 0.69 | 1.07 |
| Finland | 30-34 years | 0.72 | 0.69 | 1.17 |
| Singapore | 30-34 years | 0.73 | 0.65 | 1.07 |
| Bermuda | 30-34 years | 0.75 | 0.69 | 1.41 |
| Iceland | 30-34 years | 0.76 | 0.72 | 1.32 |
| United Kingdom | 30-34 years | 0.78 | 0.73 | 1.15 |
| Slovenia | 30-34 years | 0.78 | 0.76 | 1.03 |
| Belarus | 30-34 years | 0.79 | 0.77 | 0.99 |
| Guam | 30-34 years | 0.81 | 0.63 | 1.26 |
| Montenegro | 30-34 years | 0.81 | 0.80 | 0.91 |
| Slovakia | 30-34 years | 0.83 | 0.81 | 0.89 |
| Russian Federation | 30-34 years | 0.84 | 0.76 | 1.11 |
| Germany | 30-34 years | 0.85 | 0.84 | 1.11 |
| Chad | 30-34 years | 0.86 | 0.57 | 1.11 |
| Bangladesh | 30-34 years | 0.86 | 0.74 | 1.54 |
| Republic of Moldova | 30-34 years | 0.87 | 0.85 | 1.02 |
| Burkina Faso | 30-34 years | 0.87 | 0.54 | 1.10 |
| Brunei Darussalam | 30-34 years | 0.88 | 0.87 | 1.12 |
| San Marino | 30-34 years | 0.89 | 0.85 | 1.15 |
| Benin | 30-34 years | 0.89 | 0.44 | 1.11 |
| Puerto Rico | 30-34 years | 0.89 | 0.85 | 1.58 |
| Gambia | 30-34 years | 0.90 | 0.55 | 1.11 |
| Solomon Islands | 30-34 years | 0.90 | 0.86 | 1.23 |
| Denmark | 30-34 years | 0.90 | 0.88 | 1.20 |
| Kyrgyzstan | 30-34 years | 0.91 | 0.71 | 1.16 |
| Guinea-Bissau | 30-34 years | 0.92 | 0.76 | 1.09 |
| Georgia | 30-34 years | 0.92 | 0.72 | 1.16 |
| C么te d'Ivoire | 30-34 years | 0.93 | 0.60 | 1.11 |
| Vanuatu | 30-34 years | 0.93 | 0.90 | 1.26 |
| Madagascar | 30-34 years | 0.93 | 0.69 | 1.16 |
| Mozambique | 30-34 years | 0.93 | 0.51 | 1.14 |
| Romania | 30-34 years | 0.94 | 0.94 | 0.96 |
| Gabon | 30-34 years | 0.94 | 0.75 | 1.01 |
| United States of America | 30-34 years | 0.94 | 0.87 | 1.62 |
| Togo | 30-34 years | 0.94 | 0.68 | 1.10 |
| Bhutan | 30-34 years | 0.94 | 0.85 | 1.35 |
| Guinea | 30-34 years | 0.95 | 0.78 | 1.10 |
| Senegal | 30-34 years | 0.95 | 0.63 | 1.10 |
| Australia | 30-34 years | 0.95 | 0.88 | 2.08 |
| Sierra Leone | 30-34 years | 0.96 | 0.73 | 1.11 |
| Cabo Verde | 30-34 years | 0.96 | 0.44 | 1.10 |
| Nigeria | 30-34 years | 0.96 | 0.46 | 1.13 |
| Liberia | 30-34 years | 0.96 | 0.73 | 1.11 |
| Eswatini | 30-34 years | 0.96 | 0.77 | 1.14 |
| Central African Republic | 30-34 years | 0.96 | 0.92 | 1.01 |
| Azerbaijan | 30-34 years | 0.96 | 0.62 | 1.16 |
| Cameroon | 30-34 years | 0.97 | 0.70 | 1.10 |
| Niger | 30-34 years | 0.97 | 0.75 | 1.11 |
| Czechia | 30-34 years | 0.97 | 0.97 | 0.90 |
| Namibia | 30-34 years | 0.98 | 0.71 | 1.13 |
| Democratic Republic of the Congo | 30-34 years | 0.98 | 0.96 | 1.00 |
| Bulgaria | 30-34 years | 0.98 | 0.98 | 1.01 |
| Kiribati | 30-34 years | 0.98 | 0.96 | 1.23 |
| Equatorial Guinea | 30-34 years | 0.99 | 0.97 | 1.01 |
| Zimbabwe | 30-34 years | 0.99 | 0.93 | 1.13 |
| Ghana | 30-34 years | 0.99 | 0.65 | 1.10 |
| Nepal | 30-34 years | 1.01 | 0.98 | 1.18 |
| Mongolia | 30-34 years | 1.01 | 0.85 | 1.17 |
| Angola | 30-34 years | 1.01 | 1.07 | 1.01 |
| Mauritania | 30-34 years | 1.01 | 0.74 | 1.12 |
| Croatia | 30-34 years | 1.02 | 1.01 | 1.09 |
| Spain | 30-34 years | 1.04 | 1.02 | 1.05 |
| Malawi | 30-34 years | 1.04 | 0.59 | 1.16 |
| Qatar | 30-34 years | 1.04 | 0.96 | 1.49 |
| Somalia | 30-34 years | 1.04 | 0.72 | 1.17 |
| United Republic of Tanzania | 30-34 years | 1.04 | 0.59 | 1.17 |
| China | 30-34 years | 1.04 | 0.90 | 1.13 |
| Colombia | 30-34 years | 1.05 | 0.88 | 2.01 |
| Portugal | 30-34 years | 1.05 | 1.06 | 0.75 |
| Tajikistan | 30-34 years | 1.05 | 0.93 | 1.16 |
| Uganda | 30-34 years | 1.05 | 0.56 | 1.16 |
| Eritrea | 30-34 years | 1.05 | 0.71 | 1.16 |
| Sweden | 30-34 years | 1.06 | 0.96 | 1.33 |
| Uzbekistan | 30-34 years | 1.06 | 1.01 | 1.14 |
| Burundi | 30-34 years | 1.06 | 0.72 | 1.17 |
| Congo | 30-34 years | 1.06 | 1.29 | 1.00 |
| Switzerland | 30-34 years | 1.06 | 1.05 | 1.16 |
| Botswana | 30-34 years | 1.07 | 0.91 | 1.14 |
| Guyana | 30-34 years | 1.07 | 0.87 | 1.26 |
| Djibouti | 30-34 years | 1.07 | 0.63 | 1.17 |
| South Sudan | 30-34 years | 1.07 | 0.81 | 1.17 |
| Ireland | 30-34 years | 1.07 | 1.04 | 1.17 |
| Cyprus | 30-34 years | 1.07 | 1.05 | 1.16 |
| Canada | 30-34 years | 1.07 | 1.00 | 1.41 |
| Timor-Leste | 30-34 years | 1.08 | 0.95 | 1.36 |
| Lesotho | 30-34 years | 1.09 | 1.05 | 1.14 |
| Serbia | 30-34 years | 1.09 | 1.10 | 0.86 |
| Japan | 30-34 years | 1.09 | 1.07 | 0.97 |
| Iran (Islamic Republic of) | 30-34 years | 1.10 | 1.05 | 1.13 |
| Sri Lanka | 30-34 years | 1.10 | 1.07 | 1.72 |
| Comoros | 30-34 years | 1.10 | 0.86 | 1.16 |
| Tunisia | 30-34 years | 1.10 | 1.08 | 1.59 |
| Rwanda | 30-34 years | 1.10 | 0.84 | 1.17 |
| Kenya | 30-34 years | 1.12 | 0.83 | 1.17 |
| United Arab Emirates | 30-34 years | 1.12 | 1.13 | 1.12 |
| Albania | 30-34 years | 1.13 | 1.05 | 1.16 |
| Saint Vincent and the Grenadines | 30-34 years | 1.13 | 1.01 | 1.25 |
| Estonia | 30-34 years | 1.14 | 1.21 | 0.96 |
| Zambia | 30-34 years | 1.14 | 0.97 | 1.18 |
| Ethiopia | 30-34 years | 1.14 | 0.83 | 1.23 |
| Armenia | 30-34 years | 1.14 | 1.07 | 1.17 |
| Seychelles | 30-34 years | 1.15 | 0.82 | 1.34 |
| Chile | 30-34 years | 1.15 | 1.09 | 1.46 |
| Mali | 30-34 years | 1.15 | 1.31 | 1.11 |
| Argentina | 30-34 years | 1.15 | 1.22 | 1.16 |
| Turkmenistan | 30-34 years | 1.16 | 1.17 | 1.15 |
| Belgium | 30-34 years | 1.16 | 1.14 | 1.39 |
| Viet Nam | 30-34 years | 1.16 | 1.14 | 1.41 |
| India | 30-34 years | 1.17 | 1.13 | 1.41 |
| Egypt | 30-34 years | 1.18 | 1.27 | 1.08 |
| Papua New Guinea | 30-34 years | 1.19 | 1.18 | 1.22 |
| Yemen | 30-34 years | 1.20 | 1.41 | 1.10 |
| Jordan | 30-34 years | 1.20 | 1.15 | 1.54 |
| Luxembourg | 30-34 years | 1.20 | 1.20 | 1.13 |
| Uruguay | 30-34 years | 1.20 | 1.13 | 1.45 |
| Lebanon | 30-34 years | 1.20 | 1.16 | 1.48 |
| Sao Tome and Principe | 30-34 years | 1.21 | 1.46 | 1.13 |
| Belize | 30-34 years | 1.22 | 1.02 | 1.31 |
| Trinidad and Tobago | 30-34 years | 1.22 | 1.14 | 1.29 |
| Ecuador | 30-34 years | 1.23 | 1.08 | 1.26 |
| South Africa | 30-34 years | 1.23 | 1.49 | 1.15 |
| Taiwan (Province of China) | 30-34 years | 1.24 | 1.08 | 1.72 |
| Mexico | 30-34 years | 1.25 | 0.81 | 1.40 |
| Iraq | 30-34 years | 1.25 | 1.64 | 1.12 |
| Peru | 30-34 years | 1.25 | 1.45 | 1.24 |
| Netherlands | 30-34 years | 1.26 | 1.28 | 1.10 |
| Democratic People's Republic of Korea | 30-34 years | 1.26 | 1.42 | 1.14 |
| Grenada | 30-34 years | 1.27 | 1.32 | 1.23 |
| Palestine | 30-34 years | 1.27 | 1.86 | 1.15 |
| Pakistan | 30-34 years | 1.27 | 1.28 | 1.27 |
| Paraguay | 30-34 years | 1.27 | 1.40 | 1.26 |
| Syrian Arab Republic | 30-34 years | 1.28 | 1.72 | 1.11 |
| Cuba | 30-34 years | 1.28 | 1.44 | 1.24 |
| Kuwait | 30-34 years | 1.28 | 1.20 | 1.59 |
| Suriname | 30-34 years | 1.29 | 1.41 | 1.24 |
| Costa Rica | 30-34 years | 1.29 | 1.20 | 1.32 |
| Sudan | 30-34 years | 1.30 | 1.48 | 1.16 |
| Thailand | 30-34 years | 1.32 | 1.20 | 1.38 |
| Nicaragua | 30-34 years | 1.32 | 1.55 | 1.31 |
| Myanmar | 30-34 years | 1.32 | 1.20 | 1.51 |
| Barbados | 30-34 years | 1.32 | 1.58 | 1.27 |
| Bahamas | 30-34 years | 1.32 | 1.50 | 1.27 |
| Bolivia (Plurinational State of) | 30-34 years | 1.33 | 1.73 | 1.25 |
| Algeria | 30-34 years | 1.33 | 1.91 | 1.12 |
| Guatemala | 30-34 years | 1.34 | 1.29 | 1.35 |
| El Salvador | 30-34 years | 1.34 | 1.24 | 1.36 |
| Libya | 30-34 years | 1.35 | 1.99 | 1.15 |
| Brazil | 30-34 years | 1.35 | 1.49 | 1.34 |
| Turkiye | 30-34 years | 1.36 | 1.31 | 1.59 |
| Republic of Korea | 30-34 years | 1.36 | 1.39 | 1.04 |
| Panama | 30-34 years | 1.37 | 1.72 | 1.33 |
| Malaysia | 30-34 years | 1.38 | 1.37 | 1.39 |
| North Macedonia | 30-34 years | 1.39 | 1.42 | 0.90 |
| Honduras | 30-34 years | 1.39 | 3.36 | 1.32 |
| Maldives | 30-34 years | 1.39 | 1.71 | 1.31 |
| Jamaica | 30-34 years | 1.40 | 1.75 | 1.25 |
| Kazakhstan | 30-34 years | 1.40 | 1.42 | 1.20 |
| Hungary | 30-34 years | 1.41 | 1.43 | 0.91 |
| Lao People's Democratic Republic | 30-34 years | 1.43 | 1.49 | 1.35 |
| Dominica | 30-34 years | 1.44 | 1.90 | 1.26 |
| Saint Lucia | 30-34 years | 1.46 | 1.80 | 1.29 |
| Afghanistan | 30-34 years | 1.47 | 1.76 | 1.12 |
| Saint Kitts and Nevis | 30-34 years | 1.47 | 1.46 | 1.51 |
| Tonga | 30-34 years | 1.47 | 1.72 | 1.22 |
| Dominican Republic | 30-34 years | 1.47 | 1.95 | 1.27 |
| Antigua and Barbuda | 30-34 years | 1.48 | 3.45 | 1.27 |
| Philippines | 30-34 years | 1.49 | 1.66 | 1.34 |
| Micronesia (Federated States of) | 30-34 years | 1.49 | 1.53 | 1.24 |
| Morocco | 30-34 years | 1.50 | 2.88 | 1.13 |
| Tuvalu | 30-34 years | 1.53 | 1.53 | 1.85 |
| Venezuela (Bolivarian Republic of) | 30-34 years | 1.54 | 1.50 | 1.96 |
| Cambodia | 30-34 years | 1.54 | 1.86 | 1.26 |
| Mauritius | 30-34 years | 1.54 | 2.01 | 1.32 |
| Bosnia and Herzegovina | 30-34 years | 1.58 | 1.67 | 0.89 |
| American Samoa | 30-34 years | 1.59 | 1.69 | 1.24 |
| Fiji | 30-34 years | 1.59 | 1.66 | 1.21 |
| Samoa | 30-34 years | 1.60 | 1.69 | 1.22 |
| Nauru | 30-34 years | 1.74 | 1.74 | 1.69 |
| Indonesia | 30-34 years | 1.75 | 1.87 | 1.38 |
| Niue | 30-34 years | 1.75 | 1.75 | 1.78 |
| Marshall Islands | 30-34 years | 1.76 | 1.83 | 1.24 |
| Norway | 30-34 years | 1.77 | 2.91 | 0.88 |
| Northern Mariana Islands | 30-34 years | 1.78 | 2.15 | 1.26 |
| Malta | 30-34 years | 1.88 | 1.92 | 1.13 |
| Haiti | 30-34 years | 1.90 | 2.31 | 1.26 |
| Bahrain | 30-34 years | 1.94 | 1.97 | 1.66 |
| Oman | 30-34 years | 2.08 | 2.11 | 1.62 |
| Andorra | 30-34 years | 2.08 | 2.24 | 1.23 |
| Greenland | 30-34 years | 2.15 | 2.27 | 1.46 |
| Saudi Arabia | 30-34 years | 2.15 | 2.18 | 1.78 |
| Tokelau | 30-34 years | 2.49 | 2.48 | 2.10 |
| Palau | 35-39 years | 0.37 | 0.36 | 1.50 |
| Cook Islands | 35-39 years | 0.37 | 0.35 | 1.26 |
| Poland | 35-39 years | 0.46 | 0.44 | 0.88 |
| Norway | 35-39 years | 0.49 | 0.32 | 0.87 |
| Monaco | 35-39 years | 0.58 | 0.55 | 1.09 |
| Greece | 35-39 years | 0.59 | 0.58 | 0.65 |
| Republic of Moldova | 35-39 years | 0.60 | 0.55 | 1.02 |
| Russian Federation | 35-39 years | 0.63 | 0.53 | 1.07 |
| Ukraine | 35-39 years | 0.63 | 0.60 | 1.01 |
| Germany | 35-39 years | 0.69 | 0.67 | 1.09 |
| Italy | 35-39 years | 0.71 | 0.69 | 0.91 |
| Qatar | 35-39 years | 0.71 | 0.60 | 1.54 |
| Austria | 35-39 years | 0.71 | 0.70 | 1.01 |
| Cyprus | 35-39 years | 0.72 | 0.70 | 1.13 |
| Lithuania | 35-39 years | 0.73 | 0.71 | 0.97 |
| Luxembourg | 35-39 years | 0.74 | 0.67 | 1.12 |
| Israel | 35-39 years | 0.75 | 0.72 | 1.07 |
| Kiribati | 35-39 years | 0.75 | 0.70 | 1.21 |
| Denmark | 35-39 years | 0.75 | 0.73 | 1.17 |
| New Zealand | 35-39 years | 0.76 | 0.74 | 2.19 |
| Finland | 35-39 years | 0.76 | 0.74 | 1.13 |
| United Kingdom | 35-39 years | 0.76 | 0.73 | 1.14 |
| Burkina Faso | 35-39 years | 0.77 | 0.41 | 1.11 |
| France | 35-39 years | 0.79 | 0.76 | 1.10 |
| Solomon Islands | 35-39 years | 0.79 | 0.74 | 1.20 |
| Portugal | 35-39 years | 0.80 | 0.80 | 0.72 |
| Latvia | 35-39 years | 0.81 | 0.79 | 0.97 |
| Mozambique | 35-39 years | 0.82 | 0.47 | 1.15 |
| Bermuda | 35-39 years | 0.83 | 0.78 | 1.44 |
| Malta | 35-39 years | 0.83 | 0.81 | 1.11 |
| Chad | 35-39 years | 0.83 | 0.54 | 1.12 |
| Iceland | 35-39 years | 0.84 | 0.80 | 1.27 |
| San Marino | 35-39 years | 0.84 | 0.81 | 1.15 |
| Vanuatu | 35-39 years | 0.87 | 0.83 | 1.24 |
| Benin | 35-39 years | 0.87 | 0.42 | 1.12 |
| Papua New Guinea | 35-39 years | 0.88 | 0.85 | 1.20 |
| Switzerland | 35-39 years | 0.88 | 0.85 | 1.13 |
| Georgia | 35-39 years | 0.89 | 0.72 | 1.19 |
| Zimbabwe | 35-39 years | 0.89 | 0.80 | 1.14 |
| Guinea | 35-39 years | 0.89 | 0.65 | 1.11 |
| Romania | 35-39 years | 0.91 | 0.91 | 0.91 |
| C么te d'Ivoire | 35-39 years | 0.92 | 0.56 | 1.12 |
| Japan | 35-39 years | 0.92 | 0.89 | 1.00 |
| Niger | 35-39 years | 0.92 | 0.65 | 1.11 |
| Guinea-Bissau | 35-39 years | 0.92 | 0.76 | 1.10 |
| Togo | 35-39 years | 0.92 | 0.63 | 1.11 |
| Slovenia | 35-39 years | 0.92 | 0.91 | 0.95 |
| Namibia | 35-39 years | 0.92 | 0.66 | 1.15 |
| Puerto Rico | 35-39 years | 0.92 | 0.89 | 1.64 |
| Gambia | 35-39 years | 0.93 | 0.62 | 1.12 |
| Eswatini | 35-39 years | 0.93 | 0.75 | 1.15 |
| Sierra Leone | 35-39 years | 0.93 | 0.67 | 1.11 |
| Madagascar | 35-39 years | 0.93 | 0.77 | 1.16 |
| Cameroon | 35-39 years | 0.94 | 0.65 | 1.10 |
| Senegal | 35-39 years | 0.95 | 0.64 | 1.11 |
| Liberia | 35-39 years | 0.96 | 0.73 | 1.11 |
| Nigeria | 35-39 years | 0.96 | 0.48 | 1.14 |
| Mongolia | 35-39 years | 0.96 | 0.84 | 1.19 |
| Central African Republic | 35-39 years | 0.96 | 0.93 | 1.01 |
| Estonia | 35-39 years | 0.97 | 0.97 | 0.97 |
| Chile | 35-39 years | 0.97 | 0.89 | 1.47 |
| Netherlands | 35-39 years | 0.97 | 0.95 | 1.09 |
| Cabo Verde | 35-39 years | 0.98 | 0.49 | 1.13 |
| Gabon | 35-39 years | 0.98 | 0.92 | 1.03 |
| Ghana | 35-39 years | 0.98 | 0.63 | 1.11 |
| Democratic Republic of the Congo | 35-39 years | 1.00 | 1.03 | 1.01 |
| Somalia | 35-39 years | 1.00 | 0.77 | 1.17 |
| Mauritania | 35-39 years | 1.01 | 0.78 | 1.10 |
| Equatorial Guinea | 35-39 years | 1.01 | 1.07 | 1.01 |
| Belarus | 35-39 years | 1.01 | 1.01 | 0.96 |
| Lesotho | 35-39 years | 1.01 | 0.92 | 1.15 |
| Slovakia | 35-39 years | 1.01 | 1.02 | 0.83 |
| Malawi | 35-39 years | 1.02 | 0.67 | 1.17 |
| Azerbaijan | 35-39 years | 1.02 | 0.77 | 1.18 |
| Jordan | 35-39 years | 1.02 | 0.97 | 1.57 |
| Singapore | 35-39 years | 1.02 | 0.97 | 1.15 |
| Uganda | 35-39 years | 1.02 | 0.61 | 1.17 |
| Czechia | 35-39 years | 1.03 | 1.03 | 0.85 |
| Botswana | 35-39 years | 1.03 | 0.81 | 1.16 |
| United States Virgin Islands | 35-39 years | 1.03 | 1.01 | 1.62 |
| Angola | 35-39 years | 1.03 | 1.07 | 1.01 |
| Burundi | 35-39 years | 1.03 | 0.78 | 1.17 |
| Croatia | 35-39 years | 1.04 | 1.03 | 0.96 |
| South Sudan | 35-39 years | 1.04 | 0.83 | 1.17 |
| United States of America | 35-39 years | 1.04 | 0.98 | 1.53 |
| Kyrgyzstan | 35-39 years | 1.05 | 0.97 | 1.17 |
| Canada | 35-39 years | 1.05 | 0.97 | 1.37 |
| United Republic of Tanzania | 35-39 years | 1.05 | 0.72 | 1.18 |
| Niue | 35-39 years | 1.06 | 1.04 | 1.70 |
| Ireland | 35-39 years | 1.06 | 1.04 | 1.17 |
| China | 35-39 years | 1.07 | 1.00 | 1.13 |
| Djibouti | 35-39 years | 1.08 | 0.79 | 1.18 |
| Spain | 35-39 years | 1.08 | 1.08 | 1.05 |
| Iran (Islamic Republic of) | 35-39 years | 1.08 | 0.96 | 1.16 |
| Bhutan | 35-39 years | 1.09 | 1.05 | 1.32 |
| Eritrea | 35-39 years | 1.09 | 0.94 | 1.17 |
| United Arab Emirates | 35-39 years | 1.09 | 0.99 | 1.15 |
| Tajikistan | 35-39 years | 1.09 | 1.03 | 1.16 |
| Guyana | 35-39 years | 1.09 | 0.95 | 1.27 |
| Montenegro | 35-39 years | 1.10 | 1.10 | 0.87 |
| Rwanda | 35-39 years | 1.10 | 0.92 | 1.17 |
| Timor-Leste | 35-39 years | 1.10 | 1.00 | 1.33 |
| Colombia | 35-39 years | 1.10 | 0.92 | 2.08 |
| Belgium | 35-39 years | 1.11 | 1.08 | 1.36 |
| Sweden | 35-39 years | 1.12 | 1.04 | 1.44 |
| Comoros | 35-39 years | 1.13 | 1.04 | 1.16 |
| Mali | 35-39 years | 1.13 | 1.22 | 1.11 |
| Bangladesh | 35-39 years | 1.13 | 1.08 | 1.51 |
| Albania | 35-39 years | 1.13 | 1.05 | 1.17 |
| Pakistan | 35-39 years | 1.14 | 1.12 | 1.26 |
| Ethiopia | 35-39 years | 1.14 | 0.91 | 1.23 |
| Kenya | 35-39 years | 1.14 | 1.06 | 1.18 |
| Turkmenistan | 35-39 years | 1.15 | 1.15 | 1.16 |
| Congo | 35-39 years | 1.15 | 1.52 | 1.00 |
| Sri Lanka | 35-39 years | 1.16 | 1.14 | 1.50 |
| Zambia | 35-39 years | 1.16 | 1.11 | 1.19 |
| Nepal | 35-39 years | 1.17 | 1.17 | 1.16 |
| Australia | 35-39 years | 1.17 | 1.12 | 2.31 |
| Serbia | 35-39 years | 1.18 | 1.21 | 0.81 |
| Tunisia | 35-39 years | 1.19 | 1.17 | 1.64 |
| Armenia | 35-39 years | 1.19 | 1.21 | 1.19 |
| Argentina | 35-39 years | 1.19 | 1.44 | 1.17 |
| Hungary | 35-39 years | 1.20 | 1.22 | 0.86 |
| Egypt | 35-39 years | 1.20 | 1.28 | 1.11 |
| Trinidad and Tobago | 35-39 years | 1.21 | 1.12 | 1.30 |
| Uzbekistan | 35-39 years | 1.22 | 1.28 | 1.15 |
| India | 35-39 years | 1.22 | 1.20 | 1.40 |
| Yemen | 35-39 years | 1.23 | 1.39 | 1.12 |
| Syrian Arab Republic | 35-39 years | 1.23 | 1.45 | 1.12 |
| Uruguay | 35-39 years | 1.24 | 1.20 | 1.44 |
| Lebanon | 35-39 years | 1.24 | 1.21 | 1.49 |
| Peru | 35-39 years | 1.24 | 1.36 | 1.24 |
| Tonga | 35-39 years | 1.25 | 1.31 | 1.21 |
| Tuvalu | 35-39 years | 1.25 | 1.24 | 1.79 |
| Bulgaria | 35-39 years | 1.25 | 1.26 | 0.97 |
| Micronesia (Federated States of) | 35-39 years | 1.25 | 1.26 | 1.22 |
| Taiwan (Province of China) | 35-39 years | 1.26 | 1.12 | 1.73 |
| South Africa | 35-39 years | 1.26 | 1.51 | 1.16 |
| Ecuador | 35-39 years | 1.26 | 1.24 | 1.28 |
| Brunei Darussalam | 35-39 years | 1.27 | 1.27 | 1.21 |
| Belize | 35-39 years | 1.28 | 1.26 | 1.31 |
| Kazakhstan | 35-39 years | 1.28 | 1.29 | 1.17 |
| Costa Rica | 35-39 years | 1.28 | 1.13 | 1.32 |
| Cuba | 35-39 years | 1.29 | 1.39 | 1.25 |
| Grenada | 35-39 years | 1.29 | 1.35 | 1.22 |
| Sudan | 35-39 years | 1.29 | 1.41 | 1.17 |
| Saint Vincent and the Grenadines | 35-39 years | 1.30 | 1.36 | 1.25 |
| Republic of Korea | 35-39 years | 1.30 | 1.29 | 1.09 |
| Mexico | 35-39 years | 1.30 | 1.06 | 1.41 |
| Fiji | 35-39 years | 1.32 | 1.34 | 1.20 |
| Bahamas | 35-39 years | 1.32 | 1.43 | 1.27 |
| Guam | 35-39 years | 1.32 | 1.36 | 1.27 |
| Thailand | 35-39 years | 1.32 | 1.19 | 1.39 |
| Jamaica | 35-39 years | 1.32 | 1.47 | 1.24 |
| Sao Tome and Principe | 35-39 years | 1.33 | 1.91 | 1.13 |
| Nauru | 35-39 years | 1.33 | 1.33 | 1.59 |
| Bolivia (Plurinational State of) | 35-39 years | 1.34 | 1.66 | 1.24 |
| Afghanistan | 35-39 years | 1.35 | 1.48 | 1.12 |
| Palestine | 35-39 years | 1.35 | 1.99 | 1.16 |
| Paraguay | 35-39 years | 1.35 | 1.79 | 1.26 |
| Myanmar | 35-39 years | 1.35 | 1.28 | 1.49 |
| North Macedonia | 35-39 years | 1.36 | 1.39 | 0.86 |
| Iraq | 35-39 years | 1.36 | 1.78 | 1.14 |
| Nicaragua | 35-39 years | 1.36 | 1.70 | 1.32 |
| Guatemala | 35-39 years | 1.36 | 1.85 | 1.35 |
| Marshall Islands | 35-39 years | 1.36 | 1.38 | 1.22 |
| Saint Kitts and Nevis | 35-39 years | 1.36 | 1.34 | 1.63 |
| Brazil | 35-39 years | 1.37 | 1.47 | 1.35 |
| Viet Nam | 35-39 years | 1.37 | 1.37 | 1.26 |
| El Salvador | 35-39 years | 1.38 | 1.50 | 1.38 |
| Democratic People's Republic of Korea | 35-39 years | 1.38 | 1.59 | 1.13 |
| Lao People's Democratic Republic | 35-39 years | 1.39 | 1.44 | 1.32 |
| Libya | 35-39 years | 1.39 | 1.96 | 1.16 |
| Panama | 35-39 years | 1.40 | 1.84 | 1.33 |
| Seychelles | 35-39 years | 1.40 | 1.53 | 1.34 |
| Turkiye | 35-39 years | 1.40 | 1.37 | 1.64 |
| Samoa | 35-39 years | 1.40 | 1.46 | 1.21 |
| American Samoa | 35-39 years | 1.42 | 1.49 | 1.24 |
| Suriname | 35-39 years | 1.42 | 1.74 | 1.25 |
| Algeria | 35-39 years | 1.43 | 2.02 | 1.13 |
| Kuwait | 35-39 years | 1.43 | 1.38 | 1.63 |
| Honduras | 35-39 years | 1.44 | 3.78 | 1.29 |
| Malaysia | 35-39 years | 1.45 | 1.59 | 1.38 |
| Maldives | 35-39 years | 1.47 | 2.21 | 1.28 |
| Barbados | 35-39 years | 1.49 | 2.49 | 1.27 |
| Dominica | 35-39 years | 1.52 | 2.01 | 1.25 |
| Saint Lucia | 35-39 years | 1.53 | 2.00 | 1.30 |
| Philippines | 35-39 years | 1.54 | 1.76 | 1.33 |
| Antigua and Barbuda | 35-39 years | 1.55 | 3.65 | 1.28 |
| Dominican Republic | 35-39 years | 1.56 | 2.17 | 1.27 |
| Tokelau | 35-39 years | 1.57 | 1.57 | 2.06 |
| Bosnia and Herzegovina | 35-39 years | 1.59 | 1.70 | 0.84 |
| Northern Mariana Islands | 35-39 years | 1.61 | 1.88 | 1.25 |
| Morocco | 35-39 years | 1.61 | 2.74 | 1.14 |
| Mauritius | 35-39 years | 1.61 | 2.16 | 1.34 |
| Cambodia | 35-39 years | 1.65 | 2.07 | 1.23 |
| Haiti | 35-39 years | 1.66 | 1.86 | 1.24 |
| Oman | 35-39 years | 1.74 | 1.73 | 1.66 |
| Indonesia | 35-39 years | 1.92 | 2.07 | 1.36 |
| Andorra | 35-39 years | 1.97 | 2.09 | 1.27 |
| Bahrain | 35-39 years | 1.99 | 2.01 | 1.73 |
| Venezuela (Bolivarian Republic of) | 35-39 years | 2.24 | 2.27 | 1.98 |
| Saudi Arabia | 35-39 years | 2.24 | 2.26 | 1.86 |
| Greenland | 35-39 years | 2.53 | 2.89 | 1.42 |
| Poland | 40-44 years | 0.39 | 0.38 | 0.75 |
| Palau | 40-44 years | 0.55 | 0.54 | 1.36 |
| Cook Islands | 40-44 years | 0.57 | 0.55 | 1.11 |
| Monaco | 40-44 years | 0.58 | 0.55 | 1.11 |
| Burkina Faso | 40-44 years | 0.58 | 0.36 | 1.12 |
| Germany | 40-44 years | 0.64 | 0.62 | 1.08 |
| Chad | 40-44 years | 0.65 | 0.46 | 1.12 |
| Benin | 40-44 years | 0.68 | 0.35 | 1.13 |
| Qatar | 40-44 years | 0.68 | 0.59 | 1.56 |
| Ukraine | 40-44 years | 0.70 | 0.66 | 1.05 |
| Russian Federation | 40-44 years | 0.72 | 0.63 | 1.10 |
| Guinea | 40-44 years | 0.74 | 0.57 | 1.11 |
| Republic of Moldova | 40-44 years | 0.75 | 0.72 | 1.06 |
| Romania | 40-44 years | 0.75 | 0.75 | 0.92 |
| Italy | 40-44 years | 0.76 | 0.75 | 0.82 |
| France | 40-44 years | 0.76 | 0.73 | 1.11 |
| Finland | 40-44 years | 0.76 | 0.74 | 1.13 |
| C么te d'Ivoire | 40-44 years | 0.76 | 0.49 | 1.13 |
| Togo | 40-44 years | 0.76 | 0.54 | 1.12 |
| New Zealand | 40-44 years | 0.77 | 0.75 | 1.75 |
| Guinea-Bissau | 40-44 years | 0.78 | 0.65 | 1.11 |
| Sierra Leone | 40-44 years | 0.78 | 0.58 | 1.11 |
| Niger | 40-44 years | 0.78 | 0.60 | 1.12 |
| Cameroon | 40-44 years | 0.79 | 0.55 | 1.11 |
| Georgia | 40-44 years | 0.79 | 0.64 | 1.23 |
| Senegal | 40-44 years | 0.81 | 0.56 | 1.12 |
| Gambia | 40-44 years | 0.81 | 0.59 | 1.12 |
| Cabo Verde | 40-44 years | 0.81 | 0.35 | 1.17 |
| Norway | 40-44 years | 0.81 | 0.80 | 0.86 |
| Liberia | 40-44 years | 0.81 | 0.61 | 1.11 |
| Nigeria | 40-44 years | 0.83 | 0.46 | 1.15 |
| Ghana | 40-44 years | 0.83 | 0.52 | 1.13 |
| Greece | 40-44 years | 0.84 | 0.86 | 0.71 |
| Kazakhstan | 40-44 years | 0.84 | 0.83 | 1.16 |
| Sweden | 40-44 years | 0.86 | 0.77 | 1.54 |
| Bermuda | 40-44 years | 0.86 | 0.82 | 1.44 |
| Austria | 40-44 years | 0.88 | 0.88 | 0.86 |
| Montenegro | 40-44 years | 0.89 | 0.89 | 0.89 |
| Mozambique | 40-44 years | 0.90 | 0.71 | 1.16 |
| Eswatini | 40-44 years | 0.90 | 0.77 | 1.16 |
| Namibia | 40-44 years | 0.91 | 0.70 | 1.17 |
| Latvia | 40-44 years | 0.91 | 0.89 | 1.02 |
| Mongolia | 40-44 years | 0.92 | 0.82 | 1.20 |
| Switzerland | 40-44 years | 0.92 | 0.90 | 1.12 |
| Guam | 40-44 years | 0.92 | 0.79 | 1.28 |
| Denmark | 40-44 years | 0.92 | 0.91 | 1.16 |
| Belarus | 40-44 years | 0.93 | 0.92 | 1.00 |
| Israel | 40-44 years | 0.93 | 0.91 | 1.10 |
| Cyprus | 40-44 years | 0.93 | 0.92 | 1.18 |
| Japan | 40-44 years | 0.93 | 0.91 | 1.08 |
| Mauritania | 40-44 years | 0.94 | 0.77 | 1.08 |
| Lithuania | 40-44 years | 0.94 | 0.93 | 1.03 |
| Hungary | 40-44 years | 0.94 | 0.95 | 0.88 |
| San Marino | 40-44 years | 0.95 | 0.92 | 1.20 |
| Seychelles | 40-44 years | 0.95 | 0.58 | 1.33 |
| Zimbabwe | 40-44 years | 0.96 | 0.91 | 1.15 |
| Portugal | 40-44 years | 0.96 | 0.97 | 0.75 |
| Botswana | 40-44 years | 0.98 | 0.75 | 1.18 |
| Slovenia | 40-44 years | 0.98 | 0.98 | 0.96 |
| Luxembourg | 40-44 years | 0.98 | 0.95 | 1.13 |
| Slovakia | 40-44 years | 0.99 | 1.00 | 0.82 |
| Lesotho | 40-44 years | 0.99 | 0.92 | 1.16 |
| Iceland | 40-44 years | 1.00 | 0.98 | 1.24 |
| Spain | 40-44 years | 1.00 | 0.99 | 1.13 |
| Central African Republic | 40-44 years | 1.01 | 1.01 | 1.02 |
| Uganda | 40-44 years | 1.01 | 0.70 | 1.18 |
| Malawi | 40-44 years | 1.02 | 0.80 | 1.17 |
| Croatia | 40-44 years | 1.03 | 1.03 | 0.92 |
| Gabon | 40-44 years | 1.03 | 1.04 | 1.04 |
| South Sudan | 40-44 years | 1.04 | 0.92 | 1.17 |
| Equatorial Guinea | 40-44 years | 1.04 | 1.17 | 1.01 |
| Democratic Republic of the Congo | 40-44 years | 1.05 | 1.11 | 1.01 |
| Somalia | 40-44 years | 1.05 | 0.96 | 1.17 |
| Burundi | 40-44 years | 1.06 | 0.93 | 1.17 |
| Kyrgyzstan | 40-44 years | 1.06 | 1.00 | 1.19 |
| United Republic of Tanzania | 40-44 years | 1.06 | 0.87 | 1.18 |
| Mali | 40-44 years | 1.06 | 1.03 | 1.11 |
| Iran (Islamic Republic of) | 40-44 years | 1.07 | 0.94 | 1.19 |
| Azerbaijan | 40-44 years | 1.08 | 0.95 | 1.20 |
| Australia | 40-44 years | 1.08 | 1.02 | 2.29 |
| Chile | 40-44 years | 1.08 | 1.02 | 1.45 |
| Madagascar | 40-44 years | 1.09 | 1.06 | 1.16 |
| Taiwan (Province of China) | 40-44 years | 1.09 | 0.93 | 1.67 |
| Angola | 40-44 years | 1.09 | 1.18 | 1.01 |
| China | 40-44 years | 1.10 | 1.07 | 1.13 |
| Uruguay | 40-44 years | 1.10 | 1.04 | 1.38 |
| India | 40-44 years | 1.10 | 1.08 | 1.38 |
| Belgium | 40-44 years | 1.10 | 1.08 | 1.34 |
| United Arab Emirates | 40-44 years | 1.10 | 1.01 | 1.18 |
| Canada | 40-44 years | 1.10 | 1.05 | 1.28 |
| Estonia | 40-44 years | 1.10 | 1.12 | 1.06 |
| Kiribati | 40-44 years | 1.11 | 1.11 | 1.18 |
| Colombia | 40-44 years | 1.12 | 0.95 | 2.01 |
| United States of America | 40-44 years | 1.12 | 1.08 | 1.38 |
| United Kingdom | 40-44 years | 1.12 | 1.12 | 1.14 |
| Tajikistan | 40-44 years | 1.13 | 1.12 | 1.17 |
| Rwanda | 40-44 years | 1.14 | 1.10 | 1.17 |
| Lebanon | 40-44 years | 1.14 | 1.11 | 1.46 |
| Bangladesh | 40-44 years | 1.14 | 1.11 | 1.46 |
| Serbia | 40-44 years | 1.14 | 1.16 | 0.82 |
| Timor-Leste | 40-44 years | 1.15 | 1.09 | 1.29 |
| Sri Lanka | 40-44 years | 1.15 | 1.14 | 1.22 |
| Ethiopia | 40-44 years | 1.15 | 1.03 | 1.23 |
| Albania | 40-44 years | 1.15 | 1.08 | 1.19 |
| Djibouti | 40-44 years | 1.15 | 1.11 | 1.19 |
| Armenia | 40-44 years | 1.16 | 1.12 | 1.21 |
| Netherlands | 40-44 years | 1.16 | 1.17 | 1.10 |
| Guyana | 40-44 years | 1.17 | 1.12 | 1.27 |
| Czechia | 40-44 years | 1.17 | 1.19 | 0.87 |
| Comoros | 40-44 years | 1.17 | 1.25 | 1.16 |
| Kenya | 40-44 years | 1.18 | 1.23 | 1.18 |
| Syrian Arab Republic | 40-44 years | 1.18 | 1.25 | 1.13 |
| Papua New Guinea | 40-44 years | 1.18 | 1.19 | 1.17 |
| South Africa | 40-44 years | 1.19 | 1.26 | 1.17 |
| Zambia | 40-44 years | 1.19 | 1.23 | 1.20 |
| Tunisia | 40-44 years | 1.19 | 1.18 | 1.64 |
| Argentina | 40-44 years | 1.20 | 1.32 | 1.19 |
| Eritrea | 40-44 years | 1.20 | 1.25 | 1.17 |
| Jordan | 40-44 years | 1.20 | 1.17 | 1.56 |
| Bosnia and Herzegovina | 40-44 years | 1.21 | 1.26 | 0.85 |
| Pakistan | 40-44 years | 1.21 | 1.21 | 1.23 |
| Turkmenistan | 40-44 years | 1.21 | 1.23 | 1.18 |
| Bhutan | 40-44 years | 1.22 | 1.21 | 1.28 |
| Solomon Islands | 40-44 years | 1.22 | 1.23 | 1.17 |
| Costa Rica | 40-44 years | 1.23 | 1.03 | 1.32 |
| Republic of Korea | 40-44 years | 1.25 | 1.25 | 1.18 |
| Vanuatu | 40-44 years | 1.27 | 1.27 | 1.21 |
| Malta | 40-44 years | 1.27 | 1.27 | 1.12 |
| Egypt | 40-44 years | 1.27 | 1.36 | 1.13 |
| Singapore | 40-44 years | 1.28 | 1.29 | 1.27 |
| Myanmar | 40-44 years | 1.28 | 1.19 | 1.46 |
| Cuba | 40-44 years | 1.28 | 1.34 | 1.25 |
| Puerto Rico | 40-44 years | 1.28 | 1.26 | 1.65 |
| Palestine | 40-44 years | 1.29 | 1.60 | 1.18 |
| Viet Nam | 40-44 years | 1.30 | 1.31 | 1.09 |
| Afghanistan | 40-44 years | 1.31 | 1.37 | 1.12 |
| Sudan | 40-44 years | 1.31 | 1.39 | 1.18 |
| Bulgaria | 40-44 years | 1.31 | 1.31 | 0.98 |
| Congo | 40-44 years | 1.31 | 1.73 | 1.00 |
| Ecuador | 40-44 years | 1.32 | 1.54 | 1.28 |
| Niue | 40-44 years | 1.32 | 1.31 | 1.53 |
| North Macedonia | 40-44 years | 1.32 | 1.35 | 0.88 |
| Peru | 40-44 years | 1.32 | 1.82 | 1.23 |
| Thailand | 40-44 years | 1.33 | 1.24 | 1.38 |
| Bahamas | 40-44 years | 1.33 | 1.45 | 1.28 |
| Saint Vincent and the Grenadines | 40-44 years | 1.34 | 1.42 | 1.25 |
| Kuwait | 40-44 years | 1.35 | 1.29 | 1.63 |
| Nepal | 40-44 years | 1.35 | 1.37 | 1.13 |
| Guatemala | 40-44 years | 1.35 | 1.84 | 1.33 |
| Lao People's Democratic Republic | 40-44 years | 1.36 | 1.40 | 1.27 |
| Belize | 40-44 years | 1.36 | 1.46 | 1.31 |
| Paraguay | 40-44 years | 1.37 | 1.80 | 1.26 |
| Trinidad and Tobago | 40-44 years | 1.38 | 1.49 | 1.30 |
| Panama | 40-44 years | 1.38 | 1.68 | 1.31 |
| Turkiye | 40-44 years | 1.38 | 1.35 | 1.65 |
| Nicaragua | 40-44 years | 1.39 | 1.83 | 1.32 |
| Sao Tome and Principe | 40-44 years | 1.39 | 1.69 | 1.13 |
| Ireland | 40-44 years | 1.39 | 1.41 | 1.21 |
| Iraq | 40-44 years | 1.40 | 1.65 | 1.16 |
| El Salvador | 40-44 years | 1.40 | 1.54 | 1.38 |
| Saint Kitts and Nevis | 40-44 years | 1.40 | 1.38 | 1.72 |
| Grenada | 40-44 years | 1.41 | 1.55 | 1.21 |
| Malaysia | 40-44 years | 1.41 | 1.52 | 1.36 |
| Brunei Darussalam | 40-44 years | 1.42 | 1.42 | 1.40 |
| Yemen | 40-44 years | 1.42 | 1.63 | 1.13 |
| Brazil | 40-44 years | 1.43 | 1.60 | 1.36 |
| Jamaica | 40-44 years | 1.43 | 1.72 | 1.22 |
| Uzbekistan | 40-44 years | 1.43 | 1.56 | 1.16 |
| Maldives | 40-44 years | 1.43 | 2.17 | 1.24 |
| Bolivia (Plurinational State of) | 40-44 years | 1.47 | 2.08 | 1.21 |
| Democratic People's Republic of Korea | 40-44 years | 1.47 | 1.67 | 1.12 |
| Mexico | 40-44 years | 1.48 | 1.69 | 1.41 |
| Libya | 40-44 years | 1.51 | 2.16 | 1.18 |
| Philippines | 40-44 years | 1.52 | 1.71 | 1.32 |
| Barbados | 40-44 years | 1.52 | 2.41 | 1.26 |
| Algeria | 40-44 years | 1.53 | 2.06 | 1.14 |
| Dominican Republic | 40-44 years | 1.54 | 1.99 | 1.27 |
| Dominica | 40-44 years | 1.56 | 1.99 | 1.24 |
| Tonga | 40-44 years | 1.56 | 1.88 | 1.19 |
| Honduras | 40-44 years | 1.56 | 3.89 | 1.25 |
| Suriname | 40-44 years | 1.59 | 2.10 | 1.24 |
| Greenland | 40-44 years | 1.63 | 1.70 | 1.32 |
| Cambodia | 40-44 years | 1.65 | 2.03 | 1.19 |
| Morocco | 40-44 years | 1.69 | 2.41 | 1.15 |
| Saint Lucia | 40-44 years | 1.70 | 2.21 | 1.30 |
| Fiji | 40-44 years | 1.72 | 1.81 | 1.19 |
| Haiti | 40-44 years | 1.73 | 1.91 | 1.21 |
| Mauritius | 40-44 years | 1.76 | 2.68 | 1.34 |
| Antigua and Barbuda | 40-44 years | 1.76 | 4.17 | 1.27 |
| Micronesia (Federated States of) | 40-44 years | 1.80 | 1.88 | 1.20 |
| Oman | 40-44 years | 1.82 | 1.82 | 1.67 |
| Nauru | 40-44 years | 1.87 | 1.88 | 1.46 |
| Tuvalu | 40-44 years | 1.93 | 1.93 | 1.67 |
| Marshall Islands | 40-44 years | 1.93 | 2.02 | 1.19 |
| Indonesia | 40-44 years | 1.95 | 2.11 | 1.30 |
| Samoa | 40-44 years | 2.04 | 2.24 | 1.18 |
| Tokelau | 40-44 years | 2.17 | 2.17 | 1.90 |
| American Samoa | 40-44 years | 2.18 | 2.49 | 1.23 |
| Venezuela (Bolivarian Republic of) | 40-44 years | 2.26 | 2.31 | 1.89 |
| Saudi Arabia | 40-44 years | 2.30 | 2.33 | 1.89 |
| Andorra | 40-44 years | 2.33 | 2.48 | 1.36 |
| Bahrain | 40-44 years | 2.40 | 2.50 | 1.77 |
| Northern Mariana Islands | 40-44 years | 2.48 | 3.32 | 1.23 |
| United States Virgin Islands | 40-44 years | 3.47 | 3.61 | 1.57 |
| Palau | 45-49 years | 0.31 | 0.30 | 1.24 |
| Poland | 45-49 years | 0.35 | 0.33 | 0.70 |
| Cook Islands | 45-49 years | 0.35 | 0.32 | 0.96 |
| Burkina Faso | 45-49 years | 0.58 | 0.36 | 1.13 |
| Benin | 45-49 years | 0.69 | 0.39 | 1.15 |
| Chad | 45-49 years | 0.71 | 0.55 | 1.12 |
| Ukraine | 45-49 years | 0.71 | 0.66 | 1.24 |
| Solomon Islands | 45-49 years | 0.73 | 0.70 | 1.15 |
| Monaco | 45-49 years | 0.73 | 0.70 | 1.16 |
| Papua New Guinea | 45-49 years | 0.74 | 0.71 | 1.16 |
| Togo | 45-49 years | 0.74 | 0.52 | 1.13 |
| Romania | 45-49 years | 0.74 | 0.72 | 1.01 |
| Italy | 45-49 years | 0.76 | 0.74 | 0.95 |
| C么te d'Ivoire | 45-49 years | 0.76 | 0.49 | 1.15 |
| Kiribati | 45-49 years | 0.76 | 0.73 | 1.17 |
| Guinea-Bissau | 45-49 years | 0.77 | 0.65 | 1.11 |
| Belarus | 45-49 years | 0.77 | 0.72 | 1.17 |
| Qatar | 45-49 years | 0.77 | 0.67 | 1.51 |
| Guinea | 45-49 years | 0.77 | 0.62 | 1.11 |
| Bermuda | 45-49 years | 0.78 | 0.74 | 1.38 |
| Vanuatu | 45-49 years | 0.78 | 0.75 | 1.20 |
| Germany | 45-49 years | 0.79 | 0.78 | 1.11 |
| Sierra Leone | 45-49 years | 0.79 | 0.59 | 1.11 |
| Georgia | 45-49 years | 0.79 | 0.69 | 1.27 |
| Niue | 45-49 years | 0.80 | 0.78 | 1.34 |
| Cabo Verde | 45-49 years | 0.80 | 0.34 | 1.21 |
| Cameroon | 45-49 years | 0.81 | 0.57 | 1.12 |
| Russian Federation | 45-49 years | 0.82 | 0.70 | 1.27 |
| Senegal | 45-49 years | 0.82 | 0.59 | 1.13 |
| Niger | 45-49 years | 0.82 | 0.67 | 1.12 |
| Guam | 45-49 years | 0.83 | 0.66 | 1.29 |
| Gambia | 45-49 years | 0.83 | 0.62 | 1.13 |
| Ghana | 45-49 years | 0.83 | 0.52 | 1.14 |
| France | 45-49 years | 0.84 | 0.81 | 1.15 |
| Nigeria | 45-49 years | 0.84 | 0.49 | 1.16 |
| Republic of Moldova | 45-49 years | 0.85 | 0.80 | 1.22 |
| Liberia | 45-49 years | 0.87 | 0.72 | 1.12 |
| Japan | 45-49 years | 0.88 | 0.83 | 1.18 |
| Mongolia | 45-49 years | 0.89 | 0.82 | 1.22 |
| Slovakia | 45-49 years | 0.89 | 0.89 | 0.88 |
| Hungary | 45-49 years | 0.90 | 0.89 | 0.99 |
| United States Virgin Islands | 45-49 years | 0.92 | 0.88 | 1.47 |
| Slovenia | 45-49 years | 0.93 | 0.91 | 1.07 |
| Israel | 45-49 years | 0.94 | 0.91 | 1.20 |
| Greece | 45-49 years | 0.94 | 0.96 | 0.79 |
| Mozambique | 45-49 years | 0.94 | 0.85 | 1.17 |
| Montenegro | 45-49 years | 0.94 | 0.94 | 0.99 |
| Namibia | 45-49 years | 0.94 | 0.80 | 1.19 |
| Czechia | 45-49 years | 0.95 | 0.94 | 0.97 |
| Denmark | 45-49 years | 0.95 | 0.94 | 1.23 |
| Kyrgyzstan | 45-49 years | 0.96 | 0.89 | 1.22 |
| Finland | 45-49 years | 0.97 | 0.95 | 1.19 |
| Mauritania | 45-49 years | 0.98 | 0.89 | 1.06 |
| Austria | 45-49 years | 0.98 | 0.99 | 0.89 |
| New Zealand | 45-49 years | 0.99 | 0.97 | 1.68 |
| Fiji | 45-49 years | 0.99 | 0.97 | 1.18 |
| Eswatini | 45-49 years | 0.99 | 0.91 | 1.17 |
| Uganda | 45-49 years | 1.03 | 0.84 | 1.20 |
| Botswana | 45-49 years | 1.03 | 0.88 | 1.20 |
| Uruguay | 45-49 years | 1.03 | 0.98 | 1.34 |
| Nauru | 45-49 years | 1.04 | 1.03 | 1.30 |
| Portugal | 45-49 years | 1.04 | 1.04 | 0.87 |
| United Arab Emirates | 45-49 years | 1.05 | 0.88 | 1.20 |
| Cyprus | 45-49 years | 1.05 | 1.04 | 1.37 |
| Norway | 45-49 years | 1.05 | 1.08 | 0.90 |
| Kazakhstan | 45-49 years | 1.06 | 1.05 | 1.19 |
| Lesotho | 45-49 years | 1.06 | 1.03 | 1.17 |
| Chile | 45-49 years | 1.06 | 1.00 | 1.42 |
| Iran (Islamic Republic of) | 45-49 years | 1.06 | 0.92 | 1.22 |
| Malawi | 45-49 years | 1.06 | 0.97 | 1.19 |
| Micronesia (Federated States of) | 45-49 years | 1.06 | 1.05 | 1.19 |
| Mali | 45-49 years | 1.07 | 1.06 | 1.11 |
| Spain | 45-49 years | 1.07 | 1.06 | 1.30 |
| Iceland | 45-49 years | 1.08 | 1.06 | 1.22 |
| Seychelles | 45-49 years | 1.08 | 0.70 | 1.35 |
| Saint Vincent and the Grenadines | 45-49 years | 1.10 | 1.02 | 1.25 |
| Switzerland | 45-49 years | 1.11 | 1.09 | 1.16 |
| Tunisia | 45-49 years | 1.11 | 1.09 | 1.56 |
| Sri Lanka | 45-49 years | 1.11 | 1.14 | 0.95 |
| Serbia | 45-49 years | 1.11 | 1.13 | 0.92 |
| Canada | 45-49 years | 1.12 | 1.09 | 1.18 |
| United States of America | 45-49 years | 1.12 | 1.10 | 1.24 |
| South Sudan | 45-49 years | 1.12 | 1.09 | 1.18 |
| Croatia | 45-49 years | 1.12 | 1.13 | 0.98 |
| Estonia | 45-49 years | 1.12 | 1.06 | 1.32 |
| Tonga | 45-49 years | 1.13 | 1.09 | 1.19 |
| Luxembourg | 45-49 years | 1.13 | 1.10 | 1.18 |
| Marshall Islands | 45-49 years | 1.13 | 1.13 | 1.18 |
| United Kingdom | 45-49 years | 1.13 | 1.13 | 1.19 |
| Burundi | 45-49 years | 1.14 | 1.12 | 1.17 |
| Azerbaijan | 45-49 years | 1.14 | 1.08 | 1.23 |
| Zimbabwe | 45-49 years | 1.14 | 1.14 | 1.15 |
| Lebanon | 45-49 years | 1.14 | 1.12 | 1.38 |
| Tuvalu | 45-49 years | 1.14 | 1.13 | 1.55 |
| United Republic of Tanzania | 45-49 years | 1.15 | 1.11 | 1.19 |
| Bangladesh | 45-49 years | 1.15 | 1.13 | 1.44 |
| Gabon | 45-49 years | 1.15 | 1.27 | 1.06 |
| Equatorial Guinea | 45-49 years | 1.16 | 1.42 | 1.02 |
| Bulgaria | 45-49 years | 1.16 | 1.16 | 1.06 |
| Albania | 45-49 years | 1.16 | 1.11 | 1.21 |
| Egypt | 45-49 years | 1.17 | 1.18 | 1.16 |
| Lithuania | 45-49 years | 1.18 | 1.17 | 1.23 |
| Tajikistan | 45-49 years | 1.18 | 1.18 | 1.18 |
| Somalia | 45-49 years | 1.18 | 1.19 | 1.17 |
| Central African Republic | 45-49 years | 1.18 | 1.23 | 1.02 |
| Netherlands | 45-49 years | 1.18 | 1.19 | 1.15 |
| Sweden | 45-49 years | 1.19 | 1.12 | 1.59 |
| Costa Rica | 45-49 years | 1.19 | 0.95 | 1.32 |
| China | 45-49 years | 1.19 | 1.21 | 1.15 |
| Panama | 45-49 years | 1.19 | 0.99 | 1.30 |
| Syrian Arab Republic | 45-49 years | 1.20 | 1.26 | 1.15 |
| Taiwan (Province of China) | 45-49 years | 1.20 | 1.07 | 1.57 |
| Australia | 45-49 years | 1.20 | 1.14 | 2.15 |
| Madagascar | 45-49 years | 1.21 | 1.23 | 1.16 |
| Afghanistan | 45-49 years | 1.22 | 1.24 | 1.12 |
| San Marino | 45-49 years | 1.22 | 1.20 | 1.33 |
| Grenada | 45-49 years | 1.22 | 1.22 | 1.22 |
| Samoa | 45-49 years | 1.22 | 1.24 | 1.17 |
| Guyana | 45-49 years | 1.23 | 1.21 | 1.28 |
| Rwanda | 45-49 years | 1.23 | 1.32 | 1.17 |
| Zambia | 45-49 years | 1.24 | 1.30 | 1.20 |
| Ethiopia | 45-49 years | 1.24 | 1.27 | 1.24 |
| Republic of Korea | 45-49 years | 1.24 | 1.20 | 1.33 |
| Angola | 45-49 years | 1.25 | 1.40 | 1.02 |
| Argentina | 45-49 years | 1.25 | 1.40 | 1.22 |
| Tokelau | 45-49 years | 1.25 | 1.24 | 1.71 |
| Democratic Republic of the Congo | 45-49 years | 1.25 | 1.44 | 1.01 |
| Saint Kitts and Nevis | 45-49 years | 1.26 | 1.23 | 1.72 |
| Djibouti | 45-49 years | 1.27 | 1.39 | 1.19 |
| Belize | 45-49 years | 1.28 | 1.27 | 1.31 |
| South Africa | 45-49 years | 1.28 | 1.42 | 1.18 |
| Armenia | 45-49 years | 1.29 | 1.36 | 1.23 |
| Comoros | 45-49 years | 1.30 | 1.51 | 1.16 |
| Viet Nam | 45-49 years | 1.30 | 1.37 | 0.90 |
| Iraq | 45-49 years | 1.31 | 1.42 | 1.18 |
| Latvia | 45-49 years | 1.32 | 1.34 | 1.22 |
| Palestine | 45-49 years | 1.32 | 1.60 | 1.19 |
| Turkmenistan | 45-49 years | 1.33 | 1.37 | 1.20 |
| Myanmar | 45-49 years | 1.33 | 1.27 | 1.45 |
| Suriname | 45-49 years | 1.33 | 1.44 | 1.25 |
| Sudan | 45-49 years | 1.34 | 1.41 | 1.19 |
| Kenya | 45-49 years | 1.34 | 1.66 | 1.19 |
| Belgium | 45-49 years | 1.34 | 1.34 | 1.37 |
| Turkiye | 45-49 years | 1.36 | 1.33 | 1.60 |
| Paraguay | 45-49 years | 1.36 | 1.61 | 1.26 |
| Guatemala | 45-49 years | 1.37 | 1.94 | 1.33 |
| Uzbekistan | 45-49 years | 1.37 | 1.42 | 1.18 |
| Eritrea | 45-49 years | 1.37 | 1.52 | 1.18 |
| Cuba | 45-49 years | 1.37 | 1.52 | 1.25 |
| Kuwait | 45-49 years | 1.37 | 1.32 | 1.56 |
| Nicaragua | 45-49 years | 1.38 | 1.62 | 1.33 |
| Bosnia and Herzegovina | 45-49 years | 1.38 | 1.44 | 0.95 |
| Brunei Darussalam | 45-49 years | 1.38 | 1.36 | 1.72 |
| Peru | 45-49 years | 1.39 | 2.09 | 1.23 |
| American Samoa | 45-49 years | 1.39 | 1.44 | 1.23 |
| Northern Mariana Islands | 45-49 years | 1.39 | 1.44 | 1.23 |
| Thailand | 45-49 years | 1.39 | 1.42 | 1.39 |
| Colombia | 45-49 years | 1.40 | 1.29 | 1.85 |
| Bahamas | 45-49 years | 1.40 | 1.61 | 1.29 |
| Ecuador | 45-49 years | 1.41 | 1.93 | 1.29 |
| Pakistan | 45-49 years | 1.41 | 1.43 | 1.21 |
| India | 45-49 years | 1.41 | 1.42 | 1.37 |
| Yemen | 45-49 years | 1.42 | 1.56 | 1.15 |
| North Macedonia | 45-49 years | 1.42 | 1.45 | 0.99 |
| Timor-Leste | 45-49 years | 1.42 | 1.49 | 1.26 |
| Jordan | 45-49 years | 1.43 | 1.43 | 1.50 |
| El Salvador | 45-49 years | 1.44 | 1.65 | 1.40 |
| Malta | 45-49 years | 1.45 | 1.45 | 1.22 |
| Jamaica | 45-49 years | 1.45 | 1.74 | 1.21 |
| Oman | 45-49 years | 1.45 | 1.43 | 1.64 |
| Bhutan | 45-49 years | 1.45 | 1.48 | 1.26 |
| Sao Tome and Principe | 45-49 years | 1.46 | 1.80 | 1.13 |
| Libya | 45-49 years | 1.47 | 1.94 | 1.19 |
| Trinidad and Tobago | 45-49 years | 1.47 | 1.68 | 1.30 |
| Lao People's Democratic Republic | 45-49 years | 1.48 | 1.63 | 1.25 |
| Puerto Rico | 45-49 years | 1.48 | 1.46 | 1.60 |
| Algeria | 45-49 years | 1.49 | 1.91 | 1.14 |
| Barbados | 45-49 years | 1.50 | 2.18 | 1.26 |
| Maldives | 45-49 years | 1.51 | 2.92 | 1.21 |
| Congo | 45-49 years | 1.51 | 1.98 | 1.00 |
| Philippines | 45-49 years | 1.51 | 1.70 | 1.32 |
| Malaysia | 45-49 years | 1.52 | 1.79 | 1.36 |
| Dominican Republic | 45-49 years | 1.53 | 1.95 | 1.27 |
| Dominica | 45-49 years | 1.53 | 1.86 | 1.24 |
| Nepal | 45-49 years | 1.55 | 1.59 | 1.11 |
| Brazil | 45-49 years | 1.56 | 1.86 | 1.38 |
| Singapore | 45-49 years | 1.58 | 1.57 | 1.45 |
| Mexico | 45-49 years | 1.58 | 1.82 | 1.43 |
| Greenland | 45-49 years | 1.59 | 1.67 | 1.22 |
| Cambodia | 45-49 years | 1.60 | 1.91 | 1.17 |
| Ireland | 45-49 years | 1.62 | 1.64 | 1.31 |
| Democratic People's Republic of Korea | 45-49 years | 1.63 | 1.80 | 1.12 |
| Honduras | 45-49 years | 1.69 | 3.54 | 1.23 |
| Morocco | 45-49 years | 1.69 | 2.22 | 1.16 |
| Saint Lucia | 45-49 years | 1.70 | 2.13 | 1.30 |
| Bolivia (Plurinational State of) | 45-49 years | 1.71 | 2.68 | 1.20 |
| Mauritius | 45-49 years | 1.71 | 2.54 | 1.35 |
| Antigua and Barbuda | 45-49 years | 1.72 | 3.24 | 1.27 |
| Venezuela (Bolivarian Republic of) | 45-49 years | 1.72 | 1.71 | 1.73 |
| Haiti | 45-49 years | 1.80 | 1.98 | 1.19 |
| Indonesia | 45-49 years | 2.03 | 2.26 | 1.19 |
| Bahrain | 45-49 years | 2.33 | 2.49 | 1.75 |
| Saudi Arabia | 45-49 years | 2.42 | 2.47 | 1.83 |
| Andorra | 45-49 years | 2.78 | 3.03 | 1.56 |
| Palau | 50-54 years | 0.29 | 0.28 | 1.17 |
| Cook Islands | 50-54 years | 0.32 | 0.29 | 0.85 |
| Poland | 50-54 years | 0.50 | 0.49 | 0.71 |
| Solomon Islands | 50-54 years | 0.64 | 0.62 | 1.15 |
| Romania | 50-54 years | 0.66 | 0.63 | 1.10 |
| Kiribati | 50-54 years | 0.68 | 0.65 | 1.19 |
| Papua New Guinea | 50-54 years | 0.68 | 0.66 | 1.16 |
| Vanuatu | 50-54 years | 0.69 | 0.66 | 1.22 |
| Niue | 50-54 years | 0.70 | 0.68 | 1.22 |
| Burkina Faso | 50-54 years | 0.70 | 0.58 | 1.14 |
| Belarus | 50-54 years | 0.71 | 0.63 | 1.32 |
| Germany | 50-54 years | 0.74 | 0.71 | 1.14 |
| Norway | 50-54 years | 0.76 | 0.72 | 0.94 |
| Benin | 50-54 years | 0.79 | 0.60 | 1.16 |
| Greece | 50-54 years | 0.80 | 0.79 | 0.89 |
| Bermuda | 50-54 years | 0.82 | 0.77 | 1.32 |
| Qatar | 50-54 years | 0.83 | 0.73 | 1.45 |
| Nauru | 50-54 years | 0.85 | 0.85 | 1.19 |
| Czechia | 50-54 years | 0.87 | 0.84 | 1.06 |
| Monaco | 50-54 years | 0.87 | 0.83 | 1.21 |
| Italy | 50-54 years | 0.88 | 0.85 | 1.09 |
| Cabo Verde | 50-54 years | 0.88 | 0.54 | 1.26 |
| Japan | 50-54 years | 0.88 | 0.80 | 1.34 |
| Mongolia | 50-54 years | 0.89 | 0.84 | 1.24 |
| Ukraine | 50-54 years | 0.90 | 0.84 | 1.42 |
| Georgia | 50-54 years | 0.90 | 0.84 | 1.33 |
| Fiji | 50-54 years | 0.90 | 0.88 | 1.19 |
| Kazakhstan | 50-54 years | 0.92 | 0.89 | 1.24 |
| Micronesia (Federated States of) | 50-54 years | 0.92 | 0.90 | 1.19 |
| Marshall Islands | 50-54 years | 0.95 | 0.93 | 1.18 |
| Portugal | 50-54 years | 0.95 | 0.95 | 1.01 |
| C么te d'Ivoire | 50-54 years | 0.95 | 0.84 | 1.17 |
| Namibia | 50-54 years | 0.96 | 0.85 | 1.21 |
| Chad | 50-54 years | 0.96 | 0.92 | 1.13 |
| Guam | 50-54 years | 0.97 | 0.86 | 1.30 |
| Ghana | 50-54 years | 0.97 | 0.83 | 1.15 |
| Togo | 50-54 years | 0.98 | 0.91 | 1.15 |
| France | 50-54 years | 0.98 | 0.95 | 1.19 |
| Montenegro | 50-54 years | 0.98 | 0.98 | 1.07 |
| Denmark | 50-54 years | 0.98 | 0.97 | 1.30 |
| Nigeria | 50-54 years | 1.00 | 0.86 | 1.17 |
| Tuvalu | 50-54 years | 1.00 | 0.99 | 1.49 |
| Hungary | 50-54 years | 1.00 | 1.00 | 1.08 |
| Cameroon | 50-54 years | 1.00 | 0.93 | 1.13 |
| Russian Federation | 50-54 years | 1.01 | 0.90 | 1.43 |
| United Arab Emirates | 50-54 years | 1.01 | 0.86 | 1.21 |
| Slovakia | 50-54 years | 1.01 | 1.03 | 0.93 |
| Israel | 50-54 years | 1.02 | 1.00 | 1.28 |
| Slovenia | 50-54 years | 1.03 | 1.02 | 1.16 |
| Sierra Leone | 50-54 years | 1.03 | 1.00 | 1.12 |
| Senegal | 50-54 years | 1.04 | 0.99 | 1.14 |
| Saint Vincent and the Grenadines | 50-54 years | 1.04 | 0.95 | 1.27 |
| Guinea-Bissau | 50-54 years | 1.04 | 1.02 | 1.12 |
| Eswatini | 50-54 years | 1.04 | 0.99 | 1.19 |
| Iceland | 50-54 years | 1.05 | 1.03 | 1.19 |
| Switzerland | 50-54 years | 1.05 | 1.03 | 1.20 |
| Croatia | 50-54 years | 1.06 | 1.06 | 1.08 |
| Guinea | 50-54 years | 1.06 | 1.05 | 1.11 |
| Lesotho | 50-54 years | 1.07 | 1.04 | 1.18 |
| Finland | 50-54 years | 1.07 | 1.05 | 1.27 |
| Mozambique | 50-54 years | 1.07 | 1.05 | 1.20 |
| Viet Nam | 50-54 years | 1.07 | 1.14 | 0.78 |
| Botswana | 50-54 years | 1.08 | 0.97 | 1.24 |
| Sri Lanka | 50-54 years | 1.08 | 1.15 | 0.77 |
| Tokelau | 50-54 years | 1.09 | 1.07 | 1.58 |
| Tonga | 50-54 years | 1.09 | 1.04 | 1.21 |
| Seychelles | 50-54 years | 1.10 | 0.73 | 1.39 |
| Taiwan (Province of China) | 50-54 years | 1.10 | 0.93 | 1.50 |
| Uganda | 50-54 years | 1.11 | 1.04 | 1.23 |
| Samoa | 50-54 years | 1.11 | 1.10 | 1.17 |
| Iran (Islamic Republic of) | 50-54 years | 1.11 | 1.02 | 1.25 |
| Niger | 50-54 years | 1.11 | 1.12 | 1.12 |
| Luxembourg | 50-54 years | 1.12 | 1.10 | 1.25 |
| Republic of Korea | 50-54 years | 1.12 | 1.03 | 1.49 |
| Canada | 50-54 years | 1.13 | 1.13 | 1.09 |
| Gambia | 50-54 years | 1.14 | 1.15 | 1.15 |
| Tunisia | 50-54 years | 1.14 | 1.12 | 1.47 |
| Bulgaria | 50-54 years | 1.14 | 1.14 | 1.11 |
| Costa Rica | 50-54 years | 1.14 | 0.89 | 1.35 |
| Saint Kitts and Nevis | 50-54 years | 1.15 | 1.11 | 1.69 |
| Austria | 50-54 years | 1.15 | 1.16 | 1.00 |
| Spain | 50-54 years | 1.16 | 1.14 | 1.45 |
| Zimbabwe | 50-54 years | 1.17 | 1.17 | 1.17 |
| Serbia | 50-54 years | 1.17 | 1.18 | 1.00 |
| Tajikistan | 50-54 years | 1.17 | 1.17 | 1.19 |
| Gabon | 50-54 years | 1.18 | 1.26 | 1.07 |
| Malawi | 50-54 years | 1.18 | 1.19 | 1.20 |
| Lebanon | 50-54 years | 1.19 | 1.18 | 1.30 |
| New Zealand | 50-54 years | 1.21 | 1.19 | 1.68 |
| Central African Republic | 50-54 years | 1.21 | 1.25 | 1.03 |
| Albania | 50-54 years | 1.21 | 1.19 | 1.23 |
| Australia | 50-54 years | 1.22 | 1.15 | 1.98 |
| Liberia | 50-54 years | 1.22 | 1.30 | 1.11 |
| United Kingdom | 50-54 years | 1.22 | 1.22 | 1.25 |
| Azerbaijan | 50-54 years | 1.23 | 1.22 | 1.27 |
| Kyrgyzstan | 50-54 years | 1.24 | 1.24 | 1.26 |
| Panama | 50-54 years | 1.24 | 1.15 | 1.31 |
| Guyana | 50-54 years | 1.25 | 1.24 | 1.29 |
| Iraq | 50-54 years | 1.25 | 1.29 | 1.19 |
| Argentina | 50-54 years | 1.25 | 1.27 | 1.26 |
| United States of America | 50-54 years | 1.26 | 1.29 | 1.12 |
| Lithuania | 50-54 years | 1.26 | 1.23 | 1.39 |
| Egypt | 50-54 years | 1.27 | 1.30 | 1.18 |
| Equatorial Guinea | 50-54 years | 1.27 | 1.52 | 1.02 |
| American Samoa | 50-54 years | 1.28 | 1.29 | 1.24 |
| China | 50-54 years | 1.28 | 1.31 | 1.20 |
| Mauritania | 50-54 years | 1.29 | 1.58 | 1.04 |
| United Republic of Tanzania | 50-54 years | 1.30 | 1.38 | 1.21 |
| Uruguay | 50-54 years | 1.30 | 1.30 | 1.30 |
| Netherlands | 50-54 years | 1.30 | 1.31 | 1.20 |
| Syrian Arab Republic | 50-54 years | 1.31 | 1.41 | 1.17 |
| Myanmar | 50-54 years | 1.31 | 1.25 | 1.47 |
| Grenada | 50-54 years | 1.32 | 1.35 | 1.25 |
| North Macedonia | 50-54 years | 1.33 | 1.35 | 1.09 |
| South Sudan | 50-54 years | 1.33 | 1.38 | 1.19 |
| Kuwait | 50-54 years | 1.33 | 1.30 | 1.47 |
| Afghanistan | 50-54 years | 1.33 | 1.37 | 1.12 |
| Mali | 50-54 years | 1.34 | 1.54 | 1.10 |
| Burundi | 50-54 years | 1.34 | 1.41 | 1.18 |
| India | 50-54 years | 1.34 | 1.35 | 1.38 |
| San Marino | 50-54 years | 1.34 | 1.32 | 1.45 |
| Republic of Moldova | 50-54 years | 1.34 | 1.34 | 1.36 |
| Uzbekistan | 50-54 years | 1.35 | 1.37 | 1.20 |
| South Africa | 50-54 years | 1.35 | 1.49 | 1.21 |
| Angola | 50-54 years | 1.37 | 1.51 | 1.02 |
| Cyprus | 50-54 years | 1.37 | 1.36 | 1.54 |
| Thailand | 50-54 years | 1.37 | 1.30 | 1.42 |
| Zambia | 50-54 years | 1.38 | 1.49 | 1.22 |
| Bosnia and Herzegovina | 50-54 years | 1.38 | 1.42 | 1.06 |
| Trinidad and Tobago | 50-54 years | 1.39 | 1.47 | 1.31 |
| Colombia | 50-54 years | 1.39 | 1.30 | 1.69 |
| Paraguay | 50-54 years | 1.39 | 1.56 | 1.28 |
| Bangladesh | 50-54 years | 1.39 | 1.39 | 1.45 |
| Turkiye | 50-54 years | 1.40 | 1.38 | 1.54 |
| Turkmenistan | 50-54 years | 1.40 | 1.44 | 1.23 |
| Armenia | 50-54 years | 1.40 | 1.48 | 1.28 |
| Bahamas | 50-54 years | 1.41 | 1.55 | 1.31 |
| Philippines | 50-54 years | 1.41 | 1.47 | 1.34 |
| Sweden | 50-54 years | 1.41 | 1.37 | 1.60 |
| Latvia | 50-54 years | 1.42 | 1.42 | 1.39 |
| Estonia | 50-54 years | 1.42 | 1.38 | 1.54 |
| Bhutan | 50-54 years | 1.42 | 1.43 | 1.25 |
| Suriname | 50-54 years | 1.42 | 1.56 | 1.28 |
| Democratic Republic of the Congo | 50-54 years | 1.42 | 1.61 | 1.02 |
| Chile | 50-54 years | 1.43 | 1.43 | 1.39 |
| Somalia | 50-54 years | 1.43 | 1.51 | 1.18 |
| Madagascar | 50-54 years | 1.43 | 1.49 | 1.16 |
| Guatemala | 50-54 years | 1.43 | 1.98 | 1.34 |
| Peru | 50-54 years | 1.43 | 1.96 | 1.24 |
| Cuba | 50-54 years | 1.44 | 1.59 | 1.28 |
| Rwanda | 50-54 years | 1.45 | 1.62 | 1.19 |
| Jordan | 50-54 years | 1.45 | 1.47 | 1.42 |
| Ethiopia | 50-54 years | 1.45 | 1.59 | 1.24 |
| Malta | 50-54 years | 1.46 | 1.46 | 1.32 |
| Palestine | 50-54 years | 1.46 | 1.78 | 1.21 |
| Ecuador | 50-54 years | 1.46 | 1.88 | 1.31 |
| Belgium | 50-54 years | 1.46 | 1.47 | 1.42 |
| Djibouti | 50-54 years | 1.47 | 1.66 | 1.21 |
| Yemen | 50-54 years | 1.47 | 1.59 | 1.16 |
| Sudan | 50-54 years | 1.49 | 1.59 | 1.21 |
| Nicaragua | 50-54 years | 1.49 | 1.82 | 1.35 |
| Venezuela (Bolivarian Republic of) | 50-54 years | 1.50 | 1.49 | 1.60 |
| Greenland | 50-54 years | 1.50 | 1.58 | 1.15 |
| Brunei Darussalam | 50-54 years | 1.50 | 1.47 | 2.01 |
| Malaysia | 50-54 years | 1.51 | 1.69 | 1.38 |
| Algeria | 50-54 years | 1.51 | 1.82 | 1.14 |
| Jamaica | 50-54 years | 1.51 | 1.80 | 1.20 |
| Cambodia | 50-54 years | 1.53 | 1.69 | 1.17 |
| Comoros | 50-54 years | 1.53 | 1.84 | 1.16 |
| Kenya | 50-54 years | 1.54 | 1.91 | 1.20 |
| Maldives | 50-54 years | 1.54 | 2.66 | 1.20 |
| Dominica | 50-54 years | 1.54 | 1.75 | 1.26 |
| El Salvador | 50-54 years | 1.54 | 1.84 | 1.43 |
| Lao People's Democratic Republic | 50-54 years | 1.54 | 1.69 | 1.25 |
| Pakistan | 50-54 years | 1.55 | 1.58 | 1.21 |
| Eritrea | 50-54 years | 1.56 | 1.71 | 1.20 |
| Libya | 50-54 years | 1.57 | 2.06 | 1.19 |
| Northern Mariana Islands | 50-54 years | 1.58 | 1.75 | 1.24 |
| Dominican Republic | 50-54 years | 1.58 | 1.96 | 1.29 |
| Brazil | 50-54 years | 1.59 | 1.78 | 1.41 |
| Barbados | 50-54 years | 1.62 | 2.45 | 1.27 |
| Puerto Rico | 50-54 years | 1.62 | 1.62 | 1.54 |
| Nepal | 50-54 years | 1.64 | 1.67 | 1.11 |
| Singapore | 50-54 years | 1.65 | 1.66 | 1.60 |
| Timor-Leste | 50-54 years | 1.66 | 1.82 | 1.25 |
| Mexico | 50-54 years | 1.66 | 1.81 | 1.47 |
| Congo | 50-54 years | 1.68 | 2.06 | 1.00 |
| Belize | 50-54 years | 1.68 | 2.23 | 1.31 |
| Democratic People's Republic of Korea | 50-54 years | 1.68 | 1.79 | 1.13 |
| Ireland | 50-54 years | 1.71 | 1.74 | 1.41 |
| Mauritius | 50-54 years | 1.71 | 2.33 | 1.39 |
| Saint Lucia | 50-54 years | 1.75 | 2.14 | 1.31 |
| Morocco | 50-54 years | 1.75 | 2.13 | 1.16 |
| Oman | 50-54 years | 1.80 | 1.83 | 1.61 |
| Antigua and Barbuda | 50-54 years | 1.84 | 3.03 | 1.29 |
| Bolivia (Plurinational State of) | 50-54 years | 1.85 | 2.61 | 1.20 |
| Indonesia | 50-54 years | 1.94 | 2.15 | 1.06 |
| Haiti | 50-54 years | 1.96 | 2.14 | 1.19 |
| Sao Tome and Principe | 50-54 years | 1.99 | 2.66 | 1.13 |
| United States Virgin Islands | 50-54 years | 2.03 | 2.17 | 1.38 |
| Honduras | 50-54 years | 2.12 | 4.17 | 1.23 |
| Bahrain | 50-54 years | 2.35 | 2.56 | 1.71 |
| Andorra | 50-54 years | 3.03 | 3.29 | 1.79 |
| Saudi Arabia | 50-54 years | 3.03 | 3.24 | 1.75 |
| Palau | 55-59 years | 0.48 | 0.45 | 1.14 |
| Cook Islands | 55-59 years | 0.51 | 0.47 | 0.80 |
| Romania | 55-59 years | 0.59 | 0.55 | 1.14 |
| Poland | 55-59 years | 0.61 | 0.60 | 0.74 |
| Bermuda | 55-59 years | 0.71 | 0.65 | 1.28 |
| Germany | 55-59 years | 0.72 | 0.69 | 1.17 |
| United Arab Emirates | 55-59 years | 0.75 | 0.61 | 1.22 |
| Namibia | 55-59 years | 0.80 | 0.69 | 1.23 |
| Kazakhstan | 55-59 years | 0.81 | 0.78 | 1.28 |
| Norway | 55-59 years | 0.82 | 0.80 | 1.00 |
| Mongolia | 55-59 years | 0.83 | 0.79 | 1.26 |
| Georgia | 55-59 years | 0.84 | 0.79 | 1.38 |
| Slovenia | 55-59 years | 0.84 | 0.81 | 1.17 |
| Portugal | 55-59 years | 0.85 | 0.83 | 1.14 |
| Eswatini | 55-59 years | 0.89 | 0.83 | 1.21 |
| Lesotho | 55-59 years | 0.89 | 0.85 | 1.20 |
| Botswana | 55-59 years | 0.92 | 0.79 | 1.26 |
| France | 55-59 years | 0.92 | 0.89 | 1.21 |
| Greece | 55-59 years | 0.92 | 0.91 | 1.05 |
| Qatar | 55-59 years | 0.93 | 0.87 | 1.43 |
| Czechia | 55-59 years | 0.93 | 0.91 | 1.10 |
| Iceland | 55-59 years | 0.93 | 0.92 | 1.13 |
| Japan | 55-59 years | 0.95 | 0.83 | 1.62 |
| Italy | 55-59 years | 0.96 | 0.93 | 1.15 |
| Seychelles | 55-59 years | 0.96 | 0.61 | 1.43 |
| Slovakia | 55-59 years | 0.98 | 0.98 | 0.95 |
| Sri Lanka | 55-59 years | 0.99 | 1.06 | 0.72 |
| Zimbabwe | 55-59 years | 0.99 | 0.98 | 1.18 |
| Monaco | 55-59 years | 0.99 | 0.96 | 1.25 |
| Cabo Verde | 55-59 years | 1.00 | 0.84 | 1.31 |
| Viet Nam | 55-59 years | 1.02 | 1.09 | 0.79 |
| Papua New Guinea | 55-59 years | 1.02 | 1.01 | 1.17 |
| Solomon Islands | 55-59 years | 1.02 | 1.01 | 1.16 |
| Burkina Faso | 55-59 years | 1.02 | 1.00 | 1.16 |
| Israel | 55-59 years | 1.03 | 1.01 | 1.36 |
| Hungary | 55-59 years | 1.04 | 1.03 | 1.11 |
| Guam | 55-59 years | 1.05 | 0.97 | 1.32 |
| Benin | 55-59 years | 1.06 | 1.03 | 1.17 |
| Belarus | 55-59 years | 1.06 | 1.00 | 1.43 |
| Uganda | 55-59 years | 1.06 | 1.01 | 1.25 |
| Austria | 55-59 years | 1.07 | 1.05 | 1.15 |
| Vanuatu | 55-59 years | 1.07 | 1.06 | 1.23 |
| Croatia | 55-59 years | 1.07 | 1.06 | 1.23 |
| Finland | 55-59 years | 1.08 | 1.06 | 1.32 |
| Kyrgyzstan | 55-59 years | 1.08 | 1.06 | 1.30 |
| Saint Vincent and the Grenadines | 55-59 years | 1.08 | 1.02 | 1.28 |
| Switzerland | 55-59 years | 1.10 | 1.07 | 1.24 |
| Tunisia | 55-59 years | 1.10 | 1.08 | 1.41 |
| Mozambique | 55-59 years | 1.10 | 1.08 | 1.22 |
| Ukraine | 55-59 years | 1.10 | 1.05 | 1.55 |
| Montenegro | 55-59 years | 1.10 | 1.10 | 1.09 |
| Serbia | 55-59 years | 1.11 | 1.12 | 1.02 |
| Denmark | 55-59 years | 1.12 | 1.11 | 1.36 |
| Saint Kitts and Nevis | 55-59 years | 1.14 | 1.10 | 1.64 |
| Iran (Islamic Republic of) | 55-59 years | 1.14 | 1.10 | 1.27 |
| Lebanon | 55-59 years | 1.14 | 1.13 | 1.24 |
| Bulgaria | 55-59 years | 1.15 | 1.15 | 1.06 |
| Guyana | 55-59 years | 1.16 | 1.11 | 1.31 |
| Niue | 55-59 years | 1.16 | 1.15 | 1.15 |
| Albania | 55-59 years | 1.16 | 1.11 | 1.27 |
| Sweden | 55-59 years | 1.17 | 1.11 | 1.58 |
| Malawi | 55-59 years | 1.17 | 1.17 | 1.22 |
| Uruguay | 55-59 years | 1.17 | 1.16 | 1.26 |
| Russian Federation | 55-59 years | 1.18 | 1.09 | 1.55 |
| Republic of Moldova | 55-59 years | 1.19 | 1.15 | 1.45 |
| Costa Rica | 55-59 years | 1.20 | 1.02 | 1.37 |
| Bangladesh | 55-59 years | 1.20 | 1.19 | 1.48 |
| Tajikistan | 55-59 years | 1.20 | 1.21 | 1.20 |
| Latvia | 55-59 years | 1.20 | 1.15 | 1.49 |
| Grenada | 55-59 years | 1.20 | 1.19 | 1.28 |
| Taiwan (Province of China) | 55-59 years | 1.21 | 1.06 | 1.45 |
| South Africa | 55-59 years | 1.21 | 1.21 | 1.24 |
| Kiribati | 55-59 years | 1.22 | 1.22 | 1.20 |
| United States of America | 55-59 years | 1.22 | 1.28 | 1.01 |
| Syrian Arab Republic | 55-59 years | 1.23 | 1.24 | 1.19 |
| Egypt | 55-59 years | 1.23 | 1.24 | 1.20 |
| Argentina | 55-59 years | 1.24 | 1.21 | 1.30 |
| Republic of Korea | 55-59 years | 1.24 | 1.14 | 1.68 |
| Iraq | 55-59 years | 1.25 | 1.26 | 1.21 |
| Venezuela (Bolivarian Republic of) | 55-59 years | 1.25 | 1.22 | 1.52 |
| Turkmenistan | 55-59 years | 1.26 | 1.26 | 1.26 |
| Canada | 55-59 years | 1.26 | 1.33 | 1.02 |
| Jamaica | 55-59 years | 1.26 | 1.29 | 1.21 |
| Myanmar | 55-59 years | 1.27 | 1.21 | 1.50 |
| Colombia | 55-59 years | 1.27 | 1.19 | 1.58 |
| South Sudan | 55-59 years | 1.27 | 1.29 | 1.20 |
| Central African Republic | 55-59 years | 1.28 | 1.31 | 1.03 |
| Azerbaijan | 55-59 years | 1.28 | 1.28 | 1.30 |
| Kuwait | 55-59 years | 1.29 | 1.26 | 1.39 |
| Gabon | 55-59 years | 1.29 | 1.38 | 1.08 |
| Afghanistan | 55-59 years | 1.29 | 1.32 | 1.12 |
| Ghana | 55-59 years | 1.30 | 1.37 | 1.17 |
| United Kingdom | 55-59 years | 1.30 | 1.30 | 1.33 |
| Lithuania | 55-59 years | 1.31 | 1.27 | 1.48 |
| Burundi | 55-59 years | 1.31 | 1.34 | 1.19 |
| Trinidad and Tobago | 55-59 years | 1.32 | 1.31 | 1.32 |
| Luxembourg | 55-59 years | 1.33 | 1.33 | 1.31 |
| Bosnia and Herzegovina | 55-59 years | 1.33 | 1.36 | 1.11 |
| Spain | 55-59 years | 1.34 | 1.31 | 1.53 |
| C么te d'Ivoire | 55-59 years | 1.34 | 1.40 | 1.19 |
| China | 55-59 years | 1.34 | 1.36 | 1.28 |
| Nigeria | 55-59 years | 1.35 | 1.45 | 1.18 |
| Panama | 55-59 years | 1.35 | 1.38 | 1.33 |
| Togo | 55-59 years | 1.35 | 1.41 | 1.16 |
| Belgium | 55-59 years | 1.35 | 1.34 | 1.45 |
| Paraguay | 55-59 years | 1.36 | 1.42 | 1.30 |
| Bhutan | 55-59 years | 1.37 | 1.37 | 1.26 |
| Zambia | 55-59 years | 1.37 | 1.42 | 1.23 |
| Bahamas | 55-59 years | 1.37 | 1.42 | 1.33 |
| Netherlands | 55-59 years | 1.38 | 1.40 | 1.26 |
| Suriname | 55-59 years | 1.38 | 1.43 | 1.30 |
| Philippines | 55-59 years | 1.38 | 1.39 | 1.37 |
| Somalia | 55-59 years | 1.39 | 1.42 | 1.19 |
| North Macedonia | 55-59 years | 1.39 | 1.41 | 1.13 |
| Nauru | 55-59 years | 1.39 | 1.40 | 1.13 |
| Fiji | 55-59 years | 1.39 | 1.40 | 1.20 |
| Belize | 55-59 years | 1.39 | 1.47 | 1.32 |
| Turkiye | 55-59 years | 1.40 | 1.39 | 1.50 |
| Yemen | 55-59 years | 1.42 | 1.46 | 1.17 |
| Ethiopia | 55-59 years | 1.42 | 1.47 | 1.25 |
| Sudan | 55-59 years | 1.42 | 1.46 | 1.22 |
| Equatorial Guinea | 55-59 years | 1.43 | 1.67 | 1.03 |
| Rwanda | 55-59 years | 1.43 | 1.51 | 1.20 |
| Algeria | 55-59 years | 1.43 | 1.57 | 1.14 |
| United Republic of Tanzania | 55-59 years | 1.44 | 1.52 | 1.22 |
| Dominica | 55-59 years | 1.44 | 1.52 | 1.28 |
| Madagascar | 55-59 years | 1.44 | 1.47 | 1.16 |
| San Marino | 55-59 years | 1.44 | 1.42 | 1.57 |
| Estonia | 55-59 years | 1.44 | 1.38 | 1.64 |
| Uzbekistan | 55-59 years | 1.45 | 1.47 | 1.23 |
| Guatemala | 55-59 years | 1.45 | 1.81 | 1.36 |
| Chile | 55-59 years | 1.45 | 1.46 | 1.38 |
| Chad | 55-59 years | 1.46 | 1.52 | 1.13 |
| Jordan | 55-59 years | 1.46 | 1.49 | 1.36 |
| Cyprus | 55-59 years | 1.46 | 1.45 | 1.67 |
| Micronesia (Federated States of) | 55-59 years | 1.47 | 1.49 | 1.21 |
| Thailand | 55-59 years | 1.48 | 1.54 | 1.45 |
| Cameroon | 55-59 years | 1.48 | 1.61 | 1.15 |
| Senegal | 55-59 years | 1.49 | 1.62 | 1.15 |
| Peru | 55-59 years | 1.49 | 1.91 | 1.25 |
| Singapore | 55-59 years | 1.49 | 1.45 | 1.69 |
| Angola | 55-59 years | 1.50 | 1.60 | 1.03 |
| Marshall Islands | 55-59 years | 1.50 | 1.52 | 1.19 |
| Djibouti | 55-59 years | 1.50 | 1.61 | 1.22 |
| Cambodia | 55-59 years | 1.51 | 1.62 | 1.18 |
| Nicaragua | 55-59 years | 1.52 | 1.67 | 1.38 |
| Kenya | 55-59 years | 1.54 | 1.72 | 1.22 |
| Ireland | 55-59 years | 1.55 | 1.55 | 1.48 |
| New Zealand | 55-59 years | 1.55 | 1.54 | 1.54 |
| Guinea-Bissau | 55-59 years | 1.55 | 1.62 | 1.13 |
| Nepal | 55-59 years | 1.55 | 1.57 | 1.11 |
| Malta | 55-59 years | 1.55 | 1.56 | 1.43 |
| Tonga | 55-59 years | 1.56 | 1.70 | 1.23 |
| Barbados | 55-59 years | 1.56 | 1.96 | 1.29 |
| India | 55-59 years | 1.56 | 1.57 | 1.40 |
| Palestine | 55-59 years | 1.56 | 1.76 | 1.23 |
| Sierra Leone | 55-59 years | 1.57 | 1.70 | 1.12 |
| Eritrea | 55-59 years | 1.57 | 1.65 | 1.21 |
| Ecuador | 55-59 years | 1.58 | 1.95 | 1.33 |
| Oman | 55-59 years | 1.58 | 1.59 | 1.62 |
| Lao People's Democratic Republic | 55-59 years | 1.58 | 1.70 | 1.26 |
| Pakistan | 55-59 years | 1.58 | 1.60 | 1.21 |
| Puerto Rico | 55-59 years | 1.58 | 1.59 | 1.49 |
| Australia | 55-59 years | 1.59 | 1.58 | 1.77 |
| Armenia | 55-59 years | 1.59 | 1.69 | 1.32 |
| Democratic Republic of the Congo | 55-59 years | 1.60 | 1.74 | 1.02 |
| Cuba | 55-59 years | 1.60 | 1.79 | 1.30 |
| Tuvalu | 55-59 years | 1.61 | 1.62 | 1.45 |
| Malaysia | 55-59 years | 1.61 | 1.77 | 1.40 |
| Dominican Republic | 55-59 years | 1.61 | 1.86 | 1.32 |
| El Salvador | 55-59 years | 1.62 | 1.92 | 1.47 |
| Guinea | 55-59 years | 1.63 | 1.74 | 1.12 |
| Comoros | 55-59 years | 1.64 | 1.83 | 1.17 |
| Brazil | 55-59 years | 1.64 | 1.77 | 1.44 |
| Morocco | 55-59 years | 1.65 | 1.79 | 1.16 |
| Libya | 55-59 years | 1.66 | 1.97 | 1.20 |
| Gambia | 55-59 years | 1.66 | 1.84 | 1.16 |
| Niger | 55-59 years | 1.68 | 1.81 | 1.13 |
| Democratic People's Republic of Korea | 55-59 years | 1.71 | 1.77 | 1.15 |
| Saint Lucia | 55-59 years | 1.73 | 2.01 | 1.33 |
| Maldives | 55-59 years | 1.74 | 3.28 | 1.20 |
| Brunei Darussalam | 55-59 years | 1.75 | 1.69 | 2.24 |
| Samoa | 55-59 years | 1.75 | 1.82 | 1.18 |
| Tokelau | 55-59 years | 1.77 | 1.78 | 1.50 |
| Timor-Leste | 55-59 years | 1.80 | 1.95 | 1.25 |
| Haiti | 55-59 years | 1.81 | 1.90 | 1.19 |
| Antigua and Barbuda | 55-59 years | 1.82 | 2.52 | 1.31 |
| Mauritius | 55-59 years | 1.85 | 2.52 | 1.42 |
| Mexico | 55-59 years | 1.85 | 2.05 | 1.49 |
| United States Virgin Islands | 55-59 years | 1.90 | 1.99 | 1.34 |
| Congo | 55-59 years | 1.92 | 2.23 | 1.00 |
| Liberia | 55-59 years | 1.94 | 2.24 | 1.11 |
| Indonesia | 55-59 years | 1.97 | 2.16 | 1.03 |
| Mali | 55-59 years | 2.00 | 2.41 | 1.10 |
| American Samoa | 55-59 years | 2.01 | 2.14 | 1.25 |
| Bolivia (Plurinational State of) | 55-59 years | 2.03 | 2.58 | 1.20 |
| Mauritania | 55-59 years | 2.03 | 2.64 | 1.03 |
| Northern Mariana Islands | 55-59 years | 2.14 | 2.36 | 1.26 |
| Honduras | 55-59 years | 2.32 | 3.87 | 1.23 |
| Greenland | 55-59 years | 2.39 | 2.86 | 1.08 |
| Bahrain | 55-59 years | 2.60 | 2.82 | 1.71 |
| Saudi Arabia | 55-59 years | 3.11 | 3.35 | 1.66 |
| Andorra | 55-59 years | 3.29 | 3.55 | 2.06 |
| Sao Tome and Principe | 55-59 years | 3.43 | 4.45 | 1.13 |
| Norway | 5-9 years | 0.31 | 0.09 | 0.79 |
| Kiribati | 5-9 years | 0.44 | 0.34 | 1.17 |
| Tuvalu | 5-9 years | 0.47 | 0.44 | 1.55 |
| Denmark | 5-9 years | 0.51 | 0.40 | 1.13 |
| Cook Islands | 5-9 years | 0.57 | 0.46 | 1.40 |
| Vanuatu | 5-9 years | 0.58 | 0.44 | 1.16 |
| Chad | 5-9 years | 0.60 | 0.35 | 1.00 |
| Nauru | 5-9 years | 0.61 | 0.58 | 1.51 |
| Ukraine | 5-9 years | 0.61 | 0.19 | 1.07 |
| Guinea | 5-9 years | 0.62 | 0.34 | 1.00 |
| Guinea-Bissau | 5-9 years | 0.62 | 0.24 | 1.00 |
| Luxembourg | 5-9 years | 0.63 | 0.12 | 1.13 |
| Burkina Faso | 5-9 years | 0.63 | 0.31 | 1.01 |
| Sierra Leone | 5-9 years | 0.63 | 0.33 | 1.01 |
| Afghanistan | 5-9 years | 0.63 | 0.55 | 1.09 |
| Nigeria | 5-9 years | 0.65 | 0.30 | 1.03 |
| Marshall Islands | 5-9 years | 0.66 | 0.51 | 1.16 |
| Benin | 5-9 years | 0.67 | 0.25 | 1.00 |
| C么te d'Ivoire | 5-9 years | 0.67 | 0.31 | 1.00 |
| Fiji | 5-9 years | 0.68 | 0.60 | 1.18 |
| Bhutan | 5-9 years | 0.69 | 0.61 | 1.06 |
| Liberia | 5-9 years | 0.70 | 0.35 | 1.01 |
| Micronesia (Federated States of) | 5-9 years | 0.71 | 0.53 | 1.16 |
| Egypt | 5-9 years | 0.72 | 0.65 | 1.07 |
| Sweden | 5-9 years | 0.72 | 0.36 | 1.17 |
| Niger | 5-9 years | 0.73 | 0.44 | 0.99 |
| Togo | 5-9 years | 0.73 | 0.34 | 1.00 |
| Greece | 5-9 years | 0.73 | 0.74 | 0.64 |
| Mali | 5-9 years | 0.74 | 0.38 | 1.01 |
| Gambia | 5-9 years | 0.75 | 0.32 | 1.00 |
| Nepal | 5-9 years | 0.76 | 0.70 | 0.98 |
| Cameroon | 5-9 years | 0.76 | 0.36 | 1.00 |
| Solomon Islands | 5-9 years | 0.76 | 0.60 | 1.16 |
| Malta | 5-9 years | 0.76 | 0.71 | 1.10 |
| Colombia | 5-9 years | 0.77 | 0.56 | 1.28 |
| Senegal | 5-9 years | 0.77 | 0.36 | 1.00 |
| Ireland | 5-9 years | 0.78 | 0.48 | 1.13 |
| Cabo Verde | 5-9 years | 0.78 | 0.23 | 1.00 |
| Palau | 5-9 years | 0.78 | 0.74 | 1.32 |
| Sao Tome and Principe | 5-9 years | 0.79 | 0.28 | 1.01 |
| Mauritania | 5-9 years | 0.79 | 0.34 | 1.00 |
| Libya | 5-9 years | 0.80 | 0.73 | 1.09 |
| New Zealand | 5-9 years | 0.80 | 0.53 | 1.19 |
| Jordan | 5-9 years | 0.80 | 0.76 | 1.39 |
| Canada | 5-9 years | 0.81 | 0.75 | 0.94 |
| North Macedonia | 5-9 years | 0.82 | 0.64 | 1.08 |
| Australia | 5-9 years | 0.82 | 0.63 | 1.19 |
| Zimbabwe | 5-9 years | 0.83 | 0.76 | 1.03 |
| Samoa | 5-9 years | 0.83 | 0.61 | 1.17 |
| Eswatini | 5-9 years | 0.83 | 0.62 | 1.03 |
| Central African Republic | 5-9 years | 0.83 | 0.77 | 0.87 |
| Mozambique | 5-9 years | 0.84 | 0.36 | 1.04 |
| Congo | 5-9 years | 0.84 | 0.72 | 0.87 |
| Madagascar | 5-9 years | 0.85 | 0.62 | 1.05 |
| Niue | 5-9 years | 0.85 | 0.84 | 1.17 |
| Chile | 5-9 years | 0.85 | 0.73 | 1.11 |
| Ghana | 5-9 years | 0.85 | 0.24 | 1.00 |
| Lesotho | 5-9 years | 0.86 | 0.70 | 1.03 |
| Tokelau | 5-9 years | 0.86 | 0.85 | 1.69 |
| Qatar | 5-9 years | 0.86 | 0.79 | 1.34 |
| Sudan | 5-9 years | 0.86 | 0.81 | 1.11 |
| Gabon | 5-9 years | 0.86 | 0.72 | 0.87 |
| Guam | 5-9 years | 0.87 | 0.04 | 1.14 |
| United States of America | 5-9 years | 0.87 | 0.80 | 0.92 |
| Cyprus | 5-9 years | 0.87 | 0.74 | 1.18 |
| Angola | 5-9 years | 0.88 | 0.94 | 0.86 |
| Papua New Guinea | 5-9 years | 0.89 | 0.86 | 1.15 |
| Belarus | 5-9 years | 0.89 | 0.66 | 1.02 |
| Equatorial Guinea | 5-9 years | 0.90 | 1.09 | 0.87 |
| Democratic Republic of the Congo | 5-9 years | 0.91 | 1.07 | 0.87 |
| Jamaica | 5-9 years | 0.91 | 0.54 | 1.11 |
| Montenegro | 5-9 years | 0.91 | 0.46 | 1.14 |
| Iraq | 5-9 years | 0.92 | 0.77 | 1.09 |
| Namibia | 5-9 years | 0.92 | 0.72 | 1.03 |
| Lebanon | 5-9 years | 0.93 | 0.89 | 1.35 |
| United Arab Emirates | 5-9 years | 0.93 | 0.79 | 1.09 |
| Somalia | 5-9 years | 0.94 | 0.64 | 1.05 |
| Lithuania | 5-9 years | 0.94 | 1.05 | 1.00 |
| Botswana | 5-9 years | 0.94 | 0.75 | 1.03 |
| Timor-Leste | 5-9 years | 0.94 | 0.88 | 1.14 |
| American Samoa | 5-9 years | 0.95 | 0.71 | 1.17 |
| United Republic of Tanzania | 5-9 years | 0.96 | 0.70 | 1.05 |
| Kuwait | 5-9 years | 0.96 | 0.93 | 1.40 |
| Iran (Islamic Republic of) | 5-9 years | 0.97 | 0.85 | 1.10 |
| China | 5-9 years | 0.97 | 0.61 | 1.05 |
| Germany | 5-9 years | 0.98 | 0.93 | 1.14 |
| Russian Federation | 5-9 years | 0.98 | 0.83 | 1.05 |
| Viet Nam | 5-9 years | 0.98 | 0.96 | 1.08 |
| Tonga | 5-9 years | 0.99 | 0.68 | 1.16 |
| Malawi | 5-9 years | 0.99 | 0.77 | 1.05 |
| India | 5-9 years | 1.00 | 0.95 | 1.15 |
| Djibouti | 5-9 years | 1.00 | 0.75 | 1.05 |
| Syrian Arab Republic | 5-9 years | 1.00 | 0.98 | 1.09 |
| Belize | 5-9 years | 1.01 | 0.44 | 1.12 |
| Uganda | 5-9 years | 1.01 | 0.79 | 1.06 |
| Saint Kitts and Nevis | 5-9 years | 1.01 | 0.99 | 1.24 |
| South Sudan | 5-9 years | 1.01 | 0.94 | 1.06 |
| Eritrea | 5-9 years | 1.01 | 0.85 | 1.05 |
| Zambia | 5-9 years | 1.01 | 0.80 | 1.05 |
| Mongolia | 5-9 years | 1.02 | 0.68 | 1.14 |
| Democratic People's Republic of Korea | 5-9 years | 1.03 | 0.70 | 1.06 |
| Honduras | 5-9 years | 1.03 | 0.69 | 1.06 |
| Bermuda | 5-9 years | 1.03 | 0.89 | 1.27 |
| Grenada | 5-9 years | 1.04 | 0.92 | 1.10 |
| Pakistan | 5-9 years | 1.04 | 1.05 | 1.04 |
| Tajikistan | 5-9 years | 1.04 | 0.94 | 1.15 |
| Comoros | 5-9 years | 1.04 | 1.00 | 1.05 |
| Lao People's Democratic Republic | 5-9 years | 1.04 | 1.02 | 1.12 |
| Kenya | 5-9 years | 1.04 | 0.77 | 1.06 |
| Bolivia (Plurinational State of) | 5-9 years | 1.04 | 0.87 | 1.10 |
| Guatemala | 5-9 years | 1.04 | 0.98 | 1.05 |
| Hungary | 5-9 years | 1.04 | 0.89 | 1.15 |
| Rwanda | 5-9 years | 1.05 | 1.00 | 1.05 |
| Mexico | 5-9 years | 1.05 | 0.80 | 1.09 |
| Turkmenistan | 5-9 years | 1.06 | 0.85 | 1.15 |
| Nicaragua | 5-9 years | 1.06 | 0.77 | 1.07 |
| Venezuela (Bolivarian Republic of) | 5-9 years | 1.07 | 0.98 | 1.28 |
| Indonesia | 5-9 years | 1.07 | 1.04 | 1.17 |
| Peru | 5-9 years | 1.07 | 0.83 | 1.10 |
| South Africa | 5-9 years | 1.07 | 1.11 | 1.05 |
| Costa Rica | 5-9 years | 1.07 | 0.62 | 1.09 |
| Ecuador | 5-9 years | 1.08 | 0.94 | 1.10 |
| Burundi | 5-9 years | 1.08 | 1.08 | 1.05 |
| El Salvador | 5-9 years | 1.08 | 1.14 | 1.07 |
| Cuba | 5-9 years | 1.08 | 0.92 | 1.12 |
| France | 5-9 years | 1.08 | 1.05 | 1.15 |
| Ethiopia | 5-9 years | 1.08 | 0.98 | 1.11 |
| Paraguay | 5-9 years | 1.08 | 1.11 | 1.10 |
| Estonia | 5-9 years | 1.09 | 0.83 | 1.00 |
| Japan | 5-9 years | 1.09 | 1.20 | 0.90 |
| Albania | 5-9 years | 1.10 | 0.71 | 1.13 |
| Armenia | 5-9 years | 1.10 | 0.81 | 1.15 |
| Dominican Republic | 5-9 years | 1.11 | 1.09 | 1.12 |
| Trinidad and Tobago | 5-9 years | 1.11 | 1.05 | 1.12 |
| Northern Mariana Islands | 5-9 years | 1.11 | 0.94 | 1.16 |
| Brazil | 5-9 years | 1.11 | 0.97 | 1.15 |
| Uzbekistan | 5-9 years | 1.12 | 1.09 | 1.14 |
| Panama | 5-9 years | 1.12 | 1.85 | 1.08 |
| Guyana | 5-9 years | 1.12 | 1.16 | 1.10 |
| Puerto Rico | 5-9 years | 1.12 | 0.91 | 1.28 |
| Cambodia | 5-9 years | 1.12 | 1.16 | 1.08 |
| Greenland | 5-9 years | 1.12 | 1.38 | 0.95 |
| Georgia | 5-9 years | 1.13 | 1.05 | 1.14 |
| Argentina | 5-9 years | 1.13 | 1.20 | 1.13 |
| Finland | 5-9 years | 1.13 | 1.13 | 1.11 |
| Bahamas | 5-9 years | 1.13 | 1.33 | 1.11 |
| Myanmar | 5-9 years | 1.13 | 1.10 | 1.23 |
| Tunisia | 5-9 years | 1.14 | 1.11 | 1.34 |
| Morocco | 5-9 years | 1.14 | 1.19 | 1.09 |
| Algeria | 5-9 years | 1.15 | 1.23 | 1.07 |
| Taiwan (Province of China) | 5-9 years | 1.15 | 1.03 | 1.29 |
| Palestine | 5-9 years | 1.15 | 1.19 | 1.09 |
| Maldives | 5-9 years | 1.16 | 1.20 | 1.14 |
| Slovakia | 5-9 years | 1.16 | 1.00 | 1.13 |
| Brunei Darussalam | 5-9 years | 1.17 | 1.83 | 0.98 |
| Republic of Korea | 5-9 years | 1.17 | 1.49 | 0.95 |
| Bulgaria | 5-9 years | 1.17 | 1.18 | 1.16 |
| Azerbaijan | 5-9 years | 1.18 | 1.22 | 1.15 |
| Kyrgyzstan | 5-9 years | 1.18 | 1.29 | 1.14 |
| Thailand | 5-9 years | 1.18 | 1.30 | 1.15 |
| Serbia | 5-9 years | 1.18 | 1.05 | 1.10 |
| Kazakhstan | 5-9 years | 1.18 | 1.17 | 1.16 |
| Latvia | 5-9 years | 1.19 | 1.69 | 1.02 |
| Singapore | 5-9 years | 1.19 | 1.40 | 0.96 |
| Czechia | 5-9 years | 1.19 | 1.32 | 1.12 |
| Republic of Moldova | 5-9 years | 1.20 | 1.26 | 1.02 |
| Oman | 5-9 years | 1.20 | 1.15 | 1.40 |
| Antigua and Barbuda | 5-9 years | 1.20 | 2.82 | 1.09 |
| Uruguay | 5-9 years | 1.21 | 1.45 | 1.08 |
| Yemen | 5-9 years | 1.21 | 1.27 | 1.07 |
| Malaysia | 5-9 years | 1.21 | 1.41 | 1.15 |
| Belgium | 5-9 years | 1.21 | 1.12 | 1.39 |
| Mauritius | 5-9 years | 1.22 | 1.41 | 1.14 |
| Barbados | 5-9 years | 1.22 | 3.19 | 1.09 |
| Austria | 5-9 years | 1.22 | 1.68 | 0.78 |
| Dominica | 5-9 years | 1.23 | 1.54 | 1.11 |
| United Kingdom | 5-9 years | 1.25 | 1.30 | 1.15 |
| Saudi Arabia | 5-9 years | 1.26 | 1.16 | 1.47 |
| Turkiye | 5-9 years | 1.27 | 1.24 | 1.42 |
| Monaco | 5-9 years | 1.29 | 1.56 | 1.12 |
| Saint Lucia | 5-9 years | 1.29 | 2.17 | 1.10 |
| Romania | 5-9 years | 1.31 | 1.40 | 1.16 |
| Suriname | 5-9 years | 1.31 | 1.79 | 1.11 |
| Philippines | 5-9 years | 1.34 | 1.46 | 1.17 |
| Sri Lanka | 5-9 years | 1.37 | 1.40 | 1.19 |
| Israel | 5-9 years | 1.42 | 1.57 | 1.12 |
| Seychelles | 5-9 years | 1.42 | 18.86 | 1.15 |
| Portugal | 5-9 years | 1.46 | 1.85 | 0.86 |
| San Marino | 5-9 years | 1.49 | 1.87 | 1.15 |
| Italy | 5-9 years | 1.50 | 1.76 | 1.16 |
| Poland | 5-9 years | 1.51 | 1.92 | 1.05 |
| Bosnia and Herzegovina | 5-9 years | 1.51 | 1.80 | 1.15 |
| United States Virgin Islands | 5-9 years | 1.54 | 3.77 | 1.29 |
| Netherlands | 5-9 years | 1.54 | 2.14 | 1.15 |
| Spain | 5-9 years | 1.57 | 1.66 | 1.29 |
| Iceland | 5-9 years | 1.63 | 1.93 | 1.47 |
| Haiti | 5-9 years | 1.64 | 1.81 | 1.10 |
| Saint Vincent and the Grenadines | 5-9 years | 1.66 | 4.05 | 1.10 |
| Switzerland | 5-9 years | 1.68 | 2.32 | 1.14 |
| Bahrain | 5-9 years | 1.74 | 1.82 | 1.44 |
| Croatia | 5-9 years | 1.92 | 2.54 | 1.42 |
| Andorra | 5-9 years | 2.01 | 4.53 | 1.13 |
| Bangladesh | 5-9 years | 2.68 | 3.14 | 1.19 |
| Slovenia | 5-9 years | 2.78 | 12.99 | 1.27 |
| Palau | 60-64 years | 0.31 | 0.28 | 1.12 |
| Cook Islands | 60-64 years | 0.36 | 0.30 | 0.77 |
| Bermuda | 60-64 years | 0.61 | 0.51 | 1.26 |
| Vanuatu | 60-64 years | 0.65 | 0.63 | 1.25 |
| Solomon Islands | 60-64 years | 0.65 | 0.64 | 1.16 |
| Papua New Guinea | 60-64 years | 0.66 | 0.64 | 1.17 |
| Romania | 60-64 years | 0.66 | 0.62 | 1.17 |
| Poland | 60-64 years | 0.67 | 0.66 | 0.80 |
| Namibia | 60-64 years | 0.69 | 0.60 | 1.25 |
| Germany | 60-64 years | 0.70 | 0.67 | 1.21 |
| Cabo Verde | 60-64 years | 0.71 | 0.56 | 1.36 |
| Czechia | 60-64 years | 0.71 | 0.66 | 1.11 |
| Burkina Faso | 60-64 years | 0.72 | 0.70 | 1.17 |
| Kiribati | 60-64 years | 0.73 | 0.72 | 1.21 |
| Benin | 60-64 years | 0.74 | 0.68 | 1.18 |
| Eswatini | 60-64 years | 0.76 | 0.69 | 1.22 |
| Niue | 60-64 years | 0.76 | 0.74 | 1.11 |
| Lesotho | 60-64 years | 0.76 | 0.72 | 1.22 |
| Norway | 60-64 years | 0.78 | 0.74 | 1.06 |
| Botswana | 60-64 years | 0.79 | 0.67 | 1.28 |
| Portugal | 60-64 years | 0.81 | 0.78 | 1.21 |
| Nauru | 60-64 years | 0.84 | 0.83 | 1.08 |
| United Arab Emirates | 60-64 years | 0.86 | 0.78 | 1.22 |
| Guam | 60-64 years | 0.86 | 0.74 | 1.33 |
| Mongolia | 60-64 years | 0.89 | 0.86 | 1.27 |
| Fiji | 60-64 years | 0.89 | 0.88 | 1.22 |
| Zimbabwe | 60-64 years | 0.89 | 0.88 | 1.19 |
| France | 60-64 years | 0.90 | 0.86 | 1.24 |
| Marshall Islands | 60-64 years | 0.92 | 0.91 | 1.19 |
| Ghana | 60-64 years | 0.92 | 0.87 | 1.18 |
| Hungary | 60-64 years | 0.93 | 0.90 | 1.14 |
| Micronesia (Federated States of) | 60-64 years | 0.93 | 0.92 | 1.21 |
| Croatia | 60-64 years | 0.94 | 0.90 | 1.35 |
| Montenegro | 60-64 years | 0.94 | 0.92 | 1.09 |
| C么te d'Ivoire | 60-64 years | 0.96 | 0.92 | 1.20 |
| Saint Vincent and the Grenadines | 60-64 years | 0.96 | 0.84 | 1.30 |
| Togo | 60-64 years | 0.96 | 0.94 | 1.17 |
| Slovakia | 60-64 years | 0.96 | 0.96 | 0.97 |
| Chad | 60-64 years | 0.96 | 0.95 | 1.14 |
| Georgia | 60-64 years | 0.97 | 0.93 | 1.42 |
| Nigeria | 60-64 years | 0.97 | 0.93 | 1.20 |
| Saint Kitts and Nevis | 60-64 years | 0.99 | 0.92 | 1.60 |
| Bangladesh | 60-64 years | 0.99 | 0.98 | 1.50 |
| Tuvalu | 60-64 years | 1.00 | 0.98 | 1.40 |
| Viet Nam | 60-64 years | 1.00 | 1.11 | 0.82 |
| Sri Lanka | 60-64 years | 1.01 | 1.16 | 0.70 |
| Bulgaria | 60-64 years | 1.01 | 1.01 | 1.01 |
| Austria | 60-64 years | 1.02 | 0.99 | 1.27 |
| Greece | 60-64 years | 1.02 | 1.00 | 1.18 |
| Denmark | 60-64 years | 1.02 | 1.00 | 1.41 |
| Japan | 60-64 years | 1.02 | 0.85 | 1.82 |
| Samoa | 60-64 years | 1.04 | 1.03 | 1.20 |
| Jamaica | 60-64 years | 1.05 | 0.97 | 1.22 |
| Senegal | 60-64 years | 1.05 | 1.04 | 1.16 |
| Tonga | 60-64 years | 1.05 | 1.00 | 1.24 |
| Serbia | 60-64 years | 1.05 | 1.06 | 1.02 |
| Slovenia | 60-64 years | 1.06 | 1.04 | 1.18 |
| Guinea-Bissau | 60-64 years | 1.06 | 1.05 | 1.14 |
| Sierra Leone | 60-64 years | 1.06 | 1.06 | 1.13 |
| Switzerland | 60-64 years | 1.06 | 1.03 | 1.27 |
| Guyana | 60-64 years | 1.06 | 0.99 | 1.31 |
| Tokelau | 60-64 years | 1.06 | 1.04 | 1.42 |
| Finland | 60-64 years | 1.06 | 1.04 | 1.36 |
| Italy | 60-64 years | 1.08 | 1.06 | 1.19 |
| South Africa | 60-64 years | 1.08 | 1.03 | 1.26 |
| Trinidad and Tobago | 60-64 years | 1.08 | 0.95 | 1.33 |
| Kazakhstan | 60-64 years | 1.08 | 1.07 | 1.30 |
| Argentina | 60-64 years | 1.09 | 0.98 | 1.33 |
| Cameroon | 60-64 years | 1.09 | 1.08 | 1.16 |
| Guinea | 60-64 years | 1.12 | 1.12 | 1.12 |
| Seychelles | 60-64 years | 1.12 | 0.77 | 1.47 |
| Niger | 60-64 years | 1.12 | 1.13 | 1.13 |
| Gambia | 60-64 years | 1.13 | 1.12 | 1.17 |
| Qatar | 60-64 years | 1.13 | 1.09 | 1.44 |
| Monaco | 60-64 years | 1.13 | 1.11 | 1.27 |
| Kyrgyzstan | 60-64 years | 1.13 | 1.12 | 1.33 |
| Central African Republic | 60-64 years | 1.13 | 1.14 | 1.03 |
| United States of America | 60-64 years | 1.14 | 1.20 | 0.97 |
| Uruguay | 60-64 years | 1.15 | 1.13 | 1.24 |
| Tunisia | 60-64 years | 1.15 | 1.13 | 1.36 |
| Bhutan | 60-64 years | 1.16 | 1.15 | 1.26 |
| Iraq | 60-64 years | 1.16 | 1.15 | 1.22 |
| India | 60-64 years | 1.16 | 1.16 | 1.41 |
| Sweden | 60-64 years | 1.16 | 1.10 | 1.55 |
| Afghanistan | 60-64 years | 1.20 | 1.20 | 1.12 |
| Syrian Arab Republic | 60-64 years | 1.20 | 1.20 | 1.21 |
| Lebanon | 60-64 years | 1.20 | 1.19 | 1.20 |
| North Macedonia | 60-64 years | 1.21 | 1.22 | 1.13 |
| Suriname | 60-64 years | 1.21 | 1.15 | 1.32 |
| Albania | 60-64 years | 1.21 | 1.18 | 1.31 |
| Chile | 60-64 years | 1.22 | 1.19 | 1.38 |
| Canada | 60-64 years | 1.23 | 1.30 | 1.00 |
| Luxembourg | 60-64 years | 1.23 | 1.21 | 1.35 |
| Mozambique | 60-64 years | 1.23 | 1.23 | 1.23 |
| Grenada | 60-64 years | 1.23 | 1.22 | 1.30 |
| Bosnia and Herzegovina | 60-64 years | 1.24 | 1.25 | 1.13 |
| Iran (Islamic Republic of) | 60-64 years | 1.24 | 1.23 | 1.29 |
| Colombia | 60-64 years | 1.24 | 1.17 | 1.50 |
| Kuwait | 60-64 years | 1.25 | 1.22 | 1.33 |
| Taiwan (Province of China) | 60-64 years | 1.25 | 1.14 | 1.43 |
| Gabon | 60-64 years | 1.26 | 1.31 | 1.09 |
| Turkmenistan | 60-64 years | 1.26 | 1.26 | 1.28 |
| Venezuela (Bolivarian Republic of) | 60-64 years | 1.27 | 1.24 | 1.46 |
| Uganda | 60-64 years | 1.27 | 1.28 | 1.27 |
| Spain | 60-64 years | 1.28 | 1.23 | 1.60 |
| Israel | 60-64 years | 1.28 | 1.27 | 1.43 |
| Tajikistan | 60-64 years | 1.29 | 1.31 | 1.22 |
| Bahamas | 60-64 years | 1.30 | 1.24 | 1.36 |
| Dominica | 60-64 years | 1.30 | 1.30 | 1.31 |
| American Samoa | 60-64 years | 1.30 | 1.31 | 1.26 |
| Belarus | 60-64 years | 1.31 | 1.28 | 1.51 |
| Nepal | 60-64 years | 1.31 | 1.32 | 1.11 |
| Paraguay | 60-64 years | 1.32 | 1.31 | 1.32 |
| Republic of Korea | 60-64 years | 1.32 | 1.18 | 1.83 |
| United Kingdom | 60-64 years | 1.32 | 1.31 | 1.39 |
| Ecuador | 60-64 years | 1.33 | 1.32 | 1.34 |
| Myanmar | 60-64 years | 1.33 | 1.29 | 1.51 |
| Azerbaijan | 60-64 years | 1.33 | 1.35 | 1.33 |
| Philippines | 60-64 years | 1.34 | 1.33 | 1.39 |
| Peru | 60-64 years | 1.35 | 1.44 | 1.26 |
| Liberia | 60-64 years | 1.36 | 1.40 | 1.11 |
| Yemen | 60-64 years | 1.37 | 1.40 | 1.17 |
| Puerto Rico | 60-64 years | 1.37 | 1.35 | 1.44 |
| China | 60-64 years | 1.37 | 1.37 | 1.39 |
| Malawi | 60-64 years | 1.38 | 1.41 | 1.23 |
| Belize | 60-64 years | 1.38 | 1.44 | 1.33 |
| Egypt | 60-64 years | 1.39 | 1.41 | 1.20 |
| Belgium | 60-64 years | 1.40 | 1.38 | 1.48 |
| Jordan | 60-64 years | 1.40 | 1.43 | 1.31 |
| Sudan | 60-64 years | 1.40 | 1.43 | 1.22 |
| Equatorial Guinea | 60-64 years | 1.41 | 1.55 | 1.03 |
| Angola | 60-64 years | 1.41 | 1.47 | 1.04 |
| South Sudan | 60-64 years | 1.43 | 1.45 | 1.21 |
| Cuba | 60-64 years | 1.43 | 1.49 | 1.32 |
| Costa Rica | 60-64 years | 1.43 | 1.47 | 1.39 |
| Algeria | 60-64 years | 1.43 | 1.53 | 1.15 |
| Ukraine | 60-64 years | 1.44 | 1.41 | 1.64 |
| Australia | 60-64 years | 1.45 | 1.42 | 1.58 |
| Republic of Moldova | 60-64 years | 1.45 | 1.45 | 1.50 |
| Guatemala | 60-64 years | 1.45 | 1.78 | 1.36 |
| Russian Federation | 60-64 years | 1.46 | 1.42 | 1.63 |
| Mauritania | 60-64 years | 1.46 | 1.56 | 1.04 |
| Pakistan | 60-64 years | 1.46 | 1.47 | 1.21 |
| Turkiye | 60-64 years | 1.46 | 1.46 | 1.46 |
| Cyprus | 60-64 years | 1.46 | 1.45 | 1.75 |
| Panama | 60-64 years | 1.48 | 1.62 | 1.34 |
| Cambodia | 60-64 years | 1.48 | 1.55 | 1.19 |
| Haiti | 60-64 years | 1.48 | 1.52 | 1.20 |
| Uzbekistan | 60-64 years | 1.49 | 1.51 | 1.25 |
| Mali | 60-64 years | 1.50 | 1.57 | 1.10 |
| Morocco | 60-64 years | 1.50 | 1.57 | 1.16 |
| United States Virgin Islands | 60-64 years | 1.50 | 1.54 | 1.32 |
| Burundi | 60-64 years | 1.50 | 1.55 | 1.20 |
| Northern Mariana Islands | 60-64 years | 1.54 | 1.63 | 1.27 |
| Dominican Republic | 60-64 years | 1.54 | 1.71 | 1.34 |
| Somalia | 60-64 years | 1.54 | 1.58 | 1.19 |
| Lithuania | 60-64 years | 1.54 | 1.54 | 1.54 |
| Saint Lucia | 60-64 years | 1.55 | 1.67 | 1.35 |
| Latvia | 60-64 years | 1.55 | 1.55 | 1.54 |
| Democratic Republic of the Congo | 60-64 years | 1.55 | 1.63 | 1.02 |
| Netherlands | 60-64 years | 1.56 | 1.60 | 1.30 |
| San Marino | 60-64 years | 1.57 | 1.56 | 1.65 |
| Ethiopia | 60-64 years | 1.59 | 1.65 | 1.26 |
| Democratic People's Republic of Korea | 60-64 years | 1.59 | 1.62 | 1.16 |
| Thailand | 60-64 years | 1.59 | 1.81 | 1.46 |
| Brazil | 60-64 years | 1.59 | 1.66 | 1.47 |
| Nicaragua | 60-64 years | 1.60 | 1.81 | 1.39 |
| Antigua and Barbuda | 60-64 years | 1.60 | 1.89 | 1.34 |
| Brunei Darussalam | 60-64 years | 1.60 | 1.53 | 2.33 |
| Rwanda | 60-64 years | 1.60 | 1.70 | 1.21 |
| Malaysia | 60-64 years | 1.60 | 1.74 | 1.41 |
| Lao People's Democratic Republic | 60-64 years | 1.61 | 1.73 | 1.26 |
| Kenya | 60-64 years | 1.62 | 1.75 | 1.23 |
| Barbados | 60-64 years | 1.62 | 2.08 | 1.31 |
| Zambia | 60-64 years | 1.63 | 1.72 | 1.24 |
| Estonia | 60-64 years | 1.63 | 1.61 | 1.68 |
| Palestine | 60-64 years | 1.64 | 1.82 | 1.24 |
| Madagascar | 60-64 years | 1.65 | 1.69 | 1.17 |
| Singapore | 60-64 years | 1.68 | 1.67 | 1.73 |
| Mauritius | 60-64 years | 1.68 | 1.95 | 1.45 |
| Ireland | 60-64 years | 1.68 | 1.69 | 1.52 |
| El Salvador | 60-64 years | 1.71 | 2.12 | 1.49 |
| Eritrea | 60-64 years | 1.72 | 1.80 | 1.22 |
| Djibouti | 60-64 years | 1.74 | 1.87 | 1.23 |
| New Zealand | 60-64 years | 1.75 | 1.77 | 1.36 |
| Timor-Leste | 60-64 years | 1.76 | 1.90 | 1.25 |
| Maldives | 60-64 years | 1.76 | 3.08 | 1.21 |
| United Republic of Tanzania | 60-64 years | 1.80 | 1.94 | 1.23 |
| Bolivia (Plurinational State of) | 60-64 years | 1.82 | 2.07 | 1.21 |
| Iceland | 60-64 years | 1.83 | 1.93 | 1.06 |
| Congo | 60-64 years | 1.85 | 2.03 | 1.00 |
| Armenia | 60-64 years | 1.85 | 2.01 | 1.36 |
| Oman | 60-64 years | 1.86 | 1.90 | 1.60 |
| Comoros | 60-64 years | 1.86 | 2.05 | 1.18 |
| Libya | 60-64 years | 1.88 | 2.20 | 1.19 |
| Indonesia | 60-64 years | 1.92 | 2.13 | 1.07 |
| Mexico | 60-64 years | 2.02 | 2.24 | 1.49 |
| Greenland | 60-64 years | 2.10 | 2.57 | 1.07 |
| Malta | 60-64 years | 2.12 | 2.17 | 1.50 |
| Honduras | 60-64 years | 2.31 | 3.43 | 1.24 |
| Sao Tome and Principe | 60-64 years | 2.35 | 2.57 | 1.14 |
| Bahrain | 60-64 years | 2.54 | 2.82 | 1.71 |
| Saudi Arabia | 60-64 years | 2.81 | 3.14 | 1.58 |
| Andorra | 60-64 years | 3.35 | 3.63 | 2.25 |
| Cook Islands | 65-69 years | 0.35 | 0.28 | 0.75 |
| Papua New Guinea | 65-69 years | 0.55 | 0.54 | 1.18 |
| Bermuda | 65-69 years | 0.58 | 0.46 | 1.23 |
| Vanuatu | 65-69 years | 0.60 | 0.58 | 1.25 |
| Solomon Islands | 65-69 years | 0.60 | 0.59 | 1.16 |
| Burkina Faso | 65-69 years | 0.63 | 0.62 | 1.18 |
| Palau | 65-69 years | 0.64 | 0.62 | 1.10 |
| Cabo Verde | 65-69 years | 0.67 | 0.56 | 1.38 |
| Kiribati | 65-69 years | 0.70 | 0.69 | 1.22 |
| Niue | 65-69 years | 0.70 | 0.68 | 1.07 |
| Benin | 65-69 years | 0.73 | 0.69 | 1.19 |
| Namibia | 65-69 years | 0.73 | 0.67 | 1.27 |
| Nauru | 65-69 years | 0.74 | 0.73 | 1.04 |
| Saint Vincent and the Grenadines | 65-69 years | 0.75 | 0.59 | 1.31 |
| Fiji | 65-69 years | 0.76 | 0.74 | 1.22 |
| Czechia | 65-69 years | 0.76 | 0.71 | 1.15 |
| Lesotho | 65-69 years | 0.77 | 0.75 | 1.23 |
| Romania | 65-69 years | 0.78 | 0.74 | 1.20 |
| Poland | 65-69 years | 0.79 | 0.78 | 0.90 |
| Marshall Islands | 65-69 years | 0.80 | 0.79 | 1.20 |
| Eswatini | 65-69 years | 0.81 | 0.77 | 1.23 |
| Botswana | 65-69 years | 0.82 | 0.74 | 1.29 |
| Germany | 65-69 years | 0.83 | 0.80 | 1.28 |
| Chad | 65-69 years | 0.88 | 0.87 | 1.14 |
| Micronesia (Federated States of) | 65-69 years | 0.89 | 0.88 | 1.22 |
| Bulgaria | 65-69 years | 0.89 | 0.89 | 1.02 |
| Ghana | 65-69 years | 0.91 | 0.88 | 1.19 |
| Tuvalu | 65-69 years | 0.92 | 3.52 | 1.33 |
| Togo | 65-69 years | 0.92 | 0.91 | 1.18 |
| France | 65-69 years | 0.92 | 0.87 | 1.27 |
| C么te d'Ivoire | 65-69 years | 0.93 | 0.91 | 1.22 |
| Samoa | 65-69 years | 0.94 | 0.92 | 1.22 |
| United Arab Emirates | 65-69 years | 0.94 | 0.93 | 1.21 |
| Hungary | 65-69 years | 0.94 | 0.90 | 1.21 |
| Montenegro | 65-69 years | 0.94 | 0.93 | 1.10 |
| Mongolia | 65-69 years | 0.95 | 0.93 | 1.28 |
| Zimbabwe | 65-69 years | 0.95 | 0.94 | 1.20 |
| Guinea-Bissau | 65-69 years | 0.96 | 0.95 | 1.15 |
| Japan | 65-69 years | 0.96 | 0.79 | 1.83 |
| Norway | 65-69 years | 0.96 | 0.94 | 1.12 |
| Seychelles | 65-69 years | 0.97 | 0.64 | 1.49 |
| Tokelau | 65-69 years | 0.97 | 0.95 | 1.34 |
| Guinea | 65-69 years | 0.97 | 0.97 | 1.13 |
| Niger | 65-69 years | 0.98 | 0.98 | 1.13 |
| Sierra Leone | 65-69 years | 0.99 | 0.99 | 1.13 |
| Sri Lanka | 65-69 years | 0.99 | 1.21 | 0.62 |
| Senegal | 65-69 years | 0.99 | 0.98 | 1.17 |
| Venezuela (Bolivarian Republic of) | 65-69 years | 0.99 | 0.94 | 1.42 |
| Guam | 65-69 years | 1.00 | 0.91 | 1.34 |
| Nigeria | 65-69 years | 1.00 | 0.98 | 1.21 |
| Gambia | 65-69 years | 1.02 | 1.01 | 1.18 |
| Afghanistan | 65-69 years | 1.02 | 1.02 | 1.12 |
| Georgia | 65-69 years | 1.03 | 1.00 | 1.44 |
| Viet Nam | 65-69 years | 1.03 | 1.22 | 0.74 |
| Argentina | 65-69 years | 1.03 | 0.94 | 1.36 |
| Cameroon | 65-69 years | 1.03 | 1.02 | 1.17 |
| Colombia | 65-69 years | 1.04 | 0.92 | 1.45 |
| Iraq | 65-69 years | 1.04 | 1.02 | 1.23 |
| Croatia | 65-69 years | 1.05 | 1.01 | 1.46 |
| Finland | 65-69 years | 1.06 | 1.03 | 1.42 |
| Austria | 65-69 years | 1.07 | 1.03 | 1.36 |
| Bangladesh | 65-69 years | 1.07 | 1.06 | 1.52 |
| Tonga | 65-69 years | 1.08 | 1.04 | 1.25 |
| Saint Kitts and Nevis | 65-69 years | 1.08 | 1.00 | 1.57 |
| Slovakia | 65-69 years | 1.08 | 1.10 | 0.98 |
| Tunisia | 65-69 years | 1.09 | 1.07 | 1.31 |
| Taiwan (Province of China) | 65-69 years | 1.09 | 0.91 | 1.39 |
| Slovenia | 65-69 years | 1.09 | 1.07 | 1.23 |
| Uruguay | 65-69 years | 1.11 | 1.08 | 1.24 |
| Jamaica | 65-69 years | 1.12 | 0.36 | 1.23 |
| Portugal | 65-69 years | 1.12 | 1.11 | 1.24 |
| Serbia | 65-69 years | 1.12 | 1.14 | 1.04 |
| Denmark | 65-69 years | 1.12 | 1.10 | 1.44 |
| Guyana | 65-69 years | 1.12 | 1.07 | 1.32 |
| Albania | 65-69 years | 1.14 | 1.09 | 1.34 |
| South Africa | 65-69 years | 1.14 | 2.16 | 1.28 |
| Chile | 65-69 years | 1.14 | 1.10 | 1.39 |
| Trinidad and Tobago | 65-69 years | 1.15 | 1.05 | 1.33 |
| Switzerland | 65-69 years | 1.15 | 1.12 | 1.28 |
| United States of America | 65-69 years | 1.15 | 1.21 | 1.00 |
| Bhutan | 65-69 years | 1.16 | 1.16 | 1.26 |
| Sweden | 65-69 years | 1.17 | 1.13 | 1.52 |
| Central African Republic | 65-69 years | 1.18 | 1.18 | 1.03 |
| India | 65-69 years | 1.18 | 1.18 | 1.42 |
| Belize | 65-69 years | 1.18 | 1.08 | 1.33 |
| Lebanon | 65-69 years | 1.19 | 1.19 | 1.17 |
| Greece | 65-69 years | 1.19 | 1.19 | 1.18 |
| Monaco | 65-69 years | 1.20 | 1.19 | 1.28 |
| American Samoa | 65-69 years | 1.21 | 1.21 | 1.26 |
| Qatar | 65-69 years | 1.21 | 1.19 | 1.49 |
| Republic of Moldova | 65-69 years | 1.22 | 1.19 | 1.52 |
| Liberia | 65-69 years | 1.24 | 1.26 | 1.12 |
| Nepal | 65-69 years | 1.24 | 1.25 | 1.11 |
| Bosnia and Herzegovina | 65-69 years | 1.25 | 1.26 | 1.16 |
| Israel | 65-69 years | 1.25 | 1.22 | 1.52 |
| Jordan | 65-69 years | 1.26 | 1.27 | 1.27 |
| Mozambique | 65-69 years | 1.26 | 1.26 | 1.24 |
| Yemen | 65-69 years | 1.26 | 1.27 | 1.18 |
| Turkmenistan | 65-69 years | 1.26 | 1.26 | 1.29 |
| Paraguay | 65-69 years | 1.27 | 1.25 | 1.33 |
| China | 65-69 years | 1.27 | 1.26 | 1.42 |
| Syrian Arab Republic | 65-69 years | 1.27 | 1.28 | 1.22 |
| Kuwait | 65-69 years | 1.28 | 1.28 | 1.28 |
| Kazakhstan | 65-69 years | 1.28 | 1.28 | 1.30 |
| Gabon | 65-69 years | 1.32 | 1.35 | 1.09 |
| Myanmar | 65-69 years | 1.32 | 1.28 | 1.51 |
| North Macedonia | 65-69 years | 1.32 | 1.34 | 1.13 |
| Morocco | 65-69 years | 1.32 | 1.34 | 1.16 |
| Suriname | 65-69 years | 1.32 | 1.32 | 1.33 |
| Tajikistan | 65-69 years | 1.32 | 1.35 | 1.23 |
| Sudan | 65-69 years | 1.32 | 1.34 | 1.23 |
| Iran (Islamic Republic of) | 65-69 years | 1.33 | 1.34 | 1.30 |
| Mali | 65-69 years | 1.33 | 1.36 | 1.11 |
| Peru | 65-69 years | 1.33 | 1.38 | 1.27 |
| Panama | 65-69 years | 1.34 | 1.32 | 1.35 |
| Philippines | 65-69 years | 1.35 | 1.33 | 1.41 |
| Northern Mariana Islands | 65-69 years | 1.35 | 1.34 | 1.28 |
| Azerbaijan | 65-69 years | 1.35 | 1.36 | 1.34 |
| Uzbekistan | 65-69 years | 1.36 | 1.37 | 1.26 |
| Mauritania | 65-69 years | 1.36 | 0.55 | 1.04 |
| Algeria | 65-69 years | 1.36 | 1.41 | 1.16 |
| Uganda | 65-69 years | 1.37 | 1.39 | 1.28 |
| Costa Rica | 65-69 years | 1.37 | 1.34 | 1.41 |
| Democratic People's Republic of Korea | 65-69 years | 1.37 | 1.38 | 1.17 |
| Grenada | 65-69 years | 1.37 | 1.40 | 1.31 |
| Italy | 65-69 years | 1.38 | 1.38 | 1.25 |
| Angola | 65-69 years | 1.39 | 1.42 | 1.04 |
| Puerto Rico | 65-69 years | 1.40 | 1.39 | 1.38 |
| Republic of Korea | 65-69 years | 1.40 | 1.27 | 1.88 |
| South Sudan | 65-69 years | 1.41 | 1.42 | 1.22 |
| Guatemala | 65-69 years | 1.41 | 1.55 | 1.35 |
| Egypt | 65-69 years | 1.42 | 1.44 | 1.20 |
| Malawi | 65-69 years | 1.42 | 1.44 | 1.25 |
| Haiti | 65-69 years | 1.42 | 1.45 | 1.20 |
| United States Virgin Islands | 65-69 years | 1.42 | 1.46 | 1.32 |
| Australia | 65-69 years | 1.43 | 1.42 | 1.41 |
| Belgium | 65-69 years | 1.43 | 1.42 | 1.49 |
| Iceland | 65-69 years | 1.43 | 1.49 | 0.99 |
| Dominica | 65-69 years | 1.44 | 1.49 | 1.33 |
| United Kingdom | 65-69 years | 1.45 | 1.44 | 1.44 |
| Luxembourg | 65-69 years | 1.45 | 1.46 | 1.36 |
| Bahamas | 65-69 years | 1.45 | 1.54 | 1.37 |
| Cuba | 65-69 years | 1.46 | 1.50 | 1.33 |
| Equatorial Guinea | 65-69 years | 1.46 | 1.56 | 1.03 |
| Canada | 65-69 years | 1.46 | 1.65 | 1.02 |
| Pakistan | 65-69 years | 1.47 | 1.48 | 1.20 |
| Spain | 65-69 years | 1.49 | 1.44 | 1.70 |
| Turkiye | 65-69 years | 1.49 | 1.49 | 1.42 |
| Russian Federation | 65-69 years | 1.53 | 1.50 | 1.66 |
| Cambodia | 65-69 years | 1.53 | 1.60 | 1.19 |
| Burundi | 65-69 years | 1.53 | 1.56 | 1.21 |
| Ethiopia | 65-69 years | 1.54 | 1.57 | 1.27 |
| Brazil | 65-69 years | 1.55 | 1.57 | 1.48 |
| Democratic Republic of the Congo | 65-69 years | 1.58 | 1.64 | 1.03 |
| Ecuador | 65-69 years | 1.59 | 1.73 | 1.34 |
| Somalia | 65-69 years | 1.60 | 1.62 | 1.20 |
| Singapore | 65-69 years | 1.60 | 1.58 | 1.70 |
| Lithuania | 65-69 years | 1.61 | 1.62 | 1.56 |
| Nicaragua | 65-69 years | 1.61 | 1.76 | 1.40 |
| Kyrgyzstan | 65-69 years | 1.62 | 1.65 | 1.35 |
| Estonia | 65-69 years | 1.62 | 1.60 | 1.67 |
| New Zealand | 65-69 years | 1.63 | 1.66 | 1.20 |
| Barbados | 65-69 years | 1.63 | 2.01 | 1.32 |
| Malaysia | 65-69 years | 1.63 | 1.74 | 1.41 |
| Antigua and Barbuda | 65-69 years | 1.65 | 1.88 | 1.36 |
| Dominican Republic | 65-69 years | 1.65 | 1.90 | 1.35 |
| Zambia | 65-69 years | 1.66 | 1.71 | 1.25 |
| Lao People's Democratic Republic | 65-69 years | 1.66 | 1.76 | 1.26 |
| Cyprus | 65-69 years | 1.67 | 1.66 | 1.79 |
| San Marino | 65-69 years | 1.67 | 1.68 | 1.65 |
| Madagascar | 65-69 years | 1.67 | 1.71 | 1.18 |
| Latvia | 65-69 years | 1.68 | 1.71 | 1.56 |
| Palestine | 65-69 years | 1.68 | 1.79 | 1.25 |
| Thailand | 65-69 years | 1.71 | 2.06 | 1.47 |
| Timor-Leste | 65-69 years | 1.74 | 1.84 | 1.25 |
| Kenya | 65-69 years | 1.75 | 1.85 | 1.25 |
| Rwanda | 65-69 years | 1.75 | 1.83 | 1.22 |
| Ukraine | 65-69 years | 1.76 | 1.77 | 1.68 |
| Djibouti | 65-69 years | 1.76 | 1.84 | 1.25 |
| Belarus | 65-69 years | 1.76 | 1.82 | 1.55 |
| Eritrea | 65-69 years | 1.77 | 1.82 | 1.22 |
| Oman | 65-69 years | 1.78 | 1.82 | 1.47 |
| Netherlands | 65-69 years | 1.78 | 1.86 | 1.32 |
| El Salvador | 65-69 years | 1.79 | 2.15 | 1.49 |
| United Republic of Tanzania | 65-69 years | 1.79 | 1.87 | 1.24 |
| Saint Lucia | 65-69 years | 1.80 | 2.05 | 1.36 |
| Malta | 65-69 years | 1.82 | 1.84 | 1.52 |
| Mexico | 65-69 years | 1.86 | 1.96 | 1.49 |
| Bolivia (Plurinational State of) | 65-69 years | 1.87 | 2.03 | 1.21 |
| Libya | 65-69 years | 1.88 | 2.06 | 1.19 |
| Mauritius | 65-69 years | 1.88 | 2.31 | 1.46 |
| Indonesia | 65-69 years | 1.88 | 2.11 | 1.01 |
| Comoros | 65-69 years | 1.89 | 2.00 | 1.19 |
| Congo | 65-69 years | 1.90 | 2.03 | 1.00 |
| Brunei Darussalam | 65-69 years | 1.99 | 1.97 | 2.16 |
| Armenia | 65-69 years | 2.01 | 2.16 | 1.39 |
| Maldives | 65-69 years | 2.01 | 3.14 | 1.22 |
| Ireland | 65-69 years | 2.01 | 2.07 | 1.56 |
| Greenland | 65-69 years | 2.02 | 2.47 | 1.14 |
| Bahrain | 65-69 years | 2.15 | 2.31 | 1.66 |
| Sao Tome and Principe | 65-69 years | 2.32 | 2.45 | 1.15 |
| Honduras | 65-69 years | 2.46 | 3.24 | 1.24 |
| Saudi Arabia | 65-69 years | 2.77 | 3.11 | 1.49 |
| Andorra | 65-69 years | 3.50 | 3.83 | 2.37 |
| Cook Islands | 70-74 years | 0.41 | 0.36 | 0.74 |
| Papua New Guinea | 70-74 years | 0.57 | 0.57 | 1.19 |
| Cabo Verde | 70-74 years | 0.62 | 0.54 | 1.39 |
| Burkina Faso | 70-74 years | 0.63 | 0.61 | 1.19 |
| Bermuda | 70-74 years | 0.68 | 0.52 | 1.21 |
| Croatia | 70-74 years | 0.68 | 0.62 | 1.56 |
| Vanuatu | 70-74 years | 0.70 | 0.69 | 1.25 |
| Benin | 70-74 years | 0.71 | 0.67 | 1.19 |
| Solomon Islands | 70-74 years | 0.71 | 0.70 | 1.17 |
| Guam | 70-74 years | 0.72 | 0.60 | 1.34 |
| Palau | 70-74 years | 0.80 | 0.79 | 1.09 |
| Fiji | 70-74 years | 0.82 | 0.81 | 1.23 |
| Niue | 70-74 years | 0.82 | 0.81 | 1.04 |
| Chad | 70-74 years | 0.82 | 0.81 | 1.15 |
| Romania | 70-74 years | 0.82 | 0.79 | 1.22 |
| Namibia | 70-74 years | 0.85 | 0.81 | 1.27 |
| Saint Vincent and the Grenadines | 70-74 years | 0.86 | 0.74 | 1.32 |
| Kiribati | 70-74 years | 0.86 | 0.85 | 1.22 |
| Nauru | 70-74 years | 0.86 | 0.86 | 1.00 |
| Togo | 70-74 years | 0.87 | 0.87 | 1.19 |
| Guinea-Bissau | 70-74 years | 0.87 | 0.87 | 1.16 |
| Mongolia | 70-74 years | 0.88 | 0.85 | 1.28 |
| Lesotho | 70-74 years | 0.89 | 0.87 | 1.23 |
| Afghanistan | 70-74 years | 0.89 | 0.89 | 1.13 |
| C么te d'Ivoire | 70-74 years | 0.89 | 0.87 | 1.22 |
| Denmark | 70-74 years | 0.91 | 0.87 | 1.45 |
| Ghana | 70-74 years | 0.91 | 0.88 | 1.20 |
| Sierra Leone | 70-74 years | 0.91 | 0.90 | 1.14 |
| Guinea | 70-74 years | 0.92 | 0.91 | 1.13 |
| Viet Nam | 70-74 years | 0.92 | 1.14 | 0.63 |
| Marshall Islands | 70-74 years | 0.92 | 0.92 | 1.20 |
| Niger | 70-74 years | 0.92 | 0.91 | 1.14 |
| Sri Lanka | 70-74 years | 0.93 | 1.19 | 0.53 |
| Senegal | 70-74 years | 0.93 | 0.91 | 1.17 |
| Botswana | 70-74 years | 0.93 | 0.88 | 1.29 |
| Seychelles | 70-74 years | 0.94 | 0.69 | 1.50 |
| Gambia | 70-74 years | 0.95 | 0.93 | 1.19 |
| Bulgaria | 70-74 years | 0.96 | 0.95 | 1.05 |
| Eswatini | 70-74 years | 0.96 | 0.94 | 1.24 |
| Germany | 70-74 years | 0.96 | 0.94 | 1.34 |
| France | 70-74 years | 0.97 | 0.91 | 1.29 |
| Iraq | 70-74 years | 0.98 | 0.96 | 1.24 |
| Finland | 70-74 years | 0.98 | 0.95 | 1.47 |
| Hungary | 70-74 years | 0.99 | 0.95 | 1.30 |
| Nigeria | 70-74 years | 1.00 | 0.97 | 1.21 |
| Slovenia | 70-74 years | 1.00 | 0.97 | 1.29 |
| Tunisia | 70-74 years | 1.00 | 0.98 | 1.26 |
| Montenegro | 70-74 years | 1.01 | 1.00 | 1.10 |
| Cameroon | 70-74 years | 1.01 | 1.00 | 1.17 |
| Czechia | 70-74 years | 1.01 | 0.99 | 1.19 |
| Poland | 70-74 years | 1.03 | 1.03 | 0.99 |
| Lebanon | 70-74 years | 1.04 | 1.02 | 1.15 |
| Argentina | 70-74 years | 1.04 | 0.99 | 1.37 |
| Yemen | 70-74 years | 1.04 | 1.04 | 1.18 |
| Samoa | 70-74 years | 1.04 | 1.04 | 1.23 |
| Turkmenistan | 70-74 years | 1.05 | 1.03 | 1.30 |
| Tokelau | 70-74 years | 1.05 | 1.03 | 1.25 |
| Jamaica | 70-74 years | 1.05 | 0.98 | 1.24 |
| Tuvalu | 70-74 years | 1.06 | 1.05 | 1.25 |
| Kuwait | 70-74 years | 1.06 | 0.98 | 1.23 |
| Uruguay | 70-74 years | 1.06 | 1.02 | 1.27 |
| Japan | 70-74 years | 1.06 | 0.94 | 1.80 |
| Micronesia (Federated States of) | 70-74 years | 1.07 | 1.07 | 1.22 |
| Zimbabwe | 70-74 years | 1.07 | 1.07 | 1.21 |
| Belize | 70-74 years | 1.07 | 0.93 | 1.34 |
| Qatar | 70-74 years | 1.08 | 1.02 | 1.65 |
| Georgia | 70-74 years | 1.08 | 1.06 | 1.45 |
| Trinidad and Tobago | 70-74 years | 1.09 | 0.98 | 1.33 |
| Venezuela (Bolivarian Republic of) | 70-74 years | 1.09 | 1.04 | 1.39 |
| Guyana | 70-74 years | 1.09 | 1.03 | 1.31 |
| Tonga | 70-74 years | 1.11 | 1.09 | 1.26 |
| Colombia | 70-74 years | 1.11 | 1.00 | 1.42 |
| Kazakhstan | 70-74 years | 1.11 | 1.10 | 1.27 |
| Paraguay | 70-74 years | 1.11 | 1.04 | 1.35 |
| Morocco | 70-74 years | 1.11 | 1.11 | 1.17 |
| Liberia | 70-74 years | 1.11 | 1.12 | 1.12 |
| Tajikistan | 70-74 years | 1.12 | 1.10 | 1.23 |
| India | 70-74 years | 1.13 | 1.13 | 1.42 |
| Syrian Arab Republic | 70-74 years | 1.14 | 1.14 | 1.22 |
| Bangladesh | 70-74 years | 1.15 | 1.15 | 1.53 |
| Slovakia | 70-74 years | 1.15 | 1.19 | 1.00 |
| Norway | 70-74 years | 1.16 | 1.15 | 1.16 |
| Myanmar | 70-74 years | 1.16 | 1.11 | 1.52 |
| Sudan | 70-74 years | 1.16 | 1.16 | 1.23 |
| Bhutan | 70-74 years | 1.16 | 1.17 | 1.26 |
| Greece | 70-74 years | 1.17 | 1.18 | 1.14 |
| Philippines | 70-74 years | 1.17 | 1.13 | 1.42 |
| Austria | 70-74 years | 1.18 | 1.14 | 1.42 |
| Central African Republic | 70-74 years | 1.18 | 1.19 | 1.03 |
| China | 70-74 years | 1.19 | 1.18 | 1.44 |
| Nepal | 70-74 years | 1.19 | 1.19 | 1.10 |
| Mozambique | 70-74 years | 1.20 | 1.20 | 1.25 |
| Costa Rica | 70-74 years | 1.20 | 1.10 | 1.41 |
| Albania | 70-74 years | 1.21 | 1.19 | 1.35 |
| Mali | 70-74 years | 1.21 | 1.22 | 1.11 |
| Algeria | 70-74 years | 1.22 | 1.23 | 1.16 |
| Cuba | 70-74 years | 1.22 | 1.18 | 1.34 |
| Azerbaijan | 70-74 years | 1.22 | 1.21 | 1.34 |
| Brazil | 70-74 years | 1.23 | 1.18 | 1.49 |
| Saint Kitts and Nevis | 70-74 years | 1.24 | 1.17 | 1.57 |
| Mauritania | 70-74 years | 1.24 | 1.27 | 1.05 |
| Serbia | 70-74 years | 1.25 | 1.27 | 1.06 |
| Monaco | 70-74 years | 1.26 | 1.25 | 1.28 |
| Panama | 70-74 years | 1.26 | 1.20 | 1.36 |
| Australia | 70-74 years | 1.27 | 1.26 | 1.29 |
| Suriname | 70-74 years | 1.27 | 1.25 | 1.33 |
| Switzerland | 70-74 years | 1.28 | 1.28 | 1.28 |
| Uzbekistan | 70-74 years | 1.28 | 1.29 | 1.27 |
| South Africa | 70-74 years | 1.29 | 1.30 | 1.29 |
| North Macedonia | 70-74 years | 1.29 | 1.32 | 1.12 |
| Taiwan (Province of China) | 70-74 years | 1.29 | 1.25 | 1.34 |
| Iran (Islamic Republic of) | 70-74 years | 1.30 | 1.30 | 1.29 |
| Cambodia | 70-74 years | 1.31 | 1.32 | 1.19 |
| United States of America | 70-74 years | 1.31 | 1.46 | 1.04 |
| Canada | 70-74 years | 1.31 | 1.39 | 1.06 |
| Turkiye | 70-74 years | 1.31 | 1.31 | 1.37 |
| Jordan | 70-74 years | 1.31 | 1.34 | 1.24 |
| Democratic People's Republic of Korea | 70-74 years | 1.32 | 1.33 | 1.18 |
| Haiti | 70-74 years | 1.33 | 1.34 | 1.20 |
| Sweden | 70-74 years | 1.34 | 1.31 | 1.49 |
| Ecuador | 70-74 years | 1.34 | 1.34 | 1.35 |
| Grenada | 70-74 years | 1.34 | 1.34 | 1.31 |
| Bahamas | 70-74 years | 1.35 | 1.34 | 1.38 |
| United Kingdom | 70-74 years | 1.36 | 1.33 | 1.47 |
| American Samoa | 70-74 years | 1.38 | 1.39 | 1.27 |
| Singapore | 70-74 years | 1.38 | 1.35 | 1.63 |
| Chile | 70-74 years | 1.39 | 1.39 | 1.39 |
| Republic of Korea | 70-74 years | 1.39 | 1.29 | 1.88 |
| United States Virgin Islands | 70-74 years | 1.40 | 1.42 | 1.33 |
| Angola | 70-74 years | 1.40 | 1.43 | 1.04 |
| Malawi | 70-74 years | 1.41 | 1.42 | 1.26 |
| Oman | 70-74 years | 1.41 | 1.42 | 1.35 |
| Bosnia and Herzegovina | 70-74 years | 1.41 | 1.45 | 1.18 |
| Uganda | 70-74 years | 1.42 | 1.44 | 1.29 |
| South Sudan | 70-74 years | 1.42 | 1.44 | 1.23 |
| Kyrgyzstan | 70-74 years | 1.43 | 1.44 | 1.36 |
| Luxembourg | 70-74 years | 1.43 | 1.43 | 1.35 |
| Puerto Rico | 70-74 years | 1.43 | 1.46 | 1.34 |
| Republic of Moldova | 70-74 years | 1.44 | 1.43 | 1.51 |
| Gabon | 70-74 years | 1.44 | 1.50 | 1.09 |
| Spain | 70-74 years | 1.45 | 1.39 | 1.78 |
| Timor-Leste | 70-74 years | 1.45 | 1.48 | 1.25 |
| Guatemala | 70-74 years | 1.45 | 1.64 | 1.34 |
| Burundi | 70-74 years | 1.45 | 1.47 | 1.22 |
| Italy | 70-74 years | 1.46 | 1.48 | 1.29 |
| Egypt | 70-74 years | 1.46 | 1.48 | 1.20 |
| Peru | 70-74 years | 1.47 | 1.58 | 1.27 |
| Nicaragua | 70-74 years | 1.47 | 1.52 | 1.40 |
| Lao People's Democratic Republic | 70-74 years | 1.47 | 1.50 | 1.27 |
| Belgium | 70-74 years | 1.48 | 1.47 | 1.50 |
| Cyprus | 70-74 years | 1.48 | 1.47 | 1.77 |
| Portugal | 70-74 years | 1.49 | 1.53 | 1.22 |
| Madagascar | 70-74 years | 1.50 | 1.51 | 1.19 |
| Dominica | 70-74 years | 1.51 | 1.57 | 1.34 |
| Equatorial Guinea | 70-74 years | 1.53 | 1.63 | 1.03 |
| Ethiopia | 70-74 years | 1.54 | 1.56 | 1.27 |
| Somalia | 70-74 years | 1.54 | 1.56 | 1.21 |
| Pakistan | 70-74 years | 1.55 | 1.55 | 1.20 |
| Netherlands | 70-74 years | 1.55 | 1.59 | 1.33 |
| Barbados | 70-74 years | 1.56 | 1.80 | 1.32 |
| Malaysia | 70-74 years | 1.57 | 1.62 | 1.41 |
| Dominican Republic | 70-74 years | 1.57 | 1.72 | 1.36 |
| Libya | 70-74 years | 1.59 | 1.66 | 1.19 |
| Indonesia | 70-74 years | 1.59 | 1.75 | 0.87 |
| Saint Lucia | 70-74 years | 1.59 | 1.68 | 1.37 |
| San Marino | 70-74 years | 1.60 | 1.61 | 1.61 |
| Israel | 70-74 years | 1.60 | 1.60 | 1.59 |
| Democratic Republic of the Congo | 70-74 years | 1.61 | 1.65 | 1.03 |
| Northern Mariana Islands | 70-74 years | 1.61 | 1.68 | 1.29 |
| Rwanda | 70-74 years | 1.66 | 1.71 | 1.23 |
| Antigua and Barbuda | 70-74 years | 1.66 | 1.89 | 1.36 |
| Latvia | 70-74 years | 1.70 | 1.75 | 1.54 |
| Iceland | 70-74 years | 1.70 | 1.81 | 0.93 |
| Lithuania | 70-74 years | 1.71 | 1.76 | 1.55 |
| Zambia | 70-74 years | 1.71 | 1.76 | 1.26 |
| Djibouti | 70-74 years | 1.71 | 1.76 | 1.26 |
| El Salvador | 70-74 years | 1.73 | 2.00 | 1.48 |
| Eritrea | 70-74 years | 1.73 | 1.77 | 1.22 |
| Palestine | 70-74 years | 1.77 | 1.86 | 1.25 |
| Russian Federation | 70-74 years | 1.78 | 1.81 | 1.65 |
| United Republic of Tanzania | 70-74 years | 1.81 | 1.87 | 1.25 |
| Thailand | 70-74 years | 1.81 | 2.14 | 1.47 |
| New Zealand | 70-74 years | 1.81 | 1.91 | 1.10 |
| Comoros | 70-74 years | 1.84 | 1.91 | 1.20 |
| Mauritius | 70-74 years | 1.84 | 2.16 | 1.46 |
| Malta | 70-74 years | 1.86 | 1.90 | 1.48 |
| Kenya | 70-74 years | 1.86 | 1.95 | 1.26 |
| Belarus | 70-74 years | 1.86 | 1.96 | 1.53 |
| Congo | 70-74 years | 1.93 | 2.02 | 1.00 |
| Ukraine | 70-74 years | 1.93 | 2.00 | 1.68 |
| Maldives | 70-74 years | 1.97 | 2.69 | 1.23 |
| Armenia | 70-74 years | 1.99 | 2.13 | 1.40 |
| Estonia | 70-74 years | 2.00 | 2.19 | 1.64 |
| Ireland | 70-74 years | 2.02 | 2.08 | 1.56 |
| Mexico | 70-74 years | 2.06 | 2.23 | 1.47 |
| Bolivia (Plurinational State of) | 70-74 years | 2.08 | 2.24 | 1.21 |
| Bahrain | 70-74 years | 2.09 | 2.23 | 1.62 |
| Brunei Darussalam | 70-74 years | 2.10 | 2.12 | 1.89 |
| Honduras | 70-74 years | 2.11 | 2.51 | 1.24 |
| Greenland | 70-74 years | 2.18 | 2.75 | 1.21 |
| Sao Tome and Principe | 70-74 years | 2.22 | 2.33 | 1.15 |
| Saudi Arabia | 70-74 years | 2.22 | 2.46 | 1.42 |
| Andorra | 70-74 years | 3.28 | 3.56 | 2.38 |
| United Arab Emirates | 70-74 years | 5.50 | 6.06 | 1.21 |
| Cook Islands | 75-79 years | 0.37 | 0.31 | 0.73 |
| Costa Rica | 75-79 years | 0.40 | 1.01 | 1.41 |
| Papua New Guinea | 75-79 years | 0.42 | 0.41 | 1.20 |
| Guam | 75-79 years | 0.56 | 0.44 | 1.34 |
| Solomon Islands | 75-79 years | 0.56 | 0.56 | 1.17 |
| Vanuatu | 75-79 years | 0.58 | 0.57 | 1.25 |
| Burkina Faso | 75-79 years | 0.60 | 0.59 | 1.19 |
| Cabo Verde | 75-79 years | 0.62 | 0.57 | 1.39 |
| Fiji | 75-79 years | 0.63 | 0.62 | 1.23 |
| Benin | 75-79 years | 0.68 | 0.66 | 1.20 |
| Bermuda | 75-79 years | 0.70 | 0.55 | 1.22 |
| Niue | 75-79 years | 0.72 | 0.70 | 1.01 |
| Nauru | 75-79 years | 0.73 | 0.72 | 0.95 |
| Palau | 75-79 years | 0.73 | 0.72 | 1.07 |
| Kiribati | 75-79 years | 0.74 | 0.73 | 1.22 |
| Chad | 75-79 years | 0.76 | 0.75 | 1.15 |
| Marshall Islands | 75-79 years | 0.76 | 0.75 | 1.20 |
| Republic of Korea | 75-79 years | 0.76 | 1.69 | 1.87 |
| United States Virgin Islands | 75-79 years | 0.77 | 1.28 | 1.36 |
| Grenada | 75-79 years | 0.79 | 0.74 | 1.31 |
| Sri Lanka | 75-79 years | 0.82 | 1.10 | 0.45 |
| Sierra Leone | 75-79 years | 0.83 | 0.82 | 1.15 |
| Saint Vincent and the Grenadines | 75-79 years | 0.84 | 0.76 | 1.32 |
| Niger | 75-79 years | 0.84 | 0.83 | 1.14 |
| Guinea-Bissau | 75-79 years | 0.84 | 0.84 | 1.16 |
| C么te d'Ivoire | 75-79 years | 0.85 | 0.83 | 1.23 |
| Guinea | 75-79 years | 0.85 | 0.84 | 1.14 |
| Togo | 75-79 years | 0.85 | 0.84 | 1.19 |
| Samoa | 75-79 years | 0.87 | 0.86 | 1.23 |
| Tonga | 75-79 years | 0.87 | 0.83 | 1.27 |
| Tokelau | 75-79 years | 0.89 | 0.88 | 1.17 |
| Tuvalu | 75-79 years | 0.89 | 0.88 | 1.15 |
| Senegal | 75-79 years | 0.89 | 0.88 | 1.17 |
| Denmark | 75-79 years | 0.91 | 0.88 | 1.46 |
| Gambia | 75-79 years | 0.92 | 0.91 | 1.20 |
| Afghanistan | 75-79 years | 0.92 | 0.92 | 1.13 |
| Micronesia (Federated States of) | 75-79 years | 0.93 | 0.93 | 1.22 |
| Finland | 75-79 years | 0.95 | 0.92 | 1.50 |
| Ghana | 75-79 years | 0.96 | 0.94 | 1.20 |
| Cameroon | 75-79 years | 0.96 | 0.95 | 1.18 |
| Paraguay | 75-79 years | 0.97 | 0.89 | 1.35 |
| Jamaica | 75-79 years | 0.99 | 0.93 | 1.25 |
| Namibia | 75-79 years | 1.00 | 0.98 | 1.27 |
| Georgia | 75-79 years | 1.00 | 0.99 | 1.45 |
| Botswana | 75-79 years | 1.02 | 1.00 | 1.29 |
| Nigeria | 75-79 years | 1.02 | 1.02 | 1.21 |
| Myanmar | 75-79 years | 1.03 | 0.98 | 1.52 |
| Turkmenistan | 75-79 years | 1.03 | 1.01 | 1.30 |
| Lesotho | 75-79 years | 1.03 | 1.04 | 1.23 |
| Liberia | 75-79 years | 1.04 | 1.04 | 1.13 |
| Tunisia | 75-79 years | 1.04 | 1.03 | 1.23 |
| Philippines | 75-79 years | 1.05 | 1.00 | 1.42 |
| Panama | 75-79 years | 1.05 | 1.17 | 1.36 |
| Guyana | 75-79 years | 1.05 | 1.00 | 1.31 |
| Romania | 75-79 years | 1.05 | 1.05 | 1.23 |
| Qatar | 75-79 years | 1.06 | 0.95 | 2.06 |
| Tajikistan | 75-79 years | 1.07 | 1.06 | 1.24 |
| Yemen | 75-79 years | 1.08 | 1.08 | 1.18 |
| Trinidad and Tobago | 75-79 years | 1.08 | 1.00 | 1.33 |
| Brazil | 75-79 years | 1.08 | 1.03 | 1.50 |
| Iraq | 75-79 years | 1.08 | 1.08 | 1.24 |
| China | 75-79 years | 1.09 | 1.09 | 1.45 |
| Eswatini | 75-79 years | 1.09 | 1.09 | 1.24 |
| Uruguay | 75-79 years | 1.10 | 1.07 | 1.28 |
| Kuwait | 75-79 years | 1.11 | 1.09 | 1.18 |
| Colombia | 75-79 years | 1.11 | 1.02 | 1.40 |
| Cambodia | 75-79 years | 1.12 | 1.12 | 1.19 |
| Zimbabwe | 75-79 years | 1.12 | 1.12 | 1.22 |
| India | 75-79 years | 1.12 | 1.13 | 1.42 |
| France | 75-79 years | 1.13 | 1.10 | 1.27 |
| Croatia | 75-79 years | 1.13 | 1.10 | 1.69 |
| Ecuador | 75-79 years | 1.13 | 1.09 | 1.35 |
| Czechia | 75-79 years | 1.14 | 1.13 | 1.22 |
| Belize | 75-79 years | 1.14 | 1.05 | 1.34 |
| Mongolia | 75-79 years | 1.14 | 1.13 | 1.29 |
| Uzbekistan | 75-79 years | 1.16 | 1.16 | 1.27 |
| Argentina | 75-79 years | 1.16 | 1.15 | 1.37 |
| Japan | 75-79 years | 1.16 | 1.10 | 1.76 |
| Germany | 75-79 years | 1.16 | 1.15 | 1.39 |
| Central African Republic | 75-79 years | 1.16 | 1.17 | 1.03 |
| Chile | 75-79 years | 1.16 | 1.14 | 1.39 |
| Bulgaria | 75-79 years | 1.18 | 1.19 | 1.06 |
| Montenegro | 75-79 years | 1.18 | 1.19 | 1.09 |
| Mozambique | 75-79 years | 1.19 | 1.19 | 1.26 |
| Lebanon | 75-79 years | 1.19 | 1.19 | 1.15 |
| American Samoa | 75-79 years | 1.19 | 1.19 | 1.26 |
| Mauritania | 75-79 years | 1.19 | 1.20 | 1.06 |
| Bangladesh | 75-79 years | 1.20 | 1.20 | 1.53 |
| Morocco | 75-79 years | 1.20 | 1.21 | 1.17 |
| Mali | 75-79 years | 1.20 | 1.21 | 1.12 |
| Sudan | 75-79 years | 1.20 | 1.20 | 1.24 |
| Poland | 75-79 years | 1.21 | 1.23 | 1.03 |
| Bhutan | 75-79 years | 1.21 | 1.21 | 1.26 |
| Timor-Leste | 75-79 years | 1.22 | 1.21 | 1.25 |
| Seychelles | 75-79 years | 1.22 | 0.44 | 1.49 |
| Venezuela (Bolivarian Republic of) | 75-79 years | 1.23 | 1.20 | 1.37 |
| Azerbaijan | 75-79 years | 1.24 | 1.23 | 1.34 |
| Hungary | 75-79 years | 1.25 | 1.25 | 1.35 |
| Saint Kitts and Nevis | 75-79 years | 1.25 | 1.19 | 1.56 |
| Slovenia | 75-79 years | 1.26 | 1.26 | 1.31 |
| Democratic People's Republic of Korea | 75-79 years | 1.26 | 1.27 | 1.18 |
| Norway | 75-79 years | 1.26 | 1.26 | 1.18 |
| Nepal | 75-79 years | 1.27 | 1.27 | 1.10 |
| United States of America | 75-79 years | 1.27 | 1.39 | 1.06 |
| Cuba | 75-79 years | 1.28 | 1.28 | 1.35 |
| Guatemala | 75-79 years | 1.28 | 1.23 | 1.34 |
| Slovakia | 75-79 years | 1.28 | 1.36 | 1.00 |
| Lao People's Democratic Republic | 75-79 years | 1.29 | 1.30 | 1.27 |
| Luxembourg | 75-79 years | 1.30 | 1.30 | 1.32 |
| South Sudan | 75-79 years | 1.30 | 1.31 | 1.24 |
| Monaco | 75-79 years | 1.31 | 1.33 | 1.25 |
| Angola | 75-79 years | 1.31 | 1.33 | 1.04 |
| Malawi | 75-79 years | 1.31 | 1.32 | 1.26 |
| Taiwan (Province of China) | 75-79 years | 1.32 | 1.34 | 1.29 |
| Madagascar | 75-79 years | 1.33 | 1.34 | 1.20 |
| Northern Mariana Islands | 75-79 years | 1.34 | 1.33 | 1.29 |
| Austria | 75-79 years | 1.34 | 1.33 | 1.43 |
| Kyrgyzstan | 75-79 years | 1.35 | 1.35 | 1.37 |
| Uganda | 75-79 years | 1.36 | 1.37 | 1.29 |
| Suriname | 75-79 years | 1.36 | 1.39 | 1.33 |
| Viet Nam | 75-79 years | 1.37 | 1.10 | 0.54 |
| Burundi | 75-79 years | 1.37 | 1.38 | 1.23 |
| Indonesia | 75-79 years | 1.38 | 1.54 | 0.69 |
| Kazakhstan | 75-79 years | 1.38 | 1.42 | 1.22 |
| Peru | 75-79 years | 1.38 | 1.43 | 1.27 |
| Algeria | 75-79 years | 1.38 | 1.41 | 1.16 |
| North Macedonia | 75-79 years | 1.39 | 1.42 | 1.10 |
| Nicaragua | 75-79 years | 1.40 | 1.40 | 1.39 |
| South Africa | 75-79 years | 1.40 | 1.43 | 1.29 |
| Greece | 75-79 years | 1.40 | 1.45 | 1.08 |
| Oman | 75-79 years | 1.41 | 1.42 | 1.32 |
| Australia | 75-79 years | 1.41 | 1.43 | 1.20 |
| Serbia | 75-79 years | 1.41 | 1.47 | 1.04 |
| Bahamas | 75-79 years | 1.41 | 1.45 | 1.37 |
| Canada | 75-79 years | 1.42 | 1.54 | 1.07 |
| Albania | 75-79 years | 1.43 | 1.44 | 1.37 |
| Sweden | 75-79 years | 1.44 | 1.43 | 1.45 |
| Malaysia | 75-79 years | 1.44 | 1.46 | 1.40 |
| Equatorial Guinea | 75-79 years | 1.45 | 1.50 | 1.03 |
| Gabon | 75-79 years | 1.45 | 1.50 | 1.08 |
| Singapore | 75-79 years | 1.45 | 1.45 | 1.56 |
| Syrian Arab Republic | 75-79 years | 1.46 | 1.48 | 1.22 |
| Iceland | 75-79 years | 1.47 | 1.54 | 0.87 |
| Ethiopia | 75-79 years | 1.48 | 1.49 | 1.28 |
| Jordan | 75-79 years | 1.48 | 1.55 | 1.21 |
| Somalia | 75-79 years | 1.49 | 1.50 | 1.21 |
| Turkiye | 75-79 years | 1.49 | 1.52 | 1.32 |
| New Zealand | 75-79 years | 1.49 | 1.56 | 1.04 |
| Puerto Rico | 75-79 years | 1.50 | 1.60 | 1.32 |
| Switzerland | 75-79 years | 1.50 | 1.55 | 1.26 |
| Democratic Republic of the Congo | 75-79 years | 1.50 | 1.53 | 1.03 |
| Dominica | 75-79 years | 1.51 | 1.56 | 1.34 |
| Pakistan | 75-79 years | 1.51 | 1.51 | 1.20 |
| Italy | 75-79 years | 1.52 | 1.54 | 1.30 |
| Israel | 75-79 years | 1.53 | 1.54 | 1.54 |
| Netherlands | 75-79 years | 1.54 | 1.56 | 1.30 |
| Barbados | 75-79 years | 1.54 | 1.69 | 1.32 |
| Iran (Islamic Republic of) | 75-79 years | 1.55 | 1.58 | 1.29 |
| Belgium | 75-79 years | 1.55 | 1.56 | 1.49 |
| Portugal | 75-79 years | 1.56 | 1.62 | 1.16 |
| Saint Lucia | 75-79 years | 1.57 | 1.64 | 1.37 |
| Rwanda | 75-79 years | 1.57 | 1.60 | 1.24 |
| Libya | 75-79 years | 1.58 | 1.63 | 1.19 |
| Dominican Republic | 75-79 years | 1.58 | 1.70 | 1.36 |
| Zambia | 75-79 years | 1.59 | 1.61 | 1.27 |
| United Kingdom | 75-79 years | 1.60 | 1.63 | 1.45 |
| Djibouti | 75-79 years | 1.61 | 1.64 | 1.26 |
| San Marino | 75-79 years | 1.61 | 1.64 | 1.52 |
| United Republic of Tanzania | 75-79 years | 1.64 | 1.67 | 1.25 |
| Antigua and Barbuda | 75-79 years | 1.66 | 1.80 | 1.36 |
| Egypt | 75-79 years | 1.66 | 1.69 | 1.20 |
| Comoros | 75-79 years | 1.67 | 1.70 | 1.21 |
| Spain | 75-79 years | 1.68 | 1.66 | 1.77 |
| Thailand | 75-79 years | 1.68 | 1.83 | 1.47 |
| Eritrea | 75-79 years | 1.70 | 1.72 | 1.23 |
| El Salvador | 75-79 years | 1.70 | 1.89 | 1.47 |
| Cyprus | 75-79 years | 1.72 | 1.75 | 1.62 |
| Kenya | 75-79 years | 1.77 | 1.81 | 1.26 |
| Republic of Moldova | 75-79 years | 1.77 | 1.87 | 1.48 |
| Palestine | 75-79 years | 1.79 | 1.85 | 1.25 |
| Congo | 75-79 years | 1.80 | 1.86 | 1.00 |
| Maldives | 75-79 years | 1.85 | 2.23 | 1.24 |
| Bosnia and Herzegovina | 75-79 years | 1.86 | 1.97 | 1.17 |
| Ukraine | 75-79 years | 1.88 | 2.00 | 1.64 |
| Mauritius | 75-79 years | 1.89 | 2.13 | 1.45 |
| Mexico | 75-79 years | 1.91 | 2.03 | 1.44 |
| Honduras | 75-79 years | 1.99 | 2.24 | 1.24 |
| Bahrain | 75-79 years | 1.99 | 2.06 | 1.62 |
| Bolivia (Plurinational State of) | 75-79 years | 2.02 | 2.11 | 1.21 |
| Ireland | 75-79 years | 2.02 | 2.09 | 1.47 |
| Greenland | 75-79 years | 2.03 | 2.51 | 1.24 |
| Brunei Darussalam | 75-79 years | 2.11 | 2.16 | 1.70 |
| Sao Tome and Principe | 75-79 years | 2.13 | 2.20 | 1.16 |
| Belarus | 75-79 years | 2.19 | 2.49 | 1.51 |
| Russian Federation | 75-79 years | 2.20 | 2.36 | 1.61 |
| Estonia | 75-79 years | 2.23 | 2.70 | 1.60 |
| Saudi Arabia | 75-79 years | 2.31 | 2.59 | 1.37 |
| Malta | 75-79 years | 2.31 | 2.40 | 1.38 |
| Lithuania | 75-79 years | 2.41 | 2.91 | 1.53 |
| Latvia | 75-79 years | 2.59 | 3.22 | 1.51 |
| Armenia | 75-79 years | 2.71 | 2.94 | 1.41 |
| Andorra | 75-79 years | 3.34 | 3.68 | 2.25 |
| Haiti | 75-79 years | 4.98 | 1.19 | 1.20 |
| United Arab Emirates | 75-79 years | 6.75 | 7.04 | 1.21 |
| Papua New Guinea | 80-84 years | 0.35 | 0.34 | 1.21 |
| Cook Islands | 80-84 years | 0.35 | 0.29 | 0.73 |
| Guam | 80-84 years | 0.35 | 0.23 | 1.33 |
| Burkina Faso | 80-84 years | 0.47 | 0.46 | 1.19 |
| Solomon Islands | 80-84 years | 0.48 | 0.47 | 1.18 |
| Vanuatu | 80-84 years | 0.51 | 0.50 | 1.25 |
| Fiji | 80-84 years | 0.52 | 0.52 | 1.23 |
| Benin | 80-84 years | 0.53 | 0.51 | 1.20 |
| Cabo Verde | 80-84 years | 0.53 | 0.49 | 1.39 |
| Chad | 80-84 years | 0.58 | 0.57 | 1.16 |
| Nauru | 80-84 years | 0.62 | 0.60 | 0.93 |
| Niger | 80-84 years | 0.62 | 0.61 | 1.15 |
| Sierra Leone | 80-84 years | 0.63 | 0.62 | 1.15 |
| Guinea | 80-84 years | 0.65 | 0.64 | 1.15 |
| Togo | 80-84 years | 0.66 | 0.64 | 1.20 |
| Niue | 80-84 years | 0.66 | 0.64 | 1.01 |
| Marshall Islands | 80-84 years | 0.66 | 0.65 | 1.20 |
| C么te d'Ivoire | 80-84 years | 0.67 | 0.65 | 1.23 |
| Guinea-Bissau | 80-84 years | 0.67 | 0.66 | 1.16 |
| Kiribati | 80-84 years | 0.68 | 0.68 | 1.23 |
| Gambia | 80-84 years | 0.70 | 0.69 | 1.20 |
| Senegal | 80-84 years | 0.70 | 0.68 | 1.17 |
| Seychelles | 80-84 years | 0.70 | 0.54 | 1.48 |
| Cameroon | 80-84 years | 0.72 | 0.71 | 1.18 |
| Namibia | 80-84 years | 0.72 | 0.70 | 1.27 |
| Botswana | 80-84 years | 0.73 | 0.70 | 1.28 |
| Lesotho | 80-84 years | 0.73 | 0.72 | 1.24 |
| Samoa | 80-84 years | 0.75 | 0.74 | 1.23 |
| Tokelau | 80-84 years | 0.75 | 0.72 | 1.15 |
| Palau | 80-84 years | 0.75 | 0.74 | 1.07 |
| Sri Lanka | 80-84 years | 0.76 | 1.02 | 0.38 |
| Liberia | 80-84 years | 0.77 | 0.75 | 1.13 |
| Zimbabwe | 80-84 years | 0.77 | 0.76 | 1.22 |
| Ghana | 80-84 years | 0.78 | 0.75 | 1.20 |
| Tonga | 80-84 years | 0.78 | 0.75 | 1.27 |
| Viet Nam | 80-84 years | 0.79 | 1.03 | 0.47 |
| Tuvalu | 80-84 years | 0.79 | 0.78 | 1.11 |
| Eswatini | 80-84 years | 0.80 | 0.78 | 1.23 |
| Afghanistan | 80-84 years | 0.80 | 0.79 | 1.14 |
| Micronesia (Federated States of) | 80-84 years | 0.81 | 0.81 | 1.22 |
| Nigeria | 80-84 years | 0.83 | 0.81 | 1.21 |
| Bermuda | 80-84 years | 0.85 | 0.69 | 1.24 |
| Mali | 80-84 years | 0.86 | 0.85 | 1.12 |
| Turkmenistan | 80-84 years | 0.86 | 0.84 | 1.30 |
| Myanmar | 80-84 years | 0.88 | 0.84 | 1.53 |
| Saint Vincent and the Grenadines | 80-84 years | 0.88 | 0.81 | 1.32 |
| Mauritania | 80-84 years | 0.91 | 0.91 | 1.07 |
| Yemen | 80-84 years | 0.95 | 0.94 | 1.19 |
| Philippines | 80-84 years | 0.95 | 0.91 | 1.43 |
| Finland | 80-84 years | 0.95 | 0.93 | 1.51 |
| Cambodia | 80-84 years | 0.97 | 0.96 | 1.19 |
| Tunisia | 80-84 years | 0.97 | 0.95 | 1.19 |
| Tajikistan | 80-84 years | 0.97 | 0.96 | 1.25 |
| Denmark | 80-84 years | 0.99 | 0.98 | 1.48 |
| Bangladesh | 80-84 years | 1.00 | 0.99 | 1.54 |
| Timor-Leste | 80-84 years | 1.00 | 0.98 | 1.26 |
| Iraq | 80-84 years | 1.02 | 1.01 | 1.24 |
| China | 80-84 years | 1.02 | 1.01 | 1.48 |
| Grenada | 80-84 years | 1.02 | 1.02 | 1.31 |
| Sudan | 80-84 years | 1.02 | 1.02 | 1.24 |
| Bhutan | 80-84 years | 1.02 | 1.03 | 1.26 |
| Central African Republic | 80-84 years | 1.04 | 1.04 | 1.03 |
| Lebanon | 80-84 years | 1.04 | 1.03 | 1.15 |
| Kyrgyzstan | 80-84 years | 1.04 | 1.03 | 1.37 |
| South Africa | 80-84 years | 1.04 | 1.03 | 1.29 |
| Uzbekistan | 80-84 years | 1.04 | 1.04 | 1.27 |
| Brazil | 80-84 years | 1.04 | 0.99 | 1.50 |
| Georgia | 80-84 years | 1.05 | 1.04 | 1.44 |
| India | 80-84 years | 1.05 | 1.05 | 1.41 |
| Switzerland | 80-84 years | 1.05 | 1.03 | 1.23 |
| Qatar | 80-84 years | 1.05 | 0.89 | 2.34 |
| Lao People's Democratic Republic | 80-84 years | 1.07 | 1.05 | 1.27 |
| Kuwait | 80-84 years | 1.08 | 1.06 | 1.13 |
| Paraguay | 80-84 years | 1.09 | 1.04 | 1.36 |
| American Samoa | 80-84 years | 1.10 | 1.10 | 1.26 |
| Japan | 80-84 years | 1.10 | 1.07 | 1.68 |
| Luxembourg | 80-84 years | 1.11 | 1.09 | 1.27 |
| France | 80-84 years | 1.11 | 1.10 | 1.23 |
| Morocco | 80-84 years | 1.12 | 1.12 | 1.17 |
| Colombia | 80-84 years | 1.12 | 1.01 | 1.38 |
| Jamaica | 80-84 years | 1.13 | 1.10 | 1.25 |
| Angola | 80-84 years | 1.14 | 1.15 | 1.04 |
| Kazakhstan | 80-84 years | 1.14 | 1.15 | 1.17 |
| Democratic People's Republic of Korea | 80-84 years | 1.15 | 1.15 | 1.18 |
| Austria | 80-84 years | 1.15 | 1.14 | 1.41 |
| Chile | 80-84 years | 1.15 | 1.13 | 1.37 |
| Costa Rica | 80-84 years | 1.15 | 1.09 | 1.41 |
| Venezuela (Bolivarian Republic of) | 80-84 years | 1.15 | 1.11 | 1.34 |
| Azerbaijan | 80-84 years | 1.16 | 1.14 | 1.34 |
| Monaco | 80-84 years | 1.16 | 1.15 | 1.21 |
| Nepal | 80-84 years | 1.16 | 1.16 | 1.09 |
| Indonesia | 80-84 years | 1.16 | 1.30 | 0.50 |
| Taiwan (Province of China) | 80-84 years | 1.18 | 1.17 | 1.22 |
| Mongolia | 80-84 years | 1.19 | 1.18 | 1.29 |
| Romania | 80-84 years | 1.19 | 1.20 | 1.21 |
| New Zealand | 80-84 years | 1.22 | 1.24 | 0.98 |
| Germany | 80-84 years | 1.22 | 1.22 | 1.41 |
| Panama | 80-84 years | 1.23 | 1.18 | 1.36 |
| South Sudan | 80-84 years | 1.24 | 1.24 | 1.25 |
| Sweden | 80-84 years | 1.24 | 1.23 | 1.41 |
| Guatemala | 80-84 years | 1.25 | 1.16 | 1.33 |
| Libya | 80-84 years | 1.25 | 1.26 | 1.19 |
| Mozambique | 80-84 years | 1.27 | 1.28 | 1.26 |
| Democratic Republic of the Congo | 80-84 years | 1.27 | 1.29 | 1.03 |
| Singapore | 80-84 years | 1.27 | 1.26 | 1.47 |
| Ecuador | 80-84 years | 1.27 | 1.27 | 1.34 |
| Poland | 80-84 years | 1.28 | 1.32 | 1.03 |
| Equatorial Guinea | 80-84 years | 1.29 | 1.32 | 1.03 |
| Bulgaria | 80-84 years | 1.29 | 1.32 | 1.05 |
| Haiti | 80-84 years | 1.29 | 1.30 | 1.21 |
| Peru | 80-84 years | 1.29 | 1.30 | 1.28 |
| United States of America | 80-84 years | 1.29 | 1.42 | 1.06 |
| Gabon | 80-84 years | 1.31 | 1.33 | 1.08 |
| Madagascar | 80-84 years | 1.31 | 1.32 | 1.21 |
| Burundi | 80-84 years | 1.32 | 1.32 | 1.23 |
| Australia | 80-84 years | 1.32 | 1.33 | 1.13 |
| Guyana | 80-84 years | 1.32 | 1.33 | 1.31 |
| Canada | 80-84 years | 1.33 | 1.42 | 1.07 |
| Nicaragua | 80-84 years | 1.33 | 1.32 | 1.39 |
| Uganda | 80-84 years | 1.33 | 1.34 | 1.30 |
| Northern Mariana Islands | 80-84 years | 1.33 | 1.37 | 1.28 |
| Turkiye | 80-84 years | 1.33 | 1.35 | 1.26 |
| Uruguay | 80-84 years | 1.34 | 1.36 | 1.28 |
| Ethiopia | 80-84 years | 1.35 | 1.35 | 1.28 |
| Iran (Islamic Republic of) | 80-84 years | 1.35 | 1.36 | 1.29 |
| Algeria | 80-84 years | 1.36 | 1.39 | 1.17 |
| Malawi | 80-84 years | 1.36 | 1.37 | 1.26 |
| Somalia | 80-84 years | 1.37 | 1.38 | 1.22 |
| Pakistan | 80-84 years | 1.38 | 1.38 | 1.19 |
| Oman | 80-84 years | 1.38 | 1.39 | 1.33 |
| Montenegro | 80-84 years | 1.39 | 1.44 | 1.08 |
| Jordan | 80-84 years | 1.40 | 1.47 | 1.18 |
| Belize | 80-84 years | 1.40 | 1.46 | 1.33 |
| Argentina | 80-84 years | 1.40 | 1.41 | 1.37 |
| Cuba | 80-84 years | 1.40 | 1.42 | 1.34 |
| United States Virgin Islands | 80-84 years | 1.41 | 1.46 | 1.37 |
| Czechia | 80-84 years | 1.41 | 1.44 | 1.22 |
| Israel | 80-84 years | 1.42 | 1.42 | 1.43 |
| Ireland | 80-84 years | 1.42 | 1.43 | 1.35 |
| Portugal | 80-84 years | 1.43 | 1.48 | 1.08 |
| Suriname | 80-84 years | 1.43 | 1.49 | 1.33 |
| Puerto Rico | 80-84 years | 1.43 | 1.53 | 1.31 |
| San Marino | 80-84 years | 1.45 | 1.47 | 1.41 |
| Norway | 80-84 years | 1.47 | 1.49 | 1.18 |
| Netherlands | 80-84 years | 1.48 | 1.50 | 1.26 |
| Slovenia | 80-84 years | 1.49 | 1.51 | 1.30 |
| Palestine | 80-84 years | 1.49 | 1.51 | 1.25 |
| North Macedonia | 80-84 years | 1.49 | 1.53 | 1.07 |
| Saint Kitts and Nevis | 80-84 years | 1.50 | 1.48 | 1.54 |
| Congo | 80-84 years | 1.50 | 1.53 | 1.01 |
| Iceland | 80-84 years | 1.51 | 1.58 | 0.80 |
| Slovakia | 80-84 years | 1.51 | 1.70 | 0.97 |
| Belgium | 80-84 years | 1.51 | 1.52 | 1.48 |
| Republic of Korea | 80-84 years | 1.52 | 1.51 | 1.81 |
| Hungary | 80-84 years | 1.54 | 1.58 | 1.33 |
| Syrian Arab Republic | 80-84 years | 1.54 | 1.56 | 1.22 |
| Albania | 80-84 years | 1.54 | 1.56 | 1.37 |
| Thailand | 80-84 years | 1.54 | 1.59 | 1.47 |
| Mauritius | 80-84 years | 1.54 | 1.60 | 1.44 |
| United Republic of Tanzania | 80-84 years | 1.55 | 1.56 | 1.26 |
| Malaysia | 80-84 years | 1.55 | 1.60 | 1.40 |
| Rwanda | 80-84 years | 1.55 | 1.57 | 1.24 |
| Comoros | 80-84 years | 1.56 | 1.57 | 1.22 |
| Croatia | 80-84 years | 1.56 | 1.56 | 1.82 |
| Malta | 80-84 years | 1.56 | 1.59 | 1.25 |
| Trinidad and Tobago | 80-84 years | 1.58 | 1.70 | 1.33 |
| Djibouti | 80-84 years | 1.58 | 1.60 | 1.27 |
| Italy | 80-84 years | 1.58 | 1.62 | 1.27 |
| Zambia | 80-84 years | 1.59 | 1.61 | 1.27 |
| United Kingdom | 80-84 years | 1.59 | 1.61 | 1.42 |
| Maldives | 80-84 years | 1.60 | 1.72 | 1.24 |
| Eritrea | 80-84 years | 1.62 | 1.64 | 1.23 |
| Bahamas | 80-84 years | 1.64 | 1.86 | 1.37 |
| Greece | 80-84 years | 1.64 | 1.72 | 1.02 |
| Sao Tome and Principe | 80-84 years | 1.65 | 1.68 | 1.17 |
| Cyprus | 80-84 years | 1.70 | 1.73 | 1.46 |
| Republic of Moldova | 80-84 years | 1.72 | 1.92 | 1.45 |
| Bahrain | 80-84 years | 1.72 | 1.75 | 1.58 |
| Serbia | 80-84 years | 1.76 | 1.87 | 0.99 |
| Spain | 80-84 years | 1.77 | 1.77 | 1.71 |
| El Salvador | 80-84 years | 1.77 | 2.02 | 1.46 |
| Bolivia (Plurinational State of) | 80-84 years | 1.79 | 1.84 | 1.21 |
| Mexico | 80-84 years | 1.79 | 1.91 | 1.41 |
| Kenya | 80-84 years | 1.80 | 1.84 | 1.27 |
| Dominica | 80-84 years | 1.82 | 1.97 | 1.33 |
| Barbados | 80-84 years | 1.84 | 2.21 | 1.32 |
| Brunei Darussalam | 80-84 years | 1.84 | 1.85 | 1.57 |
| Saint Lucia | 80-84 years | 1.86 | 2.03 | 1.37 |
| Dominican Republic | 80-84 years | 1.88 | 2.18 | 1.36 |
| Ukraine | 80-84 years | 1.99 | 2.44 | 1.59 |
| Honduras | 80-84 years | 2.03 | 2.30 | 1.24 |
| Saudi Arabia | 80-84 years | 2.06 | 2.32 | 1.32 |
| Belarus | 80-84 years | 2.11 | 2.56 | 1.47 |
| Bosnia and Herzegovina | 80-84 years | 2.16 | 2.37 | 1.13 |
| Armenia | 80-84 years | 2.24 | 2.34 | 1.41 |
| Latvia | 80-84 years | 2.30 | 3.02 | 1.46 |
| Lithuania | 80-84 years | 2.32 | 2.97 | 1.48 |
| Russian Federation | 80-84 years | 2.36 | 2.69 | 1.54 |
| Egypt | 80-84 years | 2.38 | 2.42 | 1.19 |
| Greenland | 80-84 years | 2.41 | 3.33 | 1.24 |
| Antigua and Barbuda | 80-84 years | 2.53 | 3.22 | 1.35 |
| Estonia | 80-84 years | 2.61 | 3.72 | 1.54 |
| Andorra | 80-84 years | 2.82 | 3.03 | 2.03 |
| United Arab Emirates | 80-84 years | 4.55 | 4.80 | 1.21 |
| Papua New Guinea | 85-89 years | 0.28 | 0.27 | 1.21 |
| Cook Islands | 85-89 years | 0.32 | 0.27 | 0.78 |
| Fiji | 85-89 years | 0.34 | 0.34 | 1.23 |
| Guam | 85-89 years | 0.34 | 0.23 | 1.33 |
| Solomon Islands | 85-89 years | 0.40 | 0.39 | 1.18 |
| Vanuatu | 85-89 years | 0.41 | 0.41 | 1.26 |
| Burkina Faso | 85-89 years | 0.44 | 0.43 | 1.19 |
| Benin | 85-89 years | 0.46 | 0.45 | 1.20 |
| Cabo Verde | 85-89 years | 0.49 | 0.47 | 1.38 |
| Chad | 85-89 years | 0.50 | 0.49 | 1.16 |
| Nauru | 85-89 years | 0.52 | 0.50 | 1.10 |
| Niger | 85-89 years | 0.53 | 0.52 | 1.15 |
| Niue | 85-89 years | 0.55 | 0.52 | 1.15 |
| Sierra Leone | 85-89 years | 0.55 | 0.54 | 1.16 |
| Togo | 85-89 years | 0.57 | 0.56 | 1.20 |
| C么te d'Ivoire | 85-89 years | 0.57 | 0.56 | 1.23 |
| Guinea | 85-89 years | 0.57 | 0.57 | 1.16 |
| Kiribati | 85-89 years | 0.57 | 0.57 | 1.23 |
| Marshall Islands | 85-89 years | 0.58 | 0.58 | 1.20 |
| Guinea-Bissau | 85-89 years | 0.59 | 0.58 | 1.17 |
| Botswana | 85-89 years | 0.59 | 0.56 | 1.28 |
| Senegal | 85-89 years | 0.59 | 0.58 | 1.18 |
| Gambia | 85-89 years | 0.61 | 0.61 | 1.20 |
| Cameroon | 85-89 years | 0.62 | 0.61 | 1.18 |
| Samoa | 85-89 years | 0.62 | 0.61 | 1.23 |
| Namibia | 85-89 years | 0.63 | 0.61 | 1.26 |
| Ghana | 85-89 years | 0.65 | 0.63 | 1.20 |
| Tonga | 85-89 years | 0.65 | 0.63 | 1.27 |
| Tuvalu | 85-89 years | 0.66 | 0.64 | 1.38 |
| Liberia | 85-89 years | 0.66 | 0.65 | 1.13 |
| Micronesia (Federated States of) | 85-89 years | 0.67 | 0.67 | 1.22 |
| Tokelau | 85-89 years | 0.68 | 0.65 | 1.37 |
| China | 85-89 years | 0.70 | 0.69 | 1.50 |
| Palau | 85-89 years | 0.70 | 0.68 | 1.24 |
| Nigeria | 85-89 years | 0.70 | 0.69 | 1.21 |
| Mali | 85-89 years | 0.72 | 0.71 | 1.13 |
| Mauritania | 85-89 years | 0.74 | 0.73 | 1.08 |
| Sri Lanka | 85-89 years | 0.75 | 1.03 | 0.31 |
| Seychelles | 85-89 years | 0.77 | 0.67 | 1.47 |
| Tajikistan | 85-89 years | 0.78 | 0.76 | 1.25 |
| Turkmenistan | 85-89 years | 0.79 | 0.77 | 1.30 |
| Zimbabwe | 85-89 years | 0.80 | 0.80 | 1.22 |
| Finland | 85-89 years | 0.80 | 0.79 | 1.45 |
| Kyrgyzstan | 85-89 years | 0.83 | 0.82 | 1.37 |
| Uzbekistan | 85-89 years | 0.83 | 0.83 | 1.28 |
| Afghanistan | 85-89 years | 0.86 | 0.85 | 1.14 |
| Grenada | 85-89 years | 0.87 | 0.87 | 1.30 |
| Tunisia | 85-89 years | 0.88 | 0.87 | 1.15 |
| Myanmar | 85-89 years | 0.91 | 0.88 | 1.54 |
| Viet Nam | 85-89 years | 0.91 | 1.28 | 0.40 |
| South Africa | 85-89 years | 0.92 | 0.90 | 1.29 |
| American Samoa | 85-89 years | 0.92 | 0.92 | 1.26 |
| Qatar | 85-89 years | 0.93 | 0.82 | 2.08 |
| Bermuda | 85-89 years | 0.94 | 0.83 | 1.24 |
| Bangladesh | 85-89 years | 0.94 | 0.94 | 1.54 |
| Azerbaijan | 85-89 years | 0.95 | 0.93 | 1.34 |
| Angola | 85-89 years | 0.95 | 0.95 | 1.04 |
| Ecuador | 85-89 years | 0.96 | 0.93 | 1.34 |
| Central African Republic | 85-89 years | 0.96 | 0.96 | 1.03 |
| Brunei Darussalam | 85-89 years | 0.96 | 0.96 | 1.47 |
| Kazakhstan | 85-89 years | 0.97 | 0.96 | 1.11 |
| Yemen | 85-89 years | 0.97 | 0.97 | 1.19 |
| India | 85-89 years | 0.97 | 0.97 | 1.40 |
| Georgia | 85-89 years | 0.99 | 0.98 | 1.43 |
| Lesotho | 85-89 years | 0.99 | 0.99 | 1.24 |
| Oman | 85-89 years | 1.04 | 1.02 | 1.23 |
| Democratic People's Republic of Korea | 85-89 years | 1.05 | 1.06 | 1.17 |
| Cambodia | 85-89 years | 1.05 | 1.05 | 1.19 |
| Timor-Leste | 85-89 years | 1.05 | 1.05 | 1.26 |
| Saint Vincent and the Grenadines | 85-89 years | 1.06 | 1.04 | 1.31 |
| Denmark | 85-89 years | 1.06 | 1.05 | 1.58 |
| Sudan | 85-89 years | 1.06 | 1.06 | 1.24 |
| Brazil | 85-89 years | 1.06 | 1.02 | 1.51 |
| Bhutan | 85-89 years | 1.06 | 1.06 | 1.25 |
| Eswatini | 85-89 years | 1.07 | 1.07 | 1.23 |
| Kuwait | 85-89 years | 1.08 | 1.09 | 1.08 |
| Haiti | 85-89 years | 1.08 | 1.08 | 1.21 |
| Switzerland | 85-89 years | 1.09 | 1.09 | 1.20 |
| Democratic Republic of the Congo | 85-89 years | 1.09 | 1.10 | 1.03 |
| Paraguay | 85-89 years | 1.10 | 1.07 | 1.36 |
| France | 85-89 years | 1.10 | 1.11 | 1.20 |
| Taiwan (Province of China) | 85-89 years | 1.10 | 1.10 | 1.13 |
| Equatorial Guinea | 85-89 years | 1.11 | 1.12 | 1.03 |
| Jamaica | 85-89 years | 1.12 | 1.10 | 1.25 |
| Lao People's Democratic Republic | 85-89 years | 1.12 | 1.11 | 1.27 |
| Gabon | 85-89 years | 1.12 | 1.13 | 1.08 |
| Lebanon | 85-89 years | 1.13 | 1.13 | 1.13 |
| Costa Rica | 85-89 years | 1.15 | 1.10 | 1.40 |
| Monaco | 85-89 years | 1.16 | 1.17 | 1.18 |
| Romania | 85-89 years | 1.16 | 1.17 | 1.15 |
| Iraq | 85-89 years | 1.17 | 1.17 | 1.23 |
| Guatemala | 85-89 years | 1.17 | 1.09 | 1.33 |
| Venezuela (Bolivarian Republic of) | 85-89 years | 1.17 | 1.14 | 1.29 |
| United States Virgin Islands | 85-89 years | 1.17 | 1.05 | 1.36 |
| Nicaragua | 85-89 years | 1.18 | 1.13 | 1.39 |
| Northern Mariana Islands | 85-89 years | 1.19 | 1.17 | 1.27 |
| Panama | 85-89 years | 1.20 | 1.16 | 1.36 |
| Mongolia | 85-89 years | 1.20 | 1.21 | 1.29 |
| Nepal | 85-89 years | 1.20 | 1.20 | 1.09 |
| Japan | 85-89 years | 1.20 | 1.20 | 1.43 |
| Colombia | 85-89 years | 1.21 | 1.14 | 1.33 |
| Ethiopia | 85-89 years | 1.21 | 1.21 | 1.28 |
| Guyana | 85-89 years | 1.22 | 1.19 | 1.31 |
| Portugal | 85-89 years | 1.22 | 1.25 | 1.02 |
| New Zealand | 85-89 years | 1.23 | 1.25 | 0.90 |
| South Sudan | 85-89 years | 1.25 | 1.25 | 1.25 |
| Peru | 85-89 years | 1.25 | 1.25 | 1.27 |
| United States of America | 85-89 years | 1.25 | 1.36 | 1.02 |
| Montenegro | 85-89 years | 1.26 | 1.27 | 1.03 |
| Morocco | 85-89 years | 1.27 | 1.28 | 1.17 |
| Uganda | 85-89 years | 1.27 | 1.28 | 1.30 |
| Australia | 85-89 years | 1.28 | 1.29 | 1.11 |
| Singapore | 85-89 years | 1.29 | 1.30 | 1.25 |
| Trinidad and Tobago | 85-89 years | 1.29 | 1.29 | 1.32 |
| Belize | 85-89 years | 1.30 | 1.30 | 1.33 |
| Cuba | 85-89 years | 1.31 | 1.31 | 1.34 |
| Malawi | 85-89 years | 1.31 | 1.32 | 1.27 |
| Burundi | 85-89 years | 1.31 | 1.32 | 1.24 |
| Chile | 85-89 years | 1.32 | 1.32 | 1.33 |
| Congo | 85-89 years | 1.32 | 1.33 | 1.01 |
| Germany | 85-89 years | 1.33 | 1.34 | 1.37 |
| Indonesia | 85-89 years | 1.33 | 1.51 | 0.31 |
| Madagascar | 85-89 years | 1.34 | 1.35 | 1.22 |
| Libya | 85-89 years | 1.34 | 1.36 | 1.20 |
| Bulgaria | 85-89 years | 1.35 | 1.38 | 1.01 |
| Luxembourg | 85-89 years | 1.35 | 1.37 | 1.24 |
| Canada | 85-89 years | 1.38 | 1.47 | 1.06 |
| Pakistan | 85-89 years | 1.39 | 1.39 | 1.19 |
| Austria | 85-89 years | 1.39 | 1.40 | 1.37 |
| Serbia | 85-89 years | 1.41 | 1.46 | 0.92 |
| Bahamas | 85-89 years | 1.41 | 1.45 | 1.36 |
| Palestine | 85-89 years | 1.43 | 1.44 | 1.24 |
| Ireland | 85-89 years | 1.43 | 1.44 | 1.29 |
| Sweden | 85-89 years | 1.45 | 1.46 | 1.44 |
| Iceland | 85-89 years | 1.46 | 1.51 | 0.72 |
| Poland | 85-89 years | 1.46 | 1.52 | 0.96 |
| Sao Tome and Principe | 85-89 years | 1.46 | 1.47 | 1.17 |
| Cyprus | 85-89 years | 1.46 | 1.47 | 1.49 |
| Somalia | 85-89 years | 1.46 | 1.47 | 1.22 |
| Israel | 85-89 years | 1.47 | 1.48 | 1.31 |
| Mozambique | 85-89 years | 1.47 | 1.48 | 1.26 |
| Italy | 85-89 years | 1.47 | 1.49 | 1.21 |
| Suriname | 85-89 years | 1.49 | 1.55 | 1.33 |
| Czechia | 85-89 years | 1.49 | 1.51 | 1.14 |
| Comoros | 85-89 years | 1.50 | 1.51 | 1.23 |
| Albania | 85-89 years | 1.51 | 1.53 | 1.37 |
| Norway | 85-89 years | 1.52 | 1.54 | 1.17 |
| Rwanda | 85-89 years | 1.52 | 1.53 | 1.25 |
| Saint Kitts and Nevis | 85-89 years | 1.53 | 1.56 | 1.48 |
| Slovakia | 85-89 years | 1.53 | 1.72 | 0.91 |
| Bolivia (Plurinational State of) | 85-89 years | 1.54 | 1.56 | 1.22 |
| Iran (Islamic Republic of) | 85-89 years | 1.54 | 1.56 | 1.28 |
| Uruguay | 85-89 years | 1.56 | 1.63 | 1.27 |
| Djibouti | 85-89 years | 1.56 | 1.58 | 1.27 |
| Ukraine | 85-89 years | 1.57 | 1.61 | 1.52 |
| Maldives | 85-89 years | 1.58 | 1.64 | 1.25 |
| Greece | 85-89 years | 1.58 | 1.63 | 0.96 |
| Dominica | 85-89 years | 1.58 | 1.62 | 1.33 |
| Netherlands | 85-89 years | 1.59 | 1.62 | 1.27 |
| Republic of Moldova | 85-89 years | 1.59 | 1.79 | 1.40 |
| United Republic of Tanzania | 85-89 years | 1.59 | 1.61 | 1.26 |
| United Kingdom | 85-89 years | 1.59 | 1.61 | 1.37 |
| Puerto Rico | 85-89 years | 1.63 | 1.91 | 1.27 |
| Zambia | 85-89 years | 1.63 | 1.64 | 1.27 |
| Turkiye | 85-89 years | 1.63 | 1.69 | 1.19 |
| Saint Lucia | 85-89 years | 1.64 | 1.69 | 1.37 |
| Eritrea | 85-89 years | 1.64 | 1.66 | 1.24 |
| Argentina | 85-89 years | 1.66 | 1.68 | 1.37 |
| Spain | 85-89 years | 1.67 | 1.68 | 1.63 |
| Philippines | 85-89 years | 1.67 | 1.72 | 1.43 |
| Hungary | 85-89 years | 1.70 | 1.75 | 1.21 |
| El Salvador | 85-89 years | 1.71 | 1.85 | 1.45 |
| San Marino | 85-89 years | 1.71 | 1.77 | 1.33 |
| Republic of Korea | 85-89 years | 1.72 | 1.74 | 1.57 |
| Kenya | 85-89 years | 1.76 | 1.78 | 1.27 |
| Malta | 85-89 years | 1.76 | 1.80 | 1.17 |
| Belarus | 85-89 years | 1.78 | 2.14 | 1.43 |
| Belgium | 85-89 years | 1.80 | 1.83 | 1.52 |
| Slovenia | 85-89 years | 1.81 | 1.84 | 1.23 |
| Thailand | 85-89 years | 1.83 | 1.99 | 1.47 |
| Mauritius | 85-89 years | 1.86 | 2.01 | 1.44 |
| Lithuania | 85-89 years | 1.89 | 2.28 | 1.43 |
| Mexico | 85-89 years | 1.91 | 2.07 | 1.36 |
| Latvia | 85-89 years | 1.94 | 2.50 | 1.41 |
| Dominican Republic | 85-89 years | 1.95 | 2.13 | 1.35 |
| Honduras | 85-89 years | 1.97 | 2.14 | 1.24 |
| Barbados | 85-89 years | 1.98 | 2.25 | 1.32 |
| Estonia | 85-89 years | 1.99 | 2.58 | 1.47 |
| Jordan | 85-89 years | 2.01 | 2.21 | 1.13 |
| Bahrain | 85-89 years | 2.06 | 2.17 | 1.41 |
| Croatia | 85-89 years | 2.08 | 2.11 | 1.75 |
| Syrian Arab Republic | 85-89 years | 2.08 | 2.09 | 1.22 |
| North Macedonia | 85-89 years | 2.10 | 2.20 | 1.00 |
| Antigua and Barbuda | 85-89 years | 2.11 | 2.40 | 1.35 |
| Greenland | 85-89 years | 2.25 | 2.90 | 1.19 |
| Malaysia | 85-89 years | 2.27 | 2.48 | 1.39 |
| Bosnia and Herzegovina | 85-89 years | 2.28 | 2.54 | 1.05 |
| Saudi Arabia | 85-89 years | 2.30 | 2.66 | 1.23 |
| Russian Federation | 85-89 years | 2.65 | 3.50 | 1.45 |
| Algeria | 85-89 years | 2.69 | 2.73 | 1.17 |
| Armenia | 85-89 years | 2.69 | 2.78 | 1.41 |
| Andorra | 85-89 years | 2.78 | 2.96 | 1.77 |
| Egypt | 85-89 years | 4.11 | 4.13 | 1.20 |
| United Arab Emirates | 85-89 years | 5.33 | 5.41 | 1.21 |
| Sri Lanka | 90-94 years | 0.63 | 0.84 | 0.23 |
| Benin | 90-94 years | 0.66 | 0.65 | 1.20 |
| Oman | 90-94 years | 0.67 | 0.63 | 1.09 |
| Burkina Faso | 90-94 years | 0.68 | 0.68 | 1.19 |
| China | 90-94 years | 0.68 | 0.68 | 1.33 |
| Seychelles | 90-94 years | 0.69 | 0.62 | 1.46 |
| Cabo Verde | 90-94 years | 0.70 | 0.69 | 1.37 |
| Turkmenistan | 90-94 years | 0.72 | 0.71 | 1.29 |
| Tajikistan | 90-94 years | 0.74 | 0.72 | 1.26 |
| Myanmar | 90-94 years | 0.74 | 0.72 | 1.54 |
| Chad | 90-94 years | 0.75 | 0.74 | 1.16 |
| Kyrgyzstan | 90-94 years | 0.77 | 0.75 | 1.37 |
| Uzbekistan | 90-94 years | 0.81 | 0.80 | 1.28 |
| Brunei Darussalam | 90-94 years | 0.81 | 0.82 | 1.02 |
| Saint Vincent and the Grenadines | 90-94 years | 0.81 | 0.79 | 1.31 |
| Cambodia | 90-94 years | 0.81 | 0.81 | 1.19 |
| Sierra Leone | 90-94 years | 0.82 | 0.81 | 1.16 |
| Niger | 90-94 years | 0.82 | 0.82 | 1.15 |
| Timor-Leste | 90-94 years | 0.82 | 0.81 | 1.26 |
| Qatar | 90-94 years | 0.83 | 0.78 | 1.59 |
| Viet Nam | 90-94 years | 0.83 | 1.11 | 0.33 |
| Guinea | 90-94 years | 0.83 | 0.83 | 1.16 |
| Togo | 90-94 years | 0.83 | 0.83 | 1.19 |
| C么te d'Ivoire | 90-94 years | 0.84 | 0.84 | 1.23 |
| Finland | 90-94 years | 0.85 | 0.84 | 1.23 |
| Tunisia | 90-94 years | 0.85 | 0.83 | 1.11 |
| Cook Islands | 90-94 years | 0.85 | 0.86 | 0.59 |
| Papua New Guinea | 90-94 years | 0.86 | 0.86 | 1.21 |
| Senegal | 90-94 years | 0.87 | 0.86 | 1.18 |
| Afghanistan | 90-94 years | 0.87 | 0.87 | 1.14 |
| Yemen | 90-94 years | 0.87 | 0.87 | 1.19 |
| Georgia | 90-94 years | 0.88 | 0.88 | 1.43 |
| Gambia | 90-94 years | 0.88 | 0.88 | 1.20 |
| Guam | 90-94 years | 0.89 | 0.85 | 1.32 |
| Lao People's Democratic Republic | 90-94 years | 0.89 | 0.88 | 1.27 |
| Cameroon | 90-94 years | 0.90 | 0.90 | 1.18 |
| Guinea-Bissau | 90-94 years | 0.91 | 0.91 | 1.17 |
| Liberia | 90-94 years | 0.92 | 0.92 | 1.14 |
| Guatemala | 90-94 years | 0.92 | 0.82 | 1.33 |
| India | 90-94 years | 0.92 | 0.92 | 1.39 |
| Grenada | 90-94 years | 0.93 | 0.92 | 1.30 |
| Fiji | 90-94 years | 0.94 | 0.95 | 1.23 |
| Sudan | 90-94 years | 0.95 | 0.95 | 1.24 |
| Bermuda | 90-94 years | 0.95 | 0.84 | 1.23 |
| Azerbaijan | 90-94 years | 0.95 | 0.93 | 1.34 |
| Switzerland | 90-94 years | 0.95 | 0.95 | 1.17 |
| Bangladesh | 90-94 years | 0.97 | 0.97 | 1.54 |
| Angola | 90-94 years | 0.99 | 0.99 | 1.04 |
| Democratic People's Republic of Korea | 90-94 years | 1.01 | 1.01 | 1.17 |
| Nicaragua | 90-94 years | 1.01 | 0.95 | 1.38 |
| Bhutan | 90-94 years | 1.01 | 1.01 | 1.25 |
| Mauritania | 90-94 years | 1.01 | 1.01 | 1.08 |
| Nigeria | 90-94 years | 1.02 | 1.02 | 1.22 |
| Ecuador | 90-94 years | 1.03 | 1.02 | 1.34 |
| Haiti | 90-94 years | 1.04 | 1.03 | 1.21 |
| Monaco | 90-94 years | 1.05 | 1.04 | 1.15 |
| Colombia | 90-94 years | 1.05 | 0.94 | 1.27 |
| Mali | 90-94 years | 1.05 | 1.05 | 1.13 |
| Ghana | 90-94 years | 1.06 | 1.05 | 1.20 |
| Paraguay | 90-94 years | 1.06 | 1.04 | 1.36 |
| Costa Rica | 90-94 years | 1.06 | 1.02 | 1.40 |
| Denmark | 90-94 years | 1.07 | 1.06 | 1.66 |
| Democratic Republic of the Congo | 90-94 years | 1.07 | 1.07 | 1.04 |
| United States Virgin Islands | 90-94 years | 1.08 | 0.96 | 1.34 |
| Central African Republic | 90-94 years | 1.09 | 1.09 | 1.04 |
| Taiwan (Province of China) | 90-94 years | 1.09 | 1.11 | 1.03 |
| Lebanon | 90-94 years | 1.09 | 1.09 | 1.10 |
| New Zealand | 90-94 years | 1.09 | 1.10 | 0.80 |
| Panama | 90-94 years | 1.10 | 1.06 | 1.36 |
| Indonesia | 90-94 years | 1.11 | 1.22 | 0.21 |
| Botswana | 90-94 years | 1.11 | 1.11 | 1.28 |
| Palestine | 90-94 years | 1.12 | 1.12 | 1.24 |
| Equatorial Guinea | 90-94 years | 1.13 | 1.14 | 1.03 |
| Morocco | 90-94 years | 1.14 | 1.14 | 1.17 |
| Namibia | 90-94 years | 1.14 | 1.14 | 1.26 |
| Kuwait | 90-94 years | 1.15 | 1.17 | 1.05 |
| Gabon | 90-94 years | 1.15 | 1.15 | 1.08 |
| Jamaica | 90-94 years | 1.16 | 1.15 | 1.26 |
| Madagascar | 90-94 years | 1.19 | 1.19 | 1.22 |
| Iraq | 90-94 years | 1.20 | 1.20 | 1.23 |
| Trinidad and Tobago | 90-94 years | 1.20 | 1.18 | 1.32 |
| Ethiopia | 90-94 years | 1.21 | 1.21 | 1.29 |
| Portugal | 90-94 years | 1.21 | 1.22 | 0.96 |
| Brazil | 90-94 years | 1.21 | 1.19 | 1.51 |
| Nepal | 90-94 years | 1.22 | 1.22 | 1.09 |
| South Sudan | 90-94 years | 1.22 | 1.22 | 1.26 |
| Peru | 90-94 years | 1.22 | 1.22 | 1.27 |
| South Africa | 90-94 years | 1.23 | 1.23 | 1.29 |
| Kazakhstan | 90-94 years | 1.24 | 1.26 | 1.04 |
| Solomon Islands | 90-94 years | 1.26 | 1.26 | 1.18 |
| Congo | 90-94 years | 1.28 | 1.29 | 1.02 |
| Burundi | 90-94 years | 1.28 | 1.29 | 1.24 |
| Venezuela (Bolivarian Republic of) | 90-94 years | 1.29 | 1.31 | 1.24 |
| United States of America | 90-94 years | 1.29 | 1.41 | 0.95 |
| Vanuatu | 90-94 years | 1.29 | 1.29 | 1.26 |
| France | 90-94 years | 1.30 | 1.31 | 1.17 |
| Serbia | 90-94 years | 1.31 | 1.35 | 0.82 |
| Cuba | 90-94 years | 1.32 | 1.33 | 1.34 |
| Belize | 90-94 years | 1.32 | 1.33 | 1.33 |
| Luxembourg | 90-94 years | 1.32 | 1.33 | 1.22 |
| Pakistan | 90-94 years | 1.32 | 1.33 | 1.19 |
| Singapore | 90-94 years | 1.33 | 1.35 | 0.91 |
| Uganda | 90-94 years | 1.34 | 1.34 | 1.29 |
| Iceland | 90-94 years | 1.34 | 1.37 | 0.62 |
| Romania | 90-94 years | 1.35 | 1.38 | 1.07 |
| Mongolia | 90-94 years | 1.37 | 1.38 | 1.29 |
| Austria | 90-94 years | 1.37 | 1.37 | 1.32 |
| Bahamas | 90-94 years | 1.37 | 1.38 | 1.35 |
| Libya | 90-94 years | 1.39 | 1.39 | 1.20 |
| Maldives | 90-94 years | 1.42 | 1.44 | 1.25 |
| Germany | 90-94 years | 1.43 | 1.43 | 1.27 |
| Bulgaria | 90-94 years | 1.43 | 1.46 | 0.96 |
| Republic of Moldova | 90-94 years | 1.44 | 1.58 | 1.32 |
| Japan | 90-94 years | 1.44 | 1.45 | 1.06 |
| Comoros | 90-94 years | 1.47 | 1.47 | 1.24 |
| Guyana | 90-94 years | 1.48 | 1.50 | 1.31 |
| Canada | 90-94 years | 1.49 | 1.60 | 1.05 |
| Djibouti | 90-94 years | 1.50 | 1.51 | 1.27 |
| Australia | 90-94 years | 1.50 | 1.52 | 1.06 |
| Rwanda | 90-94 years | 1.50 | 1.51 | 1.25 |
| Montenegro | 90-94 years | 1.50 | 1.54 | 0.94 |
| Chile | 90-94 years | 1.52 | 1.55 | 1.27 |
| Italy | 90-94 years | 1.52 | 1.54 | 1.10 |
| Slovakia | 90-94 years | 1.53 | 1.74 | 0.85 |
| Saint Kitts and Nevis | 90-94 years | 1.54 | 1.59 | 1.41 |
| Zimbabwe | 90-94 years | 1.55 | 1.56 | 1.22 |
| Mexico | 90-94 years | 1.55 | 1.62 | 1.30 |
| Dominica | 90-94 years | 1.56 | 1.58 | 1.33 |
| Ireland | 90-94 years | 1.56 | 1.57 | 1.23 |
| Albania | 90-94 years | 1.56 | 1.57 | 1.37 |
| Eritrea | 90-94 years | 1.56 | 1.57 | 1.24 |
| Israel | 90-94 years | 1.56 | 1.58 | 1.15 |
| United Republic of Tanzania | 90-94 years | 1.57 | 1.58 | 1.26 |
| Ukraine | 90-94 years | 1.58 | 1.89 | 1.42 |
| Malta | 90-94 years | 1.59 | 1.61 | 1.09 |
| Sweden | 90-94 years | 1.60 | 1.61 | 1.37 |
| Greece | 90-94 years | 1.61 | 1.65 | 0.79 |
| Niue | 90-94 years | 1.62 | 1.63 | 0.94 |
| United Kingdom | 90-94 years | 1.62 | 1.64 | 1.27 |
| Spain | 90-94 years | 1.64 | 1.65 | 1.52 |
| Kenya | 90-94 years | 1.65 | 1.66 | 1.27 |
| El Salvador | 90-94 years | 1.65 | 1.72 | 1.44 |
| Nauru | 90-94 years | 1.66 | 1.68 | 0.96 |
| Iran (Islamic Republic of) | 90-94 years | 1.68 | 1.70 | 1.27 |
| Malawi | 90-94 years | 1.69 | 1.71 | 1.27 |
| Bolivia (Plurinational State of) | 90-94 years | 1.70 | 1.72 | 1.22 |
| Puerto Rico | 90-94 years | 1.70 | 2.16 | 1.25 |
| Netherlands | 90-94 years | 1.70 | 1.72 | 1.20 |
| Poland | 90-94 years | 1.70 | 1.80 | 0.82 |
| Belarus | 90-94 years | 1.72 | 2.04 | 1.37 |
| Thailand | 90-94 years | 1.73 | 1.82 | 1.46 |
| Norway | 90-94 years | 1.74 | 1.76 | 1.13 |
| Kiribati | 90-94 years | 1.75 | 1.76 | 1.23 |
| Saint Lucia | 90-94 years | 1.76 | 1.79 | 1.37 |
| Hungary | 90-94 years | 1.77 | 1.87 | 0.99 |
| Philippines | 90-94 years | 1.79 | 1.83 | 1.44 |
| Latvia | 90-94 years | 1.79 | 2.27 | 1.35 |
| Marshall Islands | 90-94 years | 1.80 | 1.81 | 1.21 |
| Republic of Korea | 90-94 years | 1.81 | 1.83 | 1.05 |
| Mozambique | 90-94 years | 1.81 | 1.83 | 1.25 |
| Uruguay | 90-94 years | 1.83 | 1.94 | 1.23 |
| Lithuania | 90-94 years | 1.86 | 2.25 | 1.36 |
| Belgium | 90-94 years | 1.89 | 1.91 | 1.57 |
| Dominican Republic | 90-94 years | 1.90 | 2.02 | 1.35 |
| Suriname | 90-94 years | 1.90 | 2.08 | 1.33 |
| Honduras | 90-94 years | 1.91 | 2.01 | 1.24 |
| Somalia | 90-94 years | 1.92 | 1.94 | 1.22 |
| Barbados | 90-94 years | 1.93 | 2.09 | 1.32 |
| Samoa | 90-94 years | 1.93 | 1.94 | 1.23 |
| Turkiye | 90-94 years | 1.95 | 2.05 | 1.13 |
| Argentina | 90-94 years | 1.96 | 1.97 | 1.37 |
| Mauritius | 90-94 years | 1.96 | 2.09 | 1.43 |
| Estonia | 90-94 years | 1.98 | 2.59 | 1.39 |
| Slovenia | 90-94 years | 1.99 | 2.02 | 1.10 |
| Micronesia (Federated States of) | 90-94 years | 2.00 | 2.00 | 1.22 |
| Tuvalu | 90-94 years | 2.01 | 2.02 | 1.20 |
| Bahrain | 90-94 years | 2.01 | 2.15 | 1.21 |
| Tokelau | 90-94 years | 2.04 | 2.06 | 1.13 |
| Sao Tome and Principe | 90-94 years | 2.06 | 2.08 | 1.17 |
| Saudi Arabia | 90-94 years | 2.10 | 2.33 | 1.14 |
| Czechia | 90-94 years | 2.12 | 2.19 | 0.99 |
| Cyprus | 90-94 years | 2.13 | 2.15 | 1.50 |
| Malaysia | 90-94 years | 2.14 | 2.25 | 1.39 |
| Tonga | 90-94 years | 2.16 | 2.18 | 1.27 |
| Antigua and Barbuda | 90-94 years | 2.18 | 2.42 | 1.35 |
| Zambia | 90-94 years | 2.18 | 2.22 | 1.27 |
| Jordan | 90-94 years | 2.20 | 2.41 | 1.10 |
| San Marino | 90-94 years | 2.20 | 2.30 | 1.24 |
| Syrian Arab Republic | 90-94 years | 2.21 | 2.20 | 1.22 |
| Bosnia and Herzegovina | 90-94 years | 2.32 | 2.62 | 0.95 |
| Palau | 90-94 years | 2.36 | 2.39 | 1.01 |
| Croatia | 90-94 years | 2.39 | 2.44 | 1.45 |
| North Macedonia | 90-94 years | 2.52 | 2.61 | 0.89 |
| Algeria | 90-94 years | 2.57 | 2.57 | 1.17 |
| Greenland | 90-94 years | 2.73 | 4.16 | 1.11 |
| Eswatini | 90-94 years | 2.74 | 2.96 | 1.23 |
| Andorra | 90-94 years | 2.93 | 3.09 | 1.48 |
| American Samoa | 90-94 years | 2.96 | 2.98 | 1.26 |
| Lesotho | 90-94 years | 2.97 | 3.16 | 1.24 |
| Russian Federation | 90-94 years | 3.10 | 4.76 | 1.36 |
| United Arab Emirates | 90-94 years | 3.17 | 3.25 | 1.21 |
| Armenia | 90-94 years | 3.23 | 3.33 | 1.41 |
| Egypt | 90-94 years | 4.07 | 4.07 | 1.20 |
| Northern Mariana Islands | 90-94 years | 4.12 | 4.19 | 1.27 |
| Papua New Guinea | 95+ years | 0.33 | 0.32 | 1.21 |
| Cook Islands | 95+ years | 0.33 | 0.35 | 0.11 |
| Cabo Verde | 95+ years | 0.37 | 0.37 | 1.36 |
| Benin | 95+ years | 0.38 | 0.38 | 1.20 |
| Burkina Faso | 95+ years | 0.41 | 0.40 | 1.19 |
| Guam | 95+ years | 0.42 | 0.38 | 1.32 |
| Chad | 95+ years | 0.43 | 0.42 | 1.16 |
| Sri Lanka | 95+ years | 0.45 | 0.55 | 0.15 |
| Vanuatu | 95+ years | 0.46 | 0.45 | 1.26 |
| Solomon Islands | 95+ years | 0.47 | 0.47 | 1.19 |
| Sierra Leone | 95+ years | 0.47 | 0.47 | 1.16 |
| Niger | 95+ years | 0.47 | 0.47 | 1.15 |
| Myanmar | 95+ years | 0.48 | 0.47 | 1.54 |
| C么te d'Ivoire | 95+ years | 0.48 | 0.48 | 1.23 |
| Niue | 95+ years | 0.48 | 0.49 | 0.19 |
| Seychelles | 95+ years | 0.49 | 0.44 | 1.46 |
| Togo | 95+ years | 0.49 | 0.49 | 1.19 |
| Cambodia | 95+ years | 0.52 | 0.51 | 1.19 |
| Timor-Leste | 95+ years | 0.53 | 0.52 | 1.26 |
| Liberia | 95+ years | 0.53 | 0.53 | 1.14 |
| Senegal | 95+ years | 0.53 | 0.53 | 1.18 |
| Palau | 95+ years | 0.54 | 0.54 | 0.20 |
| Guinea-Bissau | 95+ years | 0.54 | 0.54 | 1.17 |
| Nauru | 95+ years | 0.54 | 0.55 | 0.24 |
| Guinea | 95+ years | 0.54 | 0.54 | 1.16 |
| Lao People's Democratic Republic | 95+ years | 0.56 | 0.55 | 1.27 |
| Gambia | 95+ years | 0.56 | 0.56 | 1.20 |
| Ghana | 95+ years | 0.57 | 0.56 | 1.20 |
| Viet Nam | 95+ years | 0.59 | 0.71 | 0.24 |
| Eswatini | 95+ years | 0.59 | 0.56 | 1.23 |
| Nigeria | 95+ years | 0.61 | 0.60 | 1.22 |
| Mauritania | 95+ years | 0.61 | 0.60 | 1.09 |
| Cameroon | 95+ years | 0.62 | 0.62 | 1.18 |
| Kiribati | 95+ years | 0.64 | 0.63 | 1.23 |
| Marshall Islands | 95+ years | 0.65 | 0.65 | 1.21 |
| Bangladesh | 95+ years | 0.65 | 0.66 | 1.54 |
| Lesotho | 95+ years | 0.68 | 0.66 | 1.25 |
| Tuvalu | 95+ years | 0.70 | 0.71 | 0.29 |
| Micronesia (Federated States of) | 95+ years | 0.70 | 0.70 | 1.23 |
| Samoa | 95+ years | 0.72 | 0.72 | 1.23 |
| Mali | 95+ years | 0.75 | 0.74 | 1.13 |
| Indonesia | 95+ years | 0.77 | 0.81 | 0.16 |
| Tokelau | 95+ years | 0.78 | 0.78 | 0.24 |
| Turkmenistan | 95+ years | 0.78 | 0.78 | 1.29 |
| Botswana | 95+ years | 0.78 | 0.75 | 1.28 |
| Namibia | 95+ years | 0.82 | 0.81 | 1.26 |
| Tonga | 95+ years | 0.88 | 0.88 | 1.26 |
| South Africa | 95+ years | 0.89 | 0.88 | 1.29 |
| Qatar | 95+ years | 0.89 | 0.88 | 1.28 |
| Zimbabwe | 95+ years | 0.95 | 0.95 | 1.22 |
| Angola | 95+ years | 0.95 | 0.96 | 1.04 |
| Georgia | 95+ years | 0.97 | 1.01 | 1.42 |
| Switzerland | 95+ years | 0.97 | 0.97 | 1.15 |
| Gabon | 95+ years | 0.97 | 0.97 | 1.08 |
| Monaco | 95+ years | 0.97 | 0.98 | 1.12 |
| Kyrgyzstan | 95+ years | 0.97 | 0.98 | 1.38 |
| India | 95+ years | 0.98 | 0.99 | 1.40 |
| Maldives | 95+ years | 0.98 | 0.97 | 1.25 |
| Central African Republic | 95+ years | 0.99 | 0.99 | 1.04 |
| Luxembourg | 95+ years | 0.99 | 1.00 | 1.18 |
| Democratic Republic of the Congo | 95+ years | 0.99 | 0.99 | 1.04 |
| Tajikistan | 95+ years | 1.01 | 1.00 | 1.26 |
| Uzbekistan | 95+ years | 1.02 | 1.03 | 1.28 |
| Bhutan | 95+ years | 1.02 | 1.02 | 1.24 |
| Fiji | 95+ years | 1.03 | 1.03 | 1.23 |
| China | 95+ years | 1.03 | 1.05 | 1.00 |
| Guatemala | 95+ years | 1.04 | 1.01 | 1.32 |
| Equatorial Guinea | 95+ years | 1.04 | 1.05 | 1.03 |
| Costa Rica | 95+ years | 1.05 | 1.03 | 1.40 |
| Brunei Darussalam | 95+ years | 1.06 | 1.06 | 0.57 |
| Azerbaijan | 95+ years | 1.06 | 1.06 | 1.34 |
| Taiwan (Province of China) | 95+ years | 1.08 | 1.12 | 0.98 |
| American Samoa | 95+ years | 1.08 | 1.08 | 1.25 |
| Yemen | 95+ years | 1.08 | 1.09 | 1.19 |
| Afghanistan | 95+ years | 1.09 | 1.09 | 1.15 |
| Denmark | 95+ years | 1.09 | 1.09 | 1.57 |
| Congo | 95+ years | 1.10 | 1.10 | 1.02 |
| Panama | 95+ years | 1.10 | 1.09 | 1.36 |
| Paraguay | 95+ years | 1.10 | 1.10 | 1.36 |
| Burundi | 95+ years | 1.11 | 1.11 | 1.24 |
| Colombia | 95+ years | 1.11 | 1.05 | 1.23 |
| Sudan | 95+ years | 1.13 | 1.13 | 1.25 |
| Venezuela (Bolivarian Republic of) | 95+ years | 1.14 | 1.13 | 1.20 |
| North Macedonia | 95+ years | 1.15 | 1.17 | 0.70 |
| Iceland | 95+ years | 1.15 | 1.18 | 0.55 |
| South Sudan | 95+ years | 1.15 | 1.15 | 1.26 |
| Finland | 95+ years | 1.15 | 1.15 | 0.82 |
| Madagascar | 95+ years | 1.16 | 1.16 | 1.23 |
| Sao Tome and Principe | 95+ years | 1.17 | 1.17 | 1.17 |
| Kuwait | 95+ years | 1.18 | 1.26 | 1.07 |
| Iraq | 95+ years | 1.19 | 1.20 | 1.23 |
| Republic of Moldova | 95+ years | 1.20 | 1.20 | 1.22 |
| Ethiopia | 95+ years | 1.21 | 1.21 | 1.29 |
| Serbia | 95+ years | 1.21 | 1.24 | 0.66 |
| Ecuador | 95+ years | 1.23 | 1.23 | 1.34 |
| Nepal | 95+ years | 1.23 | 1.24 | 1.08 |
| Comoros | 95+ years | 1.25 | 1.26 | 1.24 |
| France | 95+ years | 1.27 | 1.29 | 1.17 |
| Portugal | 95+ years | 1.27 | 1.29 | 0.89 |
| Democratic People's Republic of Korea | 95+ years | 1.28 | 1.28 | 1.17 |
| Lebanon | 95+ years | 1.28 | 1.30 | 1.13 |
| Oman | 95+ years | 1.28 | 1.34 | 1.06 |
| Thailand | 95+ years | 1.29 | 1.27 | 1.46 |
| Mauritius | 95+ years | 1.30 | 1.29 | 1.42 |
| Mongolia | 95+ years | 1.30 | 1.31 | 1.29 |
| Tunisia | 95+ years | 1.31 | 1.33 | 1.14 |
| Germany | 95+ years | 1.32 | 1.33 | 1.15 |
| Bulgaria | 95+ years | 1.32 | 1.34 | 0.92 |
| Pakistan | 95+ years | 1.32 | 1.33 | 1.18 |
| Bermuda | 95+ years | 1.34 | 1.38 | 1.29 |
| United States of America | 95+ years | 1.35 | 1.48 | 0.87 |
| Somalia | 95+ years | 1.35 | 1.36 | 1.22 |
| Uganda | 95+ years | 1.36 | 1.37 | 1.29 |
| Djibouti | 95+ years | 1.38 | 1.39 | 1.28 |
| Peru | 95+ years | 1.40 | 1.42 | 1.28 |
| Morocco | 95+ years | 1.41 | 1.42 | 1.17 |
| Ukraine | 95+ years | 1.42 | 1.69 | 1.30 |
| Slovakia | 95+ years | 1.44 | 1.64 | 0.78 |
| Italy | 95+ years | 1.44 | 1.46 | 0.96 |
| Singapore | 95+ years | 1.45 | 1.49 | 0.55 |
| Eritrea | 95+ years | 1.45 | 1.45 | 1.24 |
| Philippines | 95+ years | 1.45 | 1.45 | 1.43 |
| Palestine | 95+ years | 1.47 | 1.49 | 1.24 |
| Saint Vincent and the Grenadines | 95+ years | 1.48 | 1.49 | 1.31 |
| El Salvador | 95+ years | 1.48 | 1.54 | 1.43 |
| Nicaragua | 95+ years | 1.49 | 1.50 | 1.38 |
| Rwanda | 95+ years | 1.49 | 1.50 | 1.25 |
| Malawi | 95+ years | 1.50 | 1.52 | 1.27 |
| Romania | 95+ years | 1.52 | 1.58 | 0.99 |
| Belarus | 95+ years | 1.52 | 1.75 | 1.27 |
| Norway | 95+ years | 1.53 | 1.55 | 1.02 |
| Albania | 95+ years | 1.53 | 1.54 | 1.37 |
| Greenland | 95+ years | 1.55 | 1.75 | 1.01 |
| Algeria | 95+ years | 1.55 | 1.57 | 1.17 |
| Kenya | 95+ years | 1.56 | 1.57 | 1.28 |
| Spain | 95+ years | 1.56 | 1.58 | 1.38 |
| United Republic of Tanzania | 95+ years | 1.58 | 1.59 | 1.27 |
| Poland | 95+ years | 1.58 | 1.65 | 0.63 |
| New Zealand | 95+ years | 1.58 | 1.61 | 0.82 |
| Northern Mariana Islands | 95+ years | 1.58 | 1.59 | 1.26 |
| San Marino | 95+ years | 1.59 | 1.60 | 1.15 |
| Austria | 95+ years | 1.59 | 1.60 | 1.35 |
| Libya | 95+ years | 1.61 | 1.64 | 1.19 |
| Canada | 95+ years | 1.61 | 1.73 | 0.99 |
| Sweden | 95+ years | 1.61 | 1.60 | 1.10 |
| Mozambique | 95+ years | 1.62 | 1.63 | 1.25 |
| Egypt | 95+ years | 1.64 | 1.64 | 1.20 |
| Netherlands | 95+ years | 1.66 | 1.68 | 0.93 |
| Ireland | 95+ years | 1.67 | 1.69 | 1.04 |
| Lithuania | 95+ years | 1.67 | 1.90 | 1.26 |
| United Kingdom | 95+ years | 1.69 | 1.71 | 1.11 |
| Latvia | 95+ years | 1.73 | 2.23 | 1.26 |
| Haiti | 95+ years | 1.74 | 1.76 | 1.22 |
| Brazil | 95+ years | 1.75 | 1.77 | 1.50 |
| Syrian Arab Republic | 95+ years | 1.75 | 1.75 | 1.23 |
| Jamaica | 95+ years | 1.77 | 1.87 | 1.26 |
| Republic of Korea | 95+ years | 1.77 | 1.81 | 0.56 |
| Israel | 95+ years | 1.80 | 1.79 | 0.99 |
| Bahamas | 95+ years | 1.81 | 1.97 | 1.35 |
| Bolivia (Plurinational State of) | 95+ years | 1.83 | 1.87 | 1.22 |
| Zambia | 95+ years | 1.86 | 1.90 | 1.28 |
| Malta | 95+ years | 1.87 | 1.84 | 1.01 |
| Australia | 95+ years | 1.87 | 1.91 | 0.89 |
| Puerto Rico | 95+ years | 1.88 | 3.05 | 1.32 |
| Bosnia and Herzegovina | 95+ years | 1.90 | 2.16 | 0.83 |
| United States Virgin Islands | 95+ years | 1.92 | 2.15 | 1.35 |
| Estonia | 95+ years | 1.95 | 2.46 | 1.29 |
| Kazakhstan | 95+ years | 1.99 | 2.07 | 0.95 |
| Saint Kitts and Nevis | 95+ years | 2.00 | 2.41 | 1.38 |
| Mexico | 95+ years | 2.01 | 2.23 | 1.25 |
| Japan | 95+ years | 2.01 | 2.00 | 0.92 |
| Croatia | 95+ years | 2.03 | 2.07 | 1.03 |
| Honduras | 95+ years | 2.05 | 2.13 | 1.24 |
| Hungary | 95+ years | 2.09 | 2.24 | 0.72 |
| Belize | 95+ years | 2.13 | 2.30 | 1.33 |
| Cyprus | 95+ years | 2.13 | 2.13 | 1.46 |
| Cuba | 95+ years | 2.16 | 2.26 | 1.35 |
| Belgium | 95+ years | 2.23 | 2.27 | 1.53 |
| Slovenia | 95+ years | 2.27 | 2.33 | 0.98 |
| Malaysia | 95+ years | 2.28 | 2.31 | 1.39 |
| Bahrain | 95+ years | 2.31 | 2.51 | 1.14 |
| Montenegro | 95+ years | 2.32 | 2.43 | 0.79 |
| Saudi Arabia | 95+ years | 2.37 | 2.66 | 1.13 |
| Turkiye | 95+ years | 2.42 | 2.62 | 1.16 |
| Trinidad and Tobago | 95+ years | 2.44 | 2.68 | 1.31 |
| Iran (Islamic Republic of) | 95+ years | 2.47 | 2.49 | 1.27 |
| Dominica | 95+ years | 2.49 | 2.62 | 1.32 |
| Czechia | 95+ years | 2.54 | 2.65 | 0.80 |
| United Arab Emirates | 95+ years | 2.55 | 2.54 | 1.21 |
| Jordan | 95+ years | 2.62 | 2.90 | 1.13 |
| Suriname | 95+ years | 2.68 | 3.16 | 1.33 |
| Uruguay | 95+ years | 2.78 | 3.07 | 1.07 |
| Russian Federation | 95+ years | 2.79 | 4.42 | 1.24 |
| Dominican Republic | 95+ years | 2.89 | 3.14 | 1.35 |
| Guyana | 95+ years | 2.95 | 3.19 | 1.31 |
| Saint Lucia | 95+ years | 3.06 | 3.18 | 1.36 |
| Barbados | 95+ years | 3.21 | 3.61 | 1.32 |
| Andorra | 95+ years | 3.32 | 3.41 | 1.26 |
| Argentina | 95+ years | 3.32 | 3.33 | 1.36 |
| Antigua and Barbuda | 95+ years | 3.43 | 3.92 | 1.35 |
| Greece | 95+ years | 3.84 | 3.86 | 0.45 |
| Armenia | 95+ years | 3.99 | 4.03 | 1.41 |
| Chile | 95+ years | 4.26 | 4.41 | 1.17 |
| Grenada | 95+ years | 6.87 | 5.99 | 1.30 |
| Abbreviations: ASDR: Age-standardized disability-adjusted life-years (DALYs) rate per 100,000. ASMR: Age-standardized mortality rate per 100,000. ASPR: Age-standardized prevalence rate per 100,000. | | | | |
