# Supplementary material for: Sex difference in the burden of rheumatic heart disease: Insights from the Global Burden of Disease Study 2021
Source: PLoS One. 2025 Oct 22;20(10):e0334914. doi: 10.1371/journal.pone.0334914 (PMC12543145; doi:10.1371/journal.pone.0334914)
Supplement: S8 Table — (DOCX) [file pone.0334914.s010.docx]

**S8 Table:** Global Extreme Values of Female/Male in 2021 across 204 countries.

| **Age Group** | **The Lowest Female/Male** | | | **The Highest Female/Male** | | |
| --- | --- | --- | --- | --- | --- | --- |
|  | **ASDR** | **ASMR** | **ASPR** | **ASDR** | **ASMR** | **ASPR** |
| <5 years | Ukraine (0.49) | Ukraine (0.18) | Greece (0.69) | Greenland (3.73) | Greenland (5.34) | Tokelau (1.80) |
| 10-14 years | United States Virgin Islands (0.20) | United States Virgin Islands (0.01) | Greece (0.62) | Tokelau (3.65) | Slovenia (12.00) | Tokelau (1.61) |
| 15-19 years | United States Virgin Islands (0.05) | United States Virgin Islands (0.01) | Greece (0.61) | Estonia (2.38) | Greenland (3.59) | Tokelau (1.58) |
| 20-24 years | Cook Islands (0.28) | Guam (0.15) | Greece (0.62) | Bahrain (2.03) | Honduras (2.87) | Tokelau (1.74) |
| 25-29 years | Brunei Darussalam (0.16) | Brunei Darussalam (0.12) | Greece (0.62) | Greenland (3.24) | Greenland (3.83) | Tokelau (1.96) |
| 30-34 years | Palau (0.44) | Cook Islands (0.43) | Greece (0.62) | Tokelau (2.49) | Antigua and Barbuda (3.45) | New Zealand (2.39) |
| 35-39 years | Palau (0.37) | Norway (0.32) | Greece (0.65) | Greenland (2.53) | Honduras (3.78) | Australia (2.31) |
| 40-44 years | Poland (0.39) | Cabo Verde (0.35) | Greece (0.71) | United States Virgin Islands (3.47) | Antigua and Barbuda (4.17) | Australia (2.29) |
| 45-49 years | Palau (0.31) | Palau (0.30) | Poland (0.70) | Andorra (2.78) | Honduras (3.54) | Australia (2.15) |
| 50-54 years | Palau (0.29) | Palau (0.28) | Poland (0.71) | Saudi Arabia (3.03) | Honduras (4.17) | Brunei Darussalam (2.01) |
| 55-59 years | Palau (0.48) | Palau (0.45) | Sri Lanka (0.72) | Sao Tome and Principe (3.43) | Sao Tome and Principe (4.45) | Brunei Darussalam (2.24) |
| 5-9 years | Norway (0.31) | Guam (0.04) | Greece (0.64) | Slovenia (2.78) | Seychelles (18.86) | Tokelau (1.69) |
| 60-64 years | Palau (0.31) | Palau (0.28) | Sri Lanka (0.70) | Andorra (3.35) | Andorra (3.63) | Brunei Darussalam (2.33) |
| 65-69 years | Cook Islands (0.35) | Cook Islands (0.28) | Sri Lanka (0.62) | Andorra (3.50) | Andorra (3.83) | Andorra (2.37) |
| 70-74 years | Cook Islands (0.41) | Cook Islands (0.36) | Sri Lanka (0.53) | United Arab Emirates (5.50) | United Arab Emirates (6.06) | Andorra (2.38) |
| 75-79 years | Cook Islands (0.37) | Cook Islands (0.31) | Sri Lanka (0.45) | United Arab Emirates (6.75) | United Arab Emirates (7.04) | Andorra (2.25) |
| 80-84 years | Papua New Guinea (0.35) | Guam (0.23) | Sri Lanka (0.38) | United Arab Emirates (4.55) | United Arab Emirates (4.80) | Qatar (2.34) |
| 85-89 years | Papua New Guinea (0.28) | Guam (0.23) | Sri Lanka (0.31) | United Arab Emirates (5.33) | United Arab Emirates (5.41) | Qatar (2.08) |
| 90-94 years | Sri Lanka (0.63) | Seychelles (0.62) | Indonesia (0.21) | Northern Mariana Islands (4.12) | Russian Federation (4.76) | Denmark (1.66) |
| 95+ years | Papua New Guinea (0.33) | Papua New Guinea (0.32) | Cook Islands (0.11) | Grenada (6.87) | Grenada (5.99) | Denmark (1.57) |
| Abbreviations: ASDR: Age-standardized disability-adjusted life-years (DALYs) rate per 100,000. ASMR: Age-standardized mortality rate per 100,000. ASPR: Age-standardized prevalence rate per 100,000. | | | | | | |
|  |  |  |  |  |  |  |
|  |  |  |  |  |  |  |
